# Global, regional, and national age-sex-specific burden of diarrhoeal diseases, their risk factors, and aetiologies, 1990–2021, for 204 countries and territories: a systematic analysis for the Global Burden of Disease Study 2021

**DOI:** 10.1016/S1473-3099(24)00691-1

**Published:** 2025-05

**Authors:** GBD 2021 Diarrhoeal Diseases Collaborators, Hmwe Hmwe Kyu, Avina Vongpradith, Regina-Mae Villanueva Dominguez, Jianing Ma, Samuel B Albertson, Amanda Novotney, Ibrahim A Khalil, Christopher E Troeger, Matthew C Doxey, Jorge R Ledesma, Sarah Brooke Sirota, Rose Grace Bender, Lucien R Swetschinski, Matthew Cunningham, Sandra Spearman, Yohannes Habtegiorgis Abate, Abdallah H A Abd Al Magied, Samar Abd ElHafeez, Meriem Abdoun, Bayeh Abera, Hassan Abidi, Richard Gyan Aboagye, Yonas Derso Abtew, Hasan Abualruz, Eman Abu-Gharbieh, Hana J Abukhadijah, Salahdein Aburuz, Isaac Yeboah Addo, Victor Adekanmbi, Charles Oluwaseun Oluwaseun Adetunji, Temitayo Esther Adeyeoluwa, Ripon Kumar Adhikary, Qorinah Estiningtyas Sakilah Adnani, Saryia Adra, Leticia Akua Adzigbli, Aanuoluwapo Adeyimika Afolabi, Muhammad Sohail Afzal, Saira Afzal, Suneth Buddhika Agampodi, Feleke Doyore Agide, Bright Opoku Ahinkorah, Aqeel Ahmad, Sajjad Ahmad, Ali Ahmed, Ayman Ahmed, Haroon Ahmed, Saeed Ahmed, Karolina Akinosoglou, Ema Akter, Salah Al Awaidy, Muaaz M Alajlani, Khurshid Alam, Almaza Albakri, Mohammed Albashtawy, Wafa A Aldhaleei, Abdelazeem M Algammal, Adel Ali Saeed Al-Gheethi, Abid Ali, Syed Shujait Ali, Waad Ali, Sheikh Mohammad Alif, Syed Mohamed Aljunid, Sabah Al-Marwani, Joseph Uy Almazan, Hesham M Al-Mekhlafi, Sami Almustanyir, Saleh A Alqahatni, Ahmad Alrawashdeh, Rami H Al-Rifai, Mohammed A Alsabri, Awais Altaf, Khalid A Altirkawi, Nelson Alvis-Guzman, Nelson J Alvis-Zakzuk, Mohammad Sharif Ibrahim Alyahya, Walid A Al-Zyoud, Dickson A Amugsi, Catalina Liliana Andrei, Sebastien Antoni, Boluwatife Stephen Anuoluwa, Iyadunni Adesola Anuoluwa, Saleha Anwar, Palwasha Anwari, Geminn Louis Carace Apostol, Jalal Arabloo, Mosab Arafat, Aleksandr Y Aravkin, Demelash Areda, Brhane Berhe Aregawi, Abdulfatai Aremu, Michael Benjamin Arndt, Akeza Awealom Asgedom, Tahira Ashraf, Seyyed Shamsadin Athari, Alok Atreya, Firayad Ayele, Davood Azadi, Gulrez Shah Azhar, Shahkaar Aziz, Ahmed Y. Azzam, Giridhara Rathnaiah Babu, Pegah Bahrami Taghanaki, Saeed Bahramian, Senthilkumar Balakrishnan, Biswajit Banik, Simachew Animen Bante, Mainak Bardhan, Till Winfried Bärnighausen, Hiba Jawdat Barqawi, Amadou Barrow, Zarrin Basharat, Quique Bassat, Mohammad-Mahdi Bastan, Saurav Basu, Prapthi Persis Bathini, Payam Behzadi, Maryam Beiranvand, Muhammad Bashir Bello, Olorunjuwon Omolaja Bello, Apostolos Beloukas, Azizullah Beran, Dinesh Bhandari, Pankaj Bhardwaj, Zulfiqar A Bhutta, Hamed Borhany, Souad Bouaoud, Michael Brauer, Danilo Buonsenso, Zahid A Butt, Mehtap Çakmak Barsbay, Luis Alberto Cámera, Angelo Capodici, Carlos A Castañeda-Orjuela, Muthia Cenderadewi, Chiranjib Chakraborty, Sandip Chakraborty, Vijay Kumar Chattu, Anis Ahmad Chaudhary, Fatemeh Chichagi, Patrick R Ching, Jesus Lorenzo Chirinos-Caceres, Hitesh Chopra, Sonali Gajanan Choudhari, Enayet Karim Chowdhury, Dinh-Toi Chu, Isaac Sunday Chukwu, Muhammad Chutiyami, Natalia Cruz-Martins, Omid Dadras, Xiaochen Dai, Lalit Dandona, Rakhi Dandona, Samuel Demissie Darcho, Jai K Das, Nihar Ranjan Dash, Ivan Delgado-Enciso, Belay Desye, Vinoth Gnana Chellaiyan Devanbu, Kuldeep Dhama, Meghnath Dhimal, Michael J Diaz, Thanh Chi Do, Sushil Dohare, Fariba Dorostkar, Ojas Prakashbhai Doshi, Leila Doshmangir, Haneil Larson Dsouza, Senbagam Duraisamy, Oyewole Christopher Durojaiye, Abdel Rahman E'mar, Abdelaziz Ed-Dra, Hisham Atan Edinur, Defi Efendi, Ferry Efendi, Foolad Eghbali, Temitope Cyrus Ekundayo, Iman El Sayed, Muhammed Elhadi, Ashraf A El-Metwally, Mohammed Elshaer, Ibrahim Elsohaby, Chadi Eltaha, Babak Eshrati, Majid Eslami, Ayesha Fahim, Ildar Ravisovich Fakhradiyev, Aliasghar Fakhri-Demeshghieh, Mohammad Farahmand, Folorunso Oludayo Fasina, Modupe Margaret Fasina, Alireza Feizkhah, Ginenus Fekadu, Nuno Ferreira, Getahun Fetensa, Florian Fischer, Takeshi Fukumoto, Blima Fux, Muktar A Gadanya, Santosh Gaihre, Márió Gajdács, Yaseen Galali, Aravind P Gandhi, Rupesh K Gautam, Miglas Welay Gebregergis, Mesfin Gebrehiwot, Teferi Gebru Gebremeskel, Motuma Erena Getachew, Genanew K Getahun, Molla Getie, Afsaneh Ghasemzadeh, Ramy Mohamed Ghazy, Sherief Ghozy, Artyom Urievich Gil, Alem Abera Girmay, Abraham Tamirat T Gizaw, Mahaveer Golechha, Pouya Goleij, Philimon N Gona, Ayman Grada, Giovanni Guarducci, Mesay Dechasa Gudeta, Vivek Kumar Gupta, Awoke Derbie Habteyohannes, Najah R Hadi, Samer Hamidi, Erin B Hamilton, Harapan Harapan, Md. Kamrul Hasan, S.M. Mahmudul Hasan, Hamidreza Hasani, Md Saquib Hasnain, Ikrama Ibrahim Hassan, Jiawei He, Mehdi Hemmati, Kamal Hezam, Mehdi Hosseinzadeh, Junjie Huang, Hong-Han Huynh, Segun Emmanuel Ibitoye, Kevin S Ikuta, Olayinka Stephen Ilesanmi, Irena M Ilic, Milena D Ilic, Sumant Inamdar, Mustafa Alhaji Isa, Md. Rabiul Islam, Sheikh Mohammed Shariful Islam, Nahlah Elkudssiah Ismail, Chidozie Declan Iwu, Kathryn H Jacobsen, Haitham Jahrami, Akhil Jain, Nityanand Jain, Ammar Abdulrahman Jairoun, Mihajlo Jakovljevic, Reza Jalilzadeh Yengejeh, Javad Javidnia, Shubha Jayaram, Mohammad Jokar, Jost B Jonas, Abel Joseph, Nitin Joseph, Jacek Jerzy Jozwiak, Hannaneh Kabir, Dler H. Hussein Kadir, Md Moustafa Kamal, Vineet Kumar Kamal, Arun Kamireddy, Tanuj Kanchan, Kehinde Kazeem Kanmodi, Suthanthira Kannan S, Rami S Kantar, Jafar Karami, Prabin Karki, Hengameh Kasraei, Harkiran Kaur, Mohammad Keykhaei, Yousef Saleh Khader, Alireza Khalilian, Faham Khamesipour, Gulfaraz Khan, Mohammad Jobair Khan, Zeeshan Ali Khan, Vishnu Khanal, Khaled Khatab, Moawiah Mohammad Khatatbeh, Amir M Khater, Khalid A Kheirallah, Feriha Fatima Khidri, Atulya Aman Khosla, Kwanghyun Kim, Yun Jin Kim, Adnan Kisa, Niranjan Kissoon, Desmond Klu, Sonali Kochhar, Ali-Asghar Kolahi, Farzad Kompani, Soewarta Kosen, Kewal Krishan, Barthelemy Kuate Defo, Md Abdul Kuddus, Mohammed Kuddus, Mukhtar Kulimbet, G Anil Kumar, Rakesh Kumar, Frank Kyei-Arthur, Chandrakant Lahariya, Dharmesh Kumar Lal, Nhi Huu Hanh Le, Seung Won Lee, Wei-Chen Lee, Yeong Yeh Lee, Ming-Chieh Li, Virendra S Ligade, Gang Liu, Shuke Liu, Xiaofeng Liu, Xuefeng Liu, Chun-Han Lo, Giancarlo Lucchetti, Lei Lv, Kashish Malhotra, Ahmad Azam Malik, Bishnu P Marasini, Miquel Martorell, Roy Rillera Marzo, Hossein Masoumi-Asl, Medha Mathur, Navgeet Mathur, Rishi P Mediratta, Elahe Meftah, Tesfahun Mekene Meto, Hadush Negash Meles, Endalkachew Belayneh Melese, Walter Mendoza, Mohsen Merati, Tuomo J Meretoja, Tomislav Mestrovic, Sachith Mettananda, Le Huu Nhat Minh, Vinaytosh Mishra, Prasanna Mithra, Ashraf Mohamadkhani, Ahmed Ismail Mohamed, Mouhand F H Mohamed, Nouh Saad Mohamed, Mustapha Mohammed, Shafiu Mohammed, Lorenzo Monasta, Mohammad Ali Moni, Rohith Motappa, Vincent Mougin, Sumaira Mubarik, Francesk Mulita, Kavita Munjal, Yanjinlkham Munkhsaikhan, Pirouz Naghavi, Gurudatta Naik, Tapas Sadasivan Nair, Hastyar Hama Rashid Najmuldeen, Shumaila Nargus, Delaram Narimani Davani, Abdulqadir J Nashwan, Zuhair S Natto, Athare Nazri-Panjaki, G Takop Nchanji, Pacifique Ndishimye, Josephine W Ngunjiri, Duc Hoang Nguyen, Nhien Ngoc Y Nguyen, Van Thanh Nguyen, Yeshambel T Nigatu, Ali Nikoobar, Vikram Niranjan, Chukwudi A Nnaji, Efaq Ali Noman, Nurulamin M Noor, Syed Toukir Ahmed Noor, Mehran Nouri, Majid Nozari, Chisom Adaobi Nri-Ezedi, Fred Nugen, Ismail A Odetokun, Adesola Adenike Ogunfowokan, Tolulope R Ojo-Akosile, Iruka N Okeke, Akinkunmi Paul Okekunle, Abdulhakeem Abayomi Olorukooba, Isaac Iyinoluwa Olufadewa, Gideon Olamilekan Oluwatunase, Verner N Orish, Doris V Ortega-Altamirano, Esteban Ortiz-Prado, Uchechukwu Levi Osuagwu, Olayinka Osuolale, Amel Ouyahia, Jagadish Rao Padubidri, Anamika Pandey, Ashok Pandey, Victoria Pando-Robles, Shahina Pardhan, Romil R Parikh, Jay Patel, Shankargouda Patil, Shrikant Pawar, Prince Peprah, Arokiasamy Perianayagam, Simone Perna, Ionela-Roxana Petcu, Anil K Philip, Roman V Polibin, Maarten J Postma, Naeimeh Pourtaheri, Jalandhar Pradhan, Elton Junio Sady Prates, Dimas Ria Angga Pribadi, Nameer Hashim Qasim, Asma Saleem Qazi, Deepthi R, Venkatraman Radhakrishnan, Fakher Rahim, Mosiur Rahman, Muhammad Aziz Rahman, Shayan Rahmani, Mohammad Rahmanian, Nazanin Rahmanian, Mahmoud Mohammed Ramadan, Shakthi Kumaran Ramasamy, Sheena Ramazanu, Muhammed Ahmed Ahmed Rameto, Pramod W Ramteke, Kritika Rana, Chhabi Lal Ranabhat, Davide Rasella, Mohammad-Mahdi Rashidi, Ashkan Rasouli-Saravani, Devarajan Rathish, Santosh Kumar Rauniyar, Salman Rawaf, Elrashdy Moustafa Mohamed Redwan, Aavishkar Raj Regmi, Kannan RR Rengasamy, Nazila Rezaei, Nima Rezaei, Mohsen Rezaeian, Abanoub Riad, Monica Rodrigues, Jefferson Antonio Buendia Rodriguez, Leonardo Roever, Ravi Rohilla, Luca Ronfani, Moustaq Karim Khan Rony, Allen Guy Ross, Shekoufeh Roudashti, Bedanta Roy, Tilleye Runghien, Mamta Sachdeva Dhingra, Basema Ahmad Saddik, Erfan Sadeghi, Mehdi Safari, Soumya Swaroop Sahoo, S. Mohammad Sajadi, Afeez Abolarinwa Salami, Mohamed A Saleh, Hossein Samadi Kafil, Yoseph Leonardo Samodra, Juan Sanabria, Rama Krishna Sanjeev, Tanmay Sarkar, Benn Sartorius, Brijesh Sathian, Maheswar Satpathy, Monika Sawhney, Austin E Schumacher, Mengistu Abayneh Sebsibe, Dragos Serban, Mahan Shafie, Samiah Shahid, Wajeehah Shahid, Masood Ali Shaikh, Sunder Sham, Muhammad Aaqib Shamim, Mehran Shams-Beyranvand, Mohammad Ali Shamshirgaran, Mohd Shanawaz, Mohammed Shannawaz, Amin Sharifan, Manoj Sharma, Vishal Sharma, Suchitra M Shenoy, Samendra P Sherchan, Mahabalesh Shetty, Pavanchand H Shetty, Desalegn Shiferaw, Aminu Shittu, Seyed Afshin Shorofi, Emmanuel Edwar Siddig, Luís Manuel Lopes Rodrigues Silva, Baljinder Singh, Jasvinder A Singh, Robert Sinto, Bogdan Socea, Heidi M Soeters, Anton Sokhan, Prashant Sood, Soroush Soraneh, Chandrashekhar T Sreeramareddy, Suresh Kumar Srinivasamurthy, Vijay Kumar Srivastava, Muhammad Haroon Stanikzai, Narayan Subedi, Vetriselvan Subramaniyan, Sahabi K Sulaiman, Muhammad Suleman, Chandan Kumar Swain, Lukasz Szarpak, Sree Sudha T Y, Seyyed Mohammad Tabatabaei, Celine Tabche, Zanan Mohammed-Ameen Taha, Ashis Talukder, Jacques Lukenze Tamuzi, Ker-Kan Tan, Sarmila Tandukar, Mohamad-Hani Temsah, Ocean Thakali, Ramna Thakur, Sathish Thirunavukkarasu, Joe Thomas, Nikhil Kenny Thomas, Jansje Henny Vera Ticoalu, Krishna Tiwari, Marcos Roberto Tovani-Palone, Khai Hoan Tram, An Thien Tran, Nghia Minh Tran, Thang Huu Tran, Samuel Joseph Tromans, Thien Tan Tri Tai Truyen, Munkhtuya Tumurkhuu, Aniefiok John Udoakang, Arit Udoh, Saeed Ullah, Muhammad Umair, Muhammad Umar, Brigid Unim, Bhaskaran Unnikrishnan, Sanaz Vahdati, Asokan Govindaraj Vaithinathan, Rohollah Valizadeh, Madhur Verma, Georgios-Ioannis Verras, Manish Vinayak, Yasir Waheed, Mandaras Tariku Walde, Yanzhong Wang, Muhammad Waqas, Kosala Gayan Weerakoon, Nuwan Darshana Wickramasinghe, Asrat Arja Wolde, Felicia Wu, Sajad Yaghoubi, Sanni Yaya, Saber Yezli, Vahit Yiğit, Dehui Yin, Dong Keon Yon, Naohiro Yonemoto, Hadiza Yusuf, Mondal Hasan Zahid, Fathiah Zakham, Leila Zaki, Iman Zare, Michael Zastrozhin, Mohammed G M Zeariya, Haijun Zhang, Zhi-Jiang Zhang, Abzal Zhumagaliuly, Hafsa Zia, Mohammad Zoladl, Ali H Mokdad, Stephen S Lim, Theo Vos, James A Platts-Mills, Jonathan F Mosser, Robert C Reiner, Simon I Hay, Mohsen Naghavi, Christopher J L Murray

## Abstract

**Background:**

Diarrhoeal diseases claim more than 1 million lives annually and are a leading cause of death in children younger than 5 years. Comprehensive global estimates of the diarrhoeal disease burden for specific age groups of children younger than 5 years are scarce, and the burden in children older than 5 years and in adults is also understudied. We used results from the Global Burden of Diseases, Injuries, and Risk Factors Study 2021 to assess the burden of, and trends in, diarrhoeal diseases overall and attributable to 13 pathogens, as well as the contributions of associated risk factors, in children and adults in 204 countries and territories from 1990 to 2021.

**Methods:**

We used the Cause of Death Ensemble modelling strategy to analyse vital registration data, verbal autopsy data, mortality surveillance data, and minimally invasive tissue sampling data. We used DisMod-MR (version 2.1), a Bayesian meta-regression tool, to analyse incidence and prevalence data identified via systematic reviews, population-based surveys, and claims and inpatient data. We calculated diarrhoeal disability-adjusted life-years (DALYs) as the sum of years of life lost (YLLs) and years lived with disability (YLDs) for each location, year, and age–sex group. For aetiology estimation, we used a counterfactual approach to quantify population-attributable fractions (PAFs). Additionally, we estimated the diarrhoeal disease burden attributable to the independent effects of risk factors using the comparative risk assessment framework.

**Findings:**

In 2021, diarrhoeal diseases caused an estimated 1·17 million (95% uncertainty interval 0·793–1·62) deaths globally, representing a 60·3% (50·6–69·0) decrease since 1990 (2·93 million [2·31–3·73] deaths). The most pronounced decline was in children younger than 5 years, with a 79·2% (72·4–84·6) decrease in diarrhoeal deaths. Global YLLs also decreased substantially, from 186 million (147–221) in 1990 to 51·4 million (39·9–65·9) in 2021. In 2021, an estimated 59·0 million (47·2–73·2) DALYs were attributable to diarrhoeal diseases globally, with 30·9 million (23·1–42·0) of these affecting children younger than 5 years. Leading risk factors for diarrhoeal DALYs included low birthweight and short gestation in the neonatal age groups, child growth failure in children aged between 1–5 months and 2–4 years, and unsafe water and poor sanitation in older children and adults. We estimated that the removal of all evaluated diarrhoeal risk factors would reduce global DALYs from 59·0 million (47·2–73·2) to 4·99 million (1·99–10·0) among all ages combined. Globally in 2021, rotavirus was the predominant cause of diarrhoeal deaths across all ages, with a PAF of 15·2% (11·4–20·1), followed by norovirus at 10·6% (2·3–17·0) and *Cryptosporidium* spp at 10·2% (7·03–14·3). In children younger than 5 years, the fatal PAF of rotavirus was 35·2% (28·7–43·0), followed by *Shigella* spp at 24·0% (15·2–37·9) and adenovirus at 23·8% (14·8–36·3). Other pathogens with a fatal PAF greater than 10% in children younger than 5 years included *Cryptosporidium spp*, *typical enteropathogenicEscherichia coli*, and enterotoxigenic *E coli* producing heat-stable toxin.

**Interpretation:**

The substantial decline in the global burden of diarrhoeal diseases since 1990, particularly in children younger than 5 years, supports the effectiveness of health interventions such as oral rehydration therapy, enhanced water, sanitation, and hygiene (WASH) infrastructure, and the introduction and scale-up of rotavirus vaccination. Targeted interventions and preventive measures against key risk factors and pathogens could further reduce this burden. Continued investment in the development and distribution of vaccines for leading pathogens remains crucial.

**Funding:**

Bill & Melinda Gates Foundation.

## Introduction

Diarrhoeal diseases, caused by various pathogens such as bacteria and viruses, are a major public health issue worldwide, responsible for more than 1 million deaths each year.^1^ These diseases are among the leading causes of death in children younger than 5 years, particularly in low-income and middle-income countries.^2^ Recognising this crucial issue, global health initiatives such as WHO and UNICEF's Global Action Plan for the Prevention and Control of Pneumonia and Diarrhoea have been established, setting ambitious goals such as ending preventable deaths from diarrhoeal diseases in children younger than 5 years by 2025.^3^ Available evidence indicates the uneven distribution of the diarrhoeal disease burden in children younger than 5 years, potentially due to factors such as differences in age-specific susceptibility and immune system development.^4^, ^5^, ^6^ Although a granular understanding of the age-specific burden can enhance refinement and prioritisation of interventions towards achieving the global goals, existing literature^7^ on the global burden of diarrhoeal diseases has predominantly reported combined results for all children younger than 5 years as a homogeneous group. Whereas diarrhoeal episodes tend to be milder in older children and adults,^8^ the diarrhoeal burden continues to put a strain on economies,^9^, ^10^ health-care systems, and the overall health of communities around the world.^11^ Although previous studies have highlighted the growing burden of diarrhoeal diseases in older people,^2^, ^12^ the burden in older children and adult age groups has not been comprehensively studied. Expanding the analytical focus to include both granular age groups aged younger than 5 years along with those aged 5 years and older (child and adult age groups) aligns with Sustainable Development Goal 3 of ensuring healthy lives and promoting wellbeing for all ages.^13^

**Research in context**

**Evidence before this study**

The burden of diarrhoeal diseases, risk factors, and aetiologies in children younger than 5 years, often considered a homogeneous group, has been extensively studied by several groups, including WHO and the Maternal and Child Epidemiology Estimation Group (WHO-MCEE) and the Global Burden of Diseases, Injuries, and Risk Factors Study (GBD). The GBD 2017 diarrhoeal diseases study evaluated the impact of risk factors and interventions on the burden of diarrhoeal diseases in children younger than 5 years across 195 countries and territories. Evidence suggests that the diarrhoeal disease burden might vary within this age group due to factors such as differences in age-specific susceptibility and immune system development. Yet, global diarrhoeal disease burden estimates for specific age groups among children younger than 5 years, attributed to various risk factors and pathogens, have been largely unavailable due to studies aggregating data for all children younger than 5 years. We searched PubMed using the terms “diarrhea”[MeSH] AND (“burden” OR “estimates”) AND (“age” OR “sex” OR “gender”) AND “global” AND “risk”, without applying any language restrictions, for articles published from Jan 1, 1990, to Jan 27, 2024. The search yielded 70 studies. We did not identify any studies that evaluated global levels of, and trends in, diarrhoeal disease burden and corresponding risk factors by granular age groups in children younger than 5 years, or in older children and adults by age and sex.

**Added value of this study**

GBD 2021 included new data sources, compared with previous GBD iterations (including GBD 2019 and GBD 2017), for diarrhoeal mortality and morbidity and corresponding aetiologies, including pathogen-specific data from the Global Pediatric Diarrhoea Surveillance Network. This study also differentiated the specific burdens of enterotoxigenic *Escherichia coli* producing heat-stable toxin (ST-ETEC) and typical enteropathogenic *E coli* (tEPEC), which were previously aggregated as all ETEC and all EPEC, respectively, in past GBD publications focusing on diarrhoeal diseases. Our study examines the burden of, and trends in, diarrhoeal diseases in children younger than 5 years across nuanced age categories and expands the scope of previous research by assessing the burden of diarrhoea in older children and adults, as well as analysing the burden attributable to risk factors by more granular age groups. Last, we provide risk-deleted burden estimates for the first time, representing the diarrhoeal disease burden that would occur if the effects of all evaluated risk factors were removed.

**Implications of all the available evidence**

The results of our study highlight the considerable progress made in reducing the burden of diarrhoeal diseases worldwide, with the most notable improvements observed in children younger than 5 years. This success reflects the effectiveness of concerted public health interventions, including the water, sanitation, and hygiene (WASH) initiative, rotavirus vaccination, and oral rehydration therapy. Our study underscores the important role of continued investment and innovation in vaccine development for leading pathogens and emphasises the importance of implementing comprehensive public health strategies that encompass improvements in WASH practices, nutrition, and access to health-care services. Effective implementation of these strategies could substantially accelerate the decline in the global burden of diarrhoeal diseases, particularly in the most vulnerable populations, and help bridge the gap in health disparities across different regions.

Previous research reports diarrhoea as the fifth leading cause of death among children younger than 5 years, with rotavirus as the leading aetiology for diarrhoea mortality among both children aged younger than 5 years and all ages combined.^2^ Although the results presented in this study continue to report rotavirus as a leading pathogen, it is important to explore other aetiologies with a high burden. Previous publications of the Global Burden of Diseases, Injuries, and Risk Factors Study (GBD) focused on diarrhoeal diseases aggregated data for the heat-stable toxin (ST) and heat-labile toxin (LT) genotypes of enterotoxigenic *Escherichia coli* (ETEC).^2^, ^14^ However, the Global Enteric Multicenter Study (GEMS) showed that ETEC strains producing ST, whether alone or alongside LT, substantially contributed to moderate-to-severe diarrhoea in children in low-income to middle-income countries.^15^ GEMS also indicated that both ST-ETEC and typical enteropathogenic *E coli* (tEPEC) strains were associated with an increased risk of diarrhoeal mortality in infants after adjusting for other pathogens and study sites,^15^ yet these were grouped under ETEC and all EPEC, respectively, in previous GBD studies.^2^, ^14^

To address crucial gaps in our understanding of diarrhoeal diseases, we used data from GBD 2021 to assess the burden of, and trends in, diarrhoeal diseases and risk factors across all age groups, including granular age groups in children younger than 5 years, by sex, for 204 countries and territories from 1990 to 2021. We also aimed to assess the burden of diarrhoeal diseases attributable to 13 pathogens, including ST-ETEC and tEPEC separately, to better assess their individual burdens and inform targeted interventions against pathogen-specific diarrhoeal diseases. Additionally, by reporting the risk-deleted burden, we offer an opportunity to assess what the theoretical burden of diarrhoea might be in ideal scenarios, in which the impact of risk factors has been removed. This manuscript was produced as part of the GBD Collaborator Network and in accordance with the GBD Protocol.

## Methods

### Overview

GBD is a systematic, scientific effort to quantify the comparative magnitude of health loss caused by diseases, injuries, and risk factors by age, sex, and geography over time. The GBD geographical hierarchy includes 204 countries and territories, which are grouped into 21 regions based on epidemiological similarities and geographical closeness. These regions are further aggregated into seven super-regions according to cause-of-death patterns. Detailed methods for GBD have been published elsewhere.^1^, ^16^ Here, we describe the methods and estimation strategies used for diarrhoeal diseases and their corresponding risk factors and pathogens.

### Burden of diarrhoeal diseases overall and due to specific aetiologies

Input data for estimating mortality from diarrhoeal diseases comprised vital registration data spanning 24 181 site-years (ie, the total number of years of data available for all locations with data), 825 site-years of sample-based vital registration data, 1785 site-years of verbal autopsy data, 575 site-years of mortality surveillance data, and nine site-years of minimally invasive tissue sampling data. The data were adjusted for incomplete death registration and garbage coding.^1^, ^17^ Using these input data, we estimated diarrhoeal disease mortality using the Cause of Death Ensemble modelling (CODEm) strategy.^1^, ^18^ This approach involves analysing a diverse array of sub-models, each using different combinations of predictive covariates, such as access to improved water sources, coverage of oral rehydration therapy, and the Socio-demographic Index.^19^ The covariates used in our analysis are detailed in appendix 1 (p 14). These sub-models include mixed-effects regression models and spatiotemporal Gaussian process regression models with cause fractions and mortality rates as the outcome variables. We tested the out-of-sample predictive validity of the sub-models, which were then combined into an ensemble with the best out-of-sample predictive performance.

Input data for estimating diarrhoeal disease morbidity included incidence and prevalence data identified via systematic reviews, population-based surveys, claims data, and inpatient data (appendix 1 pp 15–18). Before modelling, we enhanced the comparability of the data from different sources (appendix 1 pp 16–17). We used DisMod-MR (version 2.1),^1^, ^20^ a Bayesian meta-regression tool that imposes coherence between data for different parameters, to produce incidence and prevalence estimates.

For aetiology (pathogen) estimation, we used a counterfactual approach consistent with previous GBD cycles^1^, ^2^ that incorporated the pathogen-specific risk of diarrhoeal disease and the prevalence of the pathogen in diarrhoea episodes (appendix 1 pp 21–29). We estimated population attributable fractions (PAFs) for the following pathogens: adenovirus, *Aeromonas* spp, *Campylobacter* spp, *Clostridium difficile*, *Cryptosporidium* spp, *Entamoeba histolytica*, norovirus, rotavirus, non-typhoidal *Salmonella* spp, *Shigella* spp, ST-ETEC, tEPEC, and *Vibrio cholerae*. We calculated disability-adjusted life-years (DALYs) for diarrhoeal diseases—a composite measure of burden that captures both premature mortality and the prevalence and severity of diarrhoea—as the sum of years of life lost (YLLs) due to premature mortality and years lived with disability (YLDs) from GBD 2021.

### Risk factors

Detailed methods for GBD risk factor estimation have been published elsewhere.^16^ In summary, we first selected risk–outcome pairs (eg, diarrhoeal disease attributable to suboptimal breastfeeding) based on evidence of a convincing or probable causal relationship between the risk and the outcome. The list of diarrhoeal disease risk factors and the mechanism through which each risk factor could result in diarrhoeal diseases is summarised in appendix 1 (p 74). The PAFs of risk factors were quantified by estimating the risk factor exposure distributions and the relative risk of the association between each risk factor and outcome and determining the theoretical minimum risk exposure level (TMREL). More details of these methods are provided in appendix 1 (pp 31–71). The PAF is the fraction of diarrhoeal disease DALYs that would have been reduced if the exposure to the risk factor had been at the TMREL. The attributable burden was computed by multiplying the location–year–age–sex-specific PAFs of risk factors by corresponding diarrhoeal disease DALYs. We also calculated risk-deleted diarrhoeal disease DALYs to represent the DALYs that would have been observed had the risk factors been set to their corresponding TMRELs.

### Uncertainty intervals, age standardisation, percentage changes, and result presentation

We computed 95% uncertainty intervals (UIs) based on 1000 draws from the posterior distribution of each quantity of interest using the 2·5th and 97·5th percentiles of the 1000 ordered values. We used the GBD world population age standard^21^ to calculate age-standardised diarrhoeal disease mortality and DALY rates. The percentage change was calculated by subtracting the initial value (eg, for the year 1990) from the final value (eg, for the year 2021), then dividing the result by the initial value and multiplying by 100. Count estimates are presented to three significant figures, and percentages and rates are presented to 1 decimal place. We present results in aggregated age groups (all ages, <5 years, 5–14 years, 15–49 years, 50–69 years, and ≥70 years) and more granular age groups for those aged younger than 5 years (early neonatal, late neonatal, 1–5 months, 6–11 months, 12–23 months, 2–4 years) for the years 1990 to 2021. Additional age–sex-specific results for diarrhoea and aetiologies can be found in the GBD Results Tool.

### Role of the funding source

The funder of the study had no role in study design, data collection, data analysis, data interpretation, writing of the report, or the decision to submit the manuscript for publication.

## Results

### Global trends in diarrhoeal disease mortality

**Figure 1:**
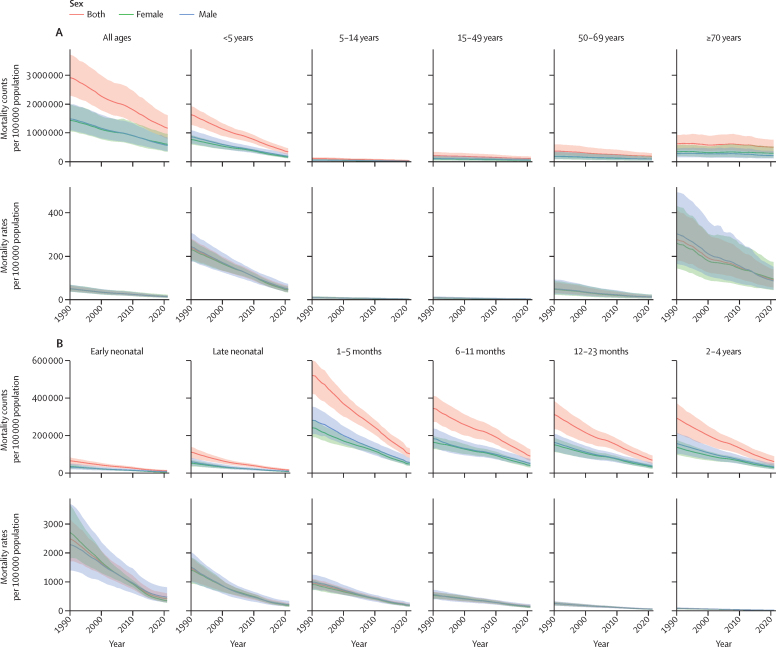
Diarrhoeal mortality rates per 100 000 and counts by broad age categories (A) and under-5 age groups (B), from 1990 to 2021 Shaded areas represent 95% uncertainty intervals. Early neonatal represents newborns aged 0–6 days. Late neonatal represents newborns aged 7–27 days.

Globally in 2021, diarrhoeal diseases accounted for 1·17 million (95% UI 0·793–1·62) deaths, a 60·3% (50·6–69·0) decline from the 2·93 million (2·31–3·73) estimated deaths in 1990. During this period, the reduction in diarrhoeal mortality rates per 100 000 population across age–sex groups varied from a 79·2% (72·4–84·6) decrease in children younger than 5 years (both males and females) to a 63·4% (46·4–73·9) decrease in women aged 70 years and older (table, figure 1).

**Table:** Diarrhoeal deaths and mortality rates in 2021 and percentage change in deaths and mortality rates between 1990 and 2021 by age, sex, and GBD super-region

	**Male**				**Female**			
	Number of deaths in 2021	Mortality rate (per 100 000 population) in 2021	Percentage change in number of deaths, 1990–2021	Percentage change in mortality rate (per 100 000 population), 1990–2021	Number of deaths in 2021	Mortality rate (per 100 000 population) in 2021	Percentage change in number of deaths, 1990–2021	Percentage change in mortality rate (per 100 000 population), 1990–2021
**Global**								
All ages	561 000 (365 000 to 841 000)	14·2 (9·2 to 21·2)	−62·4% (−71·0 to −51·6)	−74·5% (−80·3 to −67·2)	605 000 (346 000 to 966 000)	15·4 (8·8 to 24·6)	−58·0% (−71·2 to −43·8)	−71·7% (−80·6 to −62·2)
<5 years	185 000 (128 000 to 271 000)	54·5 (37·6 to 79·7)	−78·6% (−84·5 to −70·6)	−79·9% (−85·5 to −72·4)	155 000 (116 000 to 209 000)	48·7 (36·4 to 65·7)	−79·9% (−86·0 to −71·8)	−81·0% (−86·8 to −73·4)
5–14 years	18 000 (8 030 to 32 200)	2·6 (1·1 to 4·6)	−65·3% (−78·9 to −49·1)	−71·4% (−82·7 to −58·2)	15 800 (6 420 to 31 200)	2·4 (1·0 to 4·8)	−69·3% (−80·3 to −54·0)	−74·5% (−83·6 to −61·8)
15–49 years	58 700 (25 500 to 106 000)	2·9 (1·3 to 5·3)	−46·9% (−61·7 to −25·1)	−63·6% (−73·7 to −48·6)	47 800 (18 100 to 92 200)	2·5 (0·9 to 4·7)	−50·2% (−65·0 to −26·4)	−65·8% (−76·0 to −49·5)
50–69 years	92 400 (41 700 to 170 000)	13·1 (5·9 to 24·1)	−49·9% (−62·8 to −31·8)	−76·1% (−82·2 to −67·4)	94 200 (37 300 to 187 000)	12·9 (5·1 to 25·5)	−47·6% (−61·5 to −27·0)	−75·3% (−81·9 to −65·6)
≥70 years	206 000 (110 000 to 363 000)	94·8 (50·7 to 167·0)	−25·8% (−45·1 to −0·5)	−71·8% (−79·1 to −62·1)	292 000 (135 000 to 534 000)	105·4 (48·8 to 192·7)	−15·1% (−39·4 to 24·5)	−63·4% (−73·9 to −46·4)
**Central Europe, eastern Europe, and central Asia**								
All ages	2 270 (1890 to 2760)	1·1 (0·9 to 1·4)	−75·3% (−80·3 to −70·3)	−75·1% (−80·2 to −70·1)	2760 (2380 to 3210)	1·3 (1·1 to 1·5)	−66·2% (−71·4 to −60·0)	−65·9% (−71·1 to −59·7)
<5 years	1070 (701 to 1520)	8·1 (5·3 to 11·5)	−87·2% (−92·0 to −82·2)	−82·4% (−89·0 to −75·4)	919 (638 to 1310)	7·4 (5·1 to 10·6)	−87·6% (−91·5 to −82·4)	−82·5% (−87·9 to −75·2)
5–14 years	29 (17 to 47)	0·1 (0·1 to 0·2)	−74·0% (−82·6 to −60·8)	−67·4% (−78·2 to −50·8)	28 (17 to 45)	0·1 (0·1 to 0·2)	−72·5% (−81·1 to −59·7)	−64·6% (−75·6 to −48·1)
15–49 years	121 (96 to 156)	0·1 (0·1 to 0·2)	−45·0% (−53·5 to −33·1)	−43·0% (−51·9 to −30·7)	78 (65 to 98)	0·1 (0·1 to 0·1)	−46·3% (−53·3 to −37·0)	−43·8% (−51·1 to −34·1)
50–69 years	292 (263 to 325)	0·6 (0·6 to 0·7)	12·1% (3·4 to 22·2)	−9·0% (−16·1 to −0·9)	252 (230 to 275)	0·5 (0·4 to 0·5)	34·3% (24·5 to 44·3)	16·4% (7·8 to 25·1)
≥70 years	755 (684 to 838)	5·5 (5·0 to 6·1)	320·6% (270·4 to 365·0)	132·8% (105·0 to 157·3)	1480 (1260 to 1640)	5·7 (4·9 to 6·4)	409·1% (341·5 to 462·4)	248·2% (202·0 to 284·7)
**High income**								
All ages	12 100 (10 800 to 13 000)	2·3 (2·0 to 2·4)	321·1% (283·1 to 348·1)	249·1% (217·6 to 271·5)	17 700 (14 100 to 20 000)	3·2 (2·5 to 3·6)	337·9% (284·0 to 376·2)	266·2% (221·1 to 298·2)
<5 years	208 (180 to 239)	0·7 (0·6 to 0·9)	−74·5% (−78·5 to −70·4)	−71·0% (−75·6 to −66·3)	179 (154 to 205)	0·7 (0·6 to 0·8)	−71·2% (−75·2 to −66·6)	−67·4% (−71·9 to −62·2)
5–14 years	21 (19 to 24)	0·0 (0·0 to 0·0)	−34·5% (−50·2 to −18·9)	−32·5% (−48·6 to −16·4)	19 (17 to 21)	0·0 (0·0 to 0·0)	−22·0% (−42·9 to −1·5)	−19·6% (−41·2 to 1·5)
15–49 years	220 (204 to 238)	0·1 (0·1 to 0·1)	65·3% (43·8 to 90·4)	60·2% (39·4 to 84·5)	174 (163 to 184)	0·1 (0·1 to 0·1)	107·2% (79·4 to 137·3)	101·6% (74·5 to 130·9)
50–69 years	1490 (1410 to 1600)	1·1 (1·0 to 1·2)	284·4% (251·6 to 318·2)	135·7% (115·6 to 156·4)	1380 (1310 to 1460)	1·0 (0·9 to 1·0)	351·6% (309·8 to 391·8)	192·6% (165·5 to 218·7)
≥70 years	10 200 (8930 to 11 000)	15·5 (13·5 to 16·6)	573·6% (519·8 to 617·5)	191·1% (167·8 to 210·0)	16 000 (12 400 to 18 200)	18·3 (14·2 to 20·9)	430·5% (372·2 to 478·5)	188·4% (156·7 to 214·5)
**Latin America and Caribbean**								
All ages	12 600 (10 400 to 15 500)	4·3 (3·6 to 5·3)	−80·4% (−83·9 to −76·6)	−87·0% (−89·3 to −84·5)	13 000 (11 200 to 15 400)	4·3 (3·7 to 5·1)	−76·1% (−79·5 to −71·7)	−84·4% (−86·7 to −81·6)
<5 years	4610 (3290 to 6150)	19·1 (13·6 to 25·5)	−90·9% (−93·5 to −87·9)	−90·5% (−93·3 to −87·4)	3580 (2550 to 4700)	15·5 (11·0 to 20·3)	−91·3% (−93·9 to −88·6)	−90·9% (−93·5 to −88·0)
5–14 years	312 (225 to 434)	0·6 (0·5 to 0·9)	−81·4% (−85·7 to −76·1)	−81·8% (−86·0 to −76·7)	258 (194 to 355)	0·5 (0·4 to 0·8)	−83·6% (−86·7 to −79·6)	−83·6% (−86·7 to −79·6)
15–49 years	1160 (933 to 1490)	0·8 (0·6 to 1·0)	−60·7% (−66·5 to −52·4)	−75·3% (−78·9 to −70·1)	881 (719 to 1140)	0·6 (0·5 to 0·7)	−60·6% (−66·6 to −52·8)	−74·9% (−78·7 to −70·0)
50–69 years	2030 (1690 to 2500)	4·1 (3·4 to 5·1)	−41·7% (−48·8 to −32·6)	−78·2% (−80·8 to −74·7)	2020 (1740 to 2480)	3·7 (3·2 to 4·5)	−26·9% (−35·5 to −17·9)	−74·1% (−77·1 to −70·9)
≥70 years	4470 (3860 to 5240)	29·6 (25·5 to 34·7)	−23·8% (−30·4 to −16·6)	−74·7% (−76·9 to −72·3)	6240 (5230 to 7340)	32·2 (27·0 to 37·8)	−0·3% (−10·0 to 8·5)	−69·8% (−72·7 to −67·1)
**North Africa and Middle East**								
All ages	8840 (5880 to 13 700)	2·7 (1·8 to 4·2)	−83·1% (−88·3 to −77·7)	−90·9% (−93·7 to −88·0)	7160 (5030 to 9840)	2·4 (1·7 to 3·3)	−84·1% (−88·4 to −78·2)	−91·2% (−93·6 to −88·0)
<5 years	6400 (3970 to 11 100)	20·3 (12·6 to 35·2)	−86·8% (−91·2 to −81·4)	−89·0% (−92·7 to −84·5)	5070 (3580 to 7170)	17·1 (12·1 to 24·2)	−87·9% (−91·4 to −83·5)	−89·8% (−92·8 to −86·1)
5–14 years	305 (97 to 641)	0·5 (0·2 to 1·0)	−63·8% (−84·2 to −34·7)	−73·7% (−88·6 to −52·5)	243 (90 to 536)	0·4 (0·2 to 0·9)	−66·5% (−82·9 to −26·9)	−75·4% (−87·4 to −46·3)
15–49 years	387 (131 to 710)	0·2 (0·1 to 0·4)	−34·8% (−53·1 to −4·8)	−69·4% (−78·0 to −55·3)	303 (101 to 599)	0·2 (0·1 to 0·4)	−31·0% (−52·5 to 15·1)	−66·2% (−76·7 to −43·5)
50–69 years	454 (163 to 822)	1·0 (0·4 to 1·9)	−33·4% (−52·1 to 0·1)	−75·7% (−82·5 to −63·5)	372 (137 to 799)	0·9 (0·3 to 1·9)	−28·6% (−52·1 to 5·3)	−73·5% (−82·2 to −61·0)
≥70 years	1290 (497 to 2610)	13·0 (5·0 to 26·2)	−16·5% (−38·5 to 22·7)	−69·9% (−77·8 to −55·8)	1160 (442 to 2520)	11·2 (4·3 to 24·2)	−18·6% (−51·5 to 21·6)	−71·5% (−83·0 to −57·4)
**South Asia**								
All ages	249 000 (143 000 to 448 000)	26·4 (15·2 to 47·6)	−64·9% (−74·8 to −53·3)	−78·8% (−84·8 to −71·8)	323 000 (151 000 to 612 000)	35·7 (16·7 to 67·6)	−58·5% (−74·4 to −39·5)	−75·9% (−85·2 to −64·9)
<5 years	30 700 (15 200 to 50 000)	37·1 (18·4 to 60·5)	−89·4% (−95·0 to −83·7)	−89·6% (−95·1 to −83·9)	25 500 (16 400 to 37 200)	33·6 (21·6 to 49·0)	−92·1% (−94·7 to −88·5)	−92·1% (−94·7 to −88·5)
5–14 years	6000 (2480 to 11 300)	3·3 (1·4 to 6·2)	−81·2% (−88·0 to −73·6)	−85·1% (−90·5 to −79·1)	6980 (2650 to 13 000)	4·2 (1·6 to 7·8)	−79·3% (−86·8 to −69·4)	−83·5% (−89·5 to −75·7)
15–49 years	22 300 (8910 to 45 100)	4·4 (1·7 to 8·8)	−65·6% (−73·7 to −55·4)	−81·6% (−85·9 to −76·2)	23 400 (9000 to 46 900)	4·7 (1·8 to 9·5)	−60·3% (−71·8 to −46·4)	−79·5% (−85·5 to −72·4)
50–69 years	49 200 (21 400 to 95 500)	38·0 (16·5 to 73·7)	−60·1% (−69·7 to −48·9)	−82·4% (−86·6 to −77·5)	59 100 (22 600 to 124 000)	45·4 (17·4 to 95·4)	−51·8% (−66·1 to −36·4)	−81·3% (−86·9 to −75·4)
≥70 years	141 000 (73 100 to 264 000)	404·4 (210·1 to 757·9)	−29·3% (−47·7 to −2·0)	−75·6% (−81·9 to −66·2)	208 000 (92 400 to 403 000)	541·1 (240·4 to 1048·2)	−13·2% (−42·9 to 24·0)	−74·1% (−83·0 to −63·0)
**Southeast Asia, east Asia, and Oceania**								
All ages	40 900 (21 800 to 59 800)	3·7 (2·0 to 5·4)	−80·7% (−87·4 to −71·0)	−85·0% (−90·3 to −77·5)	42 500 (18 000 to 64 800)	4·0 (1·7 to 6·0)	−78·7% (−89·3 to −65·7)	−83·6% (−91·7 to −73·6)
<5 years	9060 (6100 to 12 900)	12·4 (8·4 to 17·6)	−93·2% (−95·6 to −89·0)	−91·5% (−94·4 to −86·1)	7200 (5610 to 9140)	11·0 (8·6 to 14·0)	−93·5% (−95·7 to −90·1)	−91·7% (−94·5 to −87·4)
5–14 years	918 (475 to 1750)	0·6 (0·3 to 1·1)	−85·9% (−92·0 to −73·8)	−85·2% (−91·6 to −72·5)	634 (263 to 1360)	0·4 (0·2 to 0·9)	−89·1% (−93·1 to −79·7)	−88·1% (−92·4 to −77·8)
15–49 years	4270 (1800 to 7940)	0·8 (0·3 to 1·4)	−71·6% (−83·2 to −51·1)	−75·5% (−85·5 to −57·8)	2650 (1010 to 5760)	0·5 (0·2 to 1·1)	−77·7% (−86·6 to −56·0)	−80·4% (−88·2 to −61·3)
50–69 years	9090 (3590 to 15 500)	3·5 (1·4 to 6·0)	−58·2% (−76·0 to −26·5)	−82·9% (−90·2 to −69·9)	8050 (2600 to 14 200)	3·1 (1·0 to 5·4)	−64·2% (−81·8 to −16·4)	−86·1% (−92·9 to −67·4)
≥70 years	17 500 (6660 to 26 300)	25·4 (9·6 to 38·1)	−49·2% (−71·6 to −7·7)	−84·1% (−91·1 to −71·0)	24 000 (7440 to 38 100)	28·3 (8·8 to 45·0)	−50·7% (−76·5 to 12·0)	−83·6% (−92·2 to −62·7)
**Sub-Saharan Africa**								
All ages	235 000 (156 000 to 333 000)	42·1 (28·0 to 59·7)	−46·8% (−62·8 to −24·8)	−76·8% (−83·8 to −67·2)	199 000 (129 000 to 278 000)	34·5 (22·3 to 48·3)	−43·5% (−60·9 to −21·1)	−75·6% (−83·1 to −66·0)
<5 years	133 000 (87 100 to 199 000)	151·9 (99·2 to 226·6)	−60·1% (−73·2 to −41·8)	−79·4% (−86·1 to −70·0)	113 000 (76 700 to 163 000)	132·4 (90·2 to 191·2)	−53·9% (−70·6 to −29·4)	−76·0% (−84·7 to −63·1)
5–14 years	10 400 (4510 to 18 800)	6·8 (3·0 to 12·3)	−3·3% (−54·2 to 68·6)	−57·3% (−79·8 to −25·6)	7620 (3070 to 15 600)	5·1 (2·0 to 10·4)	−19·6% (−53·6 to 66·6)	−64·0% (−79·2 to −25·4)
15–49 years	30 200 (13 300 to 54 800)	11·4 (5·0 to 20·6)	12·9% (−37·7 to 78·0)	−54·6% (−75·0 to −28·5)	20 300 (7520 to 39 600)	7·2 (2·7 to 14·1)	−8·6% (−46·1 to 81·5)	−63·5% (−78·5 to −27·5)
50–69 years	29 800 (12 900 to 51 900)	69·4 (30·0 to 120·7)	−14·2% (−55·2 to 37·2)	−61·0% (−79·6 to −37·6)	23 100 (8360 to 43 800)	48·5 (17·6 to 92·0)	−25·8% (−55·9 to 42·2)	−70·4% (−82·4 to −43·3)
≥70 years	31 200 (14 000 to 51 900)	358·1 (160·2 to 595·1)	−11·8% (−51·3 to 30·5)	−56·4% (−75·9 to −35·4)	35 100 (13 200 to 64 500)	323·2 (121·3 to 593·2)	−20·8% (−53·1 to 41·5)	−64·1% (−78·8 to −35·9)

Data in parentheses are 95% uncertainty intervals. Each section represents estimates at the global or super-region level. All ages is an aggregate of all child and adult age groups. The <5 years category is an aggregate of all granular age groups aged <5 years. Count estimates are presented to three significant figures, and percentages and rates are presented to 1 decimal place. GBD=Global Burden of Diseases, Injuries, and Risk Factors Study.

**Figure 2:**
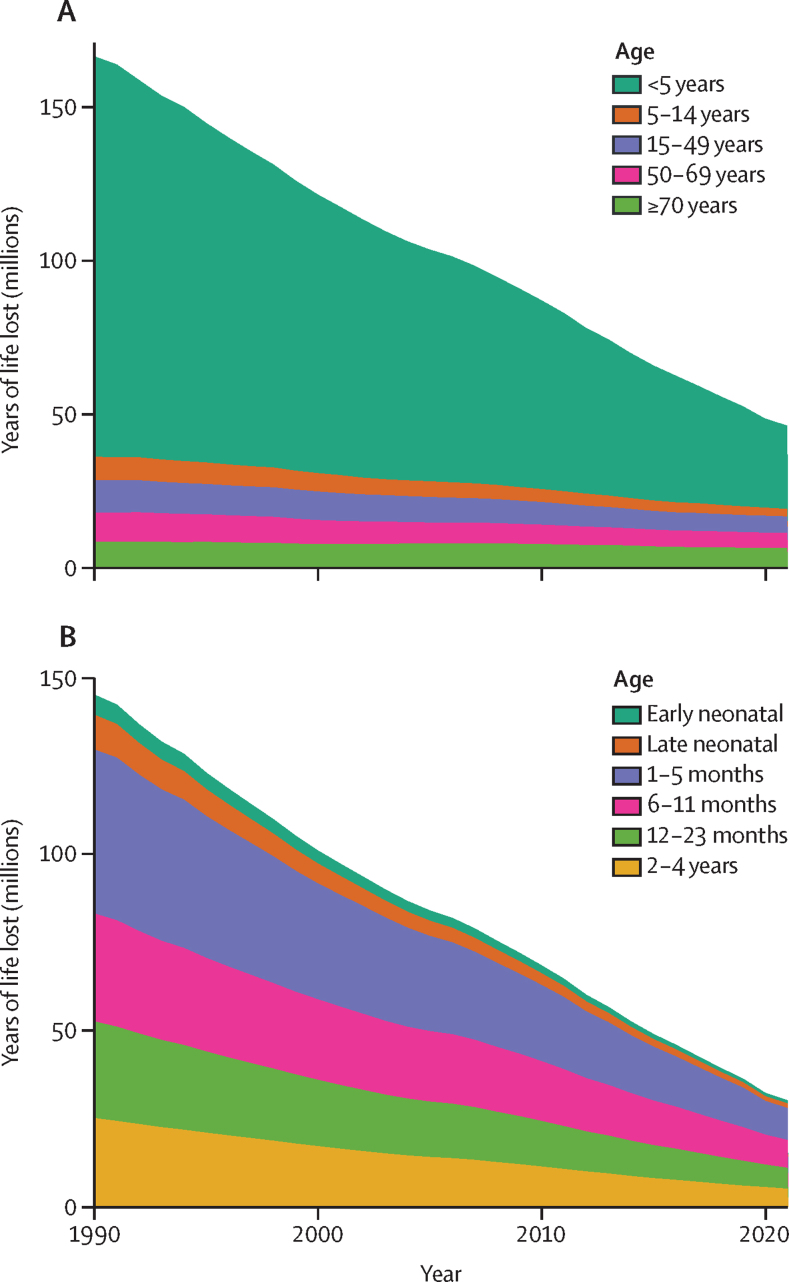
Years of life lost due to diarrhoeal diseases by broad age categories (A) and under-5 age groups (B), from 1990 to 2021 Years of life lost are shown in millions with each colour representing one age group. Early neonatal represents newborns aged 0–6 days. Late neonatal represents newborns aged 7–27 days.

The global estimated total YLLs due to diarrhoeal diseases decreased substantially, from 186 million (95% UI 147–221) in 1990 to 51·4 million (39·9–65·9) in 2021 (figure 2). Children younger than 5 years showed the most significant drop in YLLs during this period, from 146 million (114–172) in 1990 to 30·3 million (22·3–41·3) in 2021. Declines were also seen in older age groups between 1990 and 2021: YLLs decreased from 8·39 million (4·94–12·0) to 2·73 million (1·58–4·49) in those aged 5–14 years, from 11·8 million (7·22–19·0) to 6·04 million (3·41–9·84) in those aged 15–49 years, from 10·8 million (6·76–17·5) to 5·48 million (3·10–8·59) in those aged 50–69 years, and from 9·15 million (5·86–13·8) to 6·92 million (4·11–10·6) in those aged 70 years and older.

### Annualised rates of change in diarrhoeal disease mortality in 1990–2019 and 2019–2021

Before the COVID-19 pandemic, from 1990 to 2019, the global all-age diarrhoeal disease mortality rate changed at a rate of –4·2% (95% UI –5·0 to –3·5) per year, with substantial variation across regions and countries, indicating an increase, decrease, or no change in diarrhoeal disease mortality rates (appendix 2 table S1). From 2019 to 2021, the global all-age diarrhoeal disease mortality rate changed at a rate of –5·0% (–7·3 to –2·7) per year, showing similar variation across regions and countries (appendix 2 table S1).

### Variation in diarrhoeal disease mortality in 2021

In 2021, when comparing diarrhoeal disease mortality rates across different age–sex groups in children younger than 5 years, the highest global mortality rates were estimated to be in the early neonatal age group (0–6 days old), with 471·0 (95% UI 286·2–812·0) deaths per 100 000 population in males and 348·7 (274·6–471·4) deaths per 100 000 population in females. As age increased, mortality rates declined, reaching 15·1 (9·2–23·9) deaths per 100 000 population in males and 14·2 (9·2–21·5) deaths per 100 000 population in females aged 2–4 years (appendix 2 table S2). Among other age groups, the highest global mortality rates due to diarrhoeal diseases in 2021 were estimated to be in those aged 70 years and older, with 94·8 (50·7–167·0) deaths per 100 000 population in males and 105·4 (48·8–192·7) deaths per 100 000 population in females (table). Those aged 5–14 years had the lowest mortality rates: 2·6 (1·1–4·6) deaths per 100 000 population in males and 2·4 (1·0–4·8) deaths per 100 000 population in females.

**Figure 3:**
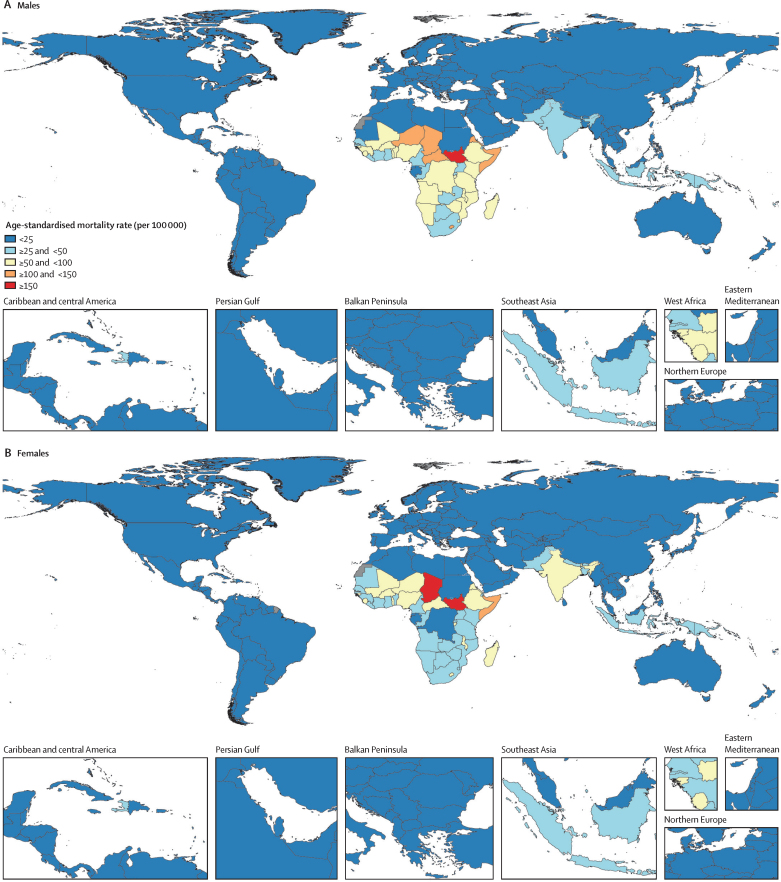
Age-standardised diarrhoeal mortality rates per 100 000 population in males (A) and females (B) in 2021 Grey shading indicates the location has no data.

Regionally, in 2021, sub-Saharan Africa had the highest mortality rates for children younger than 5 years (151·9 [95% UI 99·2–226·6] deaths per 100 000 population in males and 132·4 [90·2–191·2] deaths per 100 000 population in females), while south Asia had the highest rates in those aged 70 years and older (404·4 [210·1–757·9] deaths per 100 000 population in males and 541·1 [240·4–1048·2] deaths per 100 000 population in females; table). Although diarrhoeal mortality rates declined substantially across age groups in most super-regions, in the high-income super-region, as well as in central Europe, eastern Europe, and central Asia, the mortality rates from diarrhoea in adults aged 50–69 years and those 70 years and older either did not change or increased between 1990 and 2021 (table). At the country level, age-standardised mortality rates per 100 000 population were greater than 100 in seven countries (South Sudan, Central African Republic, Chad, Somalia, Lesotho, Niger, and Eritrea) for males and in three countries (South Sudan, Chad, and Somalia) for females in 2021 (figure 3).

### Diarrhoeal disease burden attributable to risk factors

In 2021, we estimated a global total of 59·0 million (95% UI 47·2–73·2) DALYs due to diarrhoeal diseases; 30·9 million (23·1–42·0) of these DALYs were in children younger than 5 years (appendix 2 table S3). Of the total DALYs, 54·0 million (42·2–67·1) were attributed to all evaluated diarrhoeal risk factors. Of the DALYs estimated for children younger than 5 years, 30·4 million (22·8–41·0) were attributed to all evaluated diarrhoeal risk factors.

**Figure 4:**
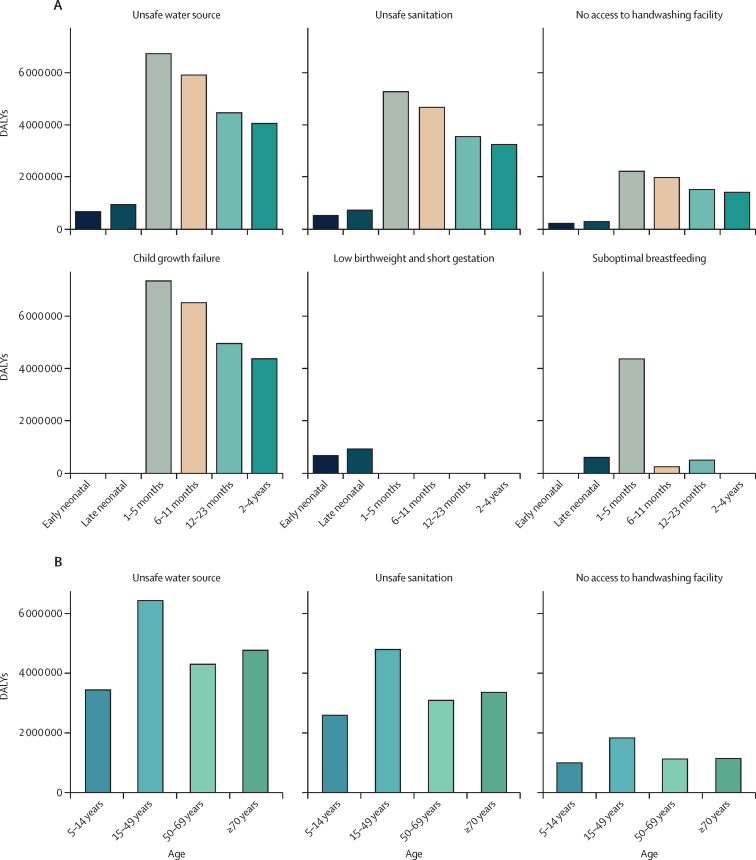
Diarrhoeal DALYs attributable to the leading risk factors in children younger than 5 years (A) and other age groups (B) in 2021 Charts represent DALYs in counts for children younger than 5 years (A) and those aged 5 years and older (B). Early neonatal represents newborns aged 0–6 days. Late neonatal represents newborns aged 7–27 days. Low birthweight is defined as any birthweight in 500 g units below the TMREL at 38 weeks or later but less than 40 weeks, and 3500 g or greater but less than 4000 g. Short gestation refers to any gestational age that falls below the gestational age TMREL at less than 37 completed weeks. Suboptimal breastfeeding includes the absence of breastmilk as a source of nourishment for children aged 6–23 months and the practice of non-exclusive breastfeeding among infants younger than 6 months. Child growth failure includes stunting, wasting, and underweight. DALY=disability-adjusted life-year. TMREL=theoretical minimum risk exposure level.

A breakdown by age among children younger than 5 years reveals that in the early neonatal group (newborns aged 0–6 days), low birthweight and short gestation was the predominant risk factor, contributing to 683 000 (95% UI 493 000–1 030 000) DALYs (figure 4). This was closely followed by unsafe water, at 666 000 (365 000–1 060 000) DALYs. Unsafe sanitation practices resulted in about 523 000 (365 000–773 000) DALYs, while no access to handwashing facilities added another 217 000 (32 700–408 000) DALYs. The leading risk factors for the late neonatal group (newborns aged 7–27 days) remained similar to those in the early neonatal group, with the addition of suboptimal breastfeeding, which contributed 607 000 (428 000–845 000) DALYs.

For infants aged 1–5 months, the largest risk factor for diarrhoeal diseases was child growth failure, accounting for 7·34 million (95% UI 3·90–9·96) DALYs (figure 4A). Unsafe water was the second leading risk factor, contributing 6·73 million (3·66–9·41) DALYs. Poor sanitation practices followed as the third leading risk factor, contributing 5·27 million (3·94–6·85) DALYs, and suboptimal breastfeeding was the fourth, contributing 4·37 million (3·19–5·84) DALYs. In infants aged 6–11 months, child growth failure remained the most significant risk factor, with 6·51 million (3·66–9·32) DALYs attributed to diarrhoeal diseases. Unsafe water and unsafe sanitation practices continued to be major risk factors, accounting for 5·91 million (3·33–8·90) and 4·67 million (3·23–6·74) DALYs, respectively. This pattern of risk factors leading to the highest numbers of DALYs persisted in those aged 12–23 months and 2–4 years (figure 4A).

For children aged 5–14 years, unsafe water was the leading risk factor for diarrhoeal diseases, contributing 3·44 million (95% UI 1·62–5·24) DALYs. Poor sanitation followed, contributing 2·59 million (1·78–3·75) DALYs. The absence of handwashing facilities contributed 0·993 million (0·141–1·96) DALYs. Unsafe water continued to pose a major health risk in adults, with total DALYs attributed to diarrhoeal diseases of 6·45 million (3·00–10·2) in those aged 15–49 years, 4·31 million (1·72–7·00) in those aged 50–69 years, and 4·77 million (1·98–8·23) in those aged 70 years and older (figure 4B). Unsafe sanitation and lack of access to handwashing facilities were the second and third leading risk factors in these age groups. The corresponding PAFs of individual risk factors for diarrhoeal diseases are presented in appendix 2 for granular age groups aged younger and older than 5 years (appendix 2 table S4 and table S5).

When considering a scenario with all diarrhoeal risk factors removed, the global all-age DALYs would decrease from 59·0 million (95% UI 47·2–73·2) to 4·99 million (1·99–10·0), and among children younger than 5 years they would decrease from 30·9 million (23·1–42·0) to 556 000 (67 100–1 400 000; appendix 2 table S2). At the super-region level, the comparison of DALYs before and after the removal of risk factors in the top five regions with the highest diarrhoeal burden in 2021 showed a substantial decrease: from 30·9 million (23·0–40·9) to 894 000 (206 000–2 210 000) DALYs in sub-Saharan Africa; from 20·4 million (15·1–29·1) to 2·34 million (0·696–5·14) DALYs in south Asia; from 3·83 million (2·91–4·79) to 591 000 (194 000–1 380 000) DALYs in southeast Asia, east Asia, and Oceania; from 1·49 million (1·13–1·99) to 210 000 (91 600–357 000) DALYs in north Africa and the Middle East; and from 1·35 million (1·11–1·63) to 260 000 (117 000–451 000) DALYs in Latin America and the Caribbean. India, Nigeria, and Pakistan would experience the most gains due to their population sizes, with a decrease in DALYs from 16·8 million (12·0–24·2) to 2·11 million (0·644–4·56) in India, from 10·1 million (6·93–13·9) to 164 000 (0–452 000) in Nigeria, and from 2·47 million (1·83–3·39) to 152 000 (35 600–363 000) in Pakistan.

### Diarrhoeal disease burden attributable to aetiologies

Globally, in 2021, among all ages, rotavirus was the leading cause of diarrhoeal deaths, with a PAF of 15·2% (95% UI 11·4–20·1), followed by norovirus at 10·6% (2·3–17·0) and *Cryptosporidium* spp at 10·2% (7·03–14·3; appendix 2 table S8). Rotavirus accounted for an estimated 176 000 (131 000–230 000) diarrhoeal deaths and 13·4 million (9·85–17·9) DALYs in 2021. In the same year, norovirus was responsible for an estimated 124 000 (25 800–224 000) diarrhoeal deaths and 5·69 million (1·88–9·67) DALYs, whereas *Cryptosporidium* spp caused 118 000 (75 300–178 000) diarrhoeal deaths and 7·37 million (4·53–11·30) DALYs.

Among children younger than 5 years, rotavirus topped the list with a fatal PAF of 35·2% (95% UI 28·7–43·0), followed by *Shigella* spp at 24·0% (15·2–37·9) and adenovirus at 23·8% (14·8–36·3) (appendix 2 table S7). Other pathogens with a fatal PAF greater than 5% included *Cryptosporidium* spp (20·1% [13·3–30·9]), tEPEC (13·6% [8·1–21·0]), ST-ETEC (13·4% [8·3–21·1]), norovirus (8·7% [3·2–14·9]), *Vibrio cholerae* (7·7% [4·8–11·3]), and *Campylobacter* spp (7·4% [3·4–13·3]). The leading pathogen, rotavirus, contributed to an estimated 120 000 (83 100–169 000) diarrhoeal deaths and 10·8 million (7·52–15·2) DALYs in 2021. *Shigella* spp and adenovirus also posed a substantial burden, with *Shigella* spp contributing to 81 800 (47 900–138 000) diarrhoeal deaths and 7·34 million (4·32–12·4) DALYs, and adenovirus contributing to 81 100 (44 900–133 000) diarrhoeal deaths and 7·32 million (4·05–12·0) DALYs.

**Figure 5:**
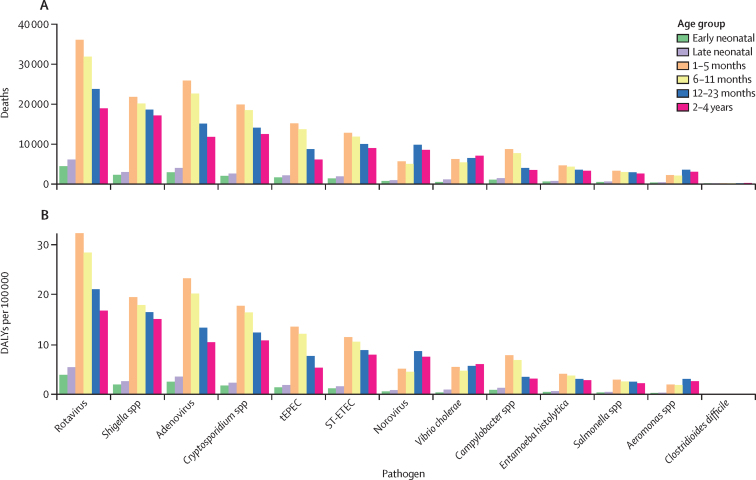
Number of diarrhoeal deaths (A) and DALYs (B) in specific age groups in children younger than 5 years attributable to 13 pathogens in 2021 Early neonatal represents newborns aged 0–6 days. Late neonatal represents newborns aged 7–27 days. DALY=disability-adjusted life-year. PAF=population attributable fraction. ST-ETEC=enterotoxigenic *Escherichia coli* producing heat-stable toxin. tEPEC=typical enteropathogenic *E coli*.

In children younger than 5 years, the 120 000 diarrhoeal deaths due to rotavirus were distributed across age groups as follows: 4270 (95% UI 2810–6540) deaths in the early neonatal group, 5940 (4130–8630) in the late neonatal group, 35 800 (25 300–48 800) in infants aged 1–5 months, 31 600 (20 100–46 900) in those aged 6–11 months, 23 500 (14 800–35 100) in those aged 12–23 months, and 18 700 (11 100–29 700) in children aged 2–4 years. The 10·8 million diarrhoeal DALYs in children younger than 5 years were distributed as follows: 0·384 million (0·253–0·589) DALYs in the early neonatal group, 0·538 million (0·374–0·781) in the late neonatal group, 3·23 million (2·28–4·40) in infants aged 1–5 months, 2·84 million (1·81–4·21) in those aged 6–11 months, 2·11 million (1·33–3·13) in those aged 12–23 months, and 1·68 million (1·01–2·63) in children aged 2–4 years. The age distributions of deaths and DALYs for rotavirus and other pathogens are illustrated in figure 5.

Although not a leading pathogen globally, *C difficile* was the main cause of diarrhoea-related deaths in high-income countries in 2021. We estimated 15 600 (95% UI 13 400–18 200) deaths and 284 000 (250 000–326 000) DALYs globally due to this pathogen (appendix 2 table S8). Specifically, in high-income regions, *C difficile* was associated with 13 100 (11 400–15 200) deaths and 218 000 (197 000–243 000) DALYs (appendix 2 table S6), predominantly affecting individuals aged 70 years and older (12 100 [10 100–14 500] deaths and 156 000 [132 000–186 000] DALYs).

More detailed diarrhoeal disease burden results by age and sex (including results for more granular age groups in adults) across locations and years are available in GBD Compare.

## Discussion

The global burden of diarrhoeal diseases has substantially decreased between 1990 and 2021, with the number of deaths reduced by 60·3% during this period. The largest decline in diarrhoeal mortality rates was observed in children younger than 5 years, with a 79·2% decrease in deaths. Despite these global declines, there were still 51·4 million YLLs in 2021, including 30·3 million in children younger than 5 years. Neonates had the highest diarrhoeal disease mortality rates despite some inherent protection against diarrhoeal diseases from maternal antibodies and breastfeeding, which is likely to be due to factors such as immune system immaturity, poor access to clean water and sanitation, suboptimal breastfeeding practices, and restricted access to health care. Substantial variation remains across regions and countries in both the levels of, and trends in, mortality due to diarrhoeal diseases. In 2021, diarrhoeal diseases globally contributed to 59·0 million DALYs, which could have been reduced to 4·99 million DALYs had all evaluated risk factors been removed. Compared with previous GBD studies of the diarrhoeal disease burden,^2^, ^14^ rotavirus and *Shigella* spp continued to be the leading pathogens causing diarrhoeal deaths in children younger than 5 years globally in 2021, while *C difficile* was the primary cause of diarrhoeal deaths in high-income countries, especially in people aged 70 years and older.

The remarkable decline in diarrhoeal mortality since 1990, especially in children younger than 5 years, represents a triumph for public health initiatives worldwide. This success can be attributed to a multipronged approach that includes widespread immunisation against rotavirus,^22^, ^23^ the implementation of improved water, sanitation, and hygiene (WASH) practices, and broader access to oral rehydration therapies and health-care services.^24^, ^25^ The rotavirus vaccines recommended by WHO and administered in more than 100 countries have contributed to marked reductions in both hospital admissions and deaths caused by diarrhoea.^23^ Despite the oral vaccines' positive impact, challenges such as incomplete vaccine coverage^26^ and the need for parenteral vaccines^27^ persist. These live oral vaccines have shown reduced effectiveness in low-income countries compared with high-income countries, highlighting the need for additional research to identify factors that influence vaccine effectiveness in different settings.^28^ Ongoing efforts aim to create new vaccines that do not rely on the oral route, which could potentially play a key role in achieving sustained control of rotavirus disease.^23^

When it comes to vaccination against other diarrhoeal pathogens, oral killed cholera vaccines have been shown to be effective in protection against cholera in endemic areas.^29^ A highly effective cholera vaccine with more than 85% protective efficacy has yet to be introduced in endemic countries due to reduced efficacy and logistical storage challenges in field delivery.^29^, ^30^ Vaccine research targeting diarrhoeal pathogens, such as *Shigella* spp, ETEC, norovirus, and *Campylobacter* spp, is ongoing and continues to address a complex array of challenges, including the genetic and antigenic heterogeneity of the pathogens.^31^, ^32^, ^33^ With the growing number of vaccines being added to WHO's Expanded Programme on Immunization, the development of combination vaccines is appealing, as such vaccines could not only reduce manufacturing costs but also streamline the immunisation schedule.^34^ For instance, the development of a combined vaccine for *Shigella* spp, ETEC, and *Campylobacter* spp is seen as a potentially important advancement for reducing the diarrhoeal disease burden.^35^

As bacterial antimicrobial resistance has emerged as a major public health threat, preventing infections through vaccination is crucial to minimising the need for antibiotics.^36^ Improper use of antibiotics for treating conditions such as ETEC-induced diarrhoea can lead to increased antimicrobial resistance; an efficacious ETEC vaccine could decrease the number of infections requiring antibiotics and reduce the risk of developing antimicrobial-resistant strains.^33^, ^37^ Additionally, the emergence of *Shigella* strains resistant to most antimicrobials is a growing global concern.^38^, ^39^, ^40^ The mass distribution of azithromycin to preschool children has been shown to reduce childhood mortality in sub-Saharan Africa, likely through reductions in respiratory infections, diarrhoea, and malaria, yet any policy advocating for mass azithromycin distribution should carefully consider the potential risk of antibiotic resistance.^41^

Although the overall decrease in diarrhoeal disease mortality is encouraging, the rise in diarrhoeal deaths attributable to *C difficile* infection among adults in certain regions, such as high-income North America and Europe, presents a new set of challenges. This trend might reflect the increasing use of antibiotics and subsequent disruption to the gut microbiome, leading to heightened susceptibility to *C difficile* infection.^42^ Addressing this issue requires a multifaceted response, including improved antibiotic stewardship, heightened infection control measures in health-care settings, and continued research into effective treatments and preventive measures for *C difficile* infection and recurrence.^43^ Fidaxomicin is the primary treatment for *C difficile* infection,^44^ while fecal microbiota transplantation has been shown to be the most cost-effective treatment option for recurrent *C difficile* infection.^45^ A recent novel oral formulation of live fecal microbiota spores approved by the US Food and Drug Administration is a major advancement in gastroenterology, although the availability of this treatment outside the USA and Canada is still uncertain, highlighting the need for international collaboration to ensure its economic viability and equitable distribution.^46^ Although an efficacious vaccine for *C difficile* infection could help mitigate the disease, clinical trials of vaccines containing toxin-based antigens from *C difficile* have shown only modest efficacy, indicating the need for future vaccines to include bacterial or spore antigens to provide enhanced protection.^47^

Regional disparities in diarrhoeal disease mortality rates are stark, with less than one death per 100 000 population in children younger than 5 years in the high-income super-region versus more than 130 deaths per 100 000 population in children younger than 5 years in sub-Saharan Africa. Despite the remarkable progress made in recent years—more than 90% of the world's population has access to improved water sources, and 2·1 billion people have gained access to improved sanitation—challenges remain, particularly in scaling up WASH infrastructure in resource-limited settings.^48^ The new ambitious safely managed services framework by UNICEF, which considers factors such as on-premises availability of drinking water and its freedom from fecal and chemical contaminants, further highlights disparities in access to clean water.^49^ In Niger, for example, while 66% of the population has access to an improved water source, only 10% have the convenience of having it available on their premises.^48^ The refinement of WASH service definitions to include factors such as the absence of contaminants can highlight previously invisible issues, such as *E coli* contamination in piped water in some countries.^50^

In addition to improving WASH infrastructure, reducing the diarrhoeal disease burden in the under-5 age groups requires interventions that address malnutrition, such as promoting exclusive breastfeeding, addressing food insecurity, and fortifying foods with essential nutrients. The link between malnutrition and increased vulnerability to diarrhoea is compounded by climate change, with extreme weather conditions such as heavy rainfall and high temperatures amplifying the risk of diarrhoeal diseases.^51^ According to a recent review, climate change can act as a triggering factor for the occurrence of diarrhoea, although the underlying causes are more complex, encompassing factors such as rainfall, human behaviour, water availability, immunity, and socioeconomic influences.^52^ It is also noteworthy that diarrhoea and malnutrition share a bidirectional relationship, with malnutrition predisposing individuals to diarrhoeal infections through impaired immune defences, and diarrhoea exacerbating malnutrition by impairing nutrient absorption.^53^ Investing in the training of health professionals could empower them to lead interdisciplinary efforts, utilising the One Health framework, to address both the immediate and long-term challenges posed by climate change and diarrhoeal risk factors.^54^

The current study addressed multiple limitations identified in previous GBD diarrhoeal disease publications. Notably, it distinguished the specific burdens of ST-ETEC and tEPEC. These two pathogens were aggregated under the broader categories of all ETEC and all EPEC in previous publications.^2^, ^14^ Additionally, our study incorporated pathogen-specific data from WHO's Global Pediatric Diarrhea Surveillance network for many high-burden countries; these data were unavailable for incorporation into earlier GBD publications. Consequently, this has led to a reshuffling in the ranking of some pathogens compared to previous findings.^2^ While the leading three pathogens remain the same in children younger than 5 years, there has been a notable shift with tEPEC (formerly aggregated with all EPEC and ranked tenth) and ST-ETEC (formerly aggregated with all ETEC and ranked eighth), which now rank as the fifth and sixth most prevalent pathogens, respectively.

Despite these improvements, some data limitations persist, particularly the paucity of data to inform the estimation of overall diarrhoea mortality, especially for sub-Saharan Africa, and the scarcity of age-specific aetiological data for individuals older than 5 years. To address the limited availability of cause of death data, we incorporated covariates linked biologically or strongly associated with diarrhoeal diseases, sourced from population-based surveys such as Demographic and Health Surveys and Multiple Indicator Cluster Surveys. Additionally, we used spatial modelling to leverage data from neighbouring countries, which, while compensating for scarce information, expanded the uncertainty intervals in years with scarce data. We used verbal autopsy data to inform our estimates where reliable vital registration data were unavailable. Although verbal autopsy data might be prone to misclassification of causes of death, validation studies of verbal autopsies in children generally indicate reasonable sensitivity and specificity for diagnosing diarrhoeal diseases.^55^, ^56^ The propagation of uncertainty from multiple sources, including sampling variance, non-sampling variance, and adjustment and standardisation methods applied to data has resulted in wide uncertainty intervals, which might have affected the accuracy of our estimates.

Currently, we assume the association between pathogen detection and odds of diarrhoeal diseases in children younger than 5 years from GEMS is applicable to older age groups. Efforts are ongoing to address this limitation by integrating more odds ratio data across different geographical locations and age groups in future GBD studies. Moreover, the availability and quality of *C difficile* data in low-income and middle-income countries are inadequate. Due to substantial variation in diagnostic and surveillance practices in these countries, there is a potential for underestimating the *C difficile* burden, which might explain why increases in deaths due to *C difficile* were seen only in high-income countries.^57^ The availability of more robust data in these countries, through enhanced diagnostic and surveillance infrastructure, could help to provide more accurate estimates.

Furthermore, although DALYs offer a composite measure of disease burden, combining premature mortality with the prevalence and severity of diarrhoeal diseases, in this study they account only for the acute effects of diarrhoea. The broader impact of diarrhoeal morbidity, which can lead to long-term consequences such as stunted physical growth and cognitive impairment, has not yet been accounted for in the DALY estimates. Studies that have attempted to quantify some of the long-term consequences suggest that diarrhoea might represent a larger burden of disease than is currently estimated by GBD.^58^, ^59^ Addressing this gap in future iterations of GBD is crucial for a more comprehensive assessment of the true burden of diarrhoeal diseases.

Last, we did not quantify the indirect impact of the COVID-19 pandemic on the diarrhoeal disease burden for GBD 2021. Data from Demographic and Health Surveys show conflicting trends and do not provide a definitive indication of the impact of the COVID-19 pandemic on diarrhoeal prevalence (appendix 2 figure S1). Although some countries have reported a slight increase in diarrhoea prevalence from the pre-pandemic to the post-pandemic period, others have observed a decrease. It is noteworthy, however, that in countries where a decline has been reported, there was already an observable trend of declining diarrhoeal prevalence before the onset of the pandemic. Consequently, it remains uncertain whether the observed decrease can be directly attributed to the implementation of non-pharmaceutical interventions or whether it simply represents a continuation of pre-existing trends. This suggests the need for cautious interpretation of the pandemic's impact and calls for a more in-depth investigation as additional data become available.

In conclusion, the substantial decline in the diarrhoeal disease burden since 1990, especially in young children, reflects the dedicated efforts to enhance WASH infrastructure, vaccination programmes, and access to oral rehydration therapy. Yet, considerable regional disparities persist, and the emergence of antibiotic resistance presents new challenges, calling for sustained efforts in vaccine research. Our study highlights the need for the implementation of holistic public health strategies that integrate WASH, nutrition, vaccination, and health-care accessibility to further reduce the diarrhoeal disease burden and bridge global health disparities.

### GBD 2021 Diarrhoeal Diseases Collaborators

### Affiliations

### Contributors

### Data sharing

In compliance with the Guidelines for Accurate and Transparent Health Estimates Reporting (GATHER), we have made the input data sources and the code for each step of the estimation process publicly available on the Global Health Data Exchange at https://ghdx.healthdata.org/gbd-2021.

## Declaration of interests

S Afzal reports support for the present manuscript from the provision of study material from HEC digital library, Pakistan; Payment or honoraria for educational events and webinars with King Edward Medical University and collaborative partners including Johns Hopkins University, University of California, and the University of Massachusetts; support for attending meetings and/or travel from King Edward Medical University; participation on a data safety monitoring board or advisory board with the National Bioethics Committee (Pakistan), King Edward Medical University Institutional Ethical Review Board, Ethical Review Board Fatima Jinnah Medical University, and Sir Ganga Ram Hospital; leadership or fiduciary role in other board, society, committee or advocacy group, paid or unpaid with Pakistan Association of Medical Editors, Fellow of Faculty of Public Health Royal Colleges UK (FFPH), Society of Prevention, Advocacy and Research (King Edward Medical University SPARK), and as a member of Pakistan Society of Infectious Diseases; other financial or non-financial interests serving as Dean of Public Health and Preventative Medicine of King Edward Medical University, as Chief Editor of *Annals of King Edward Medical University*, Directory Quality Enhancement Cell King Edward Medical University, member of Research and Publications Higher Education Commission (Pakistan), and member of the Pakistan Medical and Dental Council for Research and Publications Committee; outside the submitted work. T Bärnighausen reports grants or contracts paid to their institution from the National Institutes of Health, Alexander von Humboldt Foundation, German National Research Foundation (DFG), European Union, German Ministry of Education and Research, German Ministry of the Environment, Wellcome, and kfW; payment or honoraria for lectures, presentations, speakers bureaus, manuscript writing or educational events from *PLOS* as the Editor-in-Chief; serving on two scientific advisory boards for NIH-funded research projects in Africa on climate change and health; stock or stock options in CHEERS; outside the submitted work. A Beloukas reports grants or contracts from Gilead (research grant and sponsorship to the University of West Attica) and GSK/ViiV (research sponsorship to the University of West Attica); payment or honoraria for lectures, presentations, speakers bureaus, manuscript writing, or educational events from Gilead, GSK/ViiV paid to the University of West Attica; support for attending meetings and/or travel from Gilead, GSK/ViiV paid to the University of Attica; and receipt of equipment, materials, drugs, medical writing, gifts or other services from Cepheid; outside the submitted work. M Ilic reports support for the present manuscript from the Ministry of Science, Technological Development and Innovation of the Republic of Serbia (no. 451-03-47/2023-01/20011). I Ilic reports support for the present manuscript from the Ministry of Science, Technological Development and Innovation of the Republic of Serbia (no. 175042, 2011-2023). N E Ismail reports leadership or fiduciary roles in other board, society, committee or advocacy group, unpaid as the Bursar and Council Member of Malaysian Academy of Pharmacy, and as Committee Member of Education Chapter of the Malaysian Pharmacists Society; outside the submitted work. J Jozwiak reports payment or honoraria for lectures, presentations, speakers bureaus, manuscript writing or educational events from Novartis, Adamed, and Amgen; outside the submitted work. K Krishan reports other non-financial support from the UGC Centre of Advanced Study, CAS II, awarded to the Department of Anthropology at Punjab University in Chandigarh (India); outside the submitted work. M-C Li reports support for the present manuscript from the National Science and Technology Council, Taiwan (NSTC 113-2314-B-003-002); leadership or fiduciary roles in other board, society, committee or advocacy groups, paid or unpaid as the Technical Editor of the *Journal of the American Heart Association*; outside the submitted work. L Monasta reports support for the present manuscript from the Italian Ministry of Health (Ricerca Corrente 34/2017), payments made to the Institute for Maternal and Child Health IRCCS Burlo Garofolo. J F Mosser reports support for the present manuscript from the Bill & Melinda Gates Foundation; grant funding from Gavi; support for attending meetings and/or travel from the Bill & Melinda Gates Foundation; outside the submitted work. I N Okeke reports grants or contracts from the Bill & Melinda Gates Foundation, Wellcome Trust Sanger Institute, University of Ibadan, Oxford University, Danish Technical University, and receipt of the International Vaccine Institute Award as the Nigeria Country PI until 2021; royalties for *Genetics: Genes, Genomes and Evolution* (Oxford University Press), royalties for *Divining Without Seeds: The case for strengthening laboratory Medicine in Africa* (Cornell University Press), royalties for *Antimicrobial Resistance in Developing Countries* (Springer); consulting fees from Wellcome Trust, UK Proposal Review Panel; payment or honoraria for lectures, presentations, speakers bureaus, manuscript writing or educational events from the Microbiology Society; support for attending meetings and/or travel from the Bill & Melinda Gates Foundation, European Society for Clinical Microbiology and Infectious Disease, and the American Society for Microbiology; leadership or fiduciary role in other board, society, committee or advocacy group, paid or unpaid with the Thomas Bassir Biomedical Foundation Nigeria, the International Centre for Antimicrobial Resistance Solutions (ICARS) Technical Advisory Forum 2021–2023, and additional pro-bono roles; other financial or non-financial interests as Editor in Chief (2017–2021) of the *African Journal of Laboratory Medicine*, Senior Editor (2022–) of *Microbial Genomics*, Scientific Advisor (2020–2021) of *The Lancet Infectious Diseases*, Surveillance Lead (2017–) of AMR Technical Work Group, Nigeria Center for Disease Control, as Commissioner (2020–2022) on *The Lancet* Commission for Nigeria, and payment for media (Podcast) event from The Wellcome Trust (2024); outside the submitted work. A P Okekunle reports support for the present manuscript from the National Research Foundation of Korea funded by the Ministry of Science and ICT (2020H1D3A1A04081265); support for attending meetings and/or travel from the National Research Foundation of Korea funded by the Ministry of Science and ICT (2020H1D3A1A04081265), outside the submitted work. L Ronfani reports support for the present manuscript from Italian Ministry of Health (Ricerca Corrente 34/2017) payments made to the Institute for Maternal and Child Health IRCCS Burlo Garofolo. Y L Samodra reports leadership or fiduciary roles in other board, society, committee or advocacy groups, paid or unpaid from Benang Merah Research Center (Indonesia) as Co-founder; outside the submitted work. J Sanabria reports support for attending meetings and/or travel from continuous medical education grant from the University School of Medicine; several patents granted and pending but no royalties; and Participation on a data safety monitoring board or advisory board for quality assessment and assurance for the department; outside the submitted work. V Sharma reports other financial or non-financial support from DFSS (MHA)'s research project (DFSS28(1)2019/EMR/6) at Institute of Forensic Science & Criminology, Panjab University (Chandigarh, India); outside the submitted work. J A Singh reports consulting fees from ROMTech, Atheneum, Clearview healthcare partners, American College of Rheumatology, Yale, Hulio, Horizon Pharmaceuticals, DINORA, ANI/Exeltis, USA Inc., Frictionless Solutions, Schipher, Crealta/Horizon, Medisys, Fidia, PK Med, Two labs Inc., Adept Field Solutions, Clinical Care options, Putnam associates, Focus forward, Navigant consulting, Spherix, MedIQ, Jupiter Life Science, UBM LLC, Trio Health, Medscape, WebMD, and Practice Point communications; and the National Institutes of Health; Payment or honoraria for lectures, presentations, speakers bureaus, manuscript writing or educational events as a member of the Speaker's Bureau of Simply Speaking; support for attending meetings and/or travel as a past steering committee member of OMERACT; participation on a data safety monitoring board or advisory board with FDA Arthritis Advisory Committee; leadership or fiduciary role in other board, society, committee or advocacy group, paid or unpaid as the Past steering committee member of the OMERACT, an international organization that develops measures for clinical trials and receives arm's length funding from 12 pharmaceutical companies, the Chair of the Veterans Affairs Rheumatology Field Advisory Committee, and the editor and the Director of the UAB Cochrane Musculoskeletal Group Satellite Center on Network Meta-analysis; stock or stock options in Atai life sciences, Kintara therapeutics, Intelligent Biosolutions, Acumen pharmaceutical, TPT Global Tech, Vaxart pharmaceuticals, Atyu biopharma, Adaptimmune Therapeutics, GeoVax Labs, Pieris Pharmaceuticals, Enzolytics, Seres Therapeutics, Tonix Pharmaceuticals Holding Corp., Aebona Pharmaceuticals, and Charlotte's Web Holdings, and previously owned stock options in Amarin, Viking and Moderna pharmaceuticals; outside the submitted work. J H V Ticoalu reports leadership or fiduciary role in other board, society, committee or advocacy group, paid or unpaid as the Co-founder of Benang Merah Research Center (Indonesia); outside the submitted work. S J Tromans reports grants or contracts from the 2023 Adult Psychiatric Morbidity Survey team, collecting epidemiological data on community-based adults living in England, a contracted study from NHS Digital via the Department of Health and Social Care (payments made to the University of Leicester); outside the submitted work.
