# Supplementary material for: Global, regional, and national age-sex-specific burden of diarrhoeal diseases, their risk factors, and aetiologies, 1990–2021, for 204 countries and territories: a systematic analysis for the Global Burden of Disease Study 2021
Source: Lancet Infect Dis. 2025 May;25(5):519–36. doi: 10.1016/S1473-3099(24)00691-1 (PMC12018300; doi:10.1016/S1473-3099(24)00691-1)
Supplement: Supplementary appendix 1 [file mmc1.pdf]

THE LANCET
Infectious Diseases

## Supplementary appendix 1

This appendix formed part of the original submission and has been peer reviewed. We post it as supplied by the authors.

Supplement to: GBD 2021 Diarrhoeal Diseases Collaborators. Global, regional, and national age-sex-specific burden of diarrhoeal diseases, their risk factors, and aetiologies, 1990–2021, for 204 countries and territories: a systematic analysis for the Global Burden of Disease Study 2021. *Lancet Infect Dis* 2024; published online Dec 18. https://doi.org/10.1016/S1473-3099(24)00691-1.

# Appendix 1

## Supplementary Methods Appendix to "Global, regional, and national age-sex-specific burden of diarrhoeal diseases, its risk factors, and aetiologies, 1990-2021, for 204 countries and territories: a systematic analyses for the Global Burden of Disease Study 2021"

This appendix provides further methodological detail and results for "Global, regional, and national age-sex-specific burden of diarrhoeal diseases, its risk factors, and aetiologies, 1990-2021, for 204 countries and territories: a systematic analyses for the Global Burden of Disease Study 2021."

All the material in the paper itself is novel. However, the parts of the supplemental methods appendix include sections adapted from the GBD Capstones published in The Lancet,[1–3] and references are provided for reproduced sections.


1. Naghavi M, Ong KL, Aali A, et al. Global burden of 288 causes of death and life expectancy decomposition in 204 countries and territories and 811 subnational locations, 1990–2021: a systematic analysis for the Global Burden of Disease Study 2021. The Lancet. 2024 May 18;403(10440):2100-32.
2. Ferrari AJ, Santomauro DF, Aali A, et al. Global incidence, prevalence, years lived with disability (YLDs), disability-adjusted life-years (DALYs), and healthy life expectancy (HALE) for 371 diseases and injuries in 204 countries and territories and 811 subnational locations, 1990–2021: a systematic analysis for the Global Burden of Disease Study 2021. The Lancet. 2024 May 18;403(10440):2133-61.
3. Brauer M, Roth GA, Aravkin AY, Zheng P, Abate KH, Abate YH, Abbafati C, Abbasgholizadeh R, Abbasi MA, Abbasian M, Abbasifard M. Global burden and strength of evidence for 88 risk factors in 204 countries and 811 subnational locations, 1990–2021: a systematic analysis for the Global Burden of Disease Study 2021. The Lancet. 2024 May 18;403(10440):2162-203.

## Table of Contents


***GBD 2021 Super Regions*** ........ **5**

***Diarrhoeal disease mortality*** ........ **6**

**Flowchart** ........ **6**

**Input data** ........ **6**

**Causes of death data quality (star rating) calculation[1]** ........ **6**

**HIV/AIDS misclassification correction[1]** ........ **11**

**Modelling strategy** ........ **12**

***Diarrhoeal disease morbidity*** ........ **15**

**Flowchart** ........ **15**

**Case definition** ........ **15**

**Input data** ........ **15**

**Modelling strategy** ........ **18**

***Diarrhoeal Aetiologies*** ........ **21**

***Diarrhoeal references*** ........ **29**

***Unsafe water*** ........ **31**

**Flowchart** ........ **31**

**Input data and methodological summary** ........ **31**

**Exposure** ........ **31**

*Case definition* ........ 31

*Input data* ........ 31

*Modelling strategy* ........ 32

**Theoretical minimum-risk exposure level** ........ **35**

**Relative risks** ........ **35**

*Input data* ........ 35

*Modelling strategy* ........ 36

**Unsafe Water References** ........ **38**

***Unsafe sanitation*** ........ **38**

**Flowchart** ........ **38**

**Input data and methodological summary** ........ **39**

Exposure ........ 39

Modelling ........ 39

**Theoretical minimum-risk exposure level** ........ **40**

**Relative risks** ........ **40**

**Unsafe Sanitation References** ........ **42**

***No access to handwashing facility*** ........ ***43***

**Flowchart** ........ **43**

**Input data and methodological summary** ........ **43**

**Exposure** ........ **43**

*Case definition* ........ 43

Input data ........ 43

*Modelling strategy* ........ 43

**Theoretical minimum-risk exposure level** ........ **44**

**Relative risks** ........ **44**

*Input data* ........ 44

*Modelling strategy* ........ 44

**No access to handwashing facility References** ........ **46**

***Child growth failure*** ........ ***47***

**Combined Flowchart for Child Growth Failure and Protein Energy Malnutrition** ........ **47**

**Input data and methodological summary** ........ **48**

Exposure ........ 48

**Modelling strategy** ***Exposure estimation*** ........ **49**

**Theoretical minimum-risk exposure level** ........ **50**

**Relative risks** ........ **50**

**Child Growth Failure References** ........ **53**

***Low birthweight and short gestation*** ........ ***54***

**Flowchart** ........ **54**

**Input data and methodological summary** ........ **55**

Case definition ........ 56

Input data and data processing ........ 57

**Table 4. Input data for exposure models** ........ **58**

*Data processing* ........ 58

Modelling strategy ........ 62

**Relative risks and theoretical minimum risk exposure level** ........ **64**

Causes ........ 64

Input data ........ 65

Modelling strategy ........ 65

Population attributable fraction ........ 66

**Low Birthweight and Short Gestation References** ........ **66**

*Suboptimal breastfeeding* .......... *67*
Flowchart .......... 67
Input data and methodological summary .......... 67
Definition .......... 67
Exposure .......... 67
Input data .......... 68
Exposure .......... 68
Relative risk .......... 69
Modelling strategy .......... 69
Theoretical minimum-risk exposure level .......... 70
Relative risk .......... 70
Population attributable fraction .......... 71
Suboptimal Breastfeeding References .......... 71
Justification for the counterfactual approach .......... 72
Counterfactual Approach References .......... 72
Estimation of population attributable fractions[3] .......... 72
Calculating the burden of multiple risk factors[3] .......... 73
Risk factors and causal pathways leading to diarrhoeal diseases .......... 74
Risk Factors and Causal Pathways: References .......... 74
*Statement of GATHER Compliance* .......... *75*
*Ethics Statement* .......... *76*
*Authorship Material* .......... *77*
GBD 2021 Diarrhoeal Disease Collaborators .......... 77
Affiliations .......... 79
Authors' Contributions .......... 95
Managing the overall research enterprise .......... 95
Writing the first draft of the manuscript .......... 95
Primary responsibility for applying analytical methods to produce estimates .......... 95
Primary responsibility for seeking, cataloguing, extracting, or cleaning data; designing or coding figures and tables .......... 95
Providing data or critical feedback on data sources .......... 95
Developing methods or computational machinery .......... 96
Providing critical feedback on methods or results .......... 97
Drafting the work or revising it critically for important intellectual content .......... 99
Managing the estimation or publication process .......... 101

# GBD 2021 Super Regions

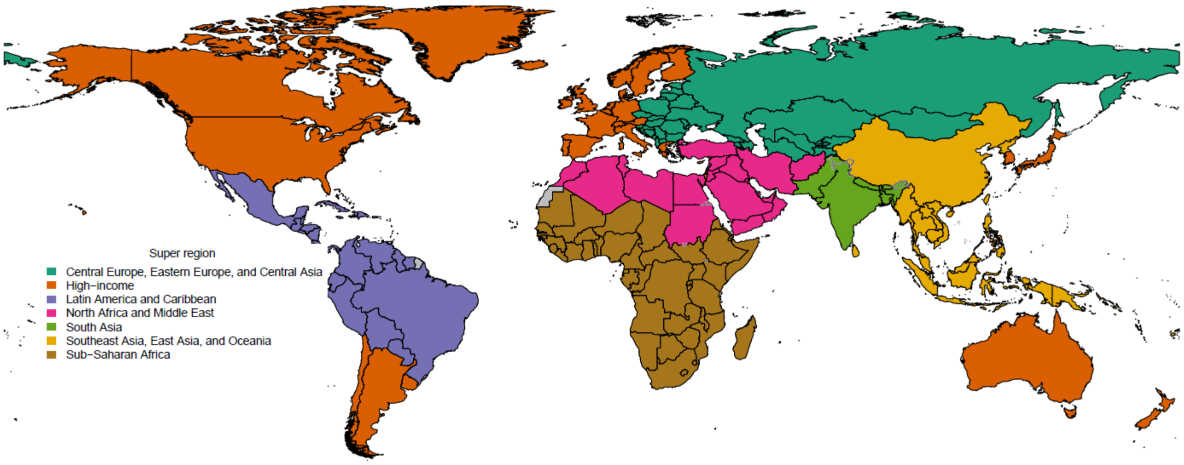

# Diarrhoeal disease mortality

## Flowchart

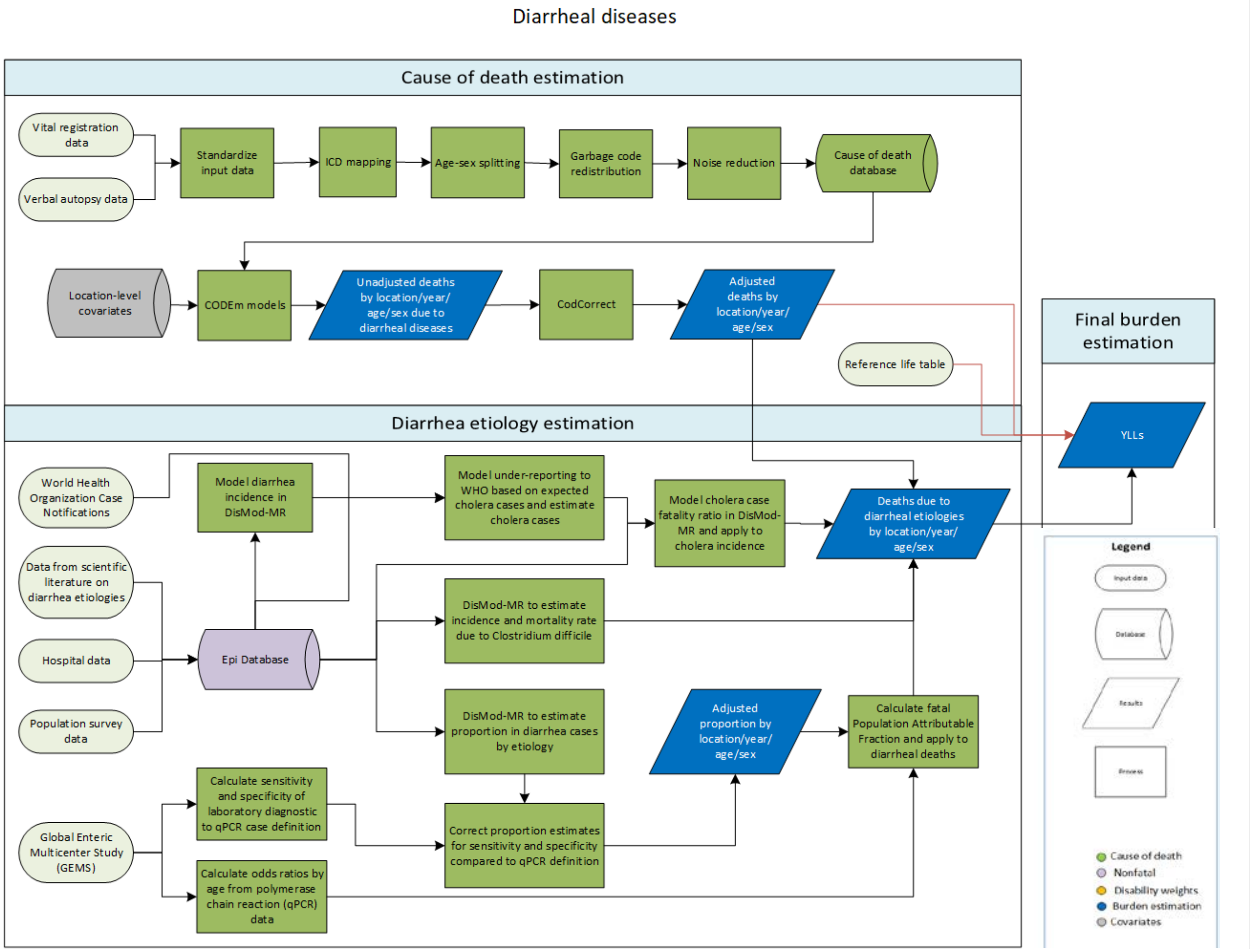


Diarrhoeal diseases are a cause of death in GBD. We also estimated the attributable deaths from 13 diarrhoeal aetiologies using an independent modelling strategy. These pathways are shown in the flowchart above and described in detail below in this report.

## Input data

***Cause of death.*** To estimate mortality from diarrheal diseases, the input data comprised vital registration data across 24,181 site-years (i.e., the total number of years of data available for each geography), 825 site-years of sample vital registration data, 1,785 site-years of data from verbal autopsy studies, 575 site-years from mortality surveillance, and 9 site-years from minimally invasive tissue sampling.

## Causes of death data quality (star rating) calculation[1]

GBD estimates are most accurate when computed with a full time series of complete VR with a low percentage of garbage codes. For GBD 2016, we developed a simple star-rating system from 0 to 5 to give

a picture of the quality of data available in a given country over the full time series used in GBD estimates. Countries improve in the star rating as they increase availability, completeness, and detail of their mortality data and reduce the percentage of deaths coded to ill-defined garbage codes or highly aggregated causes.

We assign star ratings to rate the quality of data for any given location-year. Two dimensions determine this star rating: (1) the level of completeness of death registration and (2) the percentage of total deaths determined to be major garbage or highly aggregated cause. These two values were used to create a "percentage well-certified" value between 0 and 1, determined as:

$$pct_{wellcertified} = \ completeness \times (1 - pct_{majgarbage})$$

Where:

$$completeness = \frac{registered\ deaths}{GBD\ mortality\ envelope}$$

$$pct_{majgarbage} = \frac{deaths\ coded\ to\ level\ 1\ or\ 2\ garbage\ or\ highly\ aggregated\ cause}{registered\ deaths}$$

For each verbal autopsy (VA) data source, percent well-certified is:

$$pct_{wellcertified} = \ VerbalAutopsyAdjustment \times (1 - pct_{majgarbage})$$

Where:

$$VerbalAutopsyAdjustment = \ SubAdj \times RegAdj \times AgeSexCoverage$$

And:

*SubAdj* represents 10% for subnationally representative studies, 100% for nationally representative studies. This adjustment, while arbitrary in its specific value, reflects the bias that can be associated with studies that only cover a potentially non-representative sample of a country's population. *RegAdj* represents 64% for all VA data sources. This accounts for the inaccuracy of VA in assigning CoD compared to medically verified VR. The specific multiplier 0·64 is based on the chance-corrected concordance of Physician Certified Verbal Autopsy (PCVA) versus medical certification by the Population Health Metrics Research Consortium. *Age-Sex Coverage* reflects the number of deaths estimated in the GBD mortality envelope for the ages and sexes in the study for the country and year divided by the number of deaths estimated in the GBD mortality envelope for the country and year. Studies that only cover children under 5 or maternal mortality, for example, will be highly discounted by this multiplier. In the case of VA, all garbage codes are considered ill-defined, as redistribution for VA is highly imprecise.

Once percent well-certified is calculated for each location-year of VR and each VA study-year, we then combine these into one measurement for each five-year time interval and the full time series 1980–2020. For each five-year time interval, we take the maximum percent well-certified. Then for 1980–2020, we

take the average of the maximum percentages well-certified for the seven five-year time intervals, including any five-year time interval where no data were available as a zero.

Once these values are calculated, we assign stars as follows:

5 stars: 85%–100% well-certified

4 stars: 65%–84% well-certified

3 stars: 35%–64% well-certified

2 stars: 10%–34% well-certified

1 star: >0%–9% well-certified

0 stars: No VR or VA data available or 0% well-certified

While stars are calculated for each five-year time interval, as well as the full time series from 1980 to 2020, stars in the figure below are presented for the full time series.

Overall data quality by country

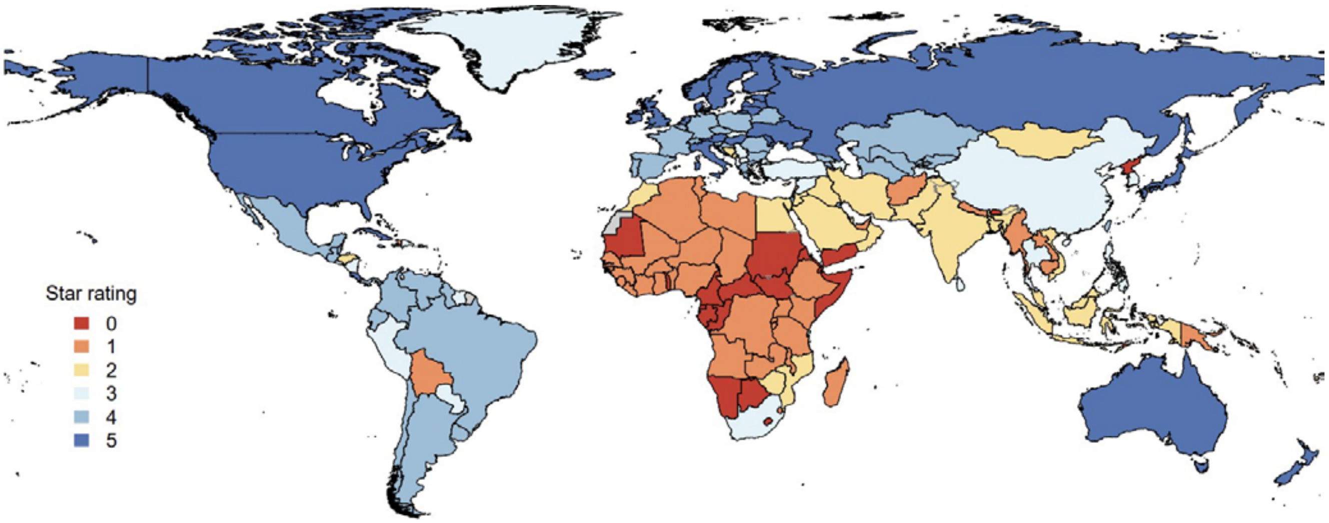


Processing cause of death data to enhance comparability and utility[1]

*Redistribution*

A crucial aspect of enhancing the comparability of data for causes of death (CoD) is to deal with uninformative, so-called garbage codes. Garbage codes to which deaths were assigned should not be considered as the underlying CoD, for example: “heart failure”, “ill-defined cancer site”, “senility”, “ill-defined external causes of injuries”, and “septicaemia”. Redistribution is therefore the process of reallocating garbage-coded deaths to plausible underlying causes. For each group of diagnostically related garbage codes, we define a set of probable underlying causes of death and the proportion of garbage-coded deaths that are redistributed to each underlying cause, separately by GBD age group, sex, location, and

year. The methods for redistributing these garbage-coded deaths have been previously described in detail.[1] While our underlying algorithm has not changed significantly since GBD 2013, several improvements were made in GBD 2019 and GBD 2021.

For each redistribution package, we defined the "universe" of data as all deaths coded to either the package's garbage codes or the package's redistribution targets for each country, year, age, and sex. We then ran a regression based on the following equation, separately for each target group and sex:

$$TG_{crt} = \alpha + \beta_1 Gar_{crt} + \beta_2 Age_{crt} Gar_{crt} + \theta_r Gar_{crt} + \gamma_r + \varepsilon_{ct}$$

Where:

$TG_{crt}$ = percentage of deaths within the given garbage code's universe which were coded to a given target group, by country

$Gar_{crt}$ = percentage of deaths within the given garbage code's universe which were coded to a given set of garbage codes

$\alpha$ = constant

$\beta_1 =$ slope coefficient describing the association between $Gar_{crt}$ and $TG_{crt}$

$\beta_2 =$ slope coefficient describing the association between the interaction $Age_{crt}Gar_{crt}$ and $G_{crt}$

$\gamma_r =$ region-specific random intercept (or super-region if the random effect on region is not significant)

$\theta_r =$ region-specific random slope (or super-region if the random effect on region is not significant)

$\varepsilon_{ct} =$ standard error, normally distributed and calculated by bootstrapping

This regression was adjusted from GBD 2013 to include fixed effects on the interaction of garbage and age to ensure smooth age patterns. The random effects on location were included to help capture geographic differences in garbage coding for various causes. The regressions were first run with a random effect on the region, and in the case of failed convergence, they were attempted again with a random effect on the superregion. When models using a random effect on the superregion failed to converge, a fixed effect model considering only age was settled upon. We made this decision after investigating diagnostic visualisations that showed unlikely gaps between proportions assigned to different age groups.

*Computing redistribution uncertainty*

We assigned redistribution variance to each data point by calculating residual variance from a regression predicting the percentage of garbage coded deaths redistributed to a cause, given the proportion of garbage codes we observed for that location, year, age, sex, cause, and the age standardized relative rate of major garbage codes across all causes. If there is a cause that has greater residual variance, we assume greater redistribution uncertainty.

In order to calculate variance, a dataset was generated containing percent garbage by location, year, age, sex and cause, where percent garbage is:

$$pct_{garbage} = \frac{deaths_{redistributed} - deaths_{raw}}{deaths_{redistributed}}$$

A mixed-effect linear regression model was then fit to predict the logit percent of deaths from redistribution by age-standardized relative rate of major garbage.

$$logit\left(pct_{garbage_{ij}}\right) = \beta_0 + \beta_1 * \log\left(ASR_{majorgarbage_{ij}}\right) + \beta_2 * 15yearage_{ij} + \gamma_{1j} * \log\left(ASR_{majorgarbage_{ij}}\right) + u_j + e_{ij},\ \ \theta_{\{i\}} \sim N(0, \sigma^2)$$

Where:

*i* indexes dataset-location-year-age-sex-cause data points nested within *j* groups by GBD region

ASR major garbage: age standardized relative rate of major garbage

Residual variance, as estimated by the median absolute deviation (MAD), was calculated for each cause, sex, and age. The next step was to use the residual variance to calculate uncertainty around each data point in the CoD database. First, we calculated the percent garbage of each data point, treating all deaths that could not be directly mapped to a GBD cause as garbage, including level four garbage. Percent garbage was calculated as:

$$pct_{garbage} = \frac{deaths_{redistributed} - deaths_{corrected}}{deaths_{corrected}}$$

Where:

death corrected: deaths post misdiagnosis correction (Appendix Section 2.6)

deaths redistributed: deaths post redistribution (Appendix Section 2.7)

Residual variance was matched to each data point and one hundred draws were sampled from a normal distribution using the cause, age, sex, specific residual variance and mean of zero. The logit transformed

percent garbage was added to each value in the distribution. Each draw was then transformed out of logit space and the post-redistribution deaths were calculated as:

$$deaths = \frac{deaths_{corrected}}{1 - pct\_garbage}$$

Draws of deaths were processed through noise reduction before calculating the final redistribution variance passed to CODEm, which was added to the total data variance. The mean of the draws was not used as the final estimate, because it was found that the logit transformation biases the distribution of cause fractions higher than if only point estimates are used.

## HIV/AIDS misclassification correction[1]

In many location-years, certain causes of death known to be comorbid with HIV/AIDS (eg, tuberculosis, other infectious diseases) are seen to have age patterns that diverge from those observed in location-years without widespread HIV epidemics and are in fact more reflective of HIV mortality trends. In order to identify these instances, a global relative age pattern is generated using all VR deaths in countries with observed HIV prevalence less than 1% using the following:

$$RR_{asc} = \frac{R_{asc}}{\bar{x}(R_{65sc}, R_{70sc}, R_{75sc})}$$

Where $RR_{asc}$ is the relative death rate for age group *a*, sex *s*, and cause *c*; $R_{asc}$ is the rate for that age group; and $\bar{x}(R_{65sc}, R_{70sc}, R_{75sc})$ is the mean of the rates in ages 65–69, 60–74, and 75–79 for that sex and cause. This is preferable to comparing mortality rates because we are able to isolate divergence in age pattern while accounting for varying levels of overall mortality by fixing death rates to age groups that are unlikely to be confounded by the presence of HIV. Expected deaths for an identified cause were then determined to be:

$$ED_{lyasc} = \bar{x}\left(R_{ly65sc}, R_{ly70sc}, R_{ly75sc}\right) \times p_{lasc} \times RR_{asc}$$

Where $ED_{lasc}$ are deaths for location *l*, year *y*, age group *a*, sex *s*, and cause *c*; $\bar{x}(R_{l65sc}, R_{l70sc}, R_{l75sc})$ is the mean of the rates for ages 65–69, 60–74, and 75–79 for that location-year-sex-cause; $p_{lasc}$ is the population for that location-year-age-sex-cause; and $RR_{asc}$ is the global standard relative rate determined in the previous step for that age-sex-cause. The expected deaths remain attributed to that particular cause, while the difference between observed and expected are reallocated to HIV/AIDS.

Diarrhoeal cause-of-death data availability

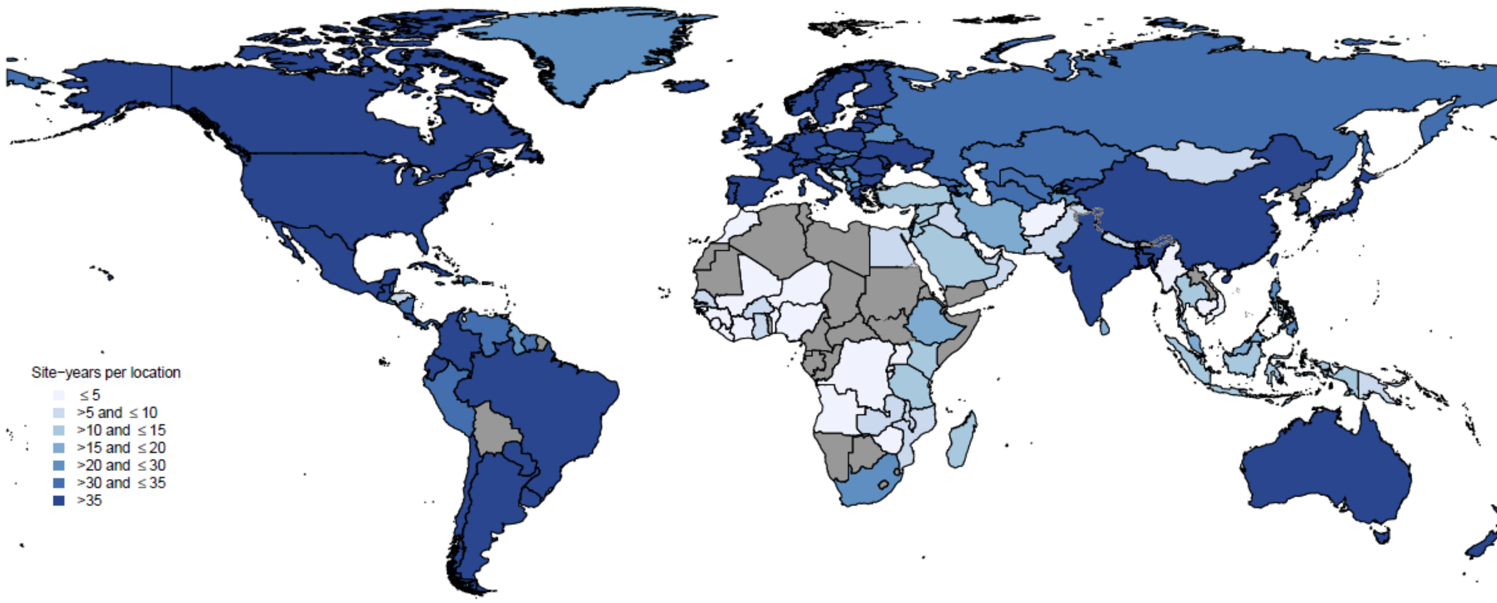


## Modelling strategy

***Cause of death.*** Diarrhoeal disease mortality was estimated in the Cause of Death Ensemble modelling platform (CODEm). We estimated diarrhoea mortality separately for males and females and for children under 5 years and older than 5 years. We used country-level covariates to inform our CODEm models (**Table 1**).

We modelled deaths due to diarrhoeal disease with two CODEm models, separately for each sex and two age categories (under 5 and 5 years and above), as the mortality trends differ substantially between these age groups. The final sex-specific models for deaths due to diarrhoeal disease were a hybridised model of separate global and data-rich models for males and females.

In the CODEm framework,[1] four families of statistical models are used: linear mixed effects regression (LMER) models of the natural log of the cause-specific death rate, LMER models of the logit of the cause fraction, spatiotemporal Gaussian process regression (ST-GPR) models of the natural logarithm of the cause-specific death rate, and ST-GPR models of the logit of the cause fraction (see the 2x2 table in Foreman et al).[7] For each family of models, all plausible relationships between covariates and the response variable are identified. Based on the evidence of a causal relationship with diarrhoeal disease mortality, covariates are ranked from 1 (proximally related) to 3 (distally related). The direction of the association between each covariate and diarrhoeal disease mortality is assigned as a prior based on the literature (Appendix Tables 1A and 1B). Because all possible combinations of selected covariates are considered for each family of models, multi-collinearity between covariates may produce implausible signs on coefficients or unstable coefficients. Each combination is therefore tested for statistical significance (covariate coefficients must have a coefficient with p-value < 0·05) and plausibility (the coefficients must have the directions expected on the basis of the

literature). Only covariate combinations meeting these criteria are retained. This selection process is run for both cause fractions and death rates, then ST-GPR and LMER-only models are created for each set of covariates. For locations with sparse data, we leverage regional patterns and covariates to assist in making predictions for these areas. As a result, the estimates for such locations are associated with wider uncertainty intervals.

The families of models that go through ST-GPR incorporate information about data variance. The main inputs for a Gaussian process regression (GPR) are a mean function, a covariance function, and data variance for each data point. These inputs are described in detail in Foreman et al.[7] Three components of data variance are now used in CODEm: sampling variance, non-sampling variance, and garbage code redistribution variance. The computation of sampling variance and non-sampling variance has not changed since previous iterations of the GBD and is also described in previous publications.[1,7] Garbage code redistribution variance is computed in the CoD database process. Since variance is additive, we calculate total data variance as the sum of sampling variance, non-sampling variance, and redistribution variance. Increased data variance in GPR may result in the GPR draws not following the data point as closely.

The performance of all models (individual and ensemble) is evaluated by means of out-of-sample predictive validity tests. Thirty percent of the data are randomly excluded from the initial model fits. These individual model fits are evaluated and ranked by using half of the excluded data (15% of the total), then used to construct the ensembles on the basis of their performance. Data are held out from the analysis on the basis of the cause-specific missingness patterns for ages and years across locations. Out-of-sample predictive validity testing is repeated 20 times for each model, which has been shown to produce stable results. These performance tests include the root mean square error (RMSE) for the log of diarrhoeal disease death rate, the direction of the predicted versus actual trend in the data, and the coverage of the predicted 95% UI.

The component models are weighted on the basis of their predictive validity rank to determine their contribution to the ensemble estimate. The relative weights are determined both by the model ranks and by a parameter ψ, whose value determines how quickly the weights taper off as rank decreases. The distribution of ψ is described in more detail in Foreman et al. A set of ensemble models is then created by using the weights constructed from the combinations of ranks and ψ values. These ensembles are tested by using the predictive validity metrics described in the previous section on the remaining 15% of the data, and the ensemble with the best performance in out-of-sample trend and RMSE is chosen as the final model. Lastly, 1000 draws are created for the final ensemble, and the number of draws contributed by each model is proportional to its weight. The mean of the draws is used as the final estimate for the CODEm process, and a 95% UI is created from the 0·025 and 0·975 quantiles of the draws.

Similar to other models of mortality in GBD, diarrhoeal disease mortality models are single-cause, requiring that the sum of all mortality models must be equal to the all-cause mortality envelope. We correct diarrhoeal disease mortality estimates, and other causes of mortality, by rescaling them according to the uncertainty around the cause-specific mortality rate. This process is called CoDCorrect and is essential to ensure internal consistency among causes of death.

**Table 1. Covariates used in diarrhoea mortality modelling. Table 1A shows the covariates used in the 0–4 years model, and Table 1B shows the covariates used in the 5–95+ years model.** The *Level* represents the strength of the association between the covariate and diarrhoea mortality from 1 (proximally related) to 3 (distally related). The *Direction* indicates the positive or negative association between the covariate and diarrhoea mortality.

**Table 1A. Covariates used in the 0–4 years model**

| Level | Covariate | Direction |
|---|---|---|
| 1 | Oral rehydration solution treatment | - |
| | Rotavirus vaccine coverage (proportion) | - |
| | Sanitation (proportion with access) | - |
| | Improved water source (proportion with access) | - |
| 2 | Zinc deficiency | + |
| | Zinc treatment for diarrhoea | - |
| | Healthcare Access and Quality Index | - |
| 3 | Maternal education years | - |
| | Socio-demographic Index (SDI) | - |
| | Lag distributed income (LDI) per capita | - |
| | No access to handwashing facility | + |

**Table 1B. Covariates used in the 5–95+ years model**

| Level | Covariate | Direction |
|---|---|---|
| 1 | Sanitation (proportion with access) | - |
| | Improved water source (proportion with access) | - |
| 2 | Healthcare Access and Quality Index | - |
| | Rotavirus vaccine coverage (proportion) | - |
| | Oral rehydration solution treatment | - |
| 3 | Education years per capita | - |
| | Lag distributed income (LDI) per capita | - |
| | Adult underweight | + |
| | Socio-demographic Index (SDI) | - |
| | No access to handwashing facility | + |

# Diarrhoeal disease morbidity

## Flowchart

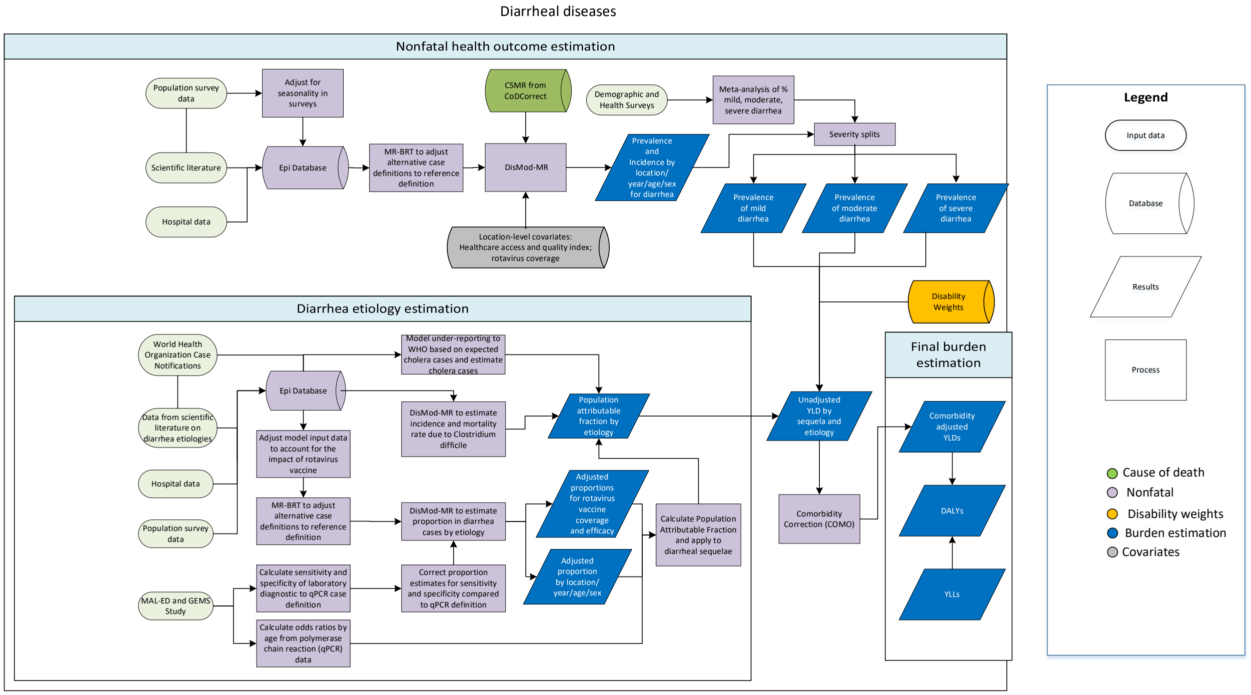


## Case definition

We defined diarrhoeal disease episodes as three or more loose stools in a 24-hour period. In the diarrhoea models, self-reported prevalence is the reference category for all data adjustments. Hospital input data use ICD-9 codes 001-009.9 and ICD-10 codes A00-A09.

The case definitions accepted for diarrhoea are shown below.

| Quantity of interest | Reference or alternative | Definition |
|---|---|---|
| Incidence or prevalence of diarrhoea | Reference | Three or more abnormally loose stools in a 24-hour period. Self-reported or parental report for children. |
| Incidence of inpatient diarrhoea episodes | Alternative | Incidence of diarrhoea episodes that become inpatients reported in health care data. |
| Incidence of diarrhoea episodes in clinical claims data | Alternative | Incidence of diarrhoea episodes reported in claims data. |

## Input data

*Model inputs*

We used three main types of data in the diarrhoea non-fatal burden estimation: hospital data, population-based surveys, and data from scientific literature.

The first type of data is the incidence of diarrhoea in hospital settings, including inpatient, outpatient, and claims data. These data were identified using the ICD-9 codes 001-009.9 and ICD-10 codes A00-A09 and were adjusted prior to modelling for multiple admissions and multiple diagnoses. To be consistent with the population-based survey data, adjusted hospital data were transformed from incidence to prevalence using the following equation:

$$Prevalence = Incidence * \frac{duration(days)}{365}$$

The second type of data are from population-representative surveys, such as the Demographic and Health Surveys and the Multiple Indicator Cluster Surveys. We converted the prevalence of maternal-reported two-week period from surveys to point prevalence in one-year age groups using this equation:

$$Point\ Prevalence = Period\ Prevalence * \frac{Duration}{(Recall\ Period + Duration - 1)}$$

Where the mean duration was the duration in days, an average of 4.3 days (4.2–4.4) in both equations.[14]

Survey data were adjusted for seasonality. Surveys are frequently conducted over several months. To account for seasonal variation in diarrhoea prevalence, we fit a mixed-effects generalised additive model for each GBD region with a forced periodicity and a random intercept by country. The ratio between the monthly model-fit diarrhoea prevalence and the corresponding regional diarrhoea prevalence is a scalar to adjust survey data by month and geography.

The third type of data are from scientific literature. Inclusion criteria include diarrhoea as the case definition, studies with a sample size of at least 100, and a study duration of at least one year to avoid bias in the seasonal timing of diarrhoea. We excluded studies that reported on diarrhoeal outbreaks exclusively and studies that combined acute gastroenteritis with and without diarrhoea. We included all literature data sources used in GBD 2019 and conducted an updated review of literature for GBD 2021 covering the period 2/7/2019 to 1/3/2020 for diarrhoea prevalence, incidence, and all diarrhoea aetiologies.

Newly identified sources were added to studies and sources identified in previous rounds of the GBD, representing data from 199 countries **(Table 1)**.

**Table 1. Unique source counts for diarrhoeal diseases by measure**

| Measure | Total sources | Countries with data |
|---|---|---|
| Prevalence | 1292 | 169 |
| Other | 450 | 116 |

### *Data crosswalks*

One of the GBD core principles is to use all available data to inform our estimates. To account for differences between studies, we conducted a meta-regression of the ratio of reference to non-reference data using the

meta-regression—Bayesian, regularised, trimmed (MR-BRT) tool. When possible, crosswalks based on data matched within studies on age, sex, and location are used. When not possible, ratios between alternative and reference case definitions/methods were based on data matched between studies, nearby in age, year, with exact matches on sex and location. We adjusted inpatient data and claims data up to the level of self-reported data (our reference case definition) (**table 2**).

Table 2. Diarrhoeal disease crosswalk coefficients

| Data input | Reference or alternative case definition | Gamma | Crosswalk covariate | Beta coefficient, logit (95% UI) |
|---|---|---|---|---|
| Self-reported diarrhoea | ref | -- | -- | -- |
| Clinical and literature, inpatient | alt | 0.36 | intercept | –5.80 (–6.51 to –5.08) |
| Claims, MarketScan | alt | 0 | intercept | 0.07 (–0.11 to 0.26) |

*Age-sex splits*

Data were age- and sex-split based on population and a modelled age-curve generated using age-specific data as inputs in MR-BRT to better estimate the distribution of non-age-specific data.

*Severity split inputs*

Diarrhoeal diseases have three severity levels: mild, moderate, and severe (**Table 3**). The proportion of diarrhoea cases that are assigned to each comes from a systematic review of diarrhoea severity.[13] Mild cases are the proportion of diarrhoea cases that did not seek medical care (64.8%); moderate cases are the proportion that sought medical care but did not have severe dehydration or bloody stool (28.9%); and severe cases are the proportion that sought medical care with severe dehydration or bloody stool (6.9%). These proportions are based on the frequency of dehydration and bloody stool among community-based studies reported in the systematic review.

**Table 3. Severity splits,** details on the severity levels for diarrhoea in GBD 2021 and the associated disability weight (DW) with that severity.

| Severity level | Lay description | Disability weight (95% CI) | Proportion |
|---|---|---|---|
| Mild | Has diarrhoea defined as 3 or more loose stools in a 24-hour period with no dehydration. | 0.074 (0.049–0.104) | 64.8% |
| Moderate | Has diarrhoea defined as 3 or more loose stools in a 24-hour period with painful cramps and feeling thirsty and any dehydration. | 0.188 (0.125–0.264) | 28.9% |

| | | | |
|---|---|---|---|
| Severe | Has diarrhoea defined as 3 or more loose stools in a 24-hour period with painful cramps and is very thirsty or feels nauseated or tired and/or severely dehydrated. | 0.247 (0.164–0.348) | 6.9% |

## Modelling strategy

### *Diarrhoea incidence and prevalence*

The non-fatal diarrhoeal disease burden is modelled in DisMod-MR 2.1, a Bayesian meta-regression modelling framework. DisMod-MR produces estimates of the incidence, prevalence, and remission of diarrhoea for each age, sex, geographical location, and year. The reference category for our input data is community-based diarrhoea episodes such as data from population-representative surveys or community cohorts. As described in the data crosswalks section above, input data that are from a different population, such as hospital inpatient groups, are adjusted before modelling by determining a meta-regression ratio of non-reference to reference data values, so that they are consistent with the reference category. Before modeling in DisMod, we ran the MR-BRT (Meta-Regression with Bayesian priors, Regularization, and Trimming) tool with excess mortality rate (EMR) computed as cause-specific mortality (CSMR) divided by prevalence as input data and healthcare access and quality index (HAQ), age, and sex as covariates to predict EMR prior data for all locations. We assumed higher HAQ to be associated with lower EMR. To examine this assumption, we analyzed the model fit across all available data, as illustrated in Figure 4. In Figure 4, there are three distinct sets of model fit lines, representing different HAQ values: light blue lines for the minimum HAQ values, darker blue lines for the 50th percentile HAQ values, and green lines for the maximum HAQ values. Within these sets, we further differentiated between genders, with solid lines representing females and dotted lines representing males. This detailed visualization clearly demonstrates the inverse relationship we hypothesized for both males and females: the light blue lines, corresponding to the lowest HAQ values, estimate higher EMR compared to the dark blue and green lines, which represent higher HAQ values. We then used location-year-age-sex specific EMR prior data as an input to DisMod, which simultaneously models prevalence, incidence, EMR, and CSMR to produce internally consistent estimates. Country-level covariates are used to inform the model **(Table 4)**.

**Table 4. Covariates.** Summary of covariates used in the diarrhoea DisMod-MR meta-regression model

| Covariate | Type | Parameter | Exponentiated beta (95% uncertainty interval) |
|---|---|---|---|
| RotaC vaccine lagged in five-year coverage | Country-level | Prevalence | 0.20 (0.19 — 0.20) |
| Socio-demographic Index | Country-level | Prevalence | 0.13 (0.12 — 0.14)) |
| Sex | Study-level | Prevalence | 1.05 (1.04 — 1.06) |
| Healthcare Access and Quality Index | Country-level | Excess mortality rate | 0.94 (0.94–0.94) |
| Sex | Study-level | Excess mortality rate | 1.33 (1.32 — 1.34) |

Figure 4. Model predictions across HAQ levels against EMR input data (log space)

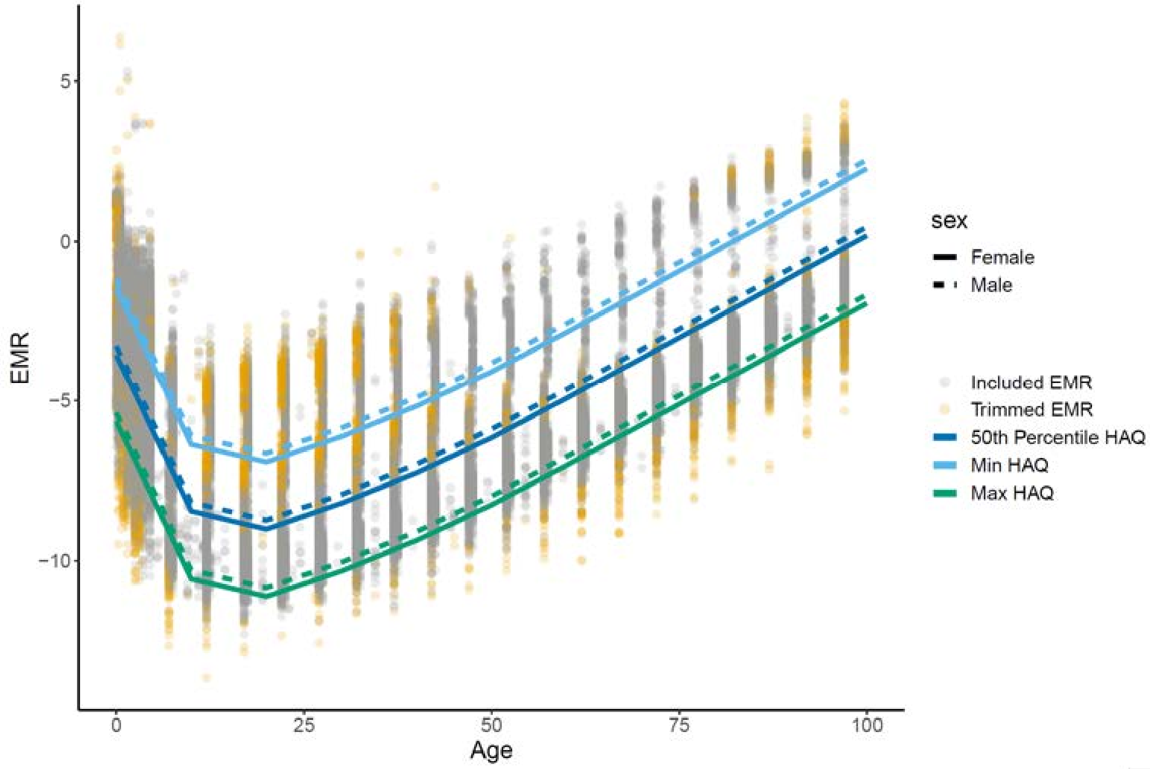


*DisMod-MR 2.1 description*[2]

The sequence of estimation in DisMod MR 2.1 occurs at five levels: global, super-region, region, country and, where applicable, subnational location. The super-region priors are generated at the global level with mixed-effects, nonlinear regression using all available data; the super-region fit, in turn, informs the region fit, and so on down the cascade. Subnational estimation was informed by the country fit and country covariates, plus an adjustment based on the average of the residuals between the subnational location's available data and it's prior. This mimicked the impact of a random effect on estimates between subnationals. At each level of the cascade, the DisMod-MR 2.1 enforces consistency between all parameters. Analysts have the choice to branch the cascade in terms of time and sex at different levels depending on data density. We used the default option to model diarrheal disease, which is to branch by sex after the global fit but to retain all years of data until the lowest level in the cascade.

The coefficients for country covariates were re-estimated at each level of the cascade. For a given location, country coefficients were calculated using both data and prior information available for that location. In GBD 2021, we generated model fits for the years 1990, 1995, 2000, 2005, 2010, 2015, 2017, 2019, 2020 and 2021, and log-linearly interpolated estimates for the intervening years. The 95% uncertainty intervals were computed based on 1000 draws from the posterior distribution of the model using the 2.5th and 97.5th percentiles of the ordered 1000 values.

*DisMod-MR 2.1 likelihood estimation*[2]

Analysts have the choice of using a Gaussian, log-Guassian, Laplace or Log-Laplace likelihood function in DisMod-MR 2.0. The default log-Gaussian equation for the data likelihood is:

$$-log\left[p\left(y_j|\Phi\right)\right] = \log\left(\sqrt{2\pi}\right) + \log\left(\delta_j + s_j\right) + \frac{1}{2}\left(\frac{\log\left(a_j + \eta_j\right) - \log\left(m_j + \eta_j\right)}{\delta_j + s_j}\right)^2$$

where, yj is a 'measurement value' (i.e. data point); Φ denotes all model random variables; ηj is the offset value, eta, for a particular 'integrand' (prevalence, incidence, remission, excess mortality rate, with-condition mortality rate, cause-specific mortality rate, relative risk or standardised mortality ratio) and aj is the adjusted measurement for data point j, defined by:

$$a_j = e^{(-u_j - c_j)} y_j$$

where $u_j$ is the total 'area effect' (i.e. the sum of the random effects at three levels of the cascade: super-region, region and country) and $c_j$ is the total covariate effect (i.e. the mean combined fixed effects for sex, study level and country level covariates), defined by:

$$c_j = \sum_{k=0}^{K[I(j)]-1} \beta_{I(j),k} \hat{X}_{k,j}$$

with standard deviation

$$s_j = \sum_{l=0}^{L[I(j)]-1} \zeta_{I(j),l} \hat{Z}_{k,j}$$

where *k* denotes the mean value of each data point in relation to a covariate (also called x-covariate); *I(j)* denotes a data point for a particular integrand, *j*; $\beta_{I(j),k}$ is the multiplier of the $k^{th}$ x-covariate for the $i^{th}$ integrand; $\hat{X}_{k,j}$ is the covariate value corresponding to the data point *j* for covariate *k*; *l* denotes the standard deviation of each data point in relation to a covariate (also called z-covariate); $\zeta_{I(j),k}$ is the multiplier of the $l^{th}$ z-covariate for the $i^{th}$ integrand; and $\delta_j$ is the standard deviation for adjusted measurement *j,* defined by:

$$\delta_j = log\left[y_j + e^{(-u_j - c_j)}\eta_j + c_j\right] - log\left[y_j + e^{(-u_j - c_j)}\eta_j\right]$$

Where $m_j$ denotes the model for the $j^{th}$ measurement, not counting effects or measurement noise and defined by:

$$m_j = \frac{1}{B(j) - A(j)} \int_{A(j)}^{B(j)} I_j(\text{a})\, \text{da}$$

where A(j) is the lower bound of the age range for a data point; B(j) is the upper bound of the age range for a data point; and I(j) denotes the function of age corresponding to the integrand for data point *j*.

The source code for DisMod-MR 2.1 as well as the wrapper code is available at the following link:

https://github.com/ihmeuw/ihmemodelling/tree/master/gbd_2017/shared_code/central_comp/nonfatal/dismo

d.

*A layman's description of DisMod MR 2.1*

DisMod-MR 2.1 is an advanced tool developed for the Global Burden of Disease study, designed to enhance the estimation of the prevalence and incidence of diseases across different geographies over time. Given that available data on prevalence, incidence, and mortality may be incomplete or limited, it employs a statistical triangulation method to provide more robust estimates. This method ensures that incidence, prevalence, and mortality estimates are internally consistent, which means they logically fit together given that prevalence is a function of incidence, recovery, and death rates. The estimation process utilizes a hierarchical approach that starts with global estimates and then refines these based on more localized data to produce estimates for 204 countries and territories over different time periods.

## Diarrhoeal Aetiologies

There are 13 aetiologies in GBD 2021 for diarrhoea: adenovirus, *Aeromonas, Campylobacter, Vibrio cholerae, Clostridium difficile, Cryptosporidium*, *Entamoeba histolytica,* Enterotoxigenic *Escherichia coli* producing heat-stable toxin (ST-ETEC), typical Enteropathogenic *Escherichia coli* (tEPEC), norovirus, rotavirus, non-typhoidal *Salmonella,* and *Shigella.* The input data to inform the proportion of diarrhoea cases that tested positive for each pathogen for all aetiologies except *C. difficile* include data from published studies identified via systematic review and the Global Pediatric Diarrhoea Surveillance Network. We completed an updated systematic literature review covering the period February 2019 to December 2020. The inclusion criteria included studies reporting pathogen-specific data with a sample size of at least 100 and studies with at least one year of follow-up. Studies merging certain pathogens and reporting aggregated outcomes were ineligible for inclusion in our analysis. We also excluded studies that exclusively reported on diarrheal outbreaks and studies that combined acute gastroenteritis with and without diarrhoea. We searched articles using a PubMed search term that combined non-specific and aetiology-specific diarrhoea using the following search string:

*(diarrhoea[title/abstract] OR diarrhea[title/abstract]) AND ( 2019/02/07:2020/12/31[PDat]) AND (incidence[title/abstract] OR prevalence[title/abstract] OR epidemiology[title/abstract] OR salmonella[title/abstract] OR aeromona*[title/abstract] OR shigell*[title/abstract] OR enteropathogenic[title/abstract] OR enterotoxigenic[title/abstract] OR campylobacter[title/abstract] OR amoebiasis[title/abstract] OR entamoeb*[title/abstract] OR cryptosporid*[title/abstract] OR rotavirus[title/abstract] OR norovirus[title/abstract] OR adenovirus[title/abstract] OR etiology[title/abstract]) NOT (appendicitis[title/abstract] OR esophag*[title/abstract] OR surger*[title/abstract] OR gastritis[title/abstract] OR liver[title/abstract] OR case report[title] OR case-report[title] OR therapy[title] OR treatment[title] Crohn[title/abstract] OR "inflammatory bowel"[title/abstract] OR irritable[title/abstract] OR travel*[title] OR Outbreak[title] OR Review[ptyp] OR vomiting[title/abstract) NOT (animals[MeSH] NOT humans[MeSH])*

We identified 924 studies, of which 60 met our inclusion criteria. We extracted data for location, sex, year, and age.

*Figure 1. Diarrhoeal disease aetiology systematic review PRISMA diagram*

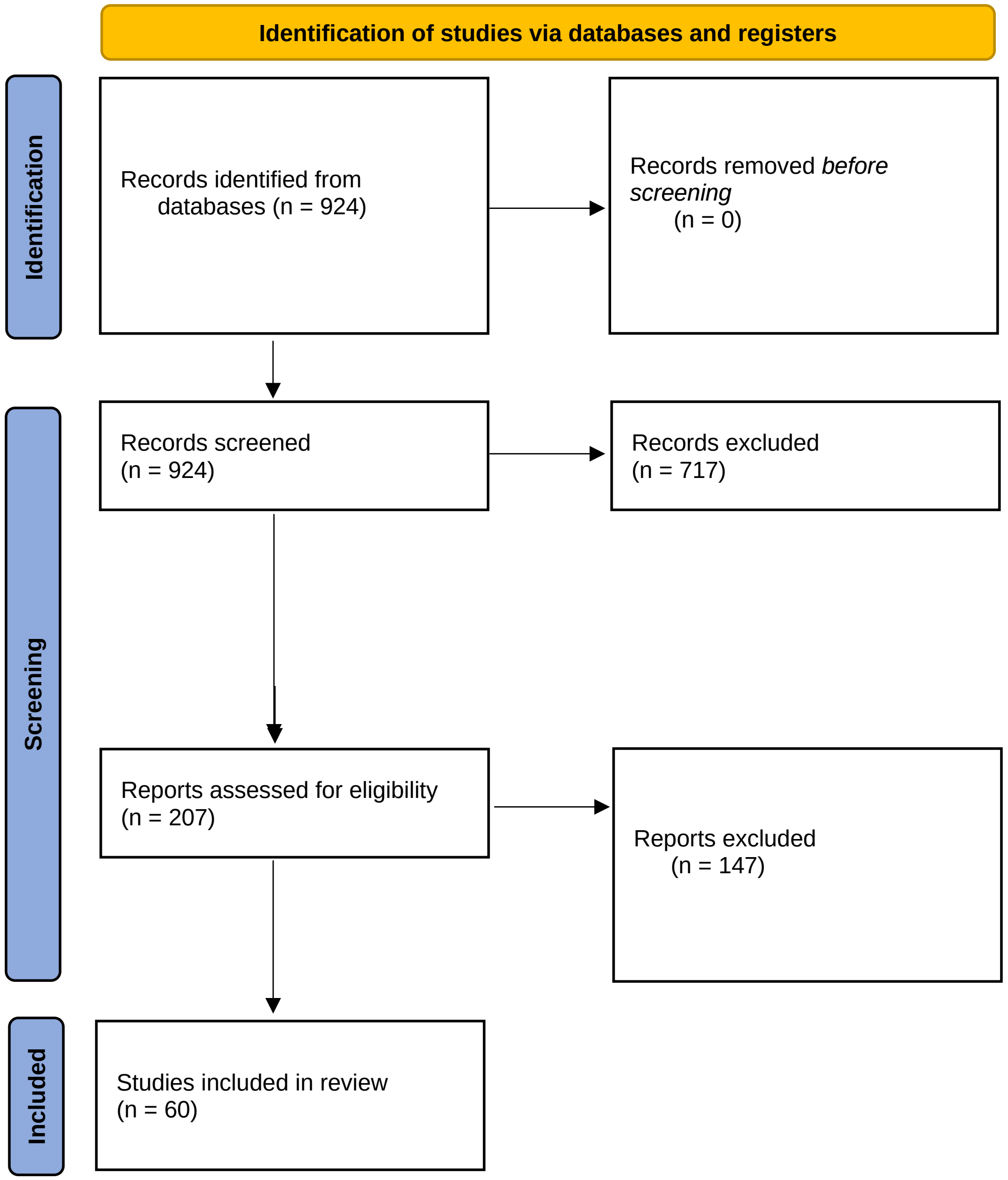

Additionally, we searched specifically for data sources detailing rotavirus coverage and vaccine efficacy using the following search string:

(((rotavirus[title/abstract] AND vaccine[ title/abstract] AND (efficacy[title/abstract] OR effectiveness[title/abstract]) AND (2019/02/07[PDAT] : 2020/12/31[PDAT])))) NOT Review[Publication Type] NOT (animals[MeSH] NOT humans[MeSH])

We identified 63 studies via PubMed and an additional 27 studies through manual reference search. Of the 90 studies identified, six met our inclusion criteria.

*Figure 2. Rotavirus vaccine efficacy systematic review PRISMA diagram*

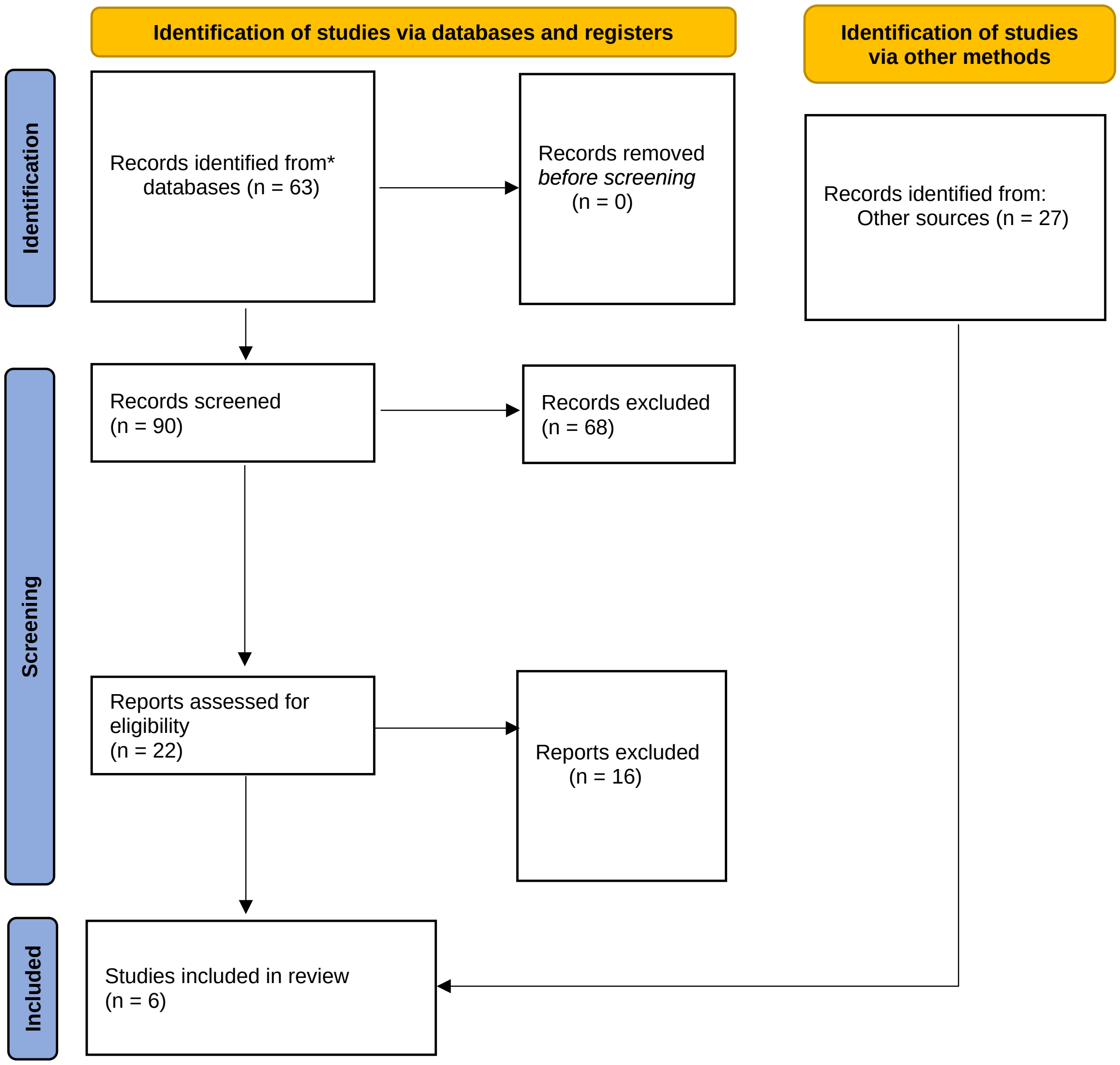


We used the Global Enteric Multicenter Study (GEMS), a seven-site, case-control study of moderate-to-severe diarrhoea in children under 5 years,[4] and the MAL-ED study,[5] a multi-site birth cohort, to calculate odds ratios

for the diarrhoeal pathogens. We analysed raw data for a systematic reanalysis, representative of the distribution of cases and controls by age and site that were tested for the presence of pathogen using quantitative polymerase chain reaction (qPCR).[6]

Data that did not use qPCR for detection were adjusted for sensitivity and specificity prior to modelling in order to standardise data regardless of detection method. Adjusting these data prior to modelling allowed us to adjust only data that did not use qPCR, as well as better control for values at extreme bounds and capture uncertainty in modelling.

Case fatality rate (CFR) data for *Clostridium difficile* were collected from ICD-coded hospital records from Austria, Brazil, Canada, Italy, Mexico, New Zealand, and the USA. ICD codes A04.7 (ICD-10) and 008.45 (ICD-9) were used to identify intestinal infections with *Clostridium difficile*. Supplemental data for Romania collected as part of the International Nosocomial Infection Control Consortium (DOI: 10.1016/j.ajic.2016.01.005) were also included. We standardised age and sex across all datasets to the following most-detailed groups using the GBD causes of death age-sex splitting algorithm for age: 0–6, 7–27, and 28–364 days, and 1–4, 5–9, 10–14, 15–19, 20–24, 25–29, 30–34, 35–39, 40–44, 45–49, 50–54, 55–59, 60–64, 65–69, 70–74, 75–79, 80–84, 85–89, 90–94, 95+ years; and sex: male and female. This algorithm is based on the assumption that the age-sex pattern of the death or case rate for a given infectious syndrome or pathogen is inherent to the pathology of the disease and is therefore constant across location and year. Crude case-fatality rates were then estimated for the input data for each GBD age group and sex.

We estimated diarrhoeal disease aetiologies independently from overall diarrhoea envelope using a counterfactual strategy for enteric adenovirus, *Aeromonas*, *Entamoeba histolytica* (amoebiasis), *Campylobacter*, *Cryptosporidium*, enteropathogenic *Escherichia coli* (EPEC), enterotoxigenic *Escherichia coli* (ETEC), norovirus, non-typhoidal *Salmonella* infections, rotavirus, and *Shigella*. *Vibrio cholerae* and *C. difficile* were modelled separately.

Diarrhoeal aetiologies are attributed to diarrhoeal cases and deaths using a counterfactual approach. We calculated a population attributable fraction (PAF) from the proportion of diarrhoea cases that are positive for each aetiology. The PAF represents the relative reduction in diarrhoea burden if there was no exposure to a given aetiology. As diarrhoea can be caused by multiple pathogens and the pathogens may co-infect, PAFs can overlap and are not scaled to sum to 100%. We calculated the non-fatal PAF from the proportion of diarrhoea cases that are positive for each aetiology. We calculated the fatal PAF from the proportion of severe diarrhoea cases that are positive for each aetiology. We assumed that hospitalised diarrhoea cases are a proxy of severe and fatal cases. We used the following formula to estimate PAF:[5]

$$PAF = Proportion * (1 - \frac{1}{OR})$$

Where *Proportion* is the proportion of diarrhoea cases positive for an aetiology and *OR* is the odds ratio of diarrhoea given the presence of the pathogen.

We dichotomised the continuous qPCR test result using the value of the cycle threshold (Ct) that most accurately discriminated between cases and controls. The Ct values range from 0 to 35 cycles representing the relative concentration of the target gene in the stool sample. A low value indicates a higher concentration of the pathogen, while a value of 35 indicates the absence of the target in the sample. We used the lower Ct

value when we had multiple Ct values for the cut-point. The case definition for each pathogen is a Ct value that is below the established cutoff point.

In non-fatal modeling, we used a generalised linear mixed effects logistic regression model to calculate the odds ratio for under 1 year and 1–2 years old for each of our pathogens from the MAL-ED study. The MAL-ED study was used exclusively because the samples tested from that study are community-based samples, which we determined were more representative of non-fatal diarrhoea than the GEMS samples, which tested only moderate-to-severe diarrhoea. The odds ratio for 1–2 years was applied to all GBD age groups over 5 years. There were three pathogen-age odds ratios that were not statistically significant: *Aeromonas* and amoebiasis in under 1 year and *Campylobacter* in 1–2 years.

For fatal modeling, we used a mixed effects conditional logistic regression model to calculate the odds ratio for ages under 1 year and 1–4 years old for each of our pathogens. The stool samples from cases and controls in GEMS were used exclusively to calculate these odds ratios as we assumed that the association between pathogens and moderate-to-severe diarrhoea is a proxy for fatal outcomes. The odds ratio for ages 1–4 years was applied to all GBD age groups over 5 years. There were three pathogen-age odds ratios that were not statistically significant: *Aeromonas* and amoebiasis in under 1 year and *Campylobacter* in 1–4 years.

If the odds ratio was not statistically significant, we transformed the odds ratios only for those aetiologies in log-space such that exponentiated values could not be below 1. The transformation was:

$$Odds\ ratio = exp(log(OR) - 1)) + 1$$

We modelled the proportion data using the Bayesian meta-regression tool DisMod-MR to estimate the proportion of positive diarrhoea cases for each separate aetiology by location/year/age/sex and to adjust for the covariates. To account for differences between studies, we also conducted a meta-regression of the ratio of reference to non-reference data using the meta-regression—Bayesian, regularised, trimmed (MR-BRT) tool. We adjusted inpatient data (Table 5) and single-pathogen data (Table 6).

Table 5. Inpatient to community crosswalk coefficients for diarrhoeal disease aetiologies, not including *Vibrio cholerae* or *C. difficile*

| Aetiology | Data input | Reference or alternative case definition | Gamma | Crosswalk covariate | Beta coefficient, logit (95% UI) |
|---|---|---|---|---|---|
| All | community-based samples | ref | -- | -- | -- |
| Adenovirus | Clinical, inpatient | alt | 0.09 | intercept | 0.25 (0.08 to 0.43) |
| *Aeromonas* | Clinical, inpatient | alt | 0.14 | intercept | 0.19 (–0.09 to 0.46) |
| Amoebiasis | Clinical, inpatient | alt | 0.80 | intercept | 0.12 (–0.47 to 0.72) |

| | | | | | |
|---|---|---|---|---|---|
| *Campylobacter* | Clinical, inpatient | alt | 0.26 | intercept | –0.11 (–0.33 to 0.12) |
| *Cryptosporidium* | Clinical, inpatient | alt | 0.05 | intercept | 0.24 (0.11 to 0.38) |
| EPEC | Clinical, inpatient | alt | 0.02 | intercept | 0.03 (–0.10 to 0.16) |
| ETEC | Clinical, inpatient | alt | 0.00 | intercept | 0.10 (-0.003 to 0.21) |
| Norovirus | Clinical, inpatient | alt | 0.04 | intercept | 0.06 (–0.06 to 0.18) |
| Rotavirus | Clinical, inpatient | alt | 0.35 | intercept | 0.70 (0.53 to 0.87) |
| *Salmonella* | Clinical, inpatient | alt | 0.15 | intercept | 0.47 (0.19 to 0.74) |
| Shigellosis | Clinical, inpatient | alt | 0.39 | intercept | 0.29 (0.02 to 0.57) |

Table 6. Single to multi-pathogen study crosswalk coefficients for diarrhoeal disease aetiologies, not including *Vibrio cholerae* or *C. difficile*

| Aetiology | Data input | Reference or alternative case definition | Gamma | Crosswalk covariate | Beta coefficient, logit (95% UI) |
|---|---|---|---|---|---|
| All | Multi-pathogen studies | ref | -- | -- | -- |
| Adenovirus | Single pathogen | alt | 0.00 | intercept | 1.06 (0.89 to 1.22) |
| *Aeromonas* | Single pathogen | alt | N/A | intercept | N/A |
| Amoebiasis | Single pathogen | alt | N/A | intercept | N/A |
| *Campylobacter* | Single pathogen | alt | N/A | intercept | N/A |

| | | | | | |
|---|---|---|---|---|---|
| *Cryptosporidium* | Single pathogen | alt | N/A | intercept | N/A |
| EPEC | Single pathogen | alt | N/A | intercept | N/A |
| ETEC | Single pathogen | alt | 0.00 | intercept | 0.14 (–0.03 to 0.31) |
| Norovirus | Single pathogen | alt | N/A | intercept | N/A |
| Rotavirus | Single pathogen | alt | 0.43 | intercept | 0.53 (0.23 to 0.84) |
| *Salmonella* | Single pathogen | alt | 0.00 | intercept | 0.78 (0.50 to 1.07) |
| Shigellosis | Single pathogen | alt | 0.00 | intercept | 0.72 (0.62 to 0.82) |

We used the estimated sensitivity and specificity of the original laboratory diagnostic test results from the pooled GEMS and MAL-ED qPCR stool samples compared to the qPCR test result to adjust our proportion before we modelled the proportions:[6]

$$Proportion_{True} = \frac{(Proportion_{Observed} + Specificity - 1)}{(Sensitivity + Specificity - 1)}$$

We used this correction to account for the fact that the proportions we used are based on a new test that is not consistent with the laboratory-based case definition (qPCR versus GEMS conventional laboratory testing for pathogens).[6] Because of differences in the type of PCR used in the original (non-reference qPCR diagnostic) between GEMS and MAL-ED in detecting norovirus, we combined the sensitivity and specificity results for norovirus such that 50% of the draws were coming from GEMS test results exclusively, and 50% of the draws were coming from MAL-ED test results exclusively. Additionally, because the original laboratory diagnostic technique used for *Campylobacter* in MAL-ED was one not commonly used, we only used GEMS to determine the sensitivity and specificity of bacterial culture compared to qPCR in detecting *Campylobacter*.[8]

In order to be consistent with the odds ratios that we obtained, we adjusted our proportion estimates of any EPEC to typical EPEC only. This adjustment was informed by all available data that reported both atypical and typical EPEC. We applied the same approach to differentiate between heat-stable toxin (ST) and heat-labile toxin-producing (LT) ETEC. This was based on work showing that ST-ETEC was much more pathogenic than LT-ETEC. As some of our proportion data were extracted for any ETEC, we determined a proportion of all ETEC that produced ST from all available data reporting both ETEC and ST-ETEC and applied that ratio to data representing all ETEC so that they represented ST-ETEC only.

For *Vibrio cholerae* (cholera), we used the literature review to estimate the expected number of cholera cases for each country-year using the incidence of diarrhoea (estimated using DisMod-MR) and the proportion of diarrhoea cases that are positive for cholera. We assigned cholera PAF using odds ratios from the qPCR results to estimate a number of cholera-attributable cases. We compared this expected number of cholera cases to the number reported to WHO at the country-year level.[11] We modelled the under-reporting fraction to correct the cholera case notification data for all countries using health system access and the diarrhoea SEV scalar to predict total cholera cases. We used the age-specific proportion of positive cholera samples in DisMod-MR and our incidence estimates to predict the number of cholera cases for each age/sex/year/location. Finally, we modelled the case fatality ratio of cholera using DisMod-MR to estimate the number of cholera deaths.

For *C. difficile*, we modelled incidence data identified via systematic review and excess mortality estimates in DisMod-MR 2.1. DisMod-MR uses a compartmental model to relate prevalence, incidence, remission, and mortality. We set remission in our model to 1 month. Excess mortality rates (EMRs) were computed based on case-fatality rates by age from hospital data and an assumed duration (1.0 month [0.3–1.7]) using the following equation:

$$EMR = -\ln(1 - CFR)/duration$$

*C. difficile* CFRs were calculated using MR-BRT, a meta-analytic mixed effects structure. The main model can be specified as follows:

$$logit(y_i) = X_i\beta + u_i 1 + \epsilon_i, \qquad \epsilon_i \sim N(0, \Sigma_i), \quad u_i \sim N(0, \gamma)$$

where

- $y_i$ contains CFRs for data source $i$
- Design matrix $X_i$ contains as columns the following covariates
    - HAQ Index
    - dummy-coded indicator for age group
        - neonatal–5 years, 5–50 years, 50–70 years, and 70 years and older
    - dummy-coded ICU indicator for data source
        - 1 if data source only compiles information on ICU patients, 0 if a mix between ICU/non-ICU patients
    - dummy-coded indicator for pathogen
- $\beta$ are fixed effect multipliers
- $\epsilon_i$ are observation error terms with known variances
- $u_i$ are data source-specific random intercepts with unknown covariance $\gamma$

We also implemented a prior on $\gamma$, the data source random effect. Many input data sources cover only a single country, leading to low variability in HAQ Index within each data source. Such collinearity adversely influenced the accuracy of the estimated effect of HAQ Index, which was instrumental in extrapolating trends from the input data to global results. To emphasise the contribution of HAQ Index over data source in the modelled estimates, we implemented a strong Gaussian prior (mean 0, standard error 0.001) on $\gamma$. Predictions for *Clostridium difficile* CFRs were generated for each country and age group as a function of each country's HAQ Index, assuming mixed ICU/non-ICU patients.

For rotavirus, we explicitly accounted for rotavirus vaccine efficacy when estimating attributable fraction, as in GBD 2019. The impact of the rotavirus vaccine is dependent on modelled vaccine coverage for a location-year and on the rotavirus vaccine efficacy (VE). Numerous studies demonstrate a difference in VE by national income and development.[12] We also determined via LASSO (least absolute shrinkage and selection operator) that Socio-demographic Index (SDI) was the best predictor of rotavirus VE. We used a meta-regression with SDI as covariate to predict the rotavirus VE by location and year.

Starting from GBD 2019, we explicitly incorporated the results from our analysis of VE to produce more robust estimates of the proportion of diarrhoea that has rotavirus over time and space. We assumed that the impact of the vaccine can be represented as 1 minus the product of the estimated vaccine coverage and VE.

$$Vaccine\ impact = 1 - vaccine\ coverage * vaccine\ efficacy$$

Both of these values vary in time and space but not by age. To avoid discontinuities in our DisMod model, we adjusted the input proportion data to remove the impact of the rotavirus vaccine by dividing the observed proportion by the vaccine impact.

$$Rotavirus\ proportion_{Adjusted} = \frac{Rotavirus\ proportion}{1 - Cov_{RotaV} * VE_{Modeled}}$$

The result from DisMod is the modelled proportion of diarrhoea positive for rotavirus in the absence of the vaccine. This modelled value is then multiplied by the impact of the rotavirus vaccine to determine the estimated proportion of diarrhoea positive for rotavirus in the presence of the vaccine. Our modified attributable fraction is then:

$$DisModPAF = Modeled\ Proportion\ (from\ DisMod)\ * \left(1 - \frac{1}{OR}\right)$$

The last step is to account for the expected impact of the rotavirus vaccine. We do this using the equation below:

$$PAF_{Rota} = DisModPAF * \frac{(1 - Cov_{RotaV} * VE_{Modeled})}{(1 - DisModPAF * Cov_{RotaV} * VE_{Modeled})}$$

Where the final attributable fraction for rotavirus is the product of the PAF estimated in DisMod-MR and the expected reduction in that PAF given modelled vaccine coverage and modelled VE by location-year, and this value is only applied to children 28 days to 5 years old. The product of the rotavirus attributable fraction and the number of deaths or cases of diarrhoea is the number of deaths and cases caused by rotavirus.

## Diarrhoeal references


1. Naghavi M, Ong KL, Aali A, Ababneh HS, Abate YH, Abbafati C, et al. Global burden of 288 causes of death and life expectancy decomposition in 204 countries and territories and 811 subnational locations, 1990–2021: a systematic analysis for the Global Burden of Disease Study 2021. The Lancet. 2024 May 18;403(10440):2100–32.

2. GBD 2021 Diseases and Injuries Collaborators. Global incidence, prevalence, years lived with disability (YLDs), disability-adjusted life-years (DALYs), and healthy life expectancy (HALE) for 371 diseases and

injuries in 204 countries and territories and 811 subnational locations, 1990-2021: a systematic analysis for the Global Burden of Disease Study 2021. Lancet Lond Engl. 2024 May 18;403(10440):2133–61.

3. Brauer M, Roth GA, Aravkin AY, Zheng P, Abate KH, Abate YH, et al. Global burden and strength of evidence for 88 risk factors in 204 countries and 811 subnational locations, 1990–2021: a systematic analysis for the Global Burden of Disease Study 2021. The Lancet. 2024 May 18;403(10440):2162–203.

4. Kotloff KL, Nataro JP, Blackwelder WC, Nasrin D, Farag TH, Panchalingam S, et al. Burden and aetiology of diarrhoeal disease in infants and young children in developing countries (the Global Enteric Multicenter Study, GEMS): a prospective, case-control study. Lancet Lond Engl. 2013 Jul 20;382(9888):209–22.

5. Platts-Mills J, Liu J, Rogawski E. Aetiology, burden and clinical characteristics of diarrhoea in children in low-resource settings using quantitative molecular diagnostics: results from the MAL-ED cohort study. Lancet Glob Health. 2018;Accepted.

6. Liu J, Gratz J, Amour C, Kibiki G, Becker S, Janaki L, et al. A laboratory-developed TaqMan Array Card for simultaneous detection of 19 enteropathogens. J Clin Microbiol. 2013 Feb;51(2):472–80.

7. Foreman KJ, Lozano R, Lopez AD, Murray CJ. Modeling causes of death: an integrated approach using CODEm. Popul Health Metr. 2012 Jan 6;10(1):1.

8. Reiczigel J, Földi J, Ozsvári L. Exact confidence limits for prevalence of a disease with an imperfect diagnostic test. Epidemiol Infect. 2010 Nov;138(11):1674–8.

9. Platts-Mills JA, Operario DJ, Houpt ER. Molecular diagnosis of diarrhea: current status and future potential. Curr Infect Dis Rep. 2012 Feb;14(1):41–6.

10. Platts-Mills JA, Liu J, Gratz J, Mduma E, Amour C, Swai N, et al. Detection of Campylobacter in stool and determination of significance by culture, enzyme immunoassay, and PCR in developing countries. J Clin Microbiol. 2014 Apr;52(4):1074–80.

11. World Health Organization. Global Health Observatory data repository: Cholera [Internet]. World Health Organization; 2016 [cited 2016 Aug 25]. Available from: http://apps.who.int/gho/data/node.main.174?lang=en

12. Lamberti LM, Ashraf S, Walker CLF, Black RE. A Systematic Review of the Effect of Rotavirus Vaccination on Diarrhea Outcomes Among Children Younger Than 5 Years. Pediatr Infect Dis J. 2016 Sep;35(9):992–8.

13. Lamberti LM, Fischer Walker CL, Black RE. Systematic review of diarrhea duration and severity in children and adults in low- and middle-income countries. BMC Public Health. 2012;12:276.

# Unsafe water

## Flowchart

Unsafe Drinking Water

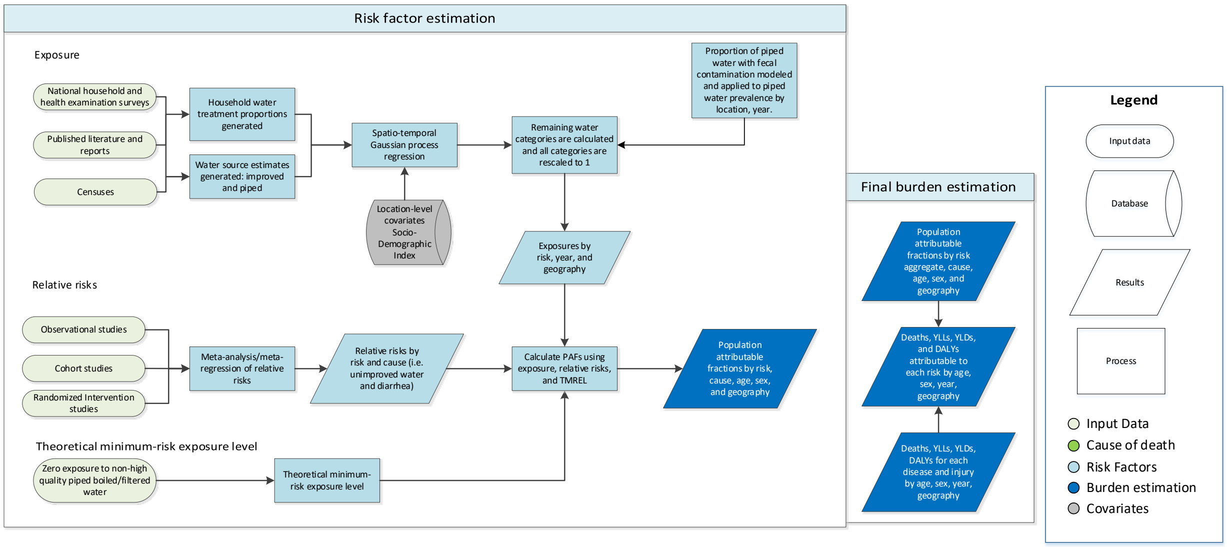


## Input data and methodological summary

## Exposure

### *Case definition*

For the Global Burden of Diseases, Injuries, and Risk Factors Study (GBD) 2021, exposure to unsafe water was defined based on (1) reported primary water source used by the household, and (2) use of household water treatment (HWT) to improve the quality of drinking water before consumption. Water sources were defined based on the WHO/UNICEF Joint Monitoring Programme for Water Supply, Sanitation and Hygiene (JMP).[1] Examples of "improved" sources include boreholes, tube wells, protected wells, and packaged or delivered water. Piped water is also considered "improved" by the JMP but is placed into its own category for GBD purposes. Examples of "unimproved" sources include unprotected springs, unprotected wells, and surface water. Additionally, four different HWTs were determined to be effective point-of-use treatments based on effect sizes calculated from a network meta-analysis: solar treatment, chlorine treatment, boiling, and filtering. For modelling purposes, we grouped solar and chlorine treatment together, as well as boiling and filtering.

### *Input data*

The search for usable data sources was conducted using the Global Health Data Exchange (GHDx) database. Water source input data came primarily from nationally representative surveys, such as the Demographic and Health Survey (DHS), the Multiple Indicator Cluster Surveys (MICS), the World Health Survey (WHS), and the DHS AIDS Indicator Survey (AIS). HWT input data were largely limited to the DHS and MICS due to data availability. Surveys that reported results at the household level were converted to the individual level using household size data to ensure that our models estimated the proportion of individuals, rather than households, exposed to a given indicator. For GBD 2021, we re-extracted nearly all of our sources from 2000 to present in an effort to standardise extraction outputs and fix past extraction errors. Additionally, we added 64 new sources for this cycle. After extraction, surveys and censuses were then tabulated to the water source

and water treatment categories of interest for each location. Table 1 provides a summary of the exposure input data.

Table 1: Exposure input data

| Input data | Exposure |
|---|---|
| Source count (total) | 1221 |
| Number of countries with data | 170 |

### *Modelling strategy*

Water source data were modelled using an ordinal framework, with two distinct models: (1) proportion of the total population that uses piped water sources, and (2) proportion of the non-piped population that uses improved water sources. Both models were estimated for all ages and both sexes combined, and produced results for each unique location-year combination. This ordinal framework allowed estimating the category with the most data (piped water prevalence) and leveraging that estimate to anchor the estimates for the improved and unimproved water categories. The results of the improved proportion model were multiplied by 1 minus the piped water prevalence to calculate improved water prevalence. The sum of improved and piped water prevalence was then subtracted from 1 to yield unimproved water prevalence.

$$\boldsymbol{Piped} = \frac{\#\ persons\ using\ piped\ water}{\#\ persons\ with\ nonmissing\ response}$$

$$\boldsymbol{Improved} = \left(\frac{\#\ persons\ using\ improved\ water}{\#\ persons\ without\ piped\ water}\right) * (1 - \boldsymbol{Piped})$$

$$\boldsymbol{Unimproved} = 1 - (\boldsymbol{Piped} + \boldsymbol{Improved})$$

HWT categories were estimated in a similar ordinal framework. Its two models were (1) proportion of the total population that does not use any water treatment methods, and (2) proportion of the population that treat their water that use boiling or filtering as their primary HWT. Like the water source models, both HWT models were estimated for all ages and both sexes combined and produced results for each unique location-year combination. The proportion of individuals who boil/filter drinking water was calculated by multiplying the proportion who boil/filter modelled previously multiplied by the prevalence of any water treatment (estimated by subtracting the prevalence of no treatment from 1). The proportion of individuals who treat their water using solar/chlorine methods was estimated by subtracting from 1 the sum of prevalence of no treatment estimates and prevalence of filter/boil treatment.

$$\boldsymbol{No\ HWT} = \frac{\#\ persons\ who\ do\ not\ treat\ water}{\#\ persons\ with\ nonmissing\ response}$$

$$\boldsymbol{Boil\ or\ filter} = \left(\frac{\#\ persons\ who\ treat\ water\ with\ boil\ or\ filter}{\#\ persons\ who\ treat\ water\ with\ any\ method}\right) * (1 - \boldsymbol{No\ HWT})$$

$$\boldsymbol{Chlorine\ or\ solar} = 1 - (\boldsymbol{No\ HWT} + \boldsymbol{Boil\ or\ filter})$$

Additionally, we modelled the microbiological quality of piped water sources primarily using data from a review by Bain et al 2014[2] that measured the proportion of piped water sources contaminated with faecal matter. We used the results from this model to split the prevalence of piped water into basic piped water and

high-quality piped water by location and year. High-quality piped water is piped water that enters the household free of contamination. Thus, HWT is irrelevant for this category, since treatment is only necessary if the water is contaminated.

$$\boldsymbol{Faecal\ contamination} = \frac{\#\ contaminated\ piped\ water\ systems}{\#\ piped\ water\ systems\ sampled}$$

$$\boldsymbol{Basic\ piped = Piped * Faecal\ contamination}$$

$$\boldsymbol{HQ\ piped = Piped - Basic\ piped}$$

Each of the models described above was modelled using a three-step modelling scheme of mixed-effect linear regression followed by spatiotemporal Gaussian process regression (ST-GPR), which produced full time-series estimates for each GBD 2021 location. Socio-demographic Index (SDI), a composite measure of development combining education per capita, income per capita, and fertility, was set as a fixed effect in the linear regression since it proved to be a significant predictor. The proportion of individuals with access to piped water was also used as a covariate in the faecal matter model. Random effects were set at GBD 2021 region and super-region levels to fit the models but were not used in the predictions. The linear regression equations for each of the five ST-GPR models used for this risk factor are listed below.

**Proportion using piped water:** $logit(data) \sim SDI + (1|level\_1) + (1|level\_2)$

**Proportion of non-piped population using improved water:** $logit(data) \sim SDI + (1|level\_1) + (1|level\_2)$

**Proportion using no HWT:** $logit(data) \sim SDI + (1|level\_1) + (1|level\_2)$

**Proportion of HWT-using population that boils/filters:** $logit(data) \sim SDI + (1|level\_1) + (1|level\_2)$

**Proportion of piped water systems contaminated with faecal matter:** $logit(data) \sim SDI + piped\ water\ access + (1|level\_1) + (1|level\_2)$

*Piped water access = proportion of individuals with access to piped water*
*(1|level_1) = super-region-level random effects*
*(2|level_2) = region-level random effects*

The process of vetting and validating models was accomplished primarily through an examination of ST-GPR scatter plots by GBD 2021 location from 1990 to 2021. Any poorly fitting datapoints were re-inspected for error at the level of extraction and survey implementation. If errors in data extraction were found, the study in question was re-extracted. In addition to SDI, a number of different potential fixed effects were considered, including lag-distributed income and urbanicity, but SDI proved to be the strongest predictor of the unsafe water categories. Uncertainty in the estimates was initially formed based on standard deviation by survey, then propagated through ST-GPR modelling by means of confidence intervals around each datapoint that reflected the point-estimate-specific variance. A datapoint with high variance, for example, would contribute relatively less influence to the model than a datapoint with lower variance.

Once models were vetted, full time-series outputs from ST-GPR modelling were then rescaled to form ten mutually exclusive categories that summed to 1 for each location-year combination. Table 2 provides the final result of this rescaling and also includes the formulas for each category.

## Table 2: Exposure categories, definitions, and formulas

| Exposure category | Definition | Formula |
|---|---|---|
| Unimproved, no HWT | Proportion of individuals who primarily use unimproved source and *do not* use any HWT to purify their drinking water. | $[1-(Piped+Improved)] * \left[\frac{\#\ persons\ who\ do\ not\ treat\ water}{\#\ persons\ with\ nonmissing\ response}\right]$ |
| Unimproved, chlorine/solar | Proportion of individuals who primarily use unimproved source, and who use solar or chlorine treatment to purify their drinking water. | $[1-(Piped+Improved)] * [1-(No\ HWT+Boil\ or\ filter)]$ |
| Unimproved, boil/filter | Proportion of individuals who primarily use unimproved source and who boil or filter to purify their drinking water. | $[1-(Piped+Improved)] * \left[\left(\frac{\#\ persons\ who\ treat\ water\ with\ boil\ or\ filter}{\#\ persons\ who\ treat\ water\ with\ any\ method}\right) * (1-No\ HWT)\right]$ |
| Improved water except piped, no HWT | Proportion of individuals who primarily use improved sources other than piped water supply and *do not* use any HWT to purify their drinking water. | $\left[\left(\frac{\#\ persons\ using\ improved\ water}{\#\ persons\ without\ piped\ water}\right)\right] * \left[\frac{\#\ persons\ who\ do\ not\ treat\ water}{\#\ persons\ with\ nonmissing\ response}\right]$ |
| Improved water except piped, chlorine/solar | Proportion of individuals who primarily use improved sources other than piped water supply, and who use solar or chlorine treatment to purify their drinking water. | $\left[\left(\frac{\#\ persons\ using\ improved\ water}{\#\ persons\ without\ piped\ water}\right)\right] * [1-(No\ HWT+Boil\ or\ filter)]$ |
| Improved water except piped, boil/filter | Proportion of individuals who primarily use improved sources other than piped water supply and who boil/filter their drinking water. | $\left[\left(\frac{\#\ persons\ using\ improved\ water}{\#\ persons\ without\ piped\ water}\right)\right] * \left[\left(\frac{\#\ persons\ who\ treat\ water\ with\ boil\ or\ filter}{\#\ persons\ who\ treat\ water\ with\ any\ method}\right) * (1-No\ HWT)\right]$ |
| Basic piped water, no HWT | Proportion of individuals who primarily use basic piped water supply and *do not* use any HWT to purify their drinking water. | $\left[\left[\frac{\#\ persons\ using\ piped\ water}{\#\ persons\ with\ nonmissing\ response}\right] * \left[\frac{\#\ contaminated\ piped\ water\ systems}{\#\ piped\ water\ systems\ sampled}\right]\right] * \left[\frac{\#\ persons\ who\ do\ not\ treat\ water}{\#\ persons\ with\ nonmissing\ response}\right]$ |
| Basic piped water, chlorine/solar | Proportion of individuals who primarily use basic piped water supply, and who *use* solar or chlorine water treatment, to purify their drinking water. | $\left[\left[\frac{\#\ persons\ using\ piped\ water}{\#\ persons\ with\ nonmissing\ response}\right] * \left[\frac{\#\ contaminated\ piped\ water\ systems}{\#\ piped\ water\ systems\ sampled}\right]\right] * [1-(No\ HWT+Boil\ or\ filter)]$ |
| Basic piped water, boil/filter | Proportion of individuals who primarily use basic piped water supply and who boil or filter to purify their drinking water. | $\left[\left[\frac{\#\ persons\ using\ piped\ water}{\#\ persons\ with\ nonmissing\ response}\right] * \left[\frac{\#\ contaminated\ piped\ water\ systems}{\#\ piped\ water\ systems\ sampled}\right]\right] * \left[\left(\frac{\#\ persons\ who\ treat\ water\ with\ boil\ or\ filter}{\#\ persons\ who\ treat\ water\ with\ any\ method}\right) * (1-No\ HWT)\right]$ |
| High-quality piped water | Proportion of individuals who primarily use high-quality piped water. | $\left[\frac{\#\ persons\ using\ piped\ water}{\#\ persons\ with\ nonmissing\ response}\right] - \left[\left[\frac{\#\ persons\ using\ piped\ water}{\#\ persons\ with\ nonmissing\ response}\right] * \left[\frac{\#\ contaminated\ piped\ water\ systems}{\#\ piped\ water\ systems\ sampled}\right]\right]$ |

## Theoretical minimum-risk exposure level

The theoretical minimum-risk exposure level for unsafe water is defined as having access to high-quality piped water.

## Relative risks

### *Input data*

For GBD 2021, unsafe water was paired with one outcome – diarrhoeal diseases – given evidence provided by relative risk studies. Input data included in the GBD 2021 unsafe water relative risk analysis are as follows:

Table 3: Relative risk input data

| Input data | Relative risk |
|---|---|
| Source count (total) | 73 |
| Number of countries with data | 36 |

Two meta-analyses (Wolf et al 2014 and Wolf et al 2018) were used to identify relative risk studies, including years 1970-2016.[3,4] Additionally, a literature review on the relationship between high-quality piped water and diarrhoea was conducted for GBD 2021, which yielded three new studies (Figure 1). We searched PubMed for relevant literature published from January 1, 1970 to July 2, 2020 (date of search), using the search string below:

("Drinking Water"[Mesh] OR "Water Quality"[Mesh] OR "Water Supply"[Mesh] OR "Piped water"[TIAB] OR "Tap water"[TIAB] OR "Potable water"[TIAB]) AND ("Diarrhea"[Mesh] OR Diarrh*[TIAB] OR "Diarrhea incidence"[TIAB] OR "Bacteriological"[TIAB] OR "Microbial water quality"[TIAB]) AND (1970[PDAT] : 3000[PDAT) NOT (animals[MeSH] NOT humans[MeSH])

*Figure 1: PRISMA diagram for systematic review of high-quality piped water and diarrheal disease*

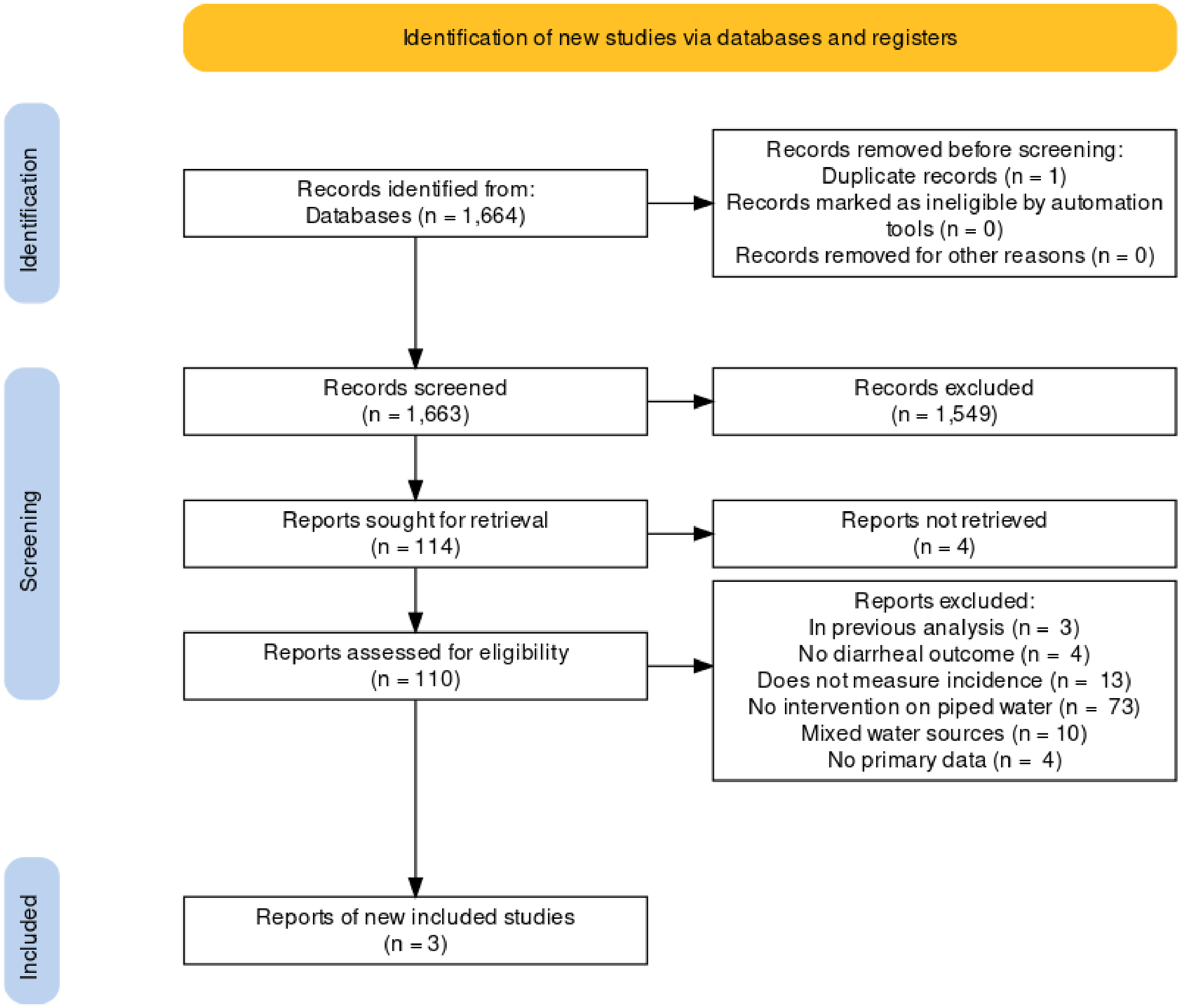


## *Modelling strategy*

In GBD 2021, relative risk values were calculated using a network meta-analysis approach with a tool called meta-regression—Bayesian, regularised, trimmed (MR-BRT). One study-level covariate – whether conformity with the study interventions was self-reported or confirmed by the researchers – was included in the network meta-analysis. Several other covariates (whether exposure was captured at the individual level or population level; whether or not the study was randomised; whether or not the study adjusted for all major known confounders; and what the study's follow-up percentage was) were considered but ultimately were not statistically significant and so were not included in the analysis. No priors were used. The risk of developing diarrhoea relative to using an unimproved water source was calculated for each of the following categories: boil or filter, solar or chlorine, improved, piped, and high-quality piped (Table 4). These model results were then combined and rescaled to match with our exposure definitions (Table 5). The combined effects of source interventions (ie, improved, piped, high-quality piped) and point-of-use interventions (ie, boil/filter, solar/chlorine) were assumed to be multiplicative. Additionally, we assumed that the lowest possible risk level is using the best source type (high-quality piped water) combined with the best point-of-use treatment (boil/filter).

*Table 4: MR-BRT network meta-analysis results (reference: unimproved water source)*

| Intervention | Relative risk (95% UI) |
|---|---|
| Boil/filter water treatment | 0.57 (0.37–0.87) |
| Chlorine/solar water treatment | 0.77 (0.51–1.15) |
| Improved water source | 0.84 (0.54–1.28) |
| Piped water source | 0.67 (0.44–1.03) |
| High-quality piped water source | 0.29 (0.12–0.70) |

*Table 5: Relative risks for each exposure category (reference: high-quality piped water)*

| Exposure category | Relative risk (95% UI) |
|---|---|
| Unimproved, no HWT | 6.93 (2.20–17.54) |
| Unimproved, chlorine/solar | 5.41 (1.59–14.02) |
| Unimproved, boil/filter | 3.87 (1.42–8.66) |
| Improved water except piped, no HWT | 5.97 (1.77–16.16) |
| Improved water except piped, chlorine/solar | 4.67 (1.25–13.20) |
| Improved water except piped, boil/filter | 3.33 (1.13–8.20) |
| Basic piped water, no HWT | 4.73 (1.33–12.59) |
| Basic piped water, chlorine/solar | 3.69 (1.01–10.14) |
| Basic piped water, boil/filter | 2.64 (0.86–6.34) |
| High-quality piped water | 1 (reference) |

Figure 2 shows the results of the MR-BRT analysis in graphical form, along with the associated "risk-outcome scores" for each category, which is a measure of how good the evidence is for that particular relative risk estimate. Prior to generating an risk-outcome score, we conducted an additional post-analysis step to detect and flag publication bias in the input data. This approach is based on the classic Egger's regression strategy, which is applied to the residuals in our model. In the current implementation, we do not correct for publication bias, but flag the risk–outcome pairs where the risk for publication bias is significant.

We did not detect publication bias based on the association between observation residuals and their standard errors (p-value = 0.195, Egger mean = –0.101, Egger SD = 0.118). The overall risk-outcome score for this risk factor is 0.231, which is the maximum of the individual category scores.

*Figure 2: Risk-outcome scores*

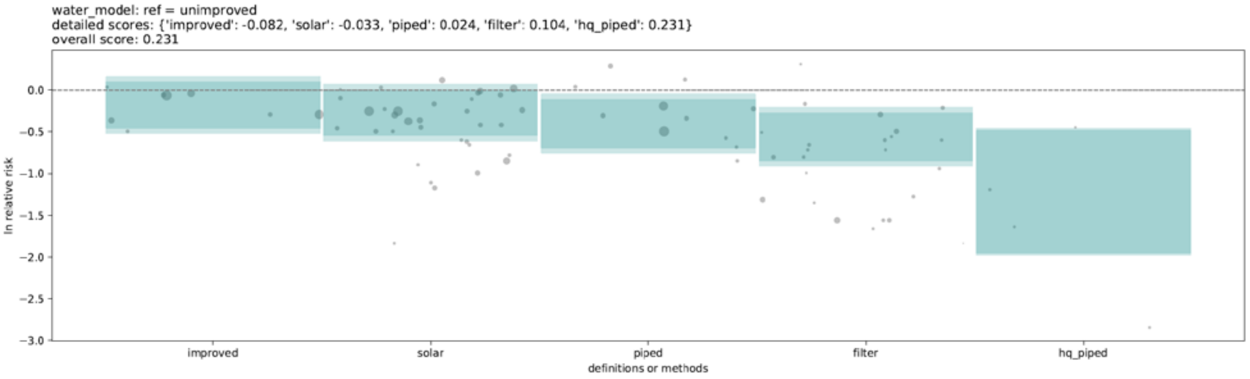


## Unsafe Water References


1. WHO/UNICEF Joint Monitoring Programme: Drinking water. https://washdata.org/monitoring/drinking-water (accessed Oct 31, 2019).
2. Bain R, Cronk R, Wright J, Yang H, Slaymaker T, Bartram J. Fecal Contamination of Drinking-Water in Low- and Middle-Income Countries: A Systematic Review and Meta-Analysis. *PLOS Medicine* 2014; **11**: e1001644.
3. Wolf J, Pruss-Ustun A, Cumming O, *et al.* Assessing the impact of drinking water and sanitation on diarrhoeal disease in low- and middle-income settings: systematic review and meta-regression. *Tropical Medicine and International Health* 2014; **19**: 928–42.
4. Wolf J, Hunter PR, Freeman MC, *et al.* Impact of drinking water, sanitation and handwashing with soap on childhood diarrhoeal disease: updated meta-analysis and meta-regression. *Tropical Medicine and International Health* 2018; **23**: 508–25.


# Unsafe sanitation

## Flowchart

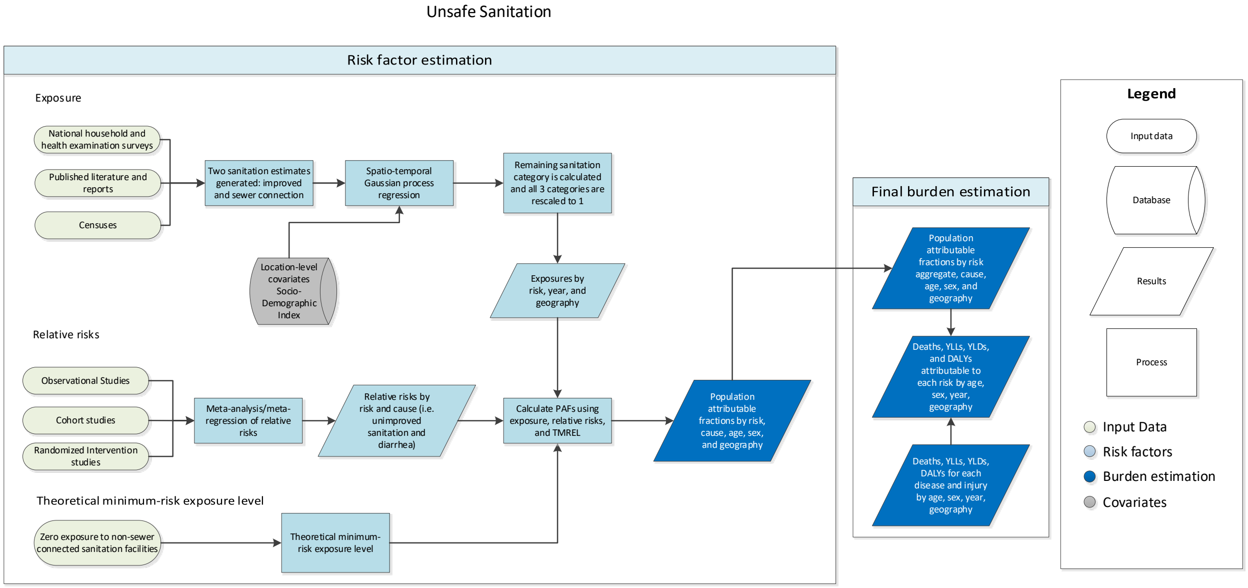

## Input data and methodological summary

### Exposure

#### Case definition

Exposure to unsafe sanitation is defined based on the primary toilet type used by households. For the Global Burden of Diseases, Injuries, and Risk Factors Study (GBD) 2021, we modelled three different categories of sanitation: unimproved, improved, and facilities with a sewer connection or septic tank. These categories were defined according to the WHO/UNICEF Joint Monitoring Programme for Water Supply, Sanitation and Hygiene (JMP).[1] Examples of "improved" sanitation facilities include ventilated improved pit latrines, composting toilets, and pit latrines with slabs. Examples of "unimproved" facilities include open pit latrines, open defecation, and toilets that flush into creeks or open fields. Sewer connection toilets include flush toilets or any toilet with connection to the sewer or septic tank.

#### Input data

The search for usable data sources was conducted using the Global Health Data Exchange (GHDx) database. Input data came primarily from nationally representative surveys, such as the Demographic and Health Survey (DHS), the Multiple Indicator Cluster Surveys (MICS), the World Health Survey (WHS), and the DHS AIDS Indicator Survey (AIS). Surveys that reported results at the household level were converted to the individual level using household size data, to ensure that our models estimated the proportion of individuals, rather than households, exposed to a given indicator. Surveys and censuses were then tabulated to two sanitation categories, sewer connection and improved sanitation, for each location. Table 1 provides a summary of the input data used.

Table 1: Exposure input data

| Input data | Exposure |
|---|---|
| Source count (total) | 1153 |
| Number of countries with data | 159 |

### Modelling

For GBD 2021, sanitation was modelled in an ordinal framework. Two distinct indicators were estimated: (1) the proportion of the total population using sewer connection or septic tank facilities, and (2) the proportion of individuals using improved sanitation within the population not connected to a sewer or septic tank. This ordinal framework allows us to estimate the category with the most data (sewer connection/septic tank prevalence) and leverage that estimate to anchor the estimates for the improved and unimproved sanitation categories. The results of the improved-proportion model are multiplied by one minus the sewer connection/septic tank prevalence to calculate improved sanitation prevalence. The sum of improved and sewer connection/septic tank prevalence are subtracted from 1 to yield unimproved sanitation prevalence.

$$\boldsymbol{Sewer} = \frac{\#\ persons\ with\ sewer\ or\ septic\ connection}{\#\ persons\ with\ nonmissing\ response}$$

$$\boldsymbol{Improved} = \left(\frac{\#\ persons\ using\ improved\ facilities}{\#\ persons\ without\ sewer\ or\ septic\ connection}\right) * (1 - \boldsymbol{Sewer})$$

$$\boldsymbol{Unimproved} = 1 - (\boldsymbol{Sewer} + \boldsymbol{Improved})$$

The two indicators were each modelled using a three-step modelling scheme of mixed effect linear regression followed by spatiotemporal Gaussian process regression (ST-GPR), which produced full time-series estimates for each GBD 2021 location. Socio-demographic Index (SDI), a composite metric combining education per capita, income per capita, and fertility, was set as a fixed effect in the linear regression since it proved to be a significant predictor. Random effects were set at GBD 2021 region and super-region levels to fit the models but were not used in the predictions. The same linear regression equation was used for both ST-GPR models (see below).

$$logit(data) \sim sdi + (1|level\_1) + (1|level\_2)$$

*SDI = Socio-demographic Index*
*(1|level_1) = super-region-level random effects*
*(2|level_2) = region-level random effects*

The process of vetting and validating models was accomplished primarily through an examination of ST-GPR scatterplots by GBD 2021 location from 1990 to 2021. Any poorly fitting datapoints were re-inspected for error at the level of extraction and survey implementation. If errors in data extraction were found, the study in question was re-extracted. In addition to SDI, a number of different potential fixed effects were considered, including lag-distributed income and urbanicity, but SDI proved to be the strongest predictor of unsafe sanitation in terms of magnitude of the coefficient. Uncertainty in the estimates was initially constructed based on standard deviation around each survey mean, then propagated through ST-GPR modelling by incorporating the variance of each datapoint in the Gaussian process regression step. A datapoint with high variance, for example, would contribute relatively less influence to the model than a datapoint with lower variance.

Once models are vetted, full time-series outputs from ST-GPR modelling are then rescaled using the above equations to form three mutually exclusive categories that sum up to 1 for each location-year combination. Table 2 provides the final result of this rescaling.

*Table 2: Exposure categories and definitions*

| **Category** | **Definition** |
|---|---|
| Unimproved sanitation | Proportion of individuals that use unimproved sanitation facilities. |
| Improved sanitation | Proportion of individuals that use improved sanitation facilities, excluding sewer connection or septic tank. |
| Sanitation facilities with sewer connection or septic tank | Proportion of individuals that use toilet facilities with sewer connection or septic tank. |

## Theoretical minimum-risk exposure level

The theoretical minimum-risk exposure level for unsafe sanitation was defined as having access to a sanitation facility with sewer connection or septic tank.

## Relative risks

For GBD 2021, unsafe sanitation was paired with one outcome, diarrhoeal diseases. Two meta-analyses, by Wolf et al 2014 and Wolf et al 2018, along with a literature review that used the same search terms as Wolf et al 2014, were used to identify relative risk studies.[2,3] Table 3 provides a summary of the relative risk data used.

Table 3: Relative risk input data

| Input data | Relative risk |
|---|---|
| Source count (total) | 16 |
| Number of countries with data | 13 |

In GBD 2021, relative risk values were calculated using a network meta-analysis approach with a tool called meta-egression—Bayesian, regularised, trimmed (MR-BRT). One study-level covariate – whether or not the study was generalisable to the general population – was included in the network meta-analysis. Several other covariates (whether exposure was captured at the individual level or population level; whether conformity with the study interventions was self-reported or confirmed by the researchers; whether or not the study was randomised; whether or not the study adjusted for all major known confounders; and what the study's follow-up percentage was) were considered but ultimately were not statistically significant and so were not used in analysis. No priors were used. We calculated the risk of developing diarrhoea for those using improved sanitation facilities and sewer or septic facilities, relative to the reference category of those using unimproved facilities. Those model results were then rescaled so that the relative risk for using sewer or septic facilities was the reference category, in order to match with our exposure definition. Table 4 shows the results of the MR-BRT analyses. Table 5 shows the relative risks that were ultimately used for modelling.

*Table 4: MR-BRT network meta-analysis results (reference: unimproved sanitation)*

| Intervention | Relative risk (95% CI) |
|---|---|
| Improved sanitation | 0.795 (0.739–0.856) |
| Sanitation facilities with sewer connection or septic tank | 0.310 (0.274–0.352) |

*Table 5: Relative risks for each exposure category (reference: sewer or septic facilities)*

| Exposure category | Relative risk (95% CI) |
|---|---|
| Unimproved sanitation | 3.22 (2.74–3.76) |
| Improved sanitation | 2.57 (2.08–3.12) |
| Sanitation facilities with sewer connection or septic tank | 1 (reference) |

Figure 1 shows the results of the MR-BRT analysis in graphical form, along with the associated "risk-outcome scores" for each category, which is a measure of how good the evidence is for that particular relative risk estimate. Prior to generating an risk-outcome score, we conducted an additional post-analysis step to detect and flag publication bias in the input data. This approach is based on the classic Egger's regression strategy, which is applied to the residuals in our model. In the current implementation, we do not correct for publication bias, but flag the risk–outcome pairs where the risk for publication bias is significant.

We did not detect publication bias based on the association between observation residuals and their standard errors (p-value = 0.337, Egger mean = -0.102, Egger SD = 0.243). The overall risk-outcome score for unsafe sanitation is 0.518, which is the maximum of the individual category scores.

*Figure 1: Risk-outcome scores*

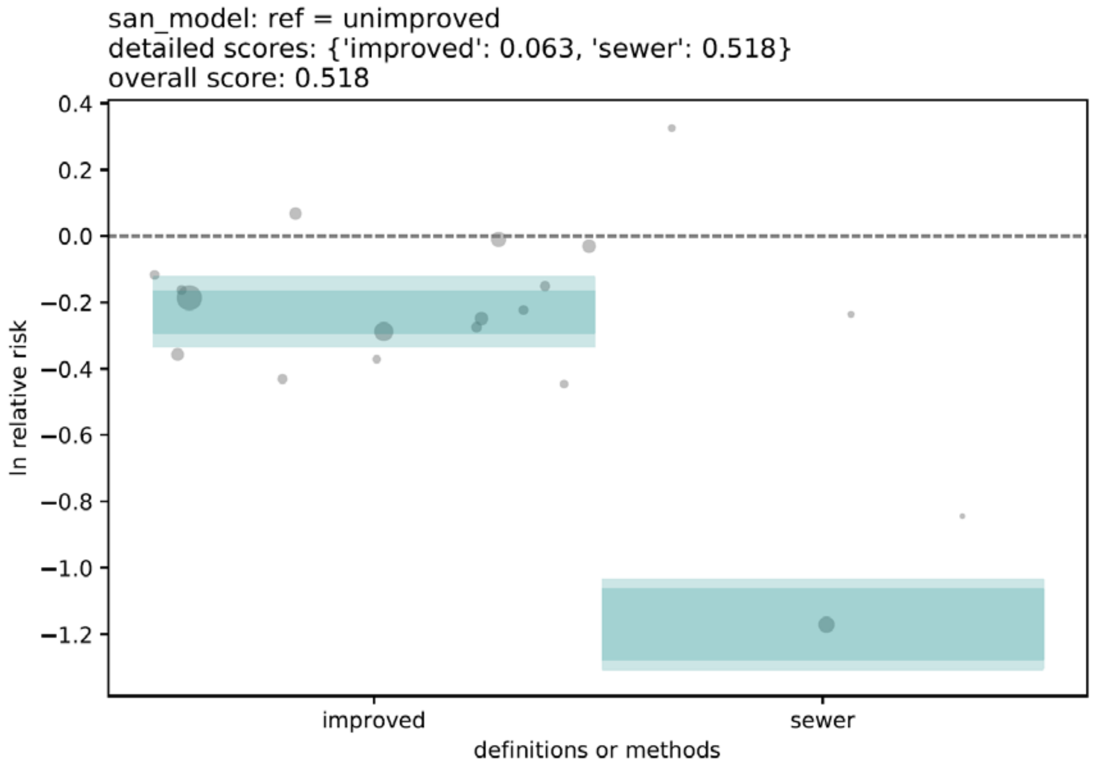


## Unsafe Sanitation References


1. WHO/UNICEF Joint Monitoring Programme: Sanitation. https://washdata.org/monitoring/sanitation (accessed Oct 31, 2019).
2. Wolf J, Pruss-Ustun A, Cumming O, *et al.* Assessing the impact of drinking water and sanitation on diarrhoeal disease in low- and middle-income settings: systematic review and meta-regression. *Tropical Medicine and International Health* 2014; **19**: 928–42.
3. Wolf J, Hunter PR, Freeman MC, *et al.* Impact of drinking water, sanitation and handwashing with soap on childhood diarrhoeal disease: updated meta-analysis and meta-regression. *Tropical Medicine and International Health* 2018; **23**: 508–25.

# No access to handwashing facility

## Flowchart

Unsafe Handwashing

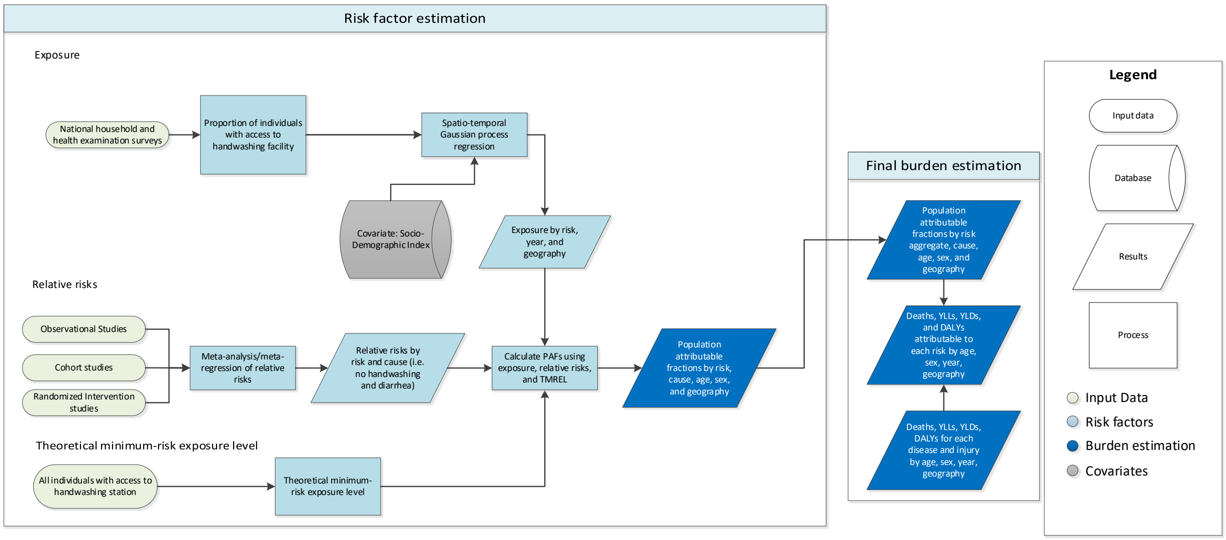


## Input data and methodological summary

## Exposure

### *Case definition*

This risk is defined as the proportion of the population without access to a handwashing facility with soap (bar, liquid, or powder/detergent), water, and wash station (either permanent or mobile).[1] If any of these is missing, then the individual is counted as not having access.

### Input data

Input data came primarily from geographically representative household surveys, including the Demographic and Health Surveys (DHS), Multiple Indicator Cluster Surveys (MICS), and Performance Monitoring and Accountability 2020 (PMA2020) surveys. For the GBD 2021 study, a large number of new data sources were added, nearly doubling the total number of sources from 98 in GBD 2019 to 177 in GBD 2021. We also re-extracted all sources previously used to ensure that we were capturing individual-level exposure, in an effort to align with the WHO/UNICEF Joint Monitoring Programme's methodology.[1] As a result of this effort, we excluded several studies used in previous GBD rounds that included ash, sand, or soil in their definition of "soap." Table 1 provides a summary of the exposure input data.

*Table 1: Exposure input data*

| Input data | Exposure |
|---|---|
| Source count (total) | 177 |
| Number of countries with data | 89 |

### *Modelling strategy*

We modelled exposure to this risk using a three-step modelling scheme of mixed effect linear regression followed by spatiotemporal Gaussian process regression (ST-GPR), which outputs full time-series estimates for

each GBD 2021 location. Two covariates were used as fixed effects in the linear regression: Socio-demographic Index (SDI), which is a composite measure of development that includes income per capita, education, and fertility, and proportion of individuals with access to piped water (see below for model equation). Random effects were set at GBD 2021 region and super-region levels to fit the model but were not used in the predictions.

$$logit(data) \sim sdi + piped\ water\ access + (1|level\_1) + (1|level\_2)$$

*SDI = Socio-demographic Index*
*Piped water access = proportion of individuals with access to piped water*
*(1|level_1) = super-region-level random effects*
*(2|level_2) = region-level random effects*

The process of vetting and validating models was accomplished primarily through an examination of ST-GPR scatterplots by GBD 2021 location from 1990 to 2021. Any poorly fitting datapoints were re-inspected for error at the level of extraction and survey implementation. If errors in data extraction were found, the study in question was re-extracted. In addition to SDI, a number of different potential fixed effects were considered, including lag-distributed income and urbanicity. However, SDI proved to be the strongest predictor.

## Theoretical minimum-risk exposure level

The theoretical minimum-risk exposure level for unsafe hygiene is defined as having access to a handwashing facility with soap (bar, liquid, or powder/detergent), water, and wash station (either permanent or mobile).

## Relative risks

### *Input data*

Input data included in the GBD 2021 hygiene relative risk analysis are as follows:

*Table 2: Relative risk input data*

| Input data | Relative risk |
|---|---|
| Source count (total) | 41 |
| Number of countries with data | 22 |

For GBD 2021, unsafe hygiene was paired with two outcomes: diarrhoeal diseases and lower respiratory infections (LRI). A meta-analysis by Cairncross and colleagues 2010[2] provided relative risk values describing the relationship between lack of facility access and diarrhoeal diseases. A meta-analysis by Rabie & Curtis 2006[3] provided relative risk evidence for the relationship between lack of facility access and LRI, including the years 1997-2004.

### *Modelling strategy*

In GBD 2021, relative risk values were calculated using a tool called meta-regression—Bayesian, regularised, trimmed (MR-BRT). For the both the diarrhoea model and the LRI model, two study-level covariates were included – whether or not the study was randomised and whether or not the percentage of the study

population lost to follow-up was greater than 15%. No priors were used. Table 3 shows the results of the relative risk analyses.

*Table 3: Relative risks (reference: access to handwashing facility)*

| Outcome | Relative risk (95% CI) |
|---|---|
| Diarrhoeal diseases | 1.52 (1.06–2.12) |
| Lower respiratory infections | 1.43 (0.82–2.30) |

Figures 2 and 3 show the funnel plots for each MR-BRT analysis, along with the associated "risk-outcome scores," which measure how good the evidence is for that particular relative risk estimate. Prior to generating an risk-outcome score, we conducted an additional post-analysis step to detect and flag publication bias in the input data. This approach is based on the classic Egger's regression strategy, which is applied to the residuals in our model. In the current implementation, we do not correct for publication bias, but flag the risk–outcome pairs where the risk for publication bias is significant.

For hygiene and diarrhoea, we detected publication bias based on the association between observation residuals and their standard errors (p-value = 0.0265, Egger mean = -0.302, Egger SD = 0.156). The risk-outcome score was 0.000828.

*Figure 2: Hygiene–diarrhoea funnel plot and risk-outcome score*

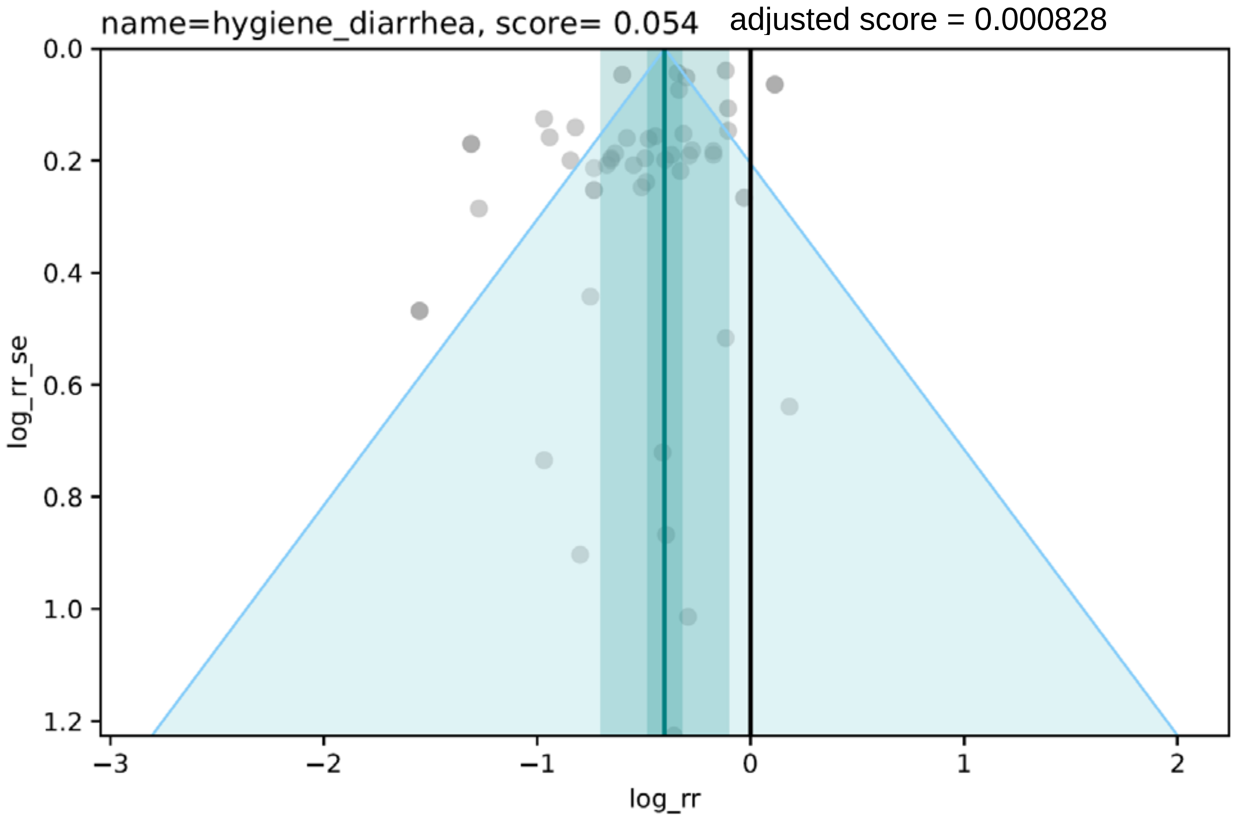

## No access to handwashing facility References


1. WHO/UNICEF. JMP Methodology: 2017 update and SDG baselines. 2018.

2. Cairncross S, Hunt C, Boisson S, *et al.* Water, sanitation and hygiene for the prevention of diarrhoea. *International Journal of Epidemiology* 2010; **39**: i193–205.

3. Rabie T, Curtis V. Handwashing and risk of respiratory infections: a quantitative systematic review. *Tropical Medicine and International Health* 2006; **11**: 258–67.

4. Jefferson T, Del Mar CB, Dooley L, Ferroni E, Al-Ansary LA, Bawazeer GA, van Driel ML, Jones MA, Thorning S, Beller EM, Clark J, Hoffmann TC, Glasziou PP, Conly JM. Physical interventions to interrupt or reduce the spread of respiratory viruses. Cochrane Database of Systematic Reviews 2020, Issue 11. Art. No.: CD006207. DOI: 10.1002/14651858.CD006207.pub5. Accessed 13 April 2022.

# Child growth failure

## Combined Flowchart for Child Growth Failure and Protein Energy Malnutrition

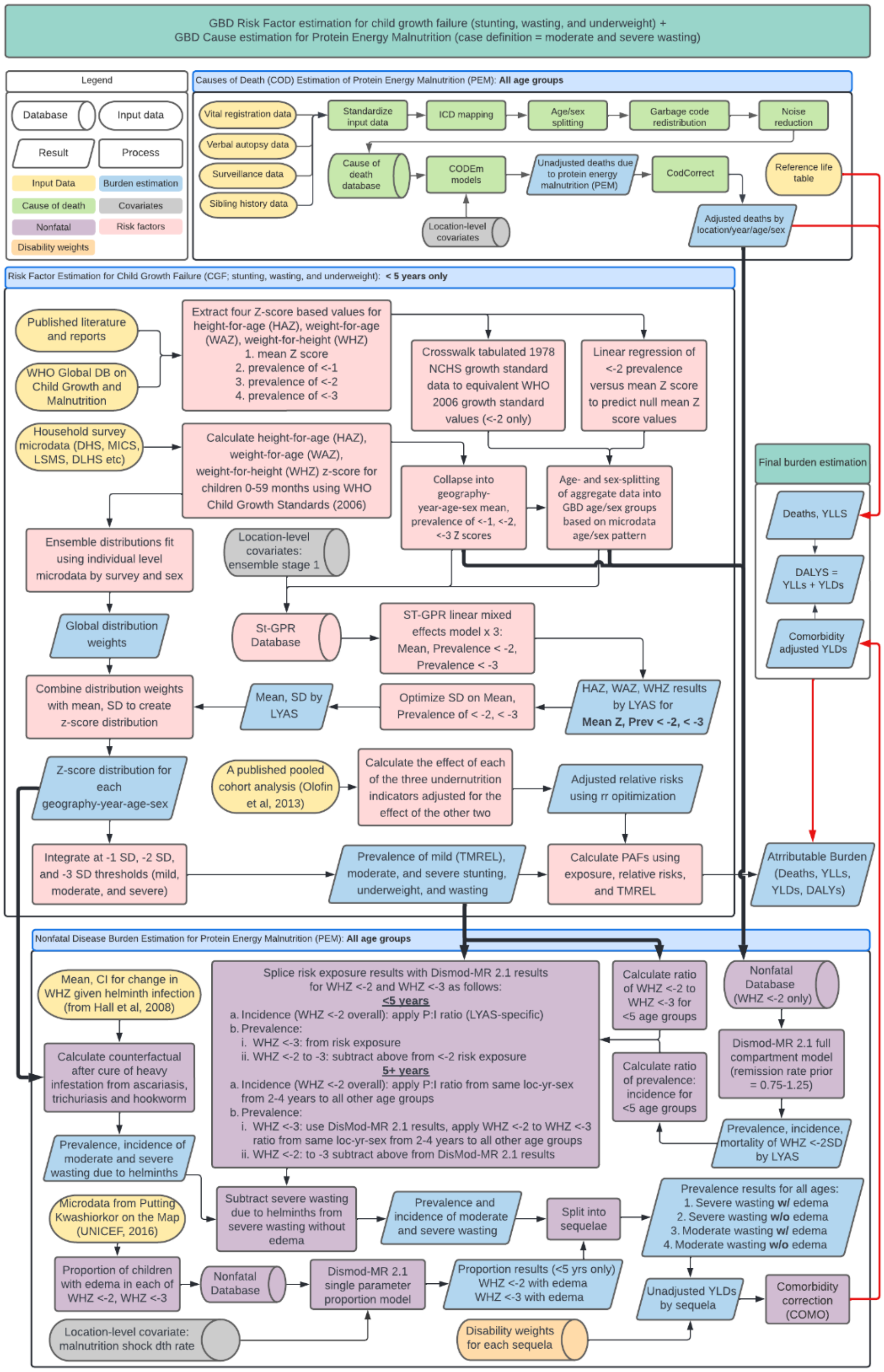

## Input data and methodological summary

### Exposure

*Case definition*

Child growth failure (CGF) is estimated using three indicators (stunting, wasting, and underweight), all of which are based on categorical definitions using the WHO 2006 growth standards for children 0-59 months. Definitions are based on Z scores from the growth standards, which were derived from an international reference population. Mild (<-1 to -2 Z score), moderate (<-2 to -3 Z score), and severe (<3 Z score) categorical prevalences were estimated for each of the three indicators.

*Input data*

There are three main inputs for the GBD child growth failure models: microdata from population surveys, tabulated data from reports and published literature, and the WHO Global Database on Child Growth and Malnutrition.[15] The primary data additions in GBD 2021 for child growth failure were from population surveys that include anthropometry. Population surveys include a variety of multi-country and country-specific survey series such as Multiple Indicator Cluster Surveys (MICS), Demographic and Health Surveys (DHS), Living Standards Measurement Surveys (LSMS), and the China Health and Nutrition Survey (CHNS), as well as other one-time country-specific surveys such as the Indonesia Family Life Survey and the Brazil National Demographic and Health Survey of Children and Women. These microdata contain information about each individual child's age (from which age in weeks and age in months are calculated), as well as height and/or weight. From that information, a height-for-age z-score (HAZ), weight-for-age z-score (WAZ), and weight-for-height z-score (WHZ) are calculated using the WHO 2006 Child Growth Standards and the LMS method.[16] Data that did not meet the following three criteria were dropped: 1) non-sex-specific data, 2) data with invalid Z-scores (HAZ, WAZ, WHZ, or BMI above 6 SD or below -6 SD), and 3) data with impossible values (negative height, weight, or age).

All available data from the WHO Global Database on Child Growth and Malnutrition were extracted in GBD 2016 – much of which are from published studies. Exclusions included examination date prior to 1985, non-population-representative studies, and those based on self-report. A systematic literature review was last completed in GBD 2010. We looked for four metrics from all sources with tabulated data: mean Z score, prevalence <-1 Z score, prevalence <-2 Z score, and prevalence <-3 Z score. All data for each metric were extracted for each of stunting (height-for-age Z score; HAZ), wasting (weight-for-height Z score; WHZ), and underweight (weight-for-age Z score; WAZ).

**Table 1: Input data counts for Child wasting exposure models**

| Input data | Exposure |
|---|---|
| Source count (total) | 1908 |
| Number of countries with data | 159 |

**Table 2: Input data counts for Child underweight exposure models**

| Input data | Exposure |
|---|---|
| Source count (total) | 1897 |
| Number of countries with data | 160 |

**Table 3: Input data counts for Child stunting exposure models**

| Input data | Exposure |
|---|---|
| Source count (total) | 1897 |
| Number of countries with data | 160 |

*Data processing*

To maximise internal consistency and comprehensiveness of the modelling dataset, we performed three data transformations. First, any data that were reported using the National Center for Health Statistics (NCHS) 1978 growth standards were crosswalked to corresponding values on the WHO 2006 Growth Standards curves based on a study that evaluated growth standard concordance.[17] Crosswalks from 1978 to 2006 growth standards were performed using OLS linear regression only on <-2 (ie, moderate) prevalence data, as that is where the concordance was most consistent. Second, for any study that lacked a measure of mean Z score for any of stunting, wasting, or underweight, we predicted a mean value for that study based on an ordinary-least-squares regression of mean Z score versus <-2 prevalence for that metric from all sources where both were available. Third, for any data that were presented as both sexes combined or for 0-59 months combined, we used the age and sex pattern from all data sources that included that detail to split into corresponding and age- and sex-specific data.

## Modelling strategy

*Exposure estimation*

The following four-step modelling process was applied in parallel to each of stunting, wasting, and underweight.

First, all microdata were fit using an ensemble modelling. A series of 10 individual distributions (normal, log-normal, log-logistic, exponential, gamma, mirror gamma, inverse gamma, gumbel, mirror gumbel, and Weibull) were fit simultaneously to each microdata source in the dataset. All component distributions that were used to derive weights were parameterised using "method of moments," meaning that each corresponding probability density function (PDF) could be described as a function of the mean and variance of the quantity of interest. From these distribution families, an ensemble distribution was parameterised using an updated methodology for GBD 2021, which has 2 main advantages over GBD 2019 methodology. Those advantages are described below.

The new ensemble modeling strategy is considered an advancement, in part, because the models were specifically fit on the portions of the distributions that constitute mild, moderate, and severe CGF. While

previous methods aimed to minimise predictive error across the entire distribution, the new GBD 2021 method aimed to minimise absolute prediction error in highly relevant areas of the curve.

The second advancement is that the optimisation process considers the fit across all microdata sources simultaneously. Therefore, the algorithm targets the set of ensemble weights that minimises the predictive error across all microdata sources collectively, as opposed to finding one set of weights for each individual microdata source and averaging those sets of weights together.

After ensemble distributions have been parameterised, the second modeling step begins. Models were developed for mean Z scores and prevalence of moderate and severe growth failure. Individual-level microdata were collapsed to calculate three metrics: mean z-score, moderate prevalence, and severe prevalence. These data were combined with those derived from literature, GHDx review, and the WHO Global Database on Child Growth and Malnutrition. Each of the three metrics was then modelled using spatiotemporal Gaussian process regression (ST-GPR), a common modelling framework used across GBD, generating estimates for each age group, sex, year, and location. Location-level covariates used in all models included Socio-demographic Index (SDI) and logit-transformed proportion of households with improved sanitation.

Third, we combined estimates of mean, prevalence (moderate and severe) with ensemble weights in an optimisation framework in order to derive the variance that would best correspond to the predicted mean and prevalence. This variance was then paired with the mean and, using the method of moments equation for each of the component distributions of the ensemble, PDF of the distribution of Z-scores were calculated for each location, year, age group, and sex.

Fourth, PDFs were integrated to determine the prevalence between -1 and -2 Z scores (mild), between -2 and -3 Z scores (moderate), and below -3 Z scores (severe). These were categorical exposures used for subsequent attributable risk analysis.

### Theoretical minimum-risk exposure level

Theoretical minimum risk exposure level (TMREL) for underweight, stunting, and wasting was assigned to be greater than or equal to -1 SD of the WHO 2006 standard weight-for-age, height-for-age, and weight-for-height curves, respectively. This has not changed since GBD 2010.

### Relative risks

The final list of outcomes paired with child growth failure risks included mortality and morbidity for lower respiratory infections (LRI), diarrhoea, malaria, measles, and protein-energy malnutrition (PEM), as shown in Table 6. These were derived from a Burden of Proof analysis that incorporated both a pooled analysis of ten prospective cohort studies by Olofin and colleagues[5] as well as relative risk estimates from Knowledge Integration (KI) studies (Table 5). For the KI studies, aggregated relative risks of disease or cause-specific mortality were calculated for 1-unit z-score bins for stunting, wasting, and underweight (e.g., relative risk of diarrhea-attributable death in children 1 to 2 years of age and with a HAZ score between -4 and -3). The burden of proof analysis uses all available relative risks with corresponding uncertainty to create continuous relative risk curves for each outcome/risk pair. These continuous risk curves are then combined with the global exposure curves for HAZ, WAZ, and WHZ, to calculate exposure-weighted relative risks for severe, moderate, and mild stunting, wasting, and underweight with uncertainty.

**Table 4: Input data counts for Child growth failure relative risk models**

| Input data | Risk |
|---|---|
| Source count (total) | 53 |

There is a high degree of correlation between stunting, wasting, and underweight. Failing to account for their covariance and assuming independence would overestimate the total burden significantly and misrepresent the attributable burden of individual CGF indicators. Inability to address these correlations is the main reason that GBD 2010 only included childhood underweight.

In order to account for the high degree of correlation between CGF indicators, GBD uses a constrained optimisation method to adjust the observed univariate RRs that come out of the Burden of Proof analysis. First we created a joint distribution of stunting, underweight, and wasting from a population of children. Second, we generated one thousand RR draws for each univariate indicator and severity based from the Burden of Proof analysis. Third, we altered these univariate RRs for the four causes (diarrhoea, LRI, malaria, and measles) and the two outcomes (mortality and morbidity) based upon interactions among the CGF indicators. An interaction occurs when the effect of one CGF indicator variable (eg, stunting) has a different effect on the outcome depending on the value of another CGF indicator variable (eg, underweight). Interaction terms alter the risk of the outcome among children with more than one indicator of CGF. These interaction terms were extracted from a pooled cohort analysis of all-cause mortality published by McDonald et al. [18] Lastly, we optimised the adjusted relative risks by minimising the error between the observed RRs (generated from Olofin et al.) and the altered RRs derived from the joint distribution and accounting for the interaction terms while ensuring that no alteration resulted in a previously identified increase in relative risk becoming protective.

For GBD 2021, we made several changes to improve the four main steps of RR adjustment. From GBD 2013 to GBD 2019, a simulated joint distribution of stunting, underweight, and wasting measures was created from the Olofin et al. meta-analysis. Sources in this meta-analysis were cross-sectional Demographic and Health Surveys (DHS). In GBD 2021, we created age-specific joint distributions of stunting, underweight, and wasting measures from 15 longitudinal studies (from 26 locations) in the Bill and Melinda Gates Foundation's Knowledge Integration (Ki) database[6]. (Study details are provided in Table 5). The RR adjustment method was strengthened in GBD 2021 by constraining optimisation in two ways. Optimisation was only permitted to alter the RR for an indicator/severity in draws where the observed RR was greater than 1, and constraints were placed on the error that penalise larger alterations to the RR. These changes enabled the estimation and utilisation of age-specific adjusted RRs for GBD 2021 burden estimation. The largest changes for GBD 2021 was conducting Burden of Proof Analyses for each cause/outcome/risk triplet using both data from Olofin et al as well as KI data. These changes result in identifying large differences in the relationship between CGF and mortality versus morbidity as well as identifying some impact of CGF on malaria.

**Table 5: Bill and Melinda Gates Foundation Knowledge Integration (KI) database study details**

| Study name | Country | Sample Size | Years conducted |
|---|---|---|---|
| Zimbabwe Vitamin A for Mothers and Babies Trial | ZWE | 14,110 | 1997-2001 |
| CMC Vellore Birth Cohort Study | IND | 373 | 2002-2006 |
| International Lipid-Based Nutrient Supplements Project | MWI | 1,206 | 2011-2014 |
| Malnutrition and Enteric Disease Study | BGD | 265 | 2009-2017 |
| Malnutrition and Enteric Disease Study | IND | 251 | 2009-2017 |
| Malnutrition and Enteric Disease Study | NEP | 240 | 2009-2017 |
| Malnutrition and Enteric Disease Study | PER | 303 | 2009-2017 |
| Malnutrition and Enteric Disease Study | BRA | 233 | 2009-2017 |
| Malnutrition and Enteric Disease Study | ZAF | 314 | 2009-2017 |
| Malnutrition and Enteric Disease Study | TZA | 262 | 2009-2017 |
| Medical Research Council Keneba | GMB | 2,867 | - |
| Performance of Rotavirus and Oral Polio Vaccines In Developing Countries | BGD | 700 | 2011-2014 |
| Community-based Intervention Trial to Compare the Impact of Preventive and Therapeutic Zinc Supplementation Programs Among Young Children in Burkina Faso | BFA | 7,634 | 2010-2012 |
| WASH Benefits Bangladesh | BGD | 4,423 | 2011-2014 |
| WASH Benefits Kenya | KEN | 5,649 | 2012-2016 |
| Promotion of Breastfeeding Intervention Trial | BLR | 16,897 | 1996-1998 |
| Childhood Malnutrition and Infection Network | BGD | 477 | 1993-1996 |
| Childhood Malnutrition and Infection Network | BRA | 119 | 1989-1998 |
| Childhood Malnutrition and Infection Network | GNB | 350 | 1987-1990 |
| Childhood Malnutrition and Infection Network | GNB | 885 | 1996-1997 |
| Childhood Malnutrition and Infection Network | PER | 210 | 1989-1991 |
| Childhood Malnutrition and Infection Network | PER | 224 | 1995-1998 |
| Delhi Infant Vitamin D Study | IND | 2,100 | 2007-2010 |
| Characterization of Respiratory pathogens endemic to Pakistan in pregnant women and newborns in urban settings | PAK | 380 | 2012-2013 |
| Impact of Zinc Supplementation in Low Birth Weight Infants on Severe Morbidity, Mortality and Zinc Status: A Randomized Controlled Trial | IND | 2,052 | 2005-2007 |
| A Trial of Zinc and Micronutrients in Tanzanian Children | TZA | 2,400 | 2007-2012 |

## Table 6: Age-Specific Adjusted RRs for each risk-outcome pair for child growth failure

| 1 to 5 months | | Incidence | | | Mortality | | |
|---|---|---|---|---|---|---|---|
| Cause | | <-3 | -3,-2 | -2,-1 | <-3 | -3,-2 | -2,-1 |
| Diarrhoea | HAZ | 1.2 (0.8, 1.7) | 1.2 (0.8, 1.6) | 1.1 (0.9, 1.5) | 3.6 (2.1, 4.4) | 2.1 (1.6, 2.6) | 1.4 (1.2, 1.6) |
| | WAZ | 1.6 (0.9, 2.7) | 1.6 (0.9, 2.6) | 1.5 (0.9, 2.4) | 6.7 (4.4, 9.3) | 3.4 (2.2, 4.7) | 1.8 (1.3, 2.2) |
| | WHZ | 1.3 (0.8, 1.8) | 1.2 (0.9, 1.7) | 1.2 (0.9, 1.6) | 40.8 (0.8, 224.7) | 12.8 (0.8, 51.5) | 4.1 (0.9, 10.6) |
| **6 to 11 months** | | **Incidence** | | | **Mortality** | | |
| **Cause** | | **<-3** | **-3,-2** | **-2,-1** | **<-3** | **-3,-2** | **-2,-1** |
| Diarrhoea | HAZ | 1.2 (0.8, 1.7) | 1.2 (0.8, 1.6) | 1.1 (0.9, 1.5) | 3.1 (2.2, 3.9) | 1.9 (1.5, 2.4) | 1.3 (1.2, 1.6) |
| | WAZ | 1.6 (0.9, 2.7) | 1.6 (0.9, 2.6) | 1.5 (0.9, 2.4) | 5.9 (3.7, 8.9) | 3.1 (2.0, 4.5) | 1.7 (1.3, 2.2) |
| | WHZ | 1.3 (0.8, 1.8) | 1.2 (0.9, 1.7) | 1.2 (0.9, 1.6) | 40.7 (0.8, 224.7) | 12.7 (0.8, 51.5) | 4.1 (0.9, 10.6) |

| 12 to 23 months | | Incidence | | | Mortality | | |
|---|---|---|---|---|---|---|---|
| Cause | | <-3 | -3,-2 | -2,-1 | <-3 | -3,-2 | -2,-1 |
| Diarrhea | HAZ | 1.2 (0.8, 1.7) | 1.2 (0.8, 1.6) | 1.1 (0.9, 1.5) | 2.7 (2.2, 3.4) | 1.7 (1.4, 2.2) | 1.3 (1.1, 1.5) |
| | WAZ | 1.6 (0.9, 2.7) | 1.6 (0.9, 2.6) | 1.5 (0.9, 2.4) | 5.4 (3.7, 8.4) | 2.9 (2.0, 4.4) | 1.6 (1.3, 2.1) |
| | WHZ | 1.3 (0.8, 1.8) | 1.2 (0.9, 1.7) | 1.2 (0.9, 1.6) | 40.7 (0.8, 225.5) | 12.7 (0.8, 51.7) | 4.0 (0.9, 10.6) |

| 2 to 4 years | | Incidence | | | Mortality | | |
|---|---|---|---|---|---|---|---|
| Cause | | <-3 | -3,-2 | -2,-1 | <-3 | -3,-2 | -2,-1 |
| Diarrhoea | HAZ | 1.2 (0.8, 1.7) | 1.2 (0.8, 1.6) | 1.1 (0.9, 1.5) | 2.8 (2.1, 3.4) | 1.8 (1.5, 2.2) | 1.3 (1.2, 1.5) |
| | WAZ | 1.6 (0.9, 2.7) | 1.6 (0.9, 2.6) | 1.5 (0.9, 2.4) | 5.6 (4.0, 8.3) | 2.9 (2.1, 4.3) | 1.6 (1.3, 2.1) |
| | WHZ | 1.3 (0.8, 1.8) | 1.2 (0.9, 1.7) | 1.2 (0.9, 1.6) | 41.3 (0.8, 229.9) | 12.8 (0.8, 52.4) | 4.0 (0.9, 10.7) |

## Child Growth Failure References


6 Jumbe NL, Murray JC, Kern S. Data Sharing and Inductive Learning – Toward Healthy Birth, Growth, and Development. N Engl J Med. 2016 Jun 23;374(25):2415-7. doj: 10.1056/NEJMp1605441. Epub 2016 May 11. PMID: 27168111.

# Low birthweight and short gestation

## Flowchart

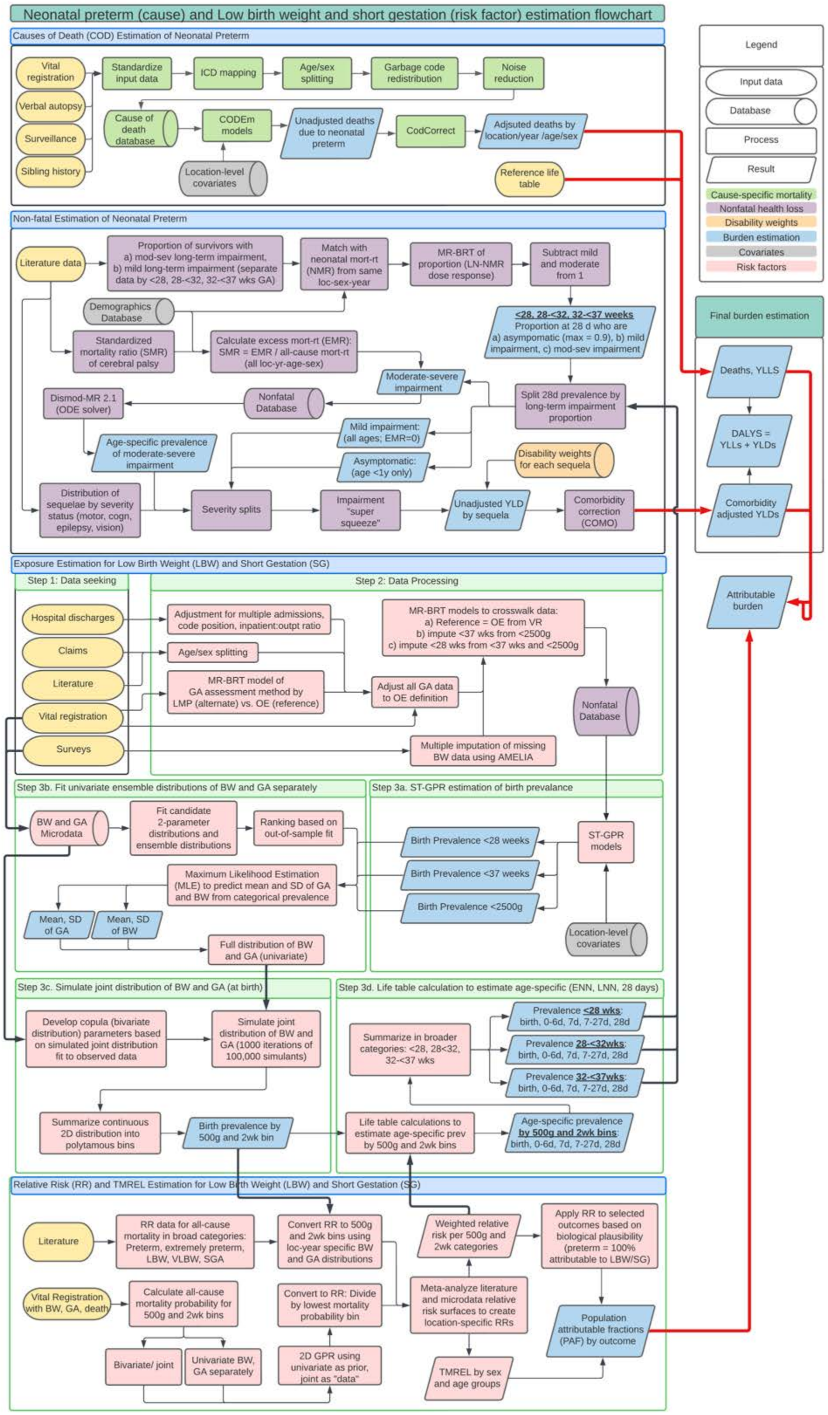

## Input data and methodological summary

Short gestational age and low birthweight are highly correlated risk factors associated with poor child health outcomes. The "low birthweight and short gestation" (LBWSG) risk factor quantifies the burden of disease attributable to increased risk of death and disability due to 1) less than ideal birthweight ("low birthweight") and 2) shorter than ideal length of gestation ("short gestation").

Within GBD, attributable burden is generally estimated separately for each individual risk factor, but the combined burden attributable to multiple risk factors is of general interest. In GBD, attributable burden due to multiple risk factors is typically estimated through a "mediation analysis" that is applied after independent estimation of each risk factor's exposure, relative risk, theoretical minimum risk exposure level (TMREL), and population attributable fraction (PAF). In the mediation analysis, a "mediation factor" adjusts the PAF of each risk factor by the amount of attributable burden mediated through the other GBD risk factors. While mediation may be common, direct quantification of the joint exposure, relative risk, and PAF of the combined risk factors is conceptually more straightforward.

In GBD 2016, LBWSG became the first (and, as of GBD 2021, only) group of GBD risk factors in which combined attributable burden is quantified by direct estimation of the joint exposure, relative risk, TMREL, and PAF of multiple risk factors. After first directly estimating the joint exposure, relative risk, TMREL, and PAF of birthweight and gestational age together, we then separate out the independent PAFs due to birthweight only or gestational age only. Because of this modelling strategy, the joint GBD risk factor quantifying the burden of disease due to both less than ideal birthweight ("low birthweight") and shorter than ideal gestational age ("short gestation") is grouped into a single "parent" risk factor termed "low birthweight and short gestation". LBWSG is disaggregated into two "child" risk factors: "low birthweight for gestation" and "short gestation for birthweight". Low birthweight for gestation quantifies the burden of disease attributable to less than ideal birthweight, after adjusting for the influence of gestational age. Likewise, short gestation for birthweight quantifies the burden of disease attributable to shortened gestational age, after adjusting for the influence of birthweight.

Ideally, the model for joint exposure and joint relative risk would be fully continuous. To simplify the computation for the analysis, a grid of 500-gram and 2-week units ("bins") is used as the LBWSG dimensions and to approximate a fully continuous joint distribution model (see Figure 1).

**Figure 3: Fully continuous analysis of joint gestational age and birthweight (left) is approximated with a grid of birthweight and gestational age with 500-gram and 2-week "bins" (right)**

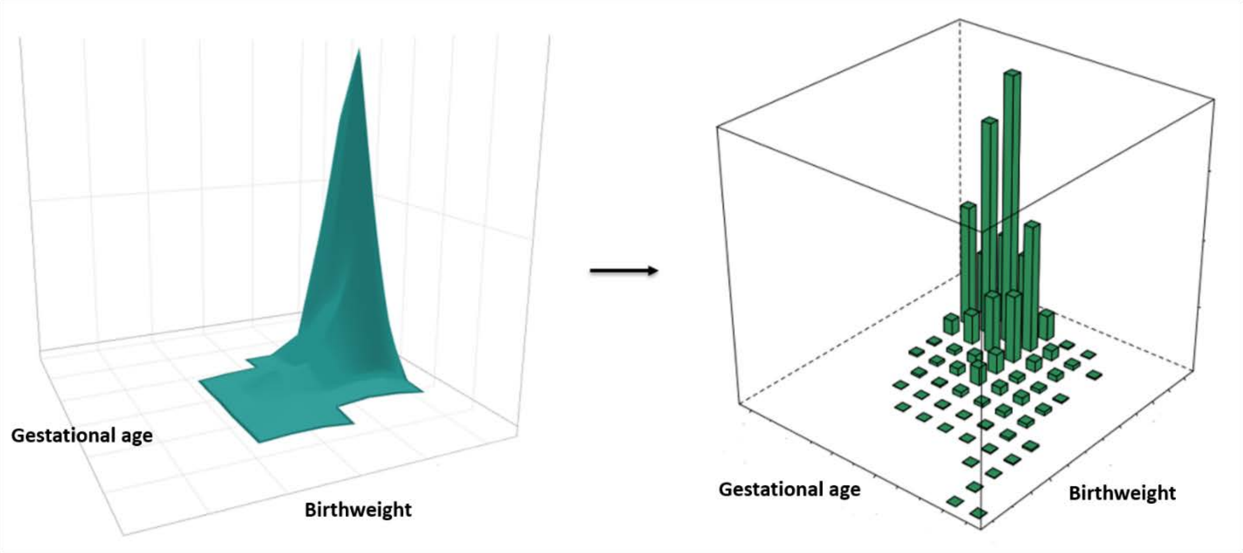


## Case definition

"Low birthweight" has historically referred to any birthweight less than 2500 grams, dichotomising birthweight into two categories: "normal" and "low". In the context of the GBD LBWSG risk factor, low birthweight refers to any birthweight less than the birthweight TMREL (the birthweight that minimises risk at the population level). Because LBWSG is estimated in a grid of 500-gram and 2-week bins, any 500-gram birthweight unit less than the TMREL, which was determined as [38, 40) weeks and [3500, 4000) g for the LBWSG parent risk factor, is considered "low birthweight". This includes, for example, birthweight of [2500, 3000) grams, which the traditional, dichotomous definition of "low birthweight" would not include.

Like birthweight, gestational age is typically classified into broad categories. "Preterm" is used to describe any newborn baby born less than 37 completed weeks of gestation. In the GBD context, "short gestation" is used to refer to all gestational ages below the gestational age TMREL.

## Exposure

In LBWSG, exposure refers to the portion of the joint distribution of gestational age and birthweight less than the TMREL, by location/year/sex (l/y/s), from birth to the end of the neonatal period. Modelling LBWSG exposure can be summarised in three steps:

A. Model univariate gestational age and birthweight distributions at birth, by l/y/s
B. Model joint distributions of gestational age and birthweight at birth, by l/y/s
C. Model joint distributions from birth to the end of the neonatal period, by l/y/s

**Table 1: Analytic steps in estimation of YLDs due to preterm birth**

| | Summary of exposure modelling strategy |
|---|---|
| **Step A**<br>Model univariate distributions at birth | 1. Model mean gestational age, prevalence of gestational age <28 weeks, and prevalence of gestational age <37 weeks, by l/y/s<br>2. Model mean birthweight and prevalence of birthweight <2500 grams, by l/y/s<br>3. Model univariate gestational age and birthweight distributions separately at birth, by l/y/s |
| **Step B**<br>Model joint distributions at birth | 1. Use copulae to model the correlation structure of the joint distribution of gestational age and birthweight, globally<br>2. Model the joint distribution of gestational age and birthweight, by location/year/sex at birth, by applying the globally modelled correlation structure to the location/year/sex-specific univariate models of gestational age and birthweight distributions |
| **Step C**<br>Model joint distributions from birth to 28 days | 1. Model all-cause mortality rates by gestational age and birthweight<br>2. Model gestational age and birthweight distributions of surviving neonates for all l/y/s from birth to end of the neonatal period, using all-cause mortality rates by gestational age and birthweight |

## Input data and data processing

Input data needed to model univariate gestational age and birthweight distributions at birth (Step A):

- Prevalence of preterm birth (<37 weeks), by l/y/s
- Prevalence of preterm birth (<28 weeks), by l/y/s
- Mean gestational age, by l/y/s
- Gestational age microdata
- Prevalence of low birthweight (<2500 grams), by l/y/s
- Mean birthweight, by l/y/s
- Birthweight microdata

To model joint distributions of gestational age and birthweight (Step B), joint microdata of gestational age and birthweight are also required. Additional inputs to modelling joint distributions from birth to 28 days (Step C) are all-cause mortality by l/y/s and joint birthweight and gestational age microdata linked to mortality outcomes.

Prevalence of extremely preterm birth (<28 weeks) and preterm birth (<37 weeks) were modelled using vital registration, survey, and clinical data. For the preterm models, only inpatient and insurance claims data were included from clinical informatics datasets; outpatient data were excluded because they were more likely to capture repeated visits by the same child rather than unique visits. Prevalence of low birthweight (<2500 grams) was modelled using only vital registration and survey data.

## *Literature review*

Before GBD 2016, available preterm birth data were sourced by a technical working group. In GBD 2016 and GBD 2017, we conducted systematic reviews to identify additional sources beyond the data already used in the models. The PubMed database was searched using the following search string:

> ((("Infant, Premature"[Mesh] OR ("infant"[All Fields] AND "premature"[All Fields]) OR "premature infant"[All Fields] OR ("preterm"[All Fields] AND "infant"[All Fields]) OR "preterm infant"[All Fields] OR ("infant, newborn"[MeSH Terms] OR ("infant"[All Fields] AND "newborn"[All Fields]) OR "newborn infant"[All Fields] OR ("newborn"[All Fields] AND "infant"[All Fields])) AND (premature[All Fields] OR preterm[All Fields]) OR "premature birth"[MeSH Terms] OR ("premature"[All Fields] AND "birth"[All Fields]) OR "premature birth"[All Fields] OR ("preterm"[All Fields] AND "birth"[All Fields]) OR "preterm birth"[All Fields]) ((("Infant, Premature"[Mesh] OR ("infant"[All Fields] AND "premature"[All Fields]) OR "premature infant"[All Fields] OR ("preterm"[All Fields] AND "infant"[All Fields]) OR "preterm infant"[All Fields] OR ("infant, newborn"[MeSH Terms] OR ("infant"[All Fields] AND "newborn"[All Fields]) OR "newborn infant"[All Fields] OR ("newborn"[All Fields] AND "infant"[All Fields])) AND (premature[All Fields] OR preterm[All Fields]) OR "premature birth"[MeSH Terms] OR ("premature"[All Fields] AND "birth"[All Fields]) OR "premature birth"[All Fields] OR ("preterm"[All Fields] AND "birth"[All Fields]) OR "preterm birth"[All Fields]) AND ("1985"[PDAT] : "3000"[PDAT]) AND "humans"[MeSH Terms].

The exclusion criteria were: studies that did not provide primary data on epidemiological parameters, non-representative studies (eg, only high-risk pregnancies), and reviews. Table 3 shows the search hits, number of full-texts reviewed, and number of extracted sources.

**Table 3. LBWSG search hits, full-text review, extracted sources**

| Search | Hits | Full-text review | Extracted | Search date |
|---|---|---|---|---|
| GBD 2017 | 16,174 | 2200 | 154 | 6/6/2017 |

**Table 4. Input data for exposure models**

| Input data | Exposure |
|---|---|
| Source count (total) | 2233 |
| Number of countries with data | 176 |

## *Data processing*

Any data that didn't fit a GBD age groups was split into age groups using a model that was run using only age-specific data. Starting in GBD 2019, as was the case with all other non-fatal analyses, we applied empirical age and sex ratios from previous models to disaggregate observations that did not entirely fit in one GBD age category or sex. Ratios were determined by dividing the result for a specific age and sex by the result for the aggregate age and sex specified in a given observation.

Low birthweight (<2500 grams) data were extracted from literature, vital registration systems, and surveys. Survey data (most commonly from DHS and MICS) were observed to have high missingness of birthweight responses. We evaluated the patterns of missingness and found a number of distinct patterns that suggested non-random omission of birthweight observations. We therefore imputed

missing birthweight values using the Amelia II (Version 1.7.6) package in R. Birthweight was predicted using the following variables also in the DHS surveys: urbanicity, sex, birthweight recorded on card, birth order, maternal education, paternal education, child age, child weight, child height, mother's age at birth, mother's weight, shared toilet facility, and household water treated.

After imputation, we completed a number of additional steps to standardize the dataset by applying a series of crosswalks. "Crosswalking" is a process of reducing non-random bias by adjusting non-standard data to the likely value had the data been collected using a reference definition, technique, or sample. Three crosswalks were applied for birthweight and gestational age data, all of the statistical models for which were developed using meta-regression – regularized, Bayesian, trimmed (MR-BRT).

First was a crosswalk for method of gestational age assessment that included three separate models. All microdata that reported GA and both obstetric estimate (OE) and last menstrual period were crosswalked to OE using the relationship derived from USA GA microdata (Figure 2). This crosswalk was developed with a spline on LMP in order to reliably match on the data that needed to be crosswalked.

Next, for all data that were only categorical, we adjusted all gestational age data to a reference definition of obstetric estimate (OE), which also included tabulations of the crosswalked microdata above.  Two alternate definitions regularly appeared and both were crosswalked separately. These were Last Menstrual Period (LMP) for each of <37 weeks and <28 weeks gestation (Tables 5 and 6) and other measure of gestation age (Table 7 and 8).

The second set of crosswalks adjusted data derived from clinical administrative sources (ie. Hospital discharges and insurance claims) to matched vital registration data using OE (Tables 9 and 10).

The third set of crosswalks served to "square the input dataset" to ensure that every location-year with data had an observation for each of <2500g (birthweight), <37 weeks, and <28 weeks. This process utilized relationships between input data types to maximize the volume of data later input to models. Low birthweight data (<2500g) were crosswalked to preterm (<37 weeks) data (Table 11), preterm to extremely preterm (Table 12), and extremely preterm to preterm (Table 13).

**Figure 2. MR-BRT OE-LMP crosswalk adjustment factor by LMP-reported gestational age**

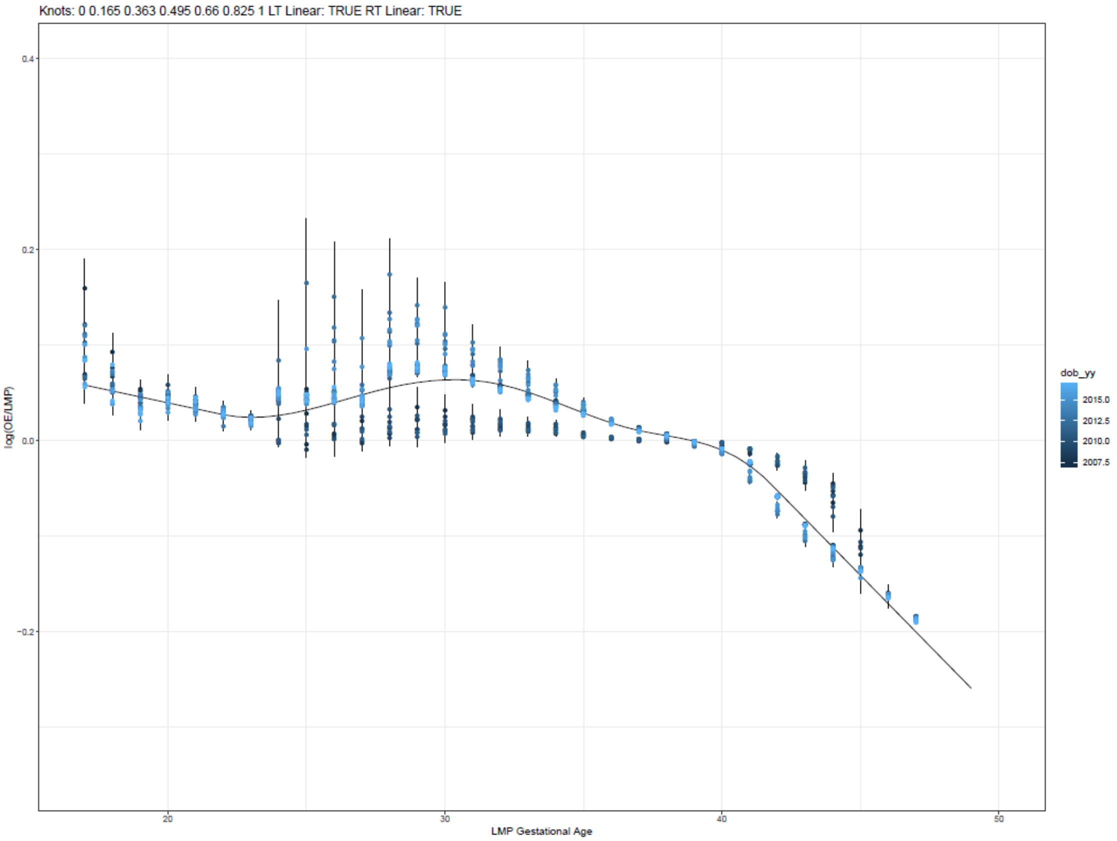


## Table 5. MR-BRT OE-LMP crosswalk adjustment factor for preterm birth (<37 weeks of gestation)

| Data input | Reference or alternative case definition | Gamma | Beta coefficient, log (95% CI) | Adjustment factor* |
|---|---|---|---|---|
| Obstetric estimate | Reference | 0.01 | --- | --- |
| Last menstrual period | Alternative | | 0.187 (0.142,0.231) | 1.205 (1.153, 1.260) |

## Table 6. MR-BRT OE-LMP crosswalk adjustment factor for extremely preterm (<28 weeks gestation)

| Data input | Reference or alternative case definition | Gamma | Beta coefficient, log (95% CI) | Adjustment factor* |
|---|---|---|---|---|
| Obstetric estimate | Reference | 0.00 | --- | --- |
| Last menstrual period | Alternative | | 0.0284 (0.268,0.300) | 1.328 (1.308, 1.349) |

## Table 7. MR-BRT OE-other measure crosswalk adjustment factor for preterm birth (<37 weeks gestation)

| Data input | Reference or alternative case definition | Gamma | Beta coefficient, log (95% CI) | Adjustment factor* |
|---|---|---|---|---|
| Obstetric estimate | Reference | 0.10 | --- | --- |
| Other measurement | Alternative | | -0.243 (--0.494, 0.009) | 0.785 (0.610, 1.01) |

### Table 8. MR-BRT OE-other measure crosswalk adjustment factor for extremely preterm birth (<28 weeks gestation)

| Data input | Reference or alternative case definition | Gamma | Beta coefficient, log (95% CI) | Adjustment factor* |
|---|---|---|---|---|
| Obstetric estimate | Reference | 0.37 | --- | --- |
| Other measurement | Alternative | | 0.154 (-0.486, 0.793) | 1.166 (0.615, 2.210) |

### Table 9. MR-BRT VR-claims crosswalk adjustment factor for preterm birth (<37 weeks gestation)

| Data input | Reference or alternative case definition | Gamma | Beta coefficient, log (95% CI) | Adjustment factor* |
|---|---|---|---|---|
| Vital registration | Reference | 0.07 | --- | --- |
| Insurance claims | Alternative | | -0.712 (-0.909, -0.515) | 0.491 (0.403, 0.597) |

### Table 10. MR-BRT VR-insurance claims crosswalk adjustment factor for extremely preterm birth (<28 weeks of gestation)

| Data input | Reference or alternative case definition | Gamma | Beta coefficient, log (95% CI) | Adjustment factor* |
|---|---|---|---|---|
| Vital registration | Reference | 0.02 | --- | --- |
| Insurance claims | Alternative | | -1.258 (-1.447, -1.07) | 0.284 (0.235, 0.344) |

### Table 11. MR-BRT low birthweight to preterm birth (<37 weeks gestation)

| Data input | Reference or alternative case definition | Gamma | Beta coefficient, log (95% CI) | Adjustment factor* |
|---|---|---|---|---|
| Preterm birth | Reference | 0.08 | --- | --- |
| Low birthweight | Alternative | | -0.479 (-0.518, -0.440) | 0.620 (0.596, 0.644) |

### Table 12. MR-BRT preterm (<37 weeks gestation) to extremely preterm (<28 weeks gestation)

| Data input | Reference or alternative case definition | Gamma | Beta coefficient, log (95% CI) | Adjustment factor* |
|---|---|---|---|---|
| 28 weeks | Reference | 0.06 | --- | --- |
| 37 weeks | Alternative | | 3.221 (3.161, 3.281) | 25.053 (23.600, 26.604) |

**Table 13. MR-BRT extremely preterm (<28 weeks gestation) to preterm (<37 weeks gestation)**

| Data input | Reference or alternative case definition | Gamma | Beta coefficient, log (95% CI) | Adjustment factor* |
|---|---|---|---|---|
| 37 weeks | Reference | 0.05 | --- | --- |
| 28 weeks | Alternative | | -3.208 (-3.266, -3.150) | 0.0404 (0.0381, 0.0428) |

**MR-BRT crosswalk adjustments can be interpreted as the factor the alternative case definition is adjusted by to reflect what it would have been had it been measured using the reference case definition. If the log/logit beta coefficient is negative, then the alternative is adjusted up to the reference. If the log/logit beta coefficient is positive, then the alternative is adjusted down to the reference.*

***The adjustment factor column is the exponentiated beta coefficient. For log beta coefficients, this is the relative rate between the two case definitions. For logit beta coefficients, this is the relative odds between the two case definitions.*

These data adjustments had the effect of dramatically increasing the size of each of the modelling datasets and are primarily responsible for most changes in preterm estimates between GBD 2019 and GBD 2021. After all crosswalks, we performed a deduplication step on GA models. Namely, if low birthweight data in countries that were 1) categorised as "data-rich" locations in cause-of-death modelling or had at least 10 consecutive years of vital registration data recording gestational age, and 2) had both preterm birth and low birthweight data, then crosswalked low birthweight data were outliered so that the model was informed only by the gestational age data.

## Modelling strategy

### *Step A: Model univariate birthweight and gestational age distributions at birth, by l/y/s*

Microdata are the ideal data source for modelling distributions; however, microdata are not widely available for birthweight and are scarcer for gestational age. Categorical prevalence data are more readily available from a wider range of locations and years for low birthweight (<2500g), extremely preterm (<28 weeks of gestation), and preterm birth (<37 weeks of gestation). Because categorical prevalence has wider availability than microdata, we use prevalence data to assist in modelling birthweight and gestational age ensemble distributions.

Ensemble distribution models can be constructed with three pieces of information: mean of the distribution, variance of the distribution, and the weights of the distributions being used in the ensemble. To model mean and variance for all l/y/s for birthweight and gestational age, we first used spatiotemporal Gaussian process regression (ST-GPR) models to model prevalence of low birthweight, extremely preterm, and preterm birth for all l/y/s at birth. To model mean birthweight for all l/y/s, OLS linear regression was used to regress mean birthweight on log-transformed low birthweight prevalence. This model was then used to predict mean birthweight for all l/y/s, using the prevalence of low birthweight (<2500 grams) modelled for all l/y/s in ST-GPR. Similarly, to model gestational age mean for all l/y/s, OLS linear regression model was used to regress mean gestational age on log-transformed preterm prevalence. Mean gestational age for all l/y/s was predicted using the preterm birth (<37 weeks) estimated modelled in ST-GPR.

Global ensemble weights for gestational age were derived by using all available gestational age and birthweight microdata in Table 14 to select the ensemble weights. The distribution families

included in the optimization process were exponential, gamma, gumbel, Weibull, log-normal, normal, mirrored gamma, and mirrored gumbel. As an advancement in GBD 2021, ensemble weights were fit that specifically targeted the fit at 28 weeks and 37 weeks for gestational age and 1500 grams and 2500 grams for low birthweight. In previous GBD cycles the fit of these models had been optimized to reduce error across the entire distribution. Additionally, as an improvement in GBD 2021, this ensemble weight fitting strategy optimized on all microdata sources simultaneously, as opposed to separately.

For each l/y/s, given the mean and ensemble weights, the variance was optimised to minimise error on the prevalence of preterm birth (<37 weeks) for the gestational age distribution and prevalence of low birthweight (<2500 grams) for the birthweight distribution.

*Step B: Model joint birthweight and gestational age distributions at birth, by l/y/s*

In order to model the joint distribution of gestational age and birthweight from separate distributions, information was needed about the correlation between the two distributions. Distributions of gestational age and birthweight are not independent; the Spearman correlation for each country where joint microdata were available (Table 14), pooling across all years of data available, ranged from 0.25 to 0.49. The overall Spearman correlation was 0.38, pooling across all countries in the dataset.

**Table 14. Summary of microdata inputs**

| ***Location*** | **Years of data** | **Total births*** | **Format of data** | **Spearman correlation** | **Used in ensemble weight selection** | **Used in copula parameter selection** | **Used in relative risk models** |
|---|---|---|---|---|---|---|---|
| *BRA* | 2016 | 2,854,380 | Microdata | 0.37 | Yes | Yes | No |
| *ECU* | 2003–2015 | 2,473,039 | Microdata | 0.34 | Yes | Yes | No |
| *ESP* | 1990–2014 | 8,537,220 | Microdata | 0.42 | Yes | Yes | No |
| *JPN* | 1995–2015 | 23,644,506 | Tabulations | 0.41 | No | No | Yes |
| *MEX* | 2008–2012 | 10,256,117 | Microdata | 0.35 | Yes | Yes | No |
| *NOR* | 1990–2014 | 1,489,210 | Microdata | 0.44 | Yes | Yes | Yes |
| *NZL* | 1990–2016 | 1,600,501 | Microdata | 0.25 | Yes | Yes | Yes |
| *SGP* | 1993–2015 | 972,775 | Tabulations | 0.41 | No | No | Yes |
| *TWN* | 1998–2002 | 1,331,760 | Tabulations | 0.38 | No | No | Yes |
| *URY* | 1996–2014 | 698,622 | Microdata | 0.49 | Yes | Yes | No |
| *USA* | 1990–2014 | 81,929,879 | Microdata | 0.38 | Yes | Yes | Yes |

** Pooled across all years and sexes, excluding data missing year of birth, gestational age, or birthweight*

Joint distributions between the birthweight and gestational age marginal distributions were modelled with copulae. The Copula and VineCopula packages in R were used to select the optimal copula family and copula parameters to model the joint distribution, using joint microdata from the country-years in Table 14. The copula family selected from the microdata was “Survival BB8”, with theta parameter set to 1.75 and delta parameter set to 1.

The joint distribution of birthweight and gestational age per location-year-sex was modelled using the global copula family and parameters selected and the location-year-sex gestational

age and birthweight distributions. The joint distribution was simulated 100 times to capture uncertainty. Each simulation consisted of 10,000 simulated joint birthweight and gestational age datapoints. Each joint distribution was divided into 500g by 2-week bins to match the categorical bins of the relative risk surface. Birth prevalence was then calculated for each 500g by 2-week bin.

#### *Step C: Model joint distributions from birth to the end of the neonatal period, by l/y/s*

Early neonatal prevalence and late neonatal prevalence were estimated using life table approaches for each 500g and 2-week bin. Using the all-cause early neonatal mortality rate for each location-year-sex, births per location-year-sex-bin, and the relative risks for each location-year-sex-bin in the early neonatal period, the all-cause early neonatal mortality rate was calculated for each location-year-sex-bin. The early neonatal mortality rate per bin was used to calculate the number of survivors at seven days and prevalence in the early neonatal period. Using the same process, the all-cause late neonatal mortality rate for each location-year-sex was paired with the number of survivors at seven days and late neonatal relative risks per bin to calculate late neonatal prevalence and survivors at 28 days.

## Relative risks and theoretical minimum risk exposure level

LBWSG is paired with the outcomes listed in Table 15 and is only attributed to burden in the early and late neonatal period.

**Table 15: Cause list of outcomes for low birthweight and short gestation**

| Cause name |
|---|
| Diarrhoeal diseases |
| Lower respiratory infections |
| Upper respiratory infections |
| Otitis media |
| Pneumococcal meningitis |
| *H influenzae* type B meningitis |
| Meningococcal meningitis |
| Other meningitis |
| Encephalitis |
| Neonatal preterm birth complications |
| Neonatal encephalopathy due to birth asphyxia and trauma |
| Neonatal sepsis and other neonatal infections |
| Haemolytic disease and other neonatal jaundice |
| Other neonatal disorders |
| Sudden infant death syndrome |

### Causes

The available data for deriving relative risk was only for all-cause mortality. The exception was the USA linked infant birth-death cohort data, which contained three-digit ICD causes of death,

but also had nearly 30% of deaths coded to causes that are ill-defined, or intermediate, in the GBD cause classification system. We analysed the relative risk of all-cause mortality across all available sources and selected outcomes based on criteria of biological plausibility. Some causes, most notably congenital birth defects, haemoglobinopathies, malaria, and HIV/AIDS, were excluded based on the criteria that reverse causality could not be excluded.

### Input data

In the Norway, New Zealand, and USA Linked Birth/Death Cohort microdata datasets, livebirths are reported with gestational age, birthweight, and an indicator of death at 7 days and 28 days. For this analysis, gestational age was grouped into two-week categories, and birthweight was grouped into 500-gram categories. The Taiwan, Japan, and Singapore datasets were prepared in tabulations of joint 500-gram and two-week categories. A pooled country analysis of mortality risk in the early neonatal period and late neonatal period by “small-for-gestational-age” category in developing countries in Asia and sub-Saharan Africa were also used to inform the relative risk analysis.

**Table 16: Input data for relative risk models**

| Input data | Relative risk |
|---|---|
| Source count (total) | 113 |
| Number of countries with data | 6 |

### Modelling strategy

For each location, data were pooled across years, and the risk of all-cause mortality at the early neonatal period and late neonatal period at joint birthweight and gestational age combinations was calculated. In all datasets except for the USA, sex-specific data were combined to maximise sample size. The USA analyses were sex-specific. To calculate relative risk at each 500-gram and two-week combination, logistic regression was first used to calculate mortality odds for each joint two-week gestational age and 500-gram birthweight category. Mortality odds were smoothed with Gaussian process regression, with the independent distributions of mortality odds by birthweight and mortality odds by gestational age serving as priors in the regression.

A pooled country analysis of mortality risk in the early neonatal period and late neonatal period by SGA category in developing countries in Asia and sub-Saharan Africa were also converted into 500-gram and two-week bin mortality odds surfaces. The relative risk surfaces produced from microdata and the Asia and Africa surfaces produced from the pooled country analysis were meta-analysed, resulting in a meta-analysed mortality odds surface for each location. The meta-analysed mortality odds surface for each location was smoothed using Gaussian process regression and then converted into mortality risk. To calculate mortality relative risks, the risk of each joint two-week gestational age and 500-gram birthweight category were divided by the

risk of mortality in the joint gestational age and birthweight category with the lowest mortality risk.

For each of the country-derived relative risk surfaces, the 500-gram and two-week gestational age joint bin with the lowest risk was identified. This bin differed within each country dataset. To identify the universal 500-gram and two-week gestational age category that would serve as the universal TMREL for our analysis, we chose the bins that was identified to be the TMREL in each country dataset to contribute to the universal TMREL. Therefore, the joint categories that served as our universal TMREL for the LBWSG risk factor were “38–40 weeks of gestation and 3500–4000 grams”, “38–40 weeks of gestation and 4000–4500 grams”, and “40–42 weeks of gestation and 4000–4500 grams”. As the joint TMREL, all three categories were assigned to a relative risk equal to 1.

### Population attributable fraction

The total PAF for the low birthweight and short gestation joint risk factor was calculated by summing the PAF calculated from each 500g x two-week category, with the lowest risk category among all the 500g x two-week categories serving as the TMREL. The equation for calculating PAF for each 500g x two-week category is:

$$PAF_{joasgt} = \frac{\sum_{x=1}^{u} RR_{joast}(x)P_{jasgt}(x) - RR_{joasg}(TMRE_{jas})}{\sum_{x=1}^{u} RR_{joas}(x)P_{jasgt}(x)}$$

To calculate the PAFs for the univariate risks (‘short gestation for birthweight’ and ‘low birthweight for gestation’), relative risks are first weighted by global exposure in 2019, summed across one of the dimensions (gestational age or birthweight), and then rescaled by the maximum relative risk in the TMREL block (38-42 weeks of gestation and 3500-4500 grams). Any relative risk less than 1 was set to 1. Exposure was also summed across the same dimension, and the univariate PAF equalled the sum of the product of the weighted relative risks and exposures.

## Low Birthweight and Short Gestation References


1. Katz J, Lee AC, Kozuki N, Lawn JE, Cousens S, Blencowe H, et al. Mortality risk in preterm and small-for-gestational-age infants in low-income and middle-income countries: a pooled country analysis. The Lancet. 2013;382(9890):417–25.

# Suboptimal breastfeeding

## Flowchart

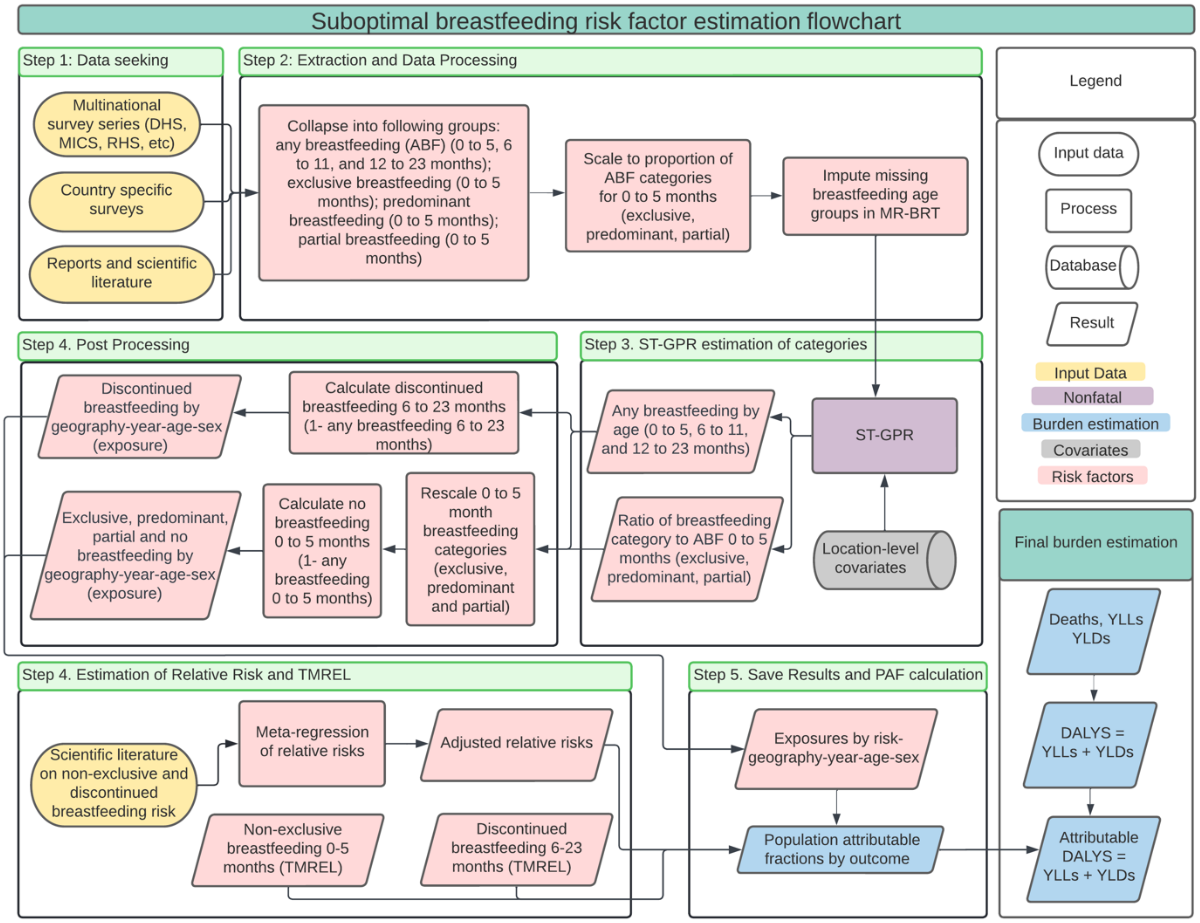


## Input data and methodological summary

## Definition

### Exposure

Exposure to suboptimal breastfeeding is composed of two distinct categories: non-exclusive breastfeeding and discontinued breastfeeding.

**Non-exclusive breastfeeding** is defined as the proportion of children under 6 months of age who are not exclusively breastfed. We then parse those not exclusively breastfed into three categories – predominant, partial, and no breastfeeding. Exclusive breastfeeding is defined as the proportion of children who receive no other food or drink except breastmilk (allowing for ORS, drops, or syrups containing vitamins, minerals, or medicines). Predominant breastfeeding is the proportion of children whose predominant source of nourishment is breastmilk but also receive other liquids. Partial breastfeeding refers to those infants who receive breastmilk as

well as food and liquids, including non-human milk and formula. No breastfeeding refers to infants who do not receive breastmilk as a source of nourishment.

**Discontinued breastfeeding** is defined as the proportion of children between 6 and 23 months who receive no breastmilk as a source of nourishment.

## Input data

### Exposure

The data used in the analysis consist mostly of processed individual-level microdata from surveys; in the cases where microdata were unavailable, we used reported tabulated data from survey reports and scientific literature. Data used to categorise type of non-exclusive breastfeeding (predominant, partial, and none) come from surveys with 24-hour dietary logs based on maternal recall.

We updated our systematic review in GBD 2021 by searching the Global Health Data Exchange (GHDx) using the keyword "breastfeeding." We prioritised extraction of surveys with microdata and new surveys from major survey series such as Demographic and Health Surveys (DHS) and Multiple Indicator Cluster Surveys (MICS).

Table 1. Input data counts – suboptimal breastfeeding exposure

| | Countries with data | New sources | Total sources |
|---|---|---|---|
| Non-exclusive breastfeeding | 169 | 49 | 737 |
| Discontinued breastfeeding | 162 | 50 | 679 |

To better ensure consistency in estimates across age groups, we identified location-years where we had data for "any breastfeeding 6–11 months" but no data for "any breastfeeding 12–23 months." We then imputed data for "any breastfeeding 12–23 months" based on the observed 6–11 month datapoint in that location-year. We estimated the imputation adjustment by meta-analysing proportion ratios of matched pairs by source-location-year for any breastfeeding in these two age groups in meta-regression—Bayesian, regularised, trimmed (MR-BRT)[1], a Bayesian meta-analytic tool.

Table 2. MR-BRT adjustment factor for any breastfeeding 12–23 months imputation

| Data input | Reference or alternative definition | Gamma | Beta coefficient, logit (95% UI)* | Adjustment factor** |
|---|---|---|---|---|
| Any breastfeeding 6–11 months | Ref | 0.19 | --- | --- |

| | | | | |
|---|---|---|---|---|
| Any breastfeeding 12–23 months | Alt | | -1.54 (-1.58 – -1.50) | 0.21 (0.21–0.22) |

**MR-BRT crosswalk adjustments can be interpreted as the factor the alternative definition is adjusted by to reflect what it would have been had it been measured using the reference definition.*
***The adjustment factor column is the exponentiated beta coefficient. For logit beta coefficients, this is the relative odds between the two definitions.*

### Relative risk

We included outcomes based on the strength of available evidence supporting a causal relationship. Studies evaluating the causal evidence for our risk-outcome pairs came primarily from studies compiled in a published review by the World Health Organization.[2] Non-exclusive breastfeeding was paired with diarrhoea and lower respiratory infection as diseases outcomes. Discontinued breastfeeding was paired with diarrhoea as an outcome.

Table 3. Input data counts – suboptimal breastfeeding relative risk

| | **Countries with data** | **New sources** | **Total sources** |
|---|---|---|---|
| Relative risk | 26 | 0 | 43 |

## Modelling strategy

### Exposure

Using the processed microdata and tabulated data from reports, we generated a complete time series from 1980 to 2022 for 1) any breastfeeding 0–5 months, 6–11 months, and 12–23 months, 2) ratio of exclusive breastfeeding to any breastfeeding 0–5 months, 3) ratio of predominant breastfeeding to any breastfeeding 0–5 months, and 4) ratio of partial breastfeeding to any breastfeeding 0–5 months using a three-step spatiotemporal Gaussian process regression. In previous GBD rounds, "any breastfeeding" was modelled separately for each of the estimated age groups. In GBD 2021 with the addition of new under-5 age groups that aligned with those ages we model, we incorporated the three age groups into a single model of "any breastfeeding." This allowed us to borrow additional strength over space, age, and time by incorporating data from all sources in one model.

The first step of the ST-GPR process is an ensemble linear mixed-effects regression of our data on a set of potentially predictive covariates taken from the GBD Study covariates database. We tested every combination of these covariates in individual, sex-specific mixed-effects linear regressions with nested random effects at the super-region, region, and location levels. We then evaluated and ranked each of these sub-models by their out-of-sample root-mean-squared error. Finally, to produce initial estimates for every location, year, age, and sex in the analysis, we averaged the 50 top-performing models where the estimated coefficients were 1) statistically significant at $p < 0.05$ and 2) in the expected direction. We tested the following

covariates in the ensemble prior: Socio-demographic Index, SEV unsafe water, total fertility rate, maternal education, antenatal care (4+ visits), HIV mortality in women of reproductive age, high BMI in women of reproductive age, and underweight in women of reproductive age.

The second, spatiotemporal smoothing step of ST-GPR calculates the residual between our stage 1 regression estimate and each of our observed datapoints and then smooths this residual, drawing strength over space, age, and time and producing a revised stage 2 estimate of birth prevalence for every location, year, and sex. The third step of ST-GPR is a Gaussian process regression, using the stage 2 estimates as a prior and the observed datapoints and their variance to 1) further smooth the residual between the stage 2 predictions and observed data and produce a final mean estimate for each location, year, and sex, and 2) estimate uncertainty around this mean estimate, quantified by taking 1000 draws from the posterior Gaussian process. More detailed information on the ST-GPR modelling process can be found in the main text methods appendix.

To generate exposure categories for non-exclusive breastfeeding, we converted the modelled ratios of exclusive, predominant, and partial breastfeeding to the total category prevalence by multiplying each ratio by the estimates of any breastfeeding among infants aged 0–5 months. This ensured that these categories sum correctly to the "any breastfeeding 0–5 months" envelope. We calculated the proportion of infants receiving no breastmilk 0–5 months of age by subtracting the estimates of current breastfeeding from 1. We performed the same operation to estimate discontinued breastfeeding in the 6–11 months and 12–23 months categories.

### Theoretical minimum-risk exposure level

For non-exclusive breastfeeding, those children that received no source of nourishment other than breastmilk ("exclusively breastfed") were considered to be at the lowest risk of any of the disease outcomes. For discontinued breastfeeding, we assumed that children aged 6–23 months who received any breastmilk as a source of nourishment to be at the lowest risk of disease outcome.

### Relative risk

We estimated relative risks for both non-exclusive and discontinued breastfeeding in a meta-analysis using the "metareg" package in Stata. For the 0–5 month age group we included diarrhoea and lower respiratory infection as outcomes, and for the 6–23 month age group we included diarrhoea as an outcome. We did not estimate separate relative risks for morbidity and mortality. The estimated relative risks are detailed in Table 4.

Table 4. Suboptimal breastfeeding relative risk estimates

| **Exposure category** | **Diarrhoea** | | **Lower respiratory infection** | |
|---|---|---|---|---|
| | **Mortality** | **Morbidity** | **Mortality** | **Morbidity** |
| ***0–5 months*** | | | | |
| Exclusive breastfeeding | 1.00 | 1.00 | 1.00 | 1.00 |

| | | | | |
|---|---|---|---|---|
| Predominant breastfeeding | 2.35 (1.67–3.23) | 2.35 (1.67–3.23) | 1.37 (1.06–1.80) | 1.37 (1.06–1.80) |
| Partial breastfeeding | 2.63 (1.94–3.48) | 2.63 (1.94–3.48) | 1.48 (1.21–1.79) | 1.48 (1.21–1.79) |
| No breastfeeding | 3.60 (2.72–4.70) | 3.60 (2.72–4.70) | 1.74 (1.49–2.03) | 1.74 (1.49–2.03) |
| | | | | |
| ***6–23 months*** | | | | |
| Any breastfeeding | 1.00 | 1.00 | -- | -- |
| Discontinued breastfeeding | 1.31 (1.11–1.55) | 1.31 (1.11–1.55) | -- | -- |

## Population attributable fraction

We used the standard GBD population attributable fraction (PAF) equation to calculate PAFs for non-exclusive breastfeeding and discontinued breastfeeding and each of their paired outcomes using exposure estimates, the theoretical minimum-risk exposure level, and relative risks.

## Suboptimal Breastfeeding References


1. Vos T, Lim SS, Abbafati C, et al. Global burden of 369 diseases and injuries in 204 countries and territories, 1990–2019: a systematic analysis for the Global Burden of Disease Study 2019. The Lancet 2020; 396: 1204–22.
2. Horta, B., Voctora, C. (2013) Short-term effects of breastfeeding: a systematic review on the benefits of breastfeeding on diarrhoea and pneumonia mortality. The World Health Organization.

## Justification for the counterfactual approach

There are two main approaches for the causal attribution of health outcomes: categorical attribution and counterfactual analysis. Categorical attribution assigns a single cause (e.g., a disease or a risk factor) or a group of causes to an event (e.g., death). Counterfactual analysis, on the other hand, estimates the impact of causes or risk factors by comparing the observed disease burden with what it would have been under an alternative hypothetical scenario, including scenarios where the risk factors are absent or reduced. This approach, which includes using a theoretical minimum risk exposure distribution to show potential health gains from reducing risk, can attribute the burden of disease to risk factors more comprehensively than categorical attribution, which might not account for the multifactorial nature of many diseases.

## Counterfactual Approach References


1. Ezzati M, Hoorn SV, Lopez AD, et al. Comparative Quantification of Mortality and Burden of Disease Attributable to Selected Risk Factors. In: Lopez AD, Mathers CD, Ezzati M, et al., editors. Global Burden of Disease and Risk Factors. Washington (DC): The International Bank for Reconstruction and Development / The World Bank; 2006. Chapter 4. Available from: https://www.ncbi.nlm.nih.gov/books/NBK11813/ Co-published by Oxford University Press, New York.
2. Mathers, C. D., M. Ezzati, A. D. Lopez, C. J. L. Murray, and A. Rodgers. 2002. "Causal Decomposition of Summary Measures of Population Health." In Summary Measures of Population Health: Concepts, Ethics, Measurement, and Applications, ed. C. J. L. Murray, J. Salomon, C. D. Mathers, and A. D. Lopez. 273–290. Geneva: World Health Organization.


## Estimation of population attributable fractions[3]

Risks are categorised on the basis of how exposure was measured: dichotomous, polytomous, and continuous. High low-density lipoprotein (LDL) cholesterol level is an example of a risk measured on a continuous scale. The PAF, which represents the proportion of risk that would be reduced in a given year if the exposure to a risk factor in the past were reduced to an ideal exposure scenario, is defined for a continuous risk factor as:[19]

$$PAF_{joasgt} = \frac{\int_{x=l}^{u} RR_{joasg}(x)P_{jasgt}(x)dx - RR_{joasg}(TMREL_{jas})}{\int_{x=l}^{u} RR_{joasg}(x)P_{jasgt}(x)dx}$$

Where $PAF_{joasgt}$ is the PAF for cause $o$ due to risk factor $j$ for age group $a$, sex $s$, location $g$, and year $t$. $RR_{joasg}(x)$ is the RR as a function of exposure level $x$ for risk factor $j$ for cause $o$, age group $a$, sex $s$, and location $g$ with the lowest level of observed exposure as $l$ and the highest as $u$; $P_{jasgt}(x)$ is the distribution of exposure at $x$ for age group $a$, sex $s$, location $g$, and year $t$; and $TMREL_{jas}$ is the TMREL for risk factor *j*, age group *a*, and sex *s*.

The $PAF_{joasgt}$ for dichotomous and polytomous risk factors for every country is defined as:

$$PAF_{joasgt} = \frac{\sum_{x=l}^{u} RR_{joasg}(x)P_{jasgt}(x) \; - \; RR_{joasg}(TMREL_{jas})}{\sum_{x=l}^{u} RR_{joasg}(x)P_{jasgt}(x)}$$

where $PAF_{joasgt}$ is the PAF for cause $o$ due to risk factor $j$ for age group $a$, sex $s$, location $g$, and year $t$. $RR_{joasg}(x)$ is the RR as a function of exposure level $x$ for risk factor $j$ for cause $o$, age group $a$, sex $s$, and location $g$ on a plausible range of exposure levels from *l* to *u*; $P_{jasgt}(x)$ is the proportion of the population in risk group (prevalence) for age group $a$, sex $s$, location $g$, and year $t$; and $TMREL_{jas}$ is the TMREL for risk factor *j*, age group *a*, and sex *s*.

## Calculating the burden of multiple risk factors[3]

A multiplicative aggregation of the PAFs of the individual risk factors was computed using the formula below:

$$PAF_{1..i} = 1 - \prod_{i=1}^{n}(1 - PAF_i)$$

where *PAF* is the population attributable fraction and *i* is each individual risk factor.

## Risk factors and causal pathways leading to diarrhoeal diseases

| Risk factor | Causal pathway that leads to diarrheal diseases |
|---|---|
| Unsafe water, unsafe sanitation, and no access to handwashing facility | Enteric pathogens enter the human gastrointestinal tract through contaminated drinking water, inadequate sanitation (e.g., unsafe disposal of human excreta), and poor hygiene practices (e.g., failing to wash one's hands after using the toilet).[1] These pathogens can cause diarrhea by producing enterotoxins that induce increased secretion and by causing inflammation that decreases absorption in the intestines.[2,3] |
| Child growth failure (underweight, stunting, wasting) | Malnutrition can predispose the host to infections, including those caused by enteric pathogens, by impairing the body's defense mechanisms and enabling their colonization of the intestine.[4] |
| Low birthweight and short gestation | The gastrointestinal tract's mucosal surfaces are underdeveloped, weakening the initial line of defense against enteric pathogens[5] |
| Suboptimal breastfeeding | Suboptimal breastfeeding can increase the risk of diarrhea through the following mechanisms[6]: (1) human milk glycans, including oligosaccharides in their free and conjugated forms, are critical for the natural immune defense mechanism protecting breastfed infants from diarrheal diseases, and suboptimal breastfeeding can diminish this benefit; and (2) while breastfeeding reduces exposure to contaminated foods and fluids, suboptimal breastfeeding can increase exposure to them, thereby elevating the risk of developing diarrheal diseases in infants. |
| Vitamin A deficiency and zinc deficiency* | Vitamin A deficiency disrupts the integrity of the intestinal mucosa and alters immune responses, leading to increased susceptibility to infections and inflammation.[7] Similarly, zinc deficiency impairs the body's resistance to enteric pathogens and disrupts water and electrolyte absorption.[8] |
| Particulate matter pollution* | Particulate matter pollution may alter the gut microbiome and immune function, impairing the intestinal barrier.[9] |

* The results for each individual risk factor are not displayed due to their small attribution; however, they are accessible for viewing at https://vizhub.healthdata.org/gbd-compare/.

### Risk Factors and Causal Pathways: References


1. Baker KK, O'Reilly CE, Levine MM, et al. Sanitation and Hygiene-Specific Risk Factors for Moderate-to-Severe Diarrhea in Young Children in the Global Enteric Multicenter Study, 2007-2011: Case-Control Study. PLoS Med 2016; 13(5): e1002010.

2. Satchell KJ. Activation and suppression of the proinflammatory immune response by Vibrio cholerae toxins. Microbes Infect 2003; 5(13): 1241-7.

3. Hodges K, Gill R. Infectious diarrhea: Cellular and molecular mechanisms. Gut Microbes 2010; 1(1): 4-21.

4. Patwari AK. Diarrhoea and malnutrition interaction. Indian J Pediatr 1999; 66(1 Suppl): S124-34.

5. Ghattas H. INFECTION | Nutritional Interactions. In: Caballero B, ed. Encyclopedia of Human Nutrition (Second Edition). Oxford: Elsevier; 2005: 47-54.

6. Lamberti LM, Fischer Walker CL, Noiman A, Victora C, Black RE. Breastfeeding and the risk for diarrhea morbidity and mortality. BMC Public Health 2011; 11(3): S15.

7. Amimo JO, Michael H, Chepngeno J, Raev SA, Saif LJ, Vlasova AN. Immune Impairment Associated with Vitamin A Deficiency: Insights from Clinical Studies and Animal Model Research. Nutrients. 2022;14(23):5038.

8. Wapnir RA. Zinc deficiency, malnutrition and the gastrointestinal tract. The Journal of nutrition. 2000 May 1;130(5):1388S-92S.

9. Salim SY, Kaplan GG, Madsen KL. Air pollution effects on the gut microbiota: a link between exposure and inflammatory disease. Gut Microbes. 2014 Mar-Apr;5(2):215-9.

## Statement of GATHER Compliance

*Appendix Table 9. Checklist of information that should be included in reports of global health estimates, with description of compliance and location of information the current study*

| # | GATHER checklist item | Description of compliance | Reference |
|---|---|---|---|
| **Objectives and funding** | | | |
| **1** | Define the indicator(s), populations (including age, sex, and geographic entities), and time period(s) for which estimates were made. | Narrative provided in paper and appendix describing indicators, definitions, populations, and time periods | Main text (Methods) and Appendix (Methods) |
| **2** | List the funding sources for the work. | Funding sources listed in paper | Summary (Funding) |
| **Data Inputs** | | | |
| *For all data inputs from multiple sources that are synthesized as part of the study:* | | | |
| **3** | Describe how the data were identified and how the data were accessed. | Narrative description of data seeking methods provided | Main text (Methods) and Appendix (Methods) |
| **4** | Specify the inclusion and exclusion criteria. Identify all ad-hoc exclusions. | Narrative about inclusion and exclusion criteria provided; ad hoc exclusions in appendix supplementary methods | Main text (Methods) and Appendix (Methods) |
| **5** | Provide information on all included data sources and their main characteristics. For each data source used, report reference information or contact name/institution, population represented, data collection method, year(s) of data collection, sex and age range, diagnostic criteria or measurement method, and sample size, as relevant. | An interactive, online data source tool that provides metadata for data sources by component, geography, cause, risk, or impairment has been developed, and data source citations provided | Appendix (Methods) with additional information about these sources available at https://ghdx.healthdata.org/gbd-2021 |
| **6** | Identify and describe any categories of input data that have potentially important biases (e.g., based on characteristics listed in item 5). | Summary of known biases included in appendix supplementary methods | Appendix (Methods) |
| *For data inputs that contribute to the analysis but were not synthesized as part of the study:* | | | |

| | | | |
|---|---|---|---|
| **7** | Describe and give sources for any other data inputs. | Included in online data source tool | Global Health Data Exchange https://ghdx.healthdata.org/gbd-2021 |
| *For all data inputs:* | | | |
| **8** | Provide all data inputs in a file format from which data can be efficiently extracted (e.g., a spreadsheet rather than a PDF), including all relevant meta-data listed in item 5. For any data inputs that cannot be shared because of ethical or legal reasons, such as third-party ownership, provide a contact name or the name of the institution that retains the right to the data. | Downloads of input data available through online data tools; input data not available in tools will be made available upon request | Global Health Data Exchange https://ghdx.healthdata.org/gbd-2021 |
| **Data analysis** | | | |
| **9** | Provide a conceptual overview of the data analysis method. A diagram may be helpful. | Flow diagram of methodological process provided, as well as narrative descriptions of modelling process | Main text (Methods) and Appendix (Methods) |
| **10** | Provide a detailed description of all steps of the analysis, including mathematical formulae. This description should cover, as relevant, data cleaning, data pre-processing, data adjustments and weighting of data sources, and mathematical or statistical model(s). | Flow diagram and detailed methods write-up covering all data extraction, processing, and modelling processes provided | Main text (Methods) and Appendix (Methods) |
| **11** | Describe how candidate models were evaluated and how the final model(s) were selected. | Provided in methodological write-up | Appendix (Methods) |
| **12** | Provide the results of an evaluation of model performance, if done, as well as the results of any relevant sensitivity analysis. | Provided in methodological write-up | Appendix (Methods) |
| **13** | Describe methods for calculating uncertainty of the estimates. State which sources of uncertainty were, and were not, accounted for in the uncertainty analysis. | Provided in main text methods narrative description and appendix methodological writeup | Main text (Methods) and Appendix (Methods) |
| **14** | State how analytic or statistical source code used to generate estimates can be accessed. | Remote code repository for access to analytic code provided | Remote code repository https://ghdx.healthdata.org/gbd-2021/code |
| **Results and Discussion** | | | |
| **15** | Provide published estimates in a file format from which data can be efficiently extracted. | Tables in appendices and online results tool | Appendix Results and https://ghdx.healthdata.org/gbd-2021 |
| **16** | Report a quantitative measure of the uncertainty of the estimates (e.g. uncertainty intervals). | Uncertainty provided with all results | Main text (Results), Appendix Results |
| **17** | Interpret results in light of existing evidence. If updating a previous set of estimates, describe the reasons for changes in estimates. | Discussion of results and methodological changes between GBD rounds provided in manuscript narrative and appendix | Main text (Methods, Results and Discussion) and Appendix (Methods) |
| **18** | Discuss limitations of the estimates. Include a discussion of any modelling assumptions or data limitations that affect interpretation of the estimates. | Discussion of limitations, including modelling assumptions and data limitations, included in manuscript narrative and appendix | Main text (Methods and Discussion) and Appendix (Methods) |

Note: A full set of granular estimates can be found in the GBD Results Tool here, https://vizhub.healthdata.org/gbd-results/

## Ethics Statement

The Global Burden of Diseases, Injuries, and Risk Factors Study used de-identified data, and the waiver of informed consent was reviewed and approved by the University of Washington Institutional Review Board (study number 9060).

# Authorship Material

## GBD 2021 Diarrhoeal Disease Collaborators

Hmwe Hmwe Kyu, Avina Vongpradith, Regina-Mae Villanueva Dominguez, Jianing Ma, Samuel B Albertson, Amanda Novotney, Ibrahim A Khalil, Christopher E Troeger, Matthew C Doxey, Jorge R Ledesma, Sarah Brooke Sirota, Rose Grace Bender, Lucien R Swetschinski, Matthew Cunningham, Sandra Spearman, Yohannes Habtegiorgis Abate, Abdallah H A Abd Al Magied, Samar Abd ElHafeez, Meriem Abdoun, Bayeh Abera, Hassan Abidi, Richard Gyan Aboagye, Yonas Derso Abtew, Hasan Abualruz, Eman Abu-Gharbieh, Hana J Abukhadijah, Salahdein Aburuz, Isaac Yeboah Addo, Victor Adekanmbi, Charles Oluwaseun Oluwaseun Adetunji, Temitayo Esther Adeyeoluwa, Ripon Kumar Adhikary, Qorinah Estiningtyas Sakilah Adnani, Saryia Adra, Leticia Akua Adzigbli, Aanuoluwapo Adeyimika Afolabi, Muhammad Sohail Afzal, Saira Afzal, Suneth Buddhika Agampodi, Feleke Doyore Agide, Bright Opoku Ahinkorah, Aqeel Ahmad, Sajjad Ahmad, Ali Ahmed, Ayman Ahmed, Haroon Ahmed, Saeed Ahmed, Karolina Akinosoglou, Ema Akter, Salah Al Awaidy, Muaaz M Alajlani, Khurshid Alam, Almaza Albakri, Mohammed Albashtawy, Wafa A Aldhaleei, Abdelazeem M Algammal, Adel Ali Saeed Al-Gheethi, Abid Ali, Syed Shujait Ali, Waad Ali, Sheikh Mohammad Alif, Syed Mohamed Aljunid, Sabah Al-Marwani, Joseph Uy Almazan, Hesham M Al-Mekhlafi, Sami Almustanyir, Saleh A Alqahatni, Ahmad Alrawashdeh, Rami H Al-Rifai, Mohammed A Alsabri, Awais Altaf, Khalid A Altirkawi, Nelson Alvis-Guzman, Nelson J Alvis-Zakzuk, Mohammad Sharif Ibrahim Alyahya, Walid A Al-Zyoud, Dickson A Amugsi, Catalina Liliana Andrei, Sebastien Antoni, Boluwatife Stephen Anuoluwa, Iyadunni Adesola Anuoluwa, Saleha Anwar, Palwasha Anwari, Geminn Louis Carace Apostol, Jalal Arabloo, Mosab Arafat, Aleksandr Y Aravkin, Demelash Areda, Brhane Berhe Aregawi, Abdulfatai Aremu, Michael Benjamin Arndt, Akeza Awealom Asgedom, Tahira Ashraf, Seyyed Shamsadin Athari, Alok Atreya, Firayad Ayele, Davood Azadi, Gulrez Shah Azhar, Shahkaar Aziz, Ahmed Y. Azzam, Giridhara Rathnaiah Babu, Pegah Bahrami Taghanaki, Saeed Bahramian, Senthilkumar Balakrishnan, Biswajit Banik, Simachew Animen Bante, Mainak Bardhan, Till Winfried Bärnighausen, Hiba Jawdat Barqawi, Amadou Barrow, Zarrin Basharat, Quique Bassat, Mohammad-Mahdi Bastan, Saurav Basu, Prapthi Persis Bathini, Payam Behzadi, Maryam Beiranvand, Muhammad Bashir Bello, Olorunjuwon Omolaja Bello, Apostolos Beloukas, Azizullah Beran, Dinesh Bhandari, Pankaj Bhardwaj, Zulfiqar A Bhutta, Hamed Borhany, Souad Bouaoud, Michael Brauer, Danilo Buonsenso, Zahid A Butt, Mehtap Çakmak Barsbay, Luis Alberto Cámera, Angelo Capodici, Carlos A Castañeda-Orjuela, Muthia Cenderadewi, Chiranjib Chakraborty, Sandip Chakraborty, Vijay Kumar Chattu, Anis Ahmad Chaudhary, Fatemeh Chichagi, Patrick R Ching, Jesus Lorenzo Chirinos-Caceres, Hitesh Chopra, Sonali Gajanan Choudhari, Enayet Karim Chowdhury, Dinh-Toi Chu, Isaac Sunday Chukwu, Muhammad Chutiyami, Natalia Cruz-Martins, Omid Dadras, Xiaochen Dai, Lalit Dandona, Rakhi Dandona, Samuel Demissie Darcho, Jai K Das, Nihar Ranjan Dash, Ivan Delgado-Enciso, Belay Desye, Vinoth Gnana Chellaiyan Devanbu, Kuldeep Dhama, Meghnath Dhimal, Michael J Diaz, Thanh Chi Do, Sushil Dohare, Fariba Dorostkar, Ojas Prakashbhai Doshi, Leila Doshmangir, Haneil Larson Dsouza, Senbagam Duraisamy, Oyewole Christopher Durojaiye, Abdel Rahman E'mar, Abdelaziz Ed-Dra, Hisham Atan Edinur, Defi Efendi, Ferry Efendi, Foolad Eghbali, Temitope Cyrus Ekundayo, Iman El Sayed, Muhammed Elhadi, Ashraf A El-Metwally, Mohammed Elshaer, Ibrahim Elsohaby, Chadi Eltaha, Babak Eshrati, Majid Eslami, Ayesha Fahim, Ildar Ravisovich Fakhradiyev, Aliasghar Fakhri-Demeshghieh, Mohammad Farahmand, Folorunso Oludayo Fasina, Modupe Margaret Fasina, Alireza Feizkhah, Ginenus Fekadu, Nuno Ferreira, Getahun Fetensa, Florian Fischer, Takeshi Fukumoto, Blima Fux, Muktar A Gadanya, Santosh Gaihre, Márió Gajdács, Yaseen Galali, Aravind P Gandhi, Rupesh K Gautam, Miglas Welay Gebregergis, Mesfin Gebrehiwot, Teferi Gebru Gebremeskel, Motuma Erena Getachew, Genanew

K Getahun, Molla Getie, Afsaneh Ghasemzadeh, Ramy Mohamed Ghazy, Sherief Ghozy, Artyom Urievich Gil, Alem Abera Girmay, Abraham Tamirat T Gizaw, Mahaveer Golechha, Pouya Goleij, Philimon N Gona, Ayman Grada, Giovanni Guarducci, Mesay Dechasa Gudeta, Vivek Kumar Gupta, Awoke Derbie Habteyohannes, Najah R Hadi, Samer Hamidi, Erin B Hamilton, Harapan Harapan, Md. Kamrul Hasan, S. M. Mahmudul Hasan, Hamidreza Hasani, Md Saquib Hasnain, Ikrama Ibrahim Hassan, Jiawei He, Mehdi Hemmati, Kamal Hezam, Mehdi Hosseinzadeh, Junjie Huang, Hong-Han Huynh, Segun Emmanuel Ibitoye, Kevin S Ikuta, Olayinka Stephen Ilesanmi, Irena M Ilic, Milena D Ilic, Sumant Inamdar, Mustafa Alhaji Isa, Md. Rabiul Islam, Sheikh Mohammed Shariful Islam, Nahlah Elkudssiah Ismail, Chidozie Declan Iwu, Kathryn H Jacobsen, Haitham Jahrami, Akhil Jain, Nityanand Jain, Ammar Abdulrahman Jairoun, Mihajlo Jakovljevic, Reza Jalilzadeh Yengejeh, Javad Javidnia, Shubha Jayaram, Mohammad Jokar, Jost B Jonas, Abel Joseph, Nitin Joseph, Jacek Jerzy Jozwiak, Hannaneh Kabir, Dler H. Hussein Kadir, Md Moustafa Kamal, Vineet Kumar Kamal, Arun Kamireddy, Tanuj Kanchan, Kehinde Kazeem Kanmodi, Suthanthira Kannan S, Rami S Kantar, Jafar Karami, Prabin Karki, Hengameh Kasraei, Harkiran Kaur, Mohammad Keykhaei, Yousef Saleh Khader, Alireza Khalilian, Faham Khamesipour, Gulfaraz Khan, Mohammad Jobair Khan, Zeeshan Ali Khan, Vishnu Khanal, Khaled Khatab, Moawiah Mohammad Khatatbeh, Amir M Khater, Khalid A Kheirallah, Feriha Fatima Khidri, Atulya Aman Khosla, Kwanghyun Kim, Yun Jin Kim, Adnan Kisa, Niranjan Kissoon, Desmond Klu, Sonali Kochhar, Ali-Asghar Kolahi, Farzad Kompani, Soewarta Kosen, Kewal Krishan, Barthelemy Kuate Defo, Md Abdul Kuddus, Mohammed Kuddus, Mukhtar Kulimbet, G Anil Kumar, Rakesh Kumar, Frank Kyei-Arthur, Chandrakant Lahariya, Dharmesh Kumar Lal, Nhi Huu Hanh Le, Seung Won Lee, Wei-Chen Lee, Yeong Yeh Lee, Ming-Chieh Li, Virendra S Ligade, Gang Liu, Shuke Liu, Xiaofeng Liu, Xuefeng Liu, Chun-Han Lo, Giancarlo Lucchetti, Lei Lv, Kashish Malhotra, Ahmad Azam Malik, Bishnu P Marasini, Miquel Martorell, Roy Rillera Marzo, Hossein Masoumi-Asl, Medha Mathur, Navgeet Mathur, Rishi P Mediratta, Elahe Meftah, Tesfahun Mekene Meto, Hadush Negash Meles, Endalkachew Belayneh Melese, Walter Mendoza, Mohsen Merati, Tuomo J Meretoja, Tomislav Mestrovic, Sachith Mettananda, Le Huu Nhat Minh, Vinaytosh Mishra, Prasanna Mithra, Ashraf Mohamadkhani, Ahmed Ismail Mohamed, Mouhand F H Mohamed, Nouh Saad Mohamed, Mustapha Mohammed, Shafiu Mohammed, Lorenzo Monasta, Mohammad Ali Moni, Rohith Motappa, Vincent Mougin, Sumaira Mubarik, Francesk Mulita, Kavita Munjal, Yanjinlkham Munkhsaikhan, Pirouz Naghavi, Gurudatta Naik, Tapas Sadasivan Nair, Hastyar Hama Rashid Najmuldeen, Shumaila Nargus, Delaram Narimani Davani, Abdulqadir J Nashwan, Zuhair S Natto, Athare Nazri-Panjaki, G Takop Nchanji, Pacifique Ndishimye, Josephine W Ngunjiri, Duc Hoang Nguyen, Nhien Ngoc Y Nguyen, Van Thanh Nguyen, Yeshambel T Nigatu, Ali Nikoobar, Vikram Niranjan, Chukwudi A Nnaji, Efaq Ali Noman, Nurulamin M Noor, Syed Toukir Ahmed Noor, Mehran Nouri, Majid Nozari, Chisom Adaobi Nri-Ezedi, Fred Nugen, Ismail A Odetokun, Adesola Adenike Ogunfowokan, Tolulope R Ojo-Akosile, Iruka N Okeke, Akinkunmi Paul Okekunle, Abdulhakeem Abayomi Olorukooba, Isaac Iyinoluwa Olufadewa, Gideon Olamilekan Oluwatunase, Verner N Orish, Doris V Ortega-Altamirano, Esteban Ortiz-Prado, Uchechukwu Levi Osuagwu, Olayinka Osuolale, Amel Ouyahia, Jagadish Rao Padubidri, Anamika Pandey, Ashok Pandey, Victoria Pando-Robles, Shahina Pardhan, Romil R Parikh, Jay Patel, Shankargouda Patil, Shrikant Pawar, Prince Peprah, Arokiasamy Perianayagam, Simone Perna, Ionela-Roxana Petcu, Anil K Philip, Roman V Polibin, Maarten J Postma, Naeimeh Pourtaheri, Jalandhar Pradhan, Elton Junio Sady Prates, Dimas Ria Angga Pribadi, Nameer Hashim Qasim, Asma Saleem Qazi, Deepthi R, Venkatraman Radhakrishnan, Fakher Rahim, Mosiur Rahman, Muhammad Aziz Rahman, Shayan Rahmani, Mohammad Rahmanian, Nazanin Rahmanian, Mahmoud Mohammed Ramadan, Shakthi Kumaran Ramasamy, Sheena Ramazanu, Muhammed Ahmed Ahmed Rameto, Pramod W

Ramteke, Kritika Rana, Chhabi Lal Ranabhat, Davide Rasella, Mohammad-Mahdi Rashidi, Ashkan Rasouli-Saravani, Devarajan Rathish, Santosh Kumar Rauniyar, Salman Rawaf, Elrashdy Moustafa Mohamed Redwan, Aavishkar Raj Regmi, Kannan RR Rengasamy, Nazila Rezaei, Nima Rezaei, Mohsen Rezaeian, Abanoub Riad, Monica Rodrigues, Jefferson Antonio Buendia Rodriguez, Leonardo Roever, Ravi Rohilla, Luca Ronfani, Moustaq Karim Khan Rony, Allen Guy Ross, Shekoufeh Roudashti, Bedanta Roy, Tilleye Runghien, Mamta Sachdeva Dhingra, Basema Ahmad Saddik, Erfan Sadeghi, Mehdi Safari, Soumya Swaroop Sahoo, S. Mohammad Sajadi, Afeez Abolarinwa Salami, Mohamed A Saleh, Hossein Samadi Kafil, Yoseph Leonardo Samodra, Juan Sanabria, Rama Krishna Sanjeev, Tanmay Sarkar, Benn Sartorius, Brijesh Sathian, Maheswar Satpathy, Monika Sawhney, Austin E Schumacher, Mengistu Abayneh Sebsibe, Dragos Serban, Mahan Shafie, Samiah Shahid, Wajeehah Shahid, Masood Ali Shaikh, Sunder Sham, Muhammad Aaqib Shamim, Mehran Shams-Beyranvand, Mohammad Ali Shamshirgaran, Mohd Shanawaz, Mohammed Shannawaz, Amin Sharifan, Manoj Sharma, Vishal Sharma, Suchitra M Shenoy, Samendra P Sherchan, Mahabalesh Shetty, Pavanchand H Shetty, Desalegn Shiferaw, Aminu Shittu, Seyed Afshin Shorofi, Emmanuel Edwar Siddig, Luís Manuel Lopes Rodrigues Silva, Baljinder Singh, Jasvinder A Singh, Robert Sinto, Bogdan Socea, Heidi M Soeters, Anton Sokhan, Prashant Sood, Soroush Soraneh, Chandrashekhar T Sreeramareddy, Suresh Kumar Srinivasamurthy, Vijay Kumar Srivastava, Muhammad Haroon Stanikzai, Narayan Subedi, Vetriselvan Subramaniyan, Sahabi K Sulaiman, Muhammad Suleman, Chandan Kumar Swain, Lukasz Szarpak, Sree Sudha T Y, Seyyed Mohammad Tabatabaei, Celine Tabche, Zanan Mohammed-Ameen Taha, Ashis Talukder, Jacques Lukenze Tamuzi, Ker-Kan Tan, Sarmila Tandukar, Mohamad-Hani Temsah, Ocean Thakali, Ramna Thakur, Sathish Thirunavukkarasu, Joe Thomas, Nikhil Kenny Thomas, Jansje Henny Vera Ticoalu, Krishna Tiwari, Marcos Roberto Tovani-Palone, Khai Hoan Tram, An Thien Tran, Nghia Minh Tran, Thang Huu Tran, Samuel Joseph Tromans, Thien Tan Tri Tai Truyen, Munkhtuya Tumurkhuu, Aniefiok John Udoakang, Arit Udoh, Saeed Ullah, Muhammad Umair, Muhammad Umar, Brigid Unim, Bhaskaran Unnikrishnan, Sanaz Vahdati, Asokan Govindaraj Vaithinathan, Rohollah Valizadeh, Madhur Verma, Georgios-Ioannis Verras, Manish Vinayak, Yasir Waheed, Mandaras Tariku Walde, Yanzhong Wang, Muhammad Waqas, Kosala Gayan Weerakoon, Nuwan Darshana Wickramasinghe, Asrat Arja Wolde, Felicia Wu, Sajad Yaghoubi, Sanni Yaya, Saber Yezli, Vahit Yiğit, Dehui Yin, Dong Keon Yon, Naohiro Yonemoto, Hadiza Yusuf, Mondal Hasan Zahid, Fathiah Zakham, Leila Zaki, Iman Zare, Michael Zastrozhin, Mohammed G M Zeariya, Haijun Zhang, Zhi-Jiang Zhang, Abzal Zhumagaliuly, Hafsa Zia, Mohammad Zoladl, Ali H Mokdad, Stephen S Lim, Theo Vos, James A Platts-Mills, Jonathan F Mosser, Robert C Reiner Jr., Simon I Hay, Mohsen Naghavi, and Christopher J L Murray.

## Affiliations

Institute for Health Metrics and Evaluation (H H Kyu PhD, A Vongpradith BA, R V Dominguez BS, S B Albertson BS, A Novotney MPH, C E Troeger MPH, J R Ledesma MPH, S B Sirota MA, R G Bender MSc, L R Swetschinski MSc, M Cunningham MSc, S Spearman MS, A Y Aravkin PhD, M B Arndt PhD, Prof M Brauer DSc, X Dai PhD, Prof L Dandona MD, Prof R Dandona PhD, E B Hamilton MPH, J He MSc, K S Ikuta MD, T Mestrovic PhD, V Mougin BA, T Runghien MSc, A E Schumacher PhD, A A Wolde MPH, Prof A H Mokdad PhD, Prof S S Lim PhD, Prof T Vos PhD, J F Mosser MD, R C Reiner Jr. PhD, Prof S I Hay FMedSci, Prof M Naghavi PhD, Prof C J L Murray DPhil), Department of Health Metrics Sciences, School of Medicine (H H

Kyu PhD, A Y Aravkin PhD, X Dai PhD, Prof R Dandona PhD, B Sartorius PhD, Prof A H Mokdad PhD, Prof S S Lim PhD, Prof T Vos PhD, R C Reiner Jr. PhD, Prof S I Hay FMedSci, Prof M Naghavi PhD, Prof C J L Murray DPhil), Department of Global Health (I A Khalil MD, M B Arndt PhD, S Kochhar MD), Department of Applied Mathematics (A Y Aravkin PhD), School of Health Systems and Public Health (C Iwu MPH), Division of Allergy and Infectious Diseases (K Tram MD), Department of Epidemiology (H Zia BDS), University of Washington, Seattle, WA, USA; Center for Biostatistics (J Ma MS), Ohio State University, Columbus, OH, USA; Urban Indian Health Institute (M C Doxey MPH), Seattle Indian Health Board, Seattle, WA, USA; School of Medicine (R G Bender MSc), Department of Radiology and Biomedical Imaging (X Liu PhD), Department of Genetics (S Pawar PhD), Yale University, New Haven, CT, USA; Department of Clinical Governance and Quality Improvement (Y H Abate MSc), Aleta Wondo General Hospital, Aleta Wondo, Ethiopia; College of Pharmacy (A H A Abd Al Magied BPharm), Ajman University, Ajman, United Arab Emirates; Department of Epidemiology (S Abd ElHafeez DrPH), Biomedical Informatics and Medical Statistics Department (I El Sayed PhD), Tropical Health Department (R M Ghazy PhD), Alexandria University, Alexandria, Egypt; Department of Medicine (Prof M Abdoun PhD), University of Sétif Algeria, Sétif, Algeria; Department of Health, Sétif, Algeria (Prof M Abdoun PhD); Department of Medical Microbiology (B Abera MSc, A D Habteyohannes PhD), Department of Midwifery (Prof S A Bante MSc), Bahir Dar University, Bahir Dar, Ethiopia; Department of Biology (B Abera MSc), Ghent University, Ghent, Belgium; Laboratory Technology Sciences Department (H Abidi PhD), Department of Nursing (M Zoladl PhD), Yasuj University of Medical Sciences, Yasuj, Iran; Department of Family and Community Health (R G Aboagye MPH), Department of Epidemiology and Biostatistics (L A Adzigbli BSc), Institute of Health Research (D Klu PhD), Department of Microbiology and Immunology (Prof V N Orish PhD), University of Health and Allied Sciences, Ho, Ghana; Department of Biomedical Science (Y D Abtew MSc), Department of Public Health (T Mekene Meto MPH), Arba Minch University, Arba Minch, Ethiopia; Department of Nursing (H Abualruz PhD), Al Zaytoonah University of Jordan, Amman, Jordan; Department of Clinical Sciences (Prof E Abu-Gharbieh PhD, Prof M M Ramadan PhD), Clinical Sciences Department (S Adra MD, H J Barqawi MPhil, N R Dash MD), College of Medicine (Prof B A Saddik PhD, Prof M A Saleh PhD), University of Sharjah, Sharjah, United Arab Emirates (K A Altirkawi MD); Department of Biopharmaceutics and Clinical Pharmacy (Prof E Abu-Gharbieh PhD), College of Pharmacy (Prof S Aburuz PhD), University of Jordan, Amman, Jordan; Medical Research Center-Academic Health System (H J Abukhadijah MPH), Nursing & Midwifery Research Department (NMRD) (A J Nashwan PhD), Department of Geriatric and Long Term Care (B Sathian PhD), Hamad Medical Corporation, Doha, Qatar; Department of Therapeutics (Prof S Aburuz PhD), Institute of Public Health (R H Al-Rifai PhD), Department of Medical Microbiology & Immunology (Prof G Khan PhD), United Arab Emirates University, Al Ain, United Arab Emirates; School of Medicine (I Y Addo PhD), Sydney Medical School (S Islam PhD), School of Chemical & Biomolecular Engineering (E A Noman PhD), University of Sydney, Sydney, NSW, Australia; Centre for Social Research in Health (I Y Addo PhD), School of Population Health (Prof B A Saddik PhD), University of New South Wales, Sydney, NSW, Australia; Department of Obstetrics and Gynecology (V Adekanmbi PhD), Department of Family Medicine (W Lee PhD), University of Texas Medical Branch, Galveston, TX, USA; Department of Microbiology (Prof C O O Adetunji PhD), Edo State University Uzairue, Iyamho, Nigeria; Department of Pharmacology and Therapeutics (T E Adeyeoluwa PhD), Department of Environmental and Occupational Health (B S Anuoluwa MPH), Department of Microbiology (I A Anuoluwa PhD, O O Bello PhD, T C Ekundayo PhD), Department of Anatomy (G O Oluwatunase MSc), Department of Biosciences and Biotechnology (A J Udoakang PhD), University of Medical Sciences, Ondo, Ondo, Nigeria; Department of Veterinary

Medicine (T E Adeyeoluwa PhD), Department of Health Promotion and Education (S Ibitoye PhD), Department of Pharmaceutical Microbiology (Prof I N Okeke PhD), College of Medicine (A P Okekunle PhD), Faculty of Public Health (I I Olufadewa MHS), University of Ibadan, Ibadan, Nigeria; Department of Fisheries and Marine Bioscience (R K Adhikary PhD), Jashore University of Science and Technology, Jashore, Bangladesh; Research School of Population Health (R K Adhikary PhD), Australian National University, Canberra, ACT, Australia; Department of Public Health (Q Adnani PhD), Center of Excellence in Higher Education for Pharmaceutical Care Innovation (Prof M J Postma PhD), Universitas Padjadjaran (Padjadjaran University), Bandung, Indonesia; Technical Services Directorate (A A Afolabi MPH), MSI Nigeria Reproductive Choices, Abuja, Nigeria; Department of Life Sciences (M S Afzal PhD, Prof M Umair PhD), University of Management and Technology, Lahore, Pakistan; Department of Community Medicine (Prof S Afzal PhD), King Edward Memorial Hospital, Lahore, Pakistan; Department of Public Health (Prof S Afzal PhD), Public Health Institute, Lahore, Pakistan; Department of New Initiatives (Prof S B Agampodi MD), International Vaccine Institute, Seoul, South Korea; Department of Health Education and Health Promotion (F D Agide PhD), Wachemo University, Hossana, Ethiopia; School of Public Health (B O Ahinkorah MPhil), School of Nursing and Midwifery (M Chutiyami PhD), School of Life Sciences (G Liu PhD), University of Technology Sydney, Sydney, NSW, Australia; Department of Medical Biochemistry (A Ahmad PhD), Shaqra University, Shaqra, Saudi Arabia; Department of Health and Biological Sciences (S Ahmad PhD), Abasyn University, Peshawar, Pakistan; Department of Natural Sciences (S Ahmad PhD), Gilbert and Rose-Marie Chagoury School of Medicine (Prof L Roever PhD), Lebanese American University, Beirut, Lebanon; Department of Pharmacy Practice (A Ahmed PhD), Riphah Institute of Pharmaceutical Sciences, Islamabad, Pakistan; Division of Infectious Diseases and Global Public Health (IDGPH) (A Ahmed PhD), University of California San Diego, San Diego, CA, USA; Institute of Endemic Diseases (A Ahmed MSc), Unit of Basic Medical Sciences (E E Siddig MD), University of Khartoum, Khartoum, Sudan; Swiss Tropical and Public Health Institute (A Ahmed MSc), University of Basel, Basel, Switzerland; Department of Biosciences (H Ahmed PhD), COMSATS Institute of Information Technology, Islamabad, Pakistan; Department of Biological Sciences (S Ahmed PhD, A S Qazi PhD), National University of Medical Sciences (NUMS), Rawalpindi, Pakistan; Department of Internal Medicine (K Akinosoglou PhD), University of Patras, Patras, Greece; Department of Internal Medicine and Infectious Diseases (K Akinosoglou PhD), University General Hospital of Patras, Patras, Greece; Maternal and Child Health Division (E Akter MSc, S Noor MS), International Centre for Diarrhoeal Disease Research, Bangladesh, Dhaka, Bangladesh; Department of Communicable Diseases (S Al Awaidy MSc), Ministry of Health, Muscat, Oman; Middle East, Eurasia, and Africa Influenza Stakeholders Network, Muscat, Oman (S Al Awaidy MSc); Faculty of Pharmacy (Prof M M Alajlani PhD), Al-Sham Private University, Damascus, Syria; Murdoch Business School (K Alam PhD), Murdoch University, Perth, WA, Australia; Department of Medicine (A Albakri MD), Royal Jordanian Medical Services, Amman, Jordan; Department of Community and Mental Health (Prof M Albashtawy PhD), Al al-Bayt University, Mafraq, Jordan; Division of Gastroenterology and Hepatology (W A Aldhaleei MD), Mayo Clinic, Jacksonville, FL, USA; Department of Bacteriology, Immunology, and Mycology (Prof A M Algammal PhD), Suez Canal University, Ismailia, Egypt; Global Centre for Environmental Remediation (A A S Al-Gheethi PhD), University of Newcastle, Newcastle, NSW, Australia; Cooperative Research Centre for Contamination Assessment and Remediation of the Environment, Newcastle, NSW, Australia (A A S Al-Gheethi PhD); Department of Zoology (A Ali PhD), Abdul Wali Khan University Mardan, Mardan, Pakistan; Center for Biotechnology and Microbiology (S S Ali PhD, M Suleman PhD), University of Swat, Swat, Pakistan; Department of Geography (W Ali PhD), Sultan Qaboos University, Muscat, Oman; Institute of Health and Wellbeing (S M

Alif PhD, B Banik PhD), Federation University Australia, Melbourne, VIC, Australia; School of Public Health and Preventive Medicine (S M Alif PhD), Department of Epidemiology and Preventative Medicine (E K Chowdhury PhD), Monash University, Melbourne, VIC, Australia; Department of Public Health and Community Medicine (Prof S M Aljunid PhD, Prof C T Sreeramareddy MD), International Medical University, Kuala Lumpur, Malaysia; International Centre for Casemix and Clinical Coding (Prof S M Aljunid PhD), National University of Malaysia, Bandar Tun Razak, Malaysia; Independent Consultant, Amman, Jordan (S Al-Marwani MSc); Independent Consultant, Irbid, Jordan (S Al-Marwani MSc); Department of Medicine (J U Almazan PhD), Nazarbayev University, Astana, Kazakhstan; Department of Parasitology (Prof H M Al-Mekhlafi PhD), University of Malaya, Kuala Lumpur, Malaysia; Department of Parasitology (Prof H M Al-Mekhlafi PhD), Department of Emergency Medicine (M A Alsabri MD), Sana'a University, Sana'a, Yemen; College of Medicine (S Almustanyir MD), Alfaisal University, Riyadh, Saudi Arabia; Ministry of Health, Riyadh, Saudi Arabia (S Almustanyir MD); Department of Medicine (S A Alqahatni MD), Biostatics, Epidemiology, and Science Computing Department (S Yezli PhD), King Faisal Specialist Hospital & Research Center, Riyadh, Saudi Arabia; Department of Medicine (S A Alqahatni MD), Russell H. Morgan Department of Radiology and Radiological Science (A Kamireddy MD), Department of International Health (H Zhang MS), Johns Hopkins University, Baltimore, MD, USA (E Melese MD); Department of Allied Medical Sciences (A Alrawashdeh PhD), Faculty of Medicine (Prof M S I Alyahya PhD), Department of Public Health (Prof Y S Khader PhD, Prof K A Kheirallah PhD), Jordan University of Science and Technology, Irbid, Jordan; Pediatric Emergency Medicine Department (M A Alsabri MD), St. Christopher’s Hospital for Children, Philadelphia, PA, USA; Institute of Molecular Biology and Biotechnology (A Altaf PhD, S Shahid PhD), University Institute of Radiological Sciences and Medical Imaging Technology (T Ashraf MS), University Institute of Public Health (S Nargus PhD), Research Centre for Health Sciences (RCHS) (S Shahid PhD), Department of Physics (W Shahid PhD), Lahore Business School (M Umar MBA), The University of Lahore, Lahore, Pakistan; Research Group in Health Economics (Prof N Alvis-Guzman PhD), Universidad de Cartagena (University of Cartagena), Cartagena, Colombia; Research Group in Hospital Management and Health Policies (Prof N Alvis-Guzman PhD), Department of Economic Sciences (N J Alvis-Zakzuk MSc), Universidad de la Costa (University of the Coast), Barranquilla, Colombia; National Health Observatory (N J Alvis-Zakzuk MSc), National Institute of Health, Bogota, Colombia; Department of Biomedical Engineering (W A Al-Zyoud PhD), German Jordanian University, Amman, Jordan; Department of Maternal and Child Wellbeing (D A Amugsi PhD), African Population and Health Research Center, Nairobi, Kenya; Department of Cardiology (Prof C Andrei PhD), Department of General Surgery (D Serban PhD, B Socea PhD), Carol Davila University of Medicine and Pharmacy, Bucharest, Romania; Department on Immunization, Vaccines and Biologicals (S Antoni MPH), Department of Immunization, Vaccines and Biologicals (H M Soeters PhD), World Health Organization (WHO), Geneva, Switzerland; Centre for Interdisciplinary Research in Basic Sciences (CIRBSc) (S Anwar PhD), Jamia Millia Islamia, New Delhi, India; School of Chemical and Life Sciences (SCLS) (S Anwar PhD), Jamia Hamdard, New Delhi, India; London School of Hygiene and Tropical Medicine (P Anwari MSc), University of London, Ottawa, ON, Canada; School of Medicine and Public Health (G C Apostol MD), Ateneo De Manila University, Pasig City, Philippines; Inter-Agency Committee on Environmental Health (G C Apostol MD), Department of Health Philippines, Manila, Philippines; Health Management and Economics Research Center (J Arabloo PhD), School of Medicine (M Bastan MD), Department of Medical Laboratory Sciences (F Dorostkar PhD), Iran University of Medical Sciences (F Eghbali MD), Preventive Medicine and Public Health Research Center (B Eshrati PhD), Department of Ophthalmology (H Hasani MD), Eye Research Center (H Kasraei MD), Research Center of Pediatric Infectious Diseases (F

Khamesipour PhD), Department of Pediatrics (H Masoumi-Asl MD), Iran University of Medical Sciences, Tehran, Iran; College of Pharmacy (M Arafat PhD), Al Ain University, Abu Dhabi, United Arab Emirates; College of Art and Science (D Areda PhD), Ottawa University, Surprise, AZ, USA; School of Life Sciences (D Areda PhD), Arizona State University, Tempe, AZ, USA; College of Medicine and Health Sciences (B B Aregawi PhD), Department of Midwifery (M W Gebregergis MSc), Department of Medical Laboratory Sciences (H N Meles MSc), Adigrat University, Adigrat, Ethiopia; Department of Veterinary Pharmacology and Toxicology (A Aremu PhD), Department of Veterinary Public Health and Preventive Medicine (I A Odetokun PhD), University of Ilorin, Ilorin, Nigeria; Department of Environmental Health (A A Asgedom PhD), Mekelle University, Mekelle, Ethiopia; Department of Immunology (S Athari PhD), Zanjan University of Medical Sciences, Zanjan, Iran; Department of Forensic Medicine (A Atreya MD), Lumbini Medical College, Palpa, Nepal; School of Medical Laboratory Sciences (F Ayele MSc), Department of Public Health (S D Darcho MPH), Department of Clinical Pharmacy (M D Gudeta MSc), Department of Psychiatry (M T Walde MSc), Haramaya University, Harar, Ethiopia; Laboratory Sciences Department (D Azadi PhD), Arak University of Medical Sciences, Khomein, Iran; Consultant (G S Azhar PhD), The World Bank, Washington, DC, USA; Institute of Biotechnology and Genetic Engineering (S Aziz MS), The University of Agriculture, Peshawar, Pakistan; ASIDE Healthcare, Lewes, DE, USA (A Azzam MD); Faculty of Medicine (A Azzam MD), October 6 University, 6th of October City, Egypt; Department of Population Medicine (Prof G Babu PhD), QU Health Sector (M Mohammed PhD), Social and Economic Survey Research Institute (Prof A Perianayagam PhD), Qatar University, Doha, Qatar; Department of Biostatistics (P Bahrami Taghanaki MD), Department of Medical Informatics (S Tabatabaei PhD), Clinical Research Development Unit (S Tabatabaei PhD), Mashhad University of Medical Sciences, Mashhad, Iran; School of Medicine (S Bahramian MD), Heart Failure Research Center (D Narimani Davani MD), Institute for Primordial Prevention of Non Communicable Disease (PPNCD) (S Roudashti MSc), Vice Chancellery of Health (S Roudashti MSc), Isfahan University of Medical Sciences, Isfahan, Iran; Division of Biological Sciences (S Balakrishnan PhD), Tamil Nadu State Council for Science and Technology, Chennai, India; Manna Institute (B Banik PhD), University of New England, Armidale, NSW, Australia; Miller School of Medicine (M Bardhan MD), University of Miami, Miami, FL, USA; Heidelberg Institute of Global Health (HIGH) (Prof T W Bärnighausen MD, Prof S Mohammed PhD), Heidelberg University, Heidelberg, Germany; T.H. Chan School of Public Health (Prof T W Bärnighausen MD), Department of Health Policy and Oral Epidemiology (Z S Natto DrPH), Harvard University, Boston, MA, USA; Department of Public and Environmental Health (A Barrow MPH), University of The Gambia, Banjul, The Gambia; Department of Epidemiology (A Barrow MPH), Division of Pulmonary, Critical Care, and Sleep (M Beiranvand PhD), College of Medicine (M J Diaz BS), Biology & Emerging Pathogens Institute (M H Zahid PhD), University of Florida, Gainesville, FL, USA; Alpha Genomics Private Limited, Islamabad, Pakistan (Z Basharat PhD); Barcelona Institute for Global Health (Prof Q Bassat MD), Universitat de Barcelona (University of Barcelona), Barcelona, Spain; Catalan Institution for Research and Advanced Studies (ICREA), Barcelona, Spain (Prof Q Bassat MD); Non-communicable Diseases Research Center (M Bastan MD, M Keykhaei MD, S Rahmani MD, M Rashidi MD, N Rezaei MD), Department of Scientific Research (F Chichagi MD), Iranian Research Center for HIV/AIDS (IRCHA) (O Dadras PhD), Pediatric Infectious Disease Research Center (M Farahmand PhD), Department of Immunology (J Karami PhD), Students' Scientific Research Center (SSRC) (M Keykhaei MD), Children's Medical Center (Prof F Kompani MD), School of Medicine (E Meftah MD, M Merati MD), Digestive Diseases Research Institute (A Mohamadkhani PhD), Research Center for Immunodeficiencies (Prof N Rezaei PhD), Department of Neurology (M Shafie MD), Sina Hospital (A Sharifan PharmD), Tehran University of Medical Sciences, Tehran, Iran; Department of Academics (S

Basu MD), Indian Institute of Public Health, Gurgaon, India; Department of Clinical Pharmacology (Prof P P Bathini MD), Apollo Institute of Medical Sciences and Research, Hyderabad, India; Department of Microbiology (P Behzadi PhD), Islamic Azad University, Shahr-e-Qods, Iran; Infectious Disease Research Department (M B Bello PhD), Medical Genomics Research Department (Prof M Umair PhD), King Abdullah International Medical Research Center, Riyadh, Saudi Arabia (Prof A A El-Metwally PhD); Department of Veterinary Microbiology (M B Bello PhD), Department of Veterinary Public Health and Preventive Medicine (A Shittu MSc), Usmanu Danfodiyo University, Sokoto, Sokoto, Nigeria; Department of Biomedical Sciences (Prof A Beloukas PhD), National AIDS Reference Center of Southern Greece (Prof A Beloukas PhD), University of West Attica, Athens, Greece; School of Medicine (A Beran MD), Indiana University, Indianapolis, IN, USA; School of Nursing and Midwifery (D Bhandari PhD), Monash University, Clayton, VIC, Australia; School of Public Health (D Bhandari PhD), University of Adelaide, Adelaide, SA, Australia; Department of Community Medicine and Family Medicine (Prof P Bhardwaj MD), School of Public Health (Prof P Bhardwaj MD), Department of Forensic Medicine and Toxicology (Prof T Kanchan MD), Department of Pharmacology (M Shamim MBBS, K Tiwari MBBS), All India Institute of Medical Sciences, Jodhpur, India; Centre for Global Child Health (Prof Z A Bhutta PhD), Temerty Faculty of Medicine (V Chattu MD), University of Toronto, Toronto, ON, Canada; Centre of Excellence in Women & Child Health (Prof Z A Bhutta PhD), Division of Women and Child Health (J K Das MD), Aga Khan University, Karachi, Pakistan; Internal Medicine Department (H Borhany MD), Social Determinants of Health Research Center (A Kolahi MD, A Nikoobar BSc, M Rashidi MD), School of Medicine (S Rahmani MD), Student Research Committee (M Rahmanian MD), Department of Immunology (A Rasouli-Saravani PhD), Department of Health (M Safari PhD), Shahid Beheshti University of Medical Sciences, Tehran, Iran; Department of Medicine (Prof S Bouaoud DrPH), Faculty of Medicine (Prof A Ouyahia PhD), University Ferhat Abbas of Sétif, Sétif, Algeria; Department of Epidemiology and Preventive Medicine (Prof S Bouaoud DrPH), University Hospital Saadna Abdenour, Sétif, Algeria; School of Population and Public Health (Prof M Brauer DSc), Department of Pediatrics (Prof N Kissoon MD), University of British Columbia, Vancouver, BC, Canada; Department of Woman and Child Health and Public Health (D Buonsenso MD), Fondazione Policlinico Universitario A. Gemelli IRCCS (Agostino Gemelli University Polyclinic IRCCS), Rome, Italy; Global Health Research Institute (D Buonsenso MD), Università Cattolica del Sacro Cuore (Catholic University of Sacred Heart), Rome, Italy; School of Public Health Sciences (Z A Butt PhD), University of Waterloo, Waterloo, ON, Canada; Al Shifa School of Public Health (Z A Butt PhD), Al Shifa Trust Eye Hospital, Rawalpindi, Pakistan; Faculty of Health Sciences (M Çakmak Barsbay PhD), Ankara University, Ankara, Turkiye; Department of Internal Medicine (Prof L A Cámera MD), Hospital Italiano de Buenos Aires (Italian Hospital of Buenos Aires), Buenos Aires, Argentina; Board of Directors (Prof L A Cámera MD), Argentine Society of Medicine, Buenos Aires, Argentina; Department of Health Management (Direzione Sanitaria) (A Capodici MD), IRCCS Istituto Ortopedico Rizzoli, Bologna, Italy; Interdisciplinary Research Center for Health Science (A Capodici MD), Sant'Anna School of Advanced Studies, Pisa, Italy; Colombian National Health Observatory (C A Castañeda-Orjuela PhD), Instituto Nacional de Salud (National Institute of Health), Bogota, Colombia; Epidemiology and Public Health Evaluation Group (C A Castañeda-Orjuela PhD), National University of Colombia, Bogota, Colombia; College of Public Health, Medical, and Veterinary Sciences (M Cenderadewi MPHTM), James Cook University, Townsville, QLD, Australia (M Kuddus PhD); Department of Public Health (M Cenderadewi MPHTM), University of Mataram, Mataram, Indonesia; Department of Biotechnology (Prof C Chakraborty PhD), Adamas University, Kolkata, India; Institute for Skeletal Aging & Orthopedic Surgery (Prof C Chakraborty PhD), Hallym University, Chuncheon, South Korea; State Disease Investigation

Laboratory (S Chakraborty MVSc), Animal Resources Development Department, Agartala, India; Department of Community Medicine (V Chattu MD), Datta Meghe Institute of Medical Sciences, Sawangi, India; Department of Biology (A A Chaudhary PhD), Al-Imam Mohammad Ibn Saud Islamic University, Riyadh, Saudi Arabia; Division of Infectious Diseases (P R Ching MD), Virginia Commonwealth University, Richmond, VA, USA; Department of Public Health, Administration, and Social Sciences (J L Chirinos-Caceres DrPH), Cayetano Heredia University, Lima, Peru; Centre for Research Impact & Outcome (H Chopra PhD), Chitkara University, Rajpura, India; Department of Community Medicine (Prof S G Choudhari MD), Jawaharlal Nehru Medical College, Wardha, India; School of Public Health (E K Chowdhury PhD), Curtin University, Perth, WA, Australia; The Interdisciplinary Research Group on Biomedicine and Health (D Chu PhD), Faculty of Applied Sciences (D Chu PhD), VNU International School (VNUIS), Hanoi, Vietnam; Department of Paediatric Surgery (I S Chukwu BMedSc), Federal Medical Centre, Umuahia, Nigeria; Department of Diagnostic and Therapeutic Technologies (Prof N Cruz-Martins PhD), Cooperativa de Ensino Superior Politécnico e Universitário (Polytechnic and University Higher Education Cooperative), Vila Nova de Famalicão, Portugal; Institute for Research and Innovation in Health (i3S) (Prof N Cruz-Martins PhD), University of Porto, Porto, Portugal; Department of Global Public Health and Primary Care (O Dadras PhD), University of Bergen, Bergen, Norway; Public Health Foundation of India, Gurugram, India (Prof L Dandona MD, Prof R Dandona PhD, G Kumar PhD, A Pandey PhD); Department of Biostatistics (V K Kamal PhD), Indian Council of Medical Research, New Delhi, India (Prof L Dandona MD, D K Lal MD); School of Medicine (I Delgado-Enciso DSc), University of Colima, Colima, Mexico; Department of Research (I Delgado-Enciso DSc), State Cancerology Institute of Colima, IMSS-BIENESTAR, Colima, Mexico; Department of Environmental Health (B Desye MSc, M Gebrehiwot DSc), Wollo University, Dessie, Ethiopia; Chettinad Hospital & Research Institute (Prof V Devanbu MD), Chettinad Academy of Research and Education, Chennai, India; Division of Pathology (K Dhama PhD), ICAR-Indian Veterinary Research Institute, Bareilly, India; Research Department (M Dhimal PhD, B P Marasini PhD, A Pandey MPH), Nepal Health Research Council, Kathmandu, Nepal; Institute of Occupational, Social and Environmental Medicine (M Dhimal PhD), Goethe University Frankfurt, Frankfurt am Main, Germany; Department of Medicine (T C Do MD, A T Tran MD), Department of General Medicine (N N Nguyen MD), Faculty of Medicine (N N Nguyen MD), Pham Ngoc Thach University of Medicine, Ho Chi Minh City, Vietnam; Department of Epidemiology (S Dohare MD), College of Nursing and Health Sciences (M Shanawaz MD), Jazan University, Jazan, Saudi Arabia; Independent Consultant, South Plainfield, NJ, USA (O P Doshi MSc); Department of Health Policy and Management (Prof L Doshmangir PhD), Immunology Research Center (A Ghasemzadeh MD), Drug Applied Research Center (H Samadi Kafil PhD), Tabriz University of Medical Sciences, Tabriz, Iran; Department of Pharmaceutical Regulatory Affairs and Management (V S Ligade PhD), Kasturba Medical College, Mangalore (Prof B Unnikrishnan MD), Manipal Academy of Higher Education, Manipal, India (H L Dsouza MD); Department of Forensic Medicine and Toxicology (H L Dsouza MD), Kasturba Medical College Mangalore, Mangalore, India; Faculty of Science and Humanities (S Duraisamy PhD), SRM Institute of Science and Technology, Kattankulathur, India; Department of Infection and Tropical Medicine (O C Durojaiye MPH), University of Sheffield, Sheffield, United Kingdom; Department of Pediatrics (A E'mar MD), Lerner Research Institute (X Liu PhD), Cleveland Clinic, Cleveland, OH, USA; Higher School of Technology (Prof A Ed-Dra PhD), Sultan Moulay Slimane University, Beni Mellal, Morocco; School of Health Sciences (H A Edinur PhD), Universiti Sains Malaysia (University of Science Malaysia), Kubang Kerian, Malaysia; Department of Pediatric Nursing (D Efendi MSN), University of Indonesia, Depok, Indonesia; Neonatal Intensive Care Unit (D Efendi MSN), University of Indonesia Hospital, Depok, Indonesia; Advanced Nursing Department

(F Efendi PhD), Universitas Airlangga (Airlangga University), Surabaya, Indonesia; Faculty of Medicine (M Elhadi MD), University of Tripoli, Tripoli, Libya; Houston Methodist Hospital, Houston, TX, USA (M Elhadi MD); College of Public Health and Health Informatics (Prof A A El-Metwally PhD), King Saud bin Abdulaziz University for Health Sciences, Riyadh, Saudi Arabia; Department of Clinical Pathology (Prof M Elshaer PhD), Department of Cardiology (Prof M M Ramadan PhD), Faculty of Pharmacy (Prof M A Saleh PhD), Mansoura University, Mansoura, Egypt; Department of Infectious Diseases and Public Health (I Elsohaby PhD, G Fekadu PhD), City University of Hong Kong, Hong Kong, China; Department of Animal Medicine (I Elsohaby PhD), Zagazig University, Zagazig, Egypt; Department of Pediatrics (C Eltaha MD), University of Texas, Dallas, TX, USA; Department of Bacteriology and Virology (M Eslami PhD), Cancer Research Center (M Eslami PhD), Semnan University of Medical Sciences, Semnan, Iran; Department of Oral Biology (A Fahim PhD), Riphah International University, Islamabad, Pakistan; Director of the Scientific and Technological Park (I R Fakhradiyev PhD), Atchabarov Scientific-Research Institute of Fundamental and Applied Medicine (M Kulimbet MSc, A Zhumagaliuly MD), Kazakh National Medical University, Almaty, Kazakhstan; Department of Food Hygiene and Quality Control (A Fakhri-Demeshghieh DVM), University of Tehran, Tehran, Iran; Department of Veterinary Tropical Diseases (Prof F O Fasina PhD), University of Pretoria, Pretoria, South Africa; Animal Production and Health Division (EMPRES) (Prof F O Fasina PhD), Food and Agriculture Organization of the United Nations, Rome, Italy; Charité University Berlin (M M Fasina MSc), Institute of Public Health (F Fischer PhD), Charité Universitätsmedizin Berlin (Charité University Medical Center Berlin), Berlin, Germany; Department of Social Medicine and Epidemiology (A Feizkhah MD), Guilan Road Trauma Research Center (N Rahmanian PhD), Guilan University of Medical Sciences, Rasht, Iran; Department of Pharmacy (G Fekadu PhD), Department of Nursing (G Fetensa MSc), Department of Public Health (M E Getachew MPH), Wollega University, Nekemte, Ethiopia; Department of Social Sciences (Prof N Ferreira PhD), University of Nicosia, Nicosia, Cyprus; Department of Dermatology (T Fukumoto PhD), Kobe University, Kobe, Japan; Department of Pathology (Prof B Fux PhD), Federal University of Espirito Santo, Vitória, Brazil; Department of Community Medicine (Prof M A Gadanya MD), Bayero University Kano, Kano, Nigeria; Department of Community Medicine (Prof M A Gadanya MD), Aminu Kano Teaching Hospital, Kano, Nigeria; Institute of Applied Health Sciences (S Gaihre PhD), University of Aberdeen, Aberdeen, United Kingdom; Department of Oral Biology and Experimental Dental Research (M Gajdács PhD), University of Szeged, Szeged, Hungary; Department of Food Technology (Y Galali ResM), Department of Statistics (Prof D H Kadir PhD), Salahaddin University-Erbil, Erbil, Iraq; Department of Nutrition and Dietetics (Y Galali ResM), Department of Business Administrations (Prof D H Kadir PhD), Cihan University-Erbil, Erbil, Iraq; Department of Community Medicine and Family Medicine (A P Gandhi MD), All India Institute of Medical Sciences, Nagpur, India; Department of Pharmacology (Prof R K Gautam PhD), Indore Institute of Pharmacy, Indore, India; Department of Reproductive and Family Health (T G Gebremeskel PhD), Axum College of Health Science, Axum, Ethiopia; College of Medicine and Public Health (T G Gebremeskel PhD), Department of Nursing and Health Sciences (S Shorofi PhD), Flinders University, Adelaide, SA, Australia; Department of Public Health (M E Getachew MPH), Department of Health, Behavior and Society (A T T Gizaw PhD), Institute of Health Science (A I Mohamed MSc), Department of Epidemiology (D Shiferaw MPH), Jimma University, Jimma, Ethiopia; Department of Public Health (G K Getahun MPH), Menelik II Medical and Health Science College, Addis Ababa, Ethiopia; Department of Medical Laboratory Science (M Getie MSc), Addis Ababa University, Addis Ababa, Ethiopia; Family and Community Medicine Department (R M Ghazy PhD), King Khalid University, Abha, Saudi Arabia; Departments of Radiology and Neurosurgery (S Ghozy MD), Department of Radiology (F Nugen PhD),

Department of Informatics and Radiology (S Vahdati MD), Mayo Clinic, Rochester, MN, USA; Country Office (A U Gil PhD), World Health Organization (WHO), Astana, Kazakhstan; Department of Nursing (A A Girmay MSc), Aksum University, Aksum, Ethiopia; Department of Health Systems and Policy Research (Prof M Golechha PhD), Indian Institute of Public Health, Gandhinagar, India; Department of Genetics (P Goleij MSc), Sana Institute of Higher Education, Sari, Iran; Universal Scientific Education and Research Network (USERN) (P Goleij MSc), Kermanshah University of Medical Sciences, Kermanshah, Iran; Department of Urban Public Health (Prof P N Gona PhD), University of Massachusetts Boston, Boston, MA, USA; Department of Dermatology (A Grada MD), Department of Quantitative Health Science (X Liu PhD), Department of Nutrition and Preventive Medicine (Prof J Sanabria MD), Case Western Reserve University, Cleveland, OH, USA; Post Graduate School of Public Health (G Guarducci MD), University of Siena, Siena, Italy; Faculty of Medicine Health and Human Sciences (Prof V K Gupta PhD), Australian Institute of Health Innovation (P Peprah MSc), Macquarie University, Sydney, NSW, Australia; Department of Clinical Pharmacology and Medicine (Prof N R Hadi PhD), University of Kufa, Najaf, Iraq; School of Health and Environmental Studies (Prof S Hamidi DrPH), Hamdan Bin Mohammed Smart University, Dubai, United Arab Emirates; Medical Research Unit (H Harapan PhD), Universitas Syiah Kuala (Syiah Kuala University), Banda Aceh, Indonesia; Department of Health Research Methods, Evidence, and Impact (M Hasan MPH), McMaster University, Hamilton, ON, Canada; Department of Biochemistry and Molecular Biology (M Hasan MPH), Tejgaon College, Dhaka, Bangladesh; Department of Biomedical Engineering and Public Health (S Hasan PhD), World University of Bangladesh, Dhaka, Bangladesh; Department of Pharmacy (Prof M S Hasnain PhD), Marwadi University, Rajkot, India; Public Health Department (I I Hassan PhD), Dalhatu Araf Specialist Hospital, Lafia, Nigeria; Department of Public Health (I I Hassan PhD), Federal University of Lafia, Lafia, Nigeria; Department of Medicine (M Hemmati MD), MedStar Health, Washington, DC, USA; Department of Medicine (M Hemmati MD), Georgetown University, Washington, DC, USA; Department of Microbiology (K Hezam PhD), Faculty of Applied Sciences (E A Noman PhD), Taiz University, Taiz, Yemen; School of Medicine (K Hezam PhD), Nankai University, Tianjin, China; School of Computer Science (Prof M Hosseinzadeh PhD), Duy Tan University, Da Nang, Vietnam; Jadara University Research Center (Prof M Hosseinzadeh PhD), Jadara University, Irbid, Jordan; Faculty of Medicine (J Huang MD), The Chinese University of Hong Kong, Hong Kong, China; International Master Program for Translational Science (H Huynh BS), International Ph.D. Program in Medicine (L Minh MD), Research Center for Artificial Intelligence in Medicine (L Minh MD), Taipei Medical University, Taipei, Taiwan; Division of Infectious Diseases (K S Ikuta MD), Veterans Affairs Greater Los Angeles, Los Angeles, CA, USA; West Africa RCC (O S Ilesanmi PhD), Africa Centre for Disease Control and Prevention, Abuja, Nigeria; Department of Community Medicine (O S Ilesanmi PhD), Department of Oral and Maxillofacial Surgery (A A Salami BDS), University College Hospital, Ibadan, Ibadan, Nigeria; Faculty of Medicine (I M Ilic PhD), University of Belgrade, Belgrade, Serbia; Faculty of Medical Sciences (Prof M D Ilic PhD), University of Kragujevac, Kragujevac, Serbia; Division of Gastroenterology and Hepatology (S Inamdar MD), University of Arkansas for Medical Sciences, Little Rock, AR, USA; Department of Microbiology (M A Isa PhD), Department of Clinical Pharmacy and Pharmacy Administration (H Yusuf PhD), University of Maiduguri, Maiduguri, Nigeria; Department of Biotechnology (M A Isa PhD), Sharda University, Greater Noida, India; School of Pharmacy (M Islam PhD), BRAC University, Dhaka, Bangladesh; Institute for Physical Activity and Nutrition (S Islam PhD), Deakin University, Burwood, VIC, Australia; Department of Clinical Pharmacy & Pharmacy Practice (Prof N Ismail PhD), Asian Institute of Medicine, Science and Technology, Bedong, Malaysia; Malaysian Academy of Pharmacy, Puchong, Malaysia (Prof N Ismail PhD); Department of Health Studies (K H

Jacobsen PhD), University of Richmond, Richmond, VA, USA; College of Medicine and Medical Sciences (H Jahrami PhD), Arabian Gulf University, Manama, Bahrain; Ministry of Health, Manama, Bahrain (H Jahrami PhD); Department of Leukemia (A Jain MD), The University of MD Anderson Cancer Center, Houston, TX, USA; Statistics Unit (N Jain MD), Riga Stradins University, Riga, Latvia; Department of Health and Safety (A A Jairoun PhD), Dubai Municipality, Dubai, United Arab Emirates; The World Academy of Sciences UNESCO, Trieste, Italy (Prof M Jakovljevic PhD); Shaanxi University of Technology, Hanzhong, China (Prof M Jakovljevic PhD); Department of Environmental Engineering (Prof R Jalilzadeh Yengejeh PhD), Islamic Azad University, Ahvaz, Iran; Invasive Fungi Research Center (J Javidnia PhD), Department of Biostatistics (Prof A Khalilian PhD), Department of Medical-Surgical Nursing (S Shorofi PhD), Mazandaran University of Medical Sciences, Sari, Iran; Department of Biochemistry (Prof S Jayaram MD), Government Medical College, Mysuru, India; Faculty of Veterinary Medicine (M Jokar DVM), University of Calgary, Calgary, AB, Canada; Young Researchers and Elite Club (M Jokar DVM), Islamic Azad University, Karaj, Iran; Rothschild Foundation Hospital (Prof J B Jonas MD), Institute of Molecular and Clinical Ophthalmology Basel, Paris, France; Singapore Eye Research Institute, Singapore, Singapore (Prof J B Jonas MD); Department of Gastroenterology and Hepatology (A Joseph MD), Department of Radiology (S Ramasamy MD), Stanford University, Stanford, CA, USA; Department of Community Medicine (N Joseph MD, P Mithra MD, R Motappa MD), Department of Forensic Medicine and Toxicology (Prof J Padubidri MD, P H Shetty MD), Manipal Academy of Higher Education, Mangalore, India; Department of Family Medicine and Public Health (J J Jozwiak PhD), University of Opole, Opole, Poland; Department of Bioengineering (H Kabir MSc), School of Information (F Nugen PhD), University of California Berkeley, Berkeley, CA, USA; National Centre for Epidemiology and Population Health (M M Kamal MPH), University of Sydney, Canberra, ACT, Australia; Canberra Business School (M M Kamal MPH), University of Canberra, Hawker, ACT, Australia; Division of Epidemiology and Biostatistics (V K Kamal PhD), National Institute of Epidemiology, Chennai, India; Faculty of Dentistry (K K Kanmodi MPH, A A Salami BDS), University of Puthisastra, Phnom Penh, Cambodia; Office of the Executive Director (K K Kanmodi MPH), Cephas Health Research Initiative Inc, Ibadan, Nigeria; Department of Community Medicine (S Kannan S MD), ESIC Medical College and Hospital Chennai, Chennai, India; The Hansjörg Wyss Department of Plastic and Reconstructive Surgery (R S Kantar MD), NYU Langone Health, New York, NY, USA; Cleft Lip and Palate Surgery Division (R S Kantar MD), Global Smile Foundation, Norwood, MA, USA; Laboratory Science Department (J Karami PhD), Khomein University of Medical Sciences, Khomein, Iran; Central Department of Public Health (P Karki MPH, N Subedi MPH), Department of Biotechnology (B P Marasini PhD), Kist Medical College and Teaching Hospital (A R Regmi BMedSc), Tribhuvan University, Kathmandu, Nepal; Health Policy Research Center (H Kasraei MD, M Nouri PhD), Department of Biostatistics (E Sadeghi PhD), Shiraz University of Medical Sciences, Shiraz, Iran; Public Health Foundation of India, New Delhi, India (H Kaur MPH); Halal Research Center of the Islamic Republic of Iran (IRI) (F Khamesipour PhD), Iran Food and Drug Administration, Tehran, Iran; Department of Rehabilitation Sciences (M Khan MPH), Hong Kong Polytechnic University, Hong Kong, China; Department of Medicine (Z A Khan MD), Shadan Hospital, Hyderabad, India; Department of Health (V Khanal PhD), Nepal Development Society, Chitwan, Nepal; Department of Preventable Non Communicable Disease (V Khanal PhD), Menzies School of Health Research, Alice Springs, NT, Australia; College of Health, Wellbeing and Life Sciences (Prof K Khatab PhD), Sheffield Hallam University, Sheffield, United Kingdom; College of Arts and Sciences (Prof K Khatab PhD), Ohio University, Zanesville, OH, USA; Department of Basic Medical Sciences (Prof M M Khatatbeh PhD), Yarmouk University, Irbid, Jordan; National Hepatology and Tropical Medicine Research Institute (A M Khater MD), Cairo

University, Cairo, Egypt; Department of Biochemistry (F Khidri PhD), Liaquat University Of Medical and Health Sciences, Jamshoro, Pakistan; Department of Internal Medicine (A A Khosla MD), Corewell Health East William Beaumont University Hospital, Royal Oak, MI, USA; Department of Medical Oncology (A A Khosla MD), Miami Cancer Institute, Miami, FL, USA; Graduate School of Public Health (K Kim PhD), Yonsei University, Busan, South Korea; School of Traditional Chinese Medicine (Y Kim PhD), Xiamen University Malaysia, Sepang, Malaysia; School of Health Sciences (Prof A Kisa PhD), Kristiania University College, Oslo, Norway; Department of International Health and Sustainable Development (Prof A Kisa PhD), Department of Environmental Health Sciences (S P Sherchan PhD), Tulane University, New Orleans, LA, USA; Global Healthcare Consulting, New Delhi, India (S Kochhar MD); Independent Consultant, Jakarta, Indonesia (S Kosen MD); Department of Anthropology (Prof K Krishan PhD), Institute of Forensic Science & Criminology (V Sharma PhD), Panjab University, Chandigarh, India; Department of Demography (Prof B Kuate Defo PhD), Department of Social and Preventive Medicine (Prof B Kuate Defo PhD), University of Montreal, Montreal, QC, Canada; Department of Mathematics (M Kuddus PhD), Department of Population Science and Human Resource Development (Prof M Rahman DrPH), University of Rajshahi, Rajshahi, Bangladesh; Department of Biochemistry (Prof M Kuddus PhD), College of Public Health & Health Informatics (R Kumar PhD), Department of Public Health (M G M Zeariya PhD), University of Hail, Hail, Saudi Arabia; Center of Medicine and Public Health (M Kulimbet MSc), Asfendiyarov Kazakh National Medical University, Almaty, Kazakhstan; Department of Environment and Public Health (F Kyei-Arthur PhD), University of Environment and Sustainable Development, Somanya, Ghana; Integrated Department of Epidemiology, Health Policy, Preventive Medicine and Pediatrics (Prof C Lahariya MD), Foundation for People-centric Health Systems, New Delhi, India; Centre for Health: The Specialty Practice, New Delhi, India (Prof C Lahariya MD); Faculty of Medicine (N Le MD), Department of General Medicine (V T Nguyen MD), Department of Internal Medicine (T H Tran MD), University of Medicine and Pharmacy at Ho Chi Minh City, Ho Chi Minh City, Vietnam; Department of Cardiovascular Research (N Le MD), Methodist Hospital, Merrillville, IN, USA; Department of Precision Medicine (Prof S Lee MD), Sungkyunkwan University, Suwon-si, South Korea; Department of Medicine (Prof Y Lee PhD), School of Medical Sciences (Prof Y Lee PhD), University of Science Malaysia, Kota Bharu, Malaysia; Department of Health Promotion and Health Education (M Li PhD), National Taiwan Normal University, Taipei, Taiwan; Department of Cardiology (S Liu MSc), Guiqian International General Hospital, Guiyang, China; Department of Radiology (X Liu PhD), Massachusetts General Hospital, Boston, MA, USA; Department of Internal Medicine (C Lo MD), Kirk Kerkorian School of Medicine at UNLV, Las Vegas, NV, USA; School of Medicine (Prof G Lucchetti PhD), Federal University of Juiz de Fora, Juiz de Fora, Brazil; Department of Clinical Data Science and Evidence (L Lv PhD), Novo Nordisk, Plainsboro, NJ, USA; Rama Medical College Hospital and Research Centre, Uttar Pradesh, India (K Malhotra MBBS); Institute of Applied Health Research (K Malhotra MBBS), University of Birmingham, Birmingham, United Kingdom; Rabigh Faculty of Medicine (Prof A Malik PhD), Department of Dental Public Health (Z S Natto DrPH), King Abdulaziz University, Jeddah, Saudi Arabia; Department of Nutrition and Dietetics (M Martorell PhD), Centre for Healthy Living (M Martorell PhD), University of Concepción, Concepción, Chile; Faculty of Humanities and Health Sciences (Prof R R Marzo MD), Curtin University, Sarawak, Malaysia; Jeffrey Cheah School of Medicine and Health Sciences (Prof R R Marzo MD), Monash University, Subang Jaya, Malaysia; Department of Community Medicine (M Mathur MD), Department of General Medicine (N Mathur MD), Geetanjali Medical College and Hospital, Udaipur, India; Division of Pediatric Hospital Medicine (R P Mediratta MD), Stanford University, Palo Alto, CA, USA; Department of Internal Medicine (E Melese MD), College of Medicine & Health Sciences (M A Rameto MPH), University of Gondar,

Gondar, Ethiopia; Universidad Nacional Mayor de San Marcos, Lima, Peru (W Mendoza MD); Comprehensive Cancer Center (T J Meretoja MD), Helsinki University Hospital, Helsinki, Finland; Department of Virology (F Zakham PhD), University of Helsinki, Helsinki, Finland (T J Meretoja MD); University Centre Varazdin (T Mestrovic PhD), University North, Varazdin, Croatia; Department of Paediatrics (Prof S Mettananda DPhil), University of Kelaniya, Ragama, Sri Lanka; University Paediatrics Unit (Prof S Mettananda DPhil), Colombo North Teaching Hospital, Ragama, Sri Lanka; College of Healthcare Management and Economics (V Mishra PhD), Gulf Medical University, Ajman, United Arab Emirates; Research and Development Department (V Mishra PhD), Panacea Institute of Interdisciplinary Research and Education, Varanasi, India; College of Health Science (A I Mohamed MSc), University of Hargeisa, Hargeisa, Somalia; Department of Internal Medicine (M F H Mohamed MSc), Brown University, Providence, RI, USA; Molecular Biology Unit (N S Mohamed MSc), Bio-Statistical and Molecular Biology Department (N S Mohamed MSc), Sirius Training and Research Centre, Khartoum, Sudan; Health Systems and Policy Research Unit (Prof S Mohammed PhD), Department of Community Medicine (A A Olorukooba MD), Ahmadu Bello University, Zaria, Nigeria; Clinical Epidemiology and Public Health Research Unit (L Monasta DSc, L Ronfani PhD), Burlo Garofolo Institute for Maternal and Child Health, Trieste, Italy; AI & Cyber Futures Institute (M Moni PhD), Charles Sturt University, Bathurst, NSW, Australia; Faculty of Medicine (B Sartorius PhD), The University of Queensland, Brisbane, QLD, Australia (M Moni PhD); Unit of Pharmacotherapy, Epidemiology and Economics (Prof S Mubarik PhD), University Medical Center Groningen (Prof M J Postma PhD), University of Groningen (Rijksuniversiteit Groningen), Groningen, Netherlands; Department of Epidemiology and Biostatistics (Prof S Mubarik PhD), School of Public Health (Prof Z Zhang PhD), Wuhan University, Wuhan, China; Department of Surgery (F Mulita PhD), General University Hospital of Patras, Patras, Greece; Faculty of Medicine (F Mulita PhD), University of Thessaly, Larissa, Greece; Amity Institute of Pharmacy (K Munjal PhD), Amity Institute of Public Health (M Shannawaz PhD), Amity University, Noida, India; Department of Community and Global Health (Y Munkhsaikhan MD), The University of Tokyo, Tokyo, Japan; Department of Computer Science (P Naghavi MS), University of Illinois Urbana-Champaign, Urbana, IL, USA; Department Health Services Research (G Naik MPH), University of Alabama at Birmingham, Birmingham, AL, USA; Department of Community Medicine (T S Nair MD), MOSC Medical College, Kolenchery, India; Department of Medical Laboratory Analysis (H H Najmuldeen PhD), Cihan University Sulaymaniya, Sulaymaniyah, Iraq; Department of Health Promotion (A Nazri-Panjaki MSc), Zahedan University of Medical Sciences, Zahedan, Iran; Department of Research (G Nchanji MSc), TroDDIVaT Initiative, Buea, Cameroon; Department of Microbiology and Parasitology (G Nchanji MSc), University of Buea, Buea, Cameroon; Research and Innovation Center (P Ndishimye PhD), Dalhousie University, Kigali, Rwanda; African Institute for Mathematical Sciences, Kigali, Rwanda (P Ndishimye PhD); Department of Biological Sciences (J W Ngunjiri PhD), University of Embu, Embu, Kenya; Cardiovascular Laboratory (D H Nguyen MD), Methodist Hospital, Merrillville, Merrillville, IN, USA; Department of Allergy, Immunology and Dermatology (D H Nguyen MD), Hanoi Medical University, Hanoi, Vietnam; Institute for Mental Health Policy Research (Y T Nigatu PhD), Centre for Addiction and Mental Health, Toronto, ON, Canada; Department of Public Health (V Niranjan PhD), HSE Ireland, Dublin, Ireland; Department of Public Health (V Niranjan PhD), UNICAF, Larnaca, Cyprus; Technical Department (C A Nnaji PhD), School of Public Health and Family Medicine (C A Nnaji PhD), University of Cape Town, Cape Town, South Africa; Medical Research Council Clinical Trials Unit (N M Noor MRCP), University College London, London, United Kingdom; Department of Gastroenterology (N M Noor MRCP), Cambridge University Hospitals, Cambridge, United Kingdom; Department of Statistics (S Noor MS), Shahjalal University of Science and

Technology, Sylhet, Bangladesh; Health Research Institute (M Nouri PhD), School of Medicine (S Soraneh MD), Babol University of Medical Sciences, Babol, Iran; School of Health (M Nozari PhD), Non-communicable Diseases Research Center (N Pourtaheri PhD), Bam University of Medical Sciences, Bam, Iran; Department of Paediatrics (C A Nri-Ezedi PhD), Nnamdi Azikiwe University, Awka, Nigeria; Department of Nursing Science (Prof A A Ogunfowokan PhD), Obafemi Awolowo University, Ile Ife, Nigeria; Department of Gynecology and Obstetrics (T R Ojo-Akosile MD), Department of Family and Preventive Medicine (S Thirunavukkarasu PhD), Emory University, Atlanta, GA, USA; Department of Food and Nutrition (A P Okekunle PhD), Seoul National University, Seoul, South Korea; Slum and Rural Health Initiative Research Academy (I I Olufadewa MHS), Slum and Rural Health Initiative, Ibadan, Nigeria; Department of Anatomy (G O Oluwatunase MSc), Olabisi Onabanjo University, Sagamu, Nigeria; Sickle Cell Unit (Prof V N Orish PhD), Ho Teaching Hospital, Ho, Ghana; Center for Health Systems Research (D V Ortega-Altamirano EdD), Infectious Disease Research Center (Prof V Pando-Robles PhD), National Institute of Public Health, Cuernavaca, Mexico; One Health Global Research Group (Prof E Ortiz-Prado PhD), Universidad de las Americas (University of the Americas), Quito, Ecuador; School of Medicine (U L Osuagwu PhD), Western Sydney University, Bathurst, NSW, Australia; Department of Optometry and Vision Science (U L Osuagwu PhD), University of KwaZulu-Natal, KwaZulu-Natal, South Africa; Department of Biological Sciences (O Osuolale PhD), Elizade University, Ilara-Mokin, Nigeria; Division of Infectious Diseases (Prof A Ouyahia PhD), University Hospital of Sétif, Sétif, Algeria; Research Department (A Pandey MPH), Public Health Research Society Nepal, Kathmandu, Nepal; Vision and Eye Research Institute (Prof S Pardhan PhD), Anglia Ruskin University, Cambridge, United Kingdom; Department of Epidemiology and Community Health (R R Parikh MD), University of Minnesota, Minneapolis, MN, USA; Global Health Governance Programme (J Patel BSc), College of Medicine and Veterinary Medicine (G Verras MD), University of Edinburgh, Edinburgh, United Kingdom; School of Dentistry (J Patel BSc), University of Leeds, Leeds, United Kingdom; College of Dental Medicine (Prof S Patil PhD), Roseman University of Health Sciences, South Jordan, UT, USA; Centre of Molecular Medicine and Diagnostics (COMManD) (Prof S Patil PhD), Saveetha Dental College and Hospitals (K Rengasamy PhD, M Tovani-Palone PhD), Saveetha University, Chennai, India; Department of Food, Environmental and Nutritional Sciences (Prof S Perna PhD), University of Milan, Milano, Italy; Department of Statistics and Econometrics (I Petcu PhD), Bucharest University of Economic Studies, Bucharest, Romania; School of Pharmacy (A K Philip PhD), University of Nizwa, Nizwa, Oman; Department of Epidemiology and Evidence-Based Medicine (R V Polibin PhD), I.M. Sechenov First Moscow State Medical University, Moscow, Russia; Department of Humanities and Social Sciences (Prof J Pradhan PhD), National Institute of Technology Rourkela, Rourkela, India; Department of Maternal-Child Nursing and Public Health (E J S Prates BS), Federal University of Minas Gerais, Belo Horizonte, Brazil; Health Sciences Department (D R A Pribadi MSc), Muhammadiyah University of Surakarta, Sukoharjo, Indonesia; Cihan University-Sulaimaniya Research Center (N H Qasim DSc), Cihan University-Sulaimaniya, Sulaymaniyah, Iraq; Department of Community Medicine (D R MD), ESI Post Graduate Institute of Medical Science and Research, Bengaluru, India; Department of Medical Oncology (Prof V Radhakrishnan MD), Cancer Institute (W.I.A), Chennai, India; Department of Medical Laboratory Technologies (Prof F Rahim PhD), Al-Noor Center of Research and Innovation (Prof F Rahim PhD), Alnoor University, Mousl, Iraq; Institute of Health and Wellbeing (Prof M Rahman PhD), Federation University Australia, Berwick, VIC, Australia; School of Nursing and Midwifery (Prof M Rahman PhD), La Trobe University, Melbourne, VIC, Australia; School of Nursing & Health Sciences (S Ramazanu PhD), Hong Kong Metropolitan University, Hong Kong, China; Saw Swee Hock School of Public Health (S Ramazanu PhD), Department of Surgery (K Tan PhD),

National University of Singapore, Singapore, Singapore; Department of Epidemiology (M A Rameto MPH), Jimma University, Jimma, Estonia; Department of Biotechnology (Prof P W Ramteke PhD), Hislop College, Nagpur, India; Department of Molecular Biology & Genetic Engineering (Prof P W Ramteke PhD), RTM Nagpur University, Nagpur, India; Translational Health Research Institute (K Rana PhD), Western Sydney University, Sydney, NSW, Australia; Department of Research (C L Ranabhat PhD), Eastern Scientific LLC, Richmond, KY, USA; Department of Health Promotion and Administration (C L Ranabhat PhD), Eastern Kentucky University, Richmond, KY, USA; Institute of Collective Health (Prof D Rasella PhD), Federal University of Bahia, Salvador, Brazil; Barcelona Institute for Global Health, Barcelona, Spain (Prof D Rasella PhD); Department of Family Medicine (Prof D Rathish MPH), Department of Parasitology (Prof K G Weerakoon PhD), Department of Community Medicine (N D Wickramasinghe MD), Rajarata University of Sri Lanka, Anuradhapura, Sri Lanka; Department of Global Health Policy (S K Rauniyar PhD), University of Tokyo, Tokyo, Japan; Department of Primary Care and Public Health (Prof S Rawaf MD, C Tabche MSc), The George Institute for Global Health (Prof S Yaya PhD), Imperial College London, London, United Kingdom; Academic Public Health England (Prof S Rawaf MD), Public Health England, London, United Kingdom; Department of Biological Sciences (Prof E M M Redwan PhD), King Abdulaziz University, Jeddah, Egypt; Department of Protein Research (Prof E M M Redwan PhD), Research and Academic Institution, Alexandria, Egypt; Centre for Excellence in Pharmaceutical Sciences (K Rengasamy PhD), North-West University, Potchefstroom, South Africa; Network of Immunity in Infection, Malignancy and Autoimmunity (NIIMA) (Prof N Rezaei PhD), Universal Scientific Education and Research Network (USERN), Tehran, Iran; Department of Epidemiology and Biostatistics (Prof M Rezaeian PhD), Rafsanjan University of Medical Sciences, Rafsanjan, Iran; Department of Public Health (A Riad PhD), Czech National Centre for Evidence-based Healthcare and Knowledge Translation (A Riad PhD), Masaryk University, Brno, Czech Republic; Department of Geography and Demography (M Rodrigues PhD), University of Coimbra, Coimbra, Portugal; Department of Pharmacology and Toxicology (Prof J A B Rodriguez PhD), University of Antioquia, Medellin, Colombia; Warwick Medical School (Prof J A B Rodriguez PhD), University of Warwick, Coventry, United Kingdom; Department of Clinical Research (Prof L Roever PhD), University of Sao Paulo, Ribeirão Preto, Brazil; Department of Community Medicine (R Rohilla MD), Government Medical College and Hospital, Chandigarh, India; Department of Public Health (M Rony MPH), Bangladesh Open University, Gazipur, Bangladesh; Rural Health Research Institute (Prof A G Ross MD), Charles Sturt University, Orange, NSW, Australia; Faculty of Medicine (B Roy PhD), Quest International University Perak, Ipoh, Malaysia; Nuffield Department of Medicine (T Runghien MSc, B Sartorius PhD), University of Oxford, Oxford, United Kingdom; Department of Pharmaceuticals (Prof M Sachdeva Dhingra PhD), Bihar College of Pharmacy, Patna, India; Department of Community Medicine and Family Medicine (S S Sahoo MD, M Verma MD), All India Institute of Medical Sciences, Bathinda, India; Department of Nutrition and Dietetics (Prof S Sajadi PhD), Cihan University, Erbil, Erbil, Iraq; Institute of Epidemiology and Preventive Medicine (Y L Samodra PhD), National Taiwan University, Taipei, Taiwan; Benang Merah Research Center (BMRC), Minahasa Utara, Indonesia (Y L Samodra PhD); Department of Surgery (Prof J Sanabria MD), Marshall University, Huntington, WV, USA; Department of Pediatrics (Prof R K Sanjeev MD), SRM University, Chennai, India; Department of Food Processing Technology (T Sarkar PhD), West Bengal State Council of Technical Education, Malda, India; Faculty of Health & Social Sciences (B Sathian PhD), Bournemouth University, Bournemouth, United Kingdom; UGC Centre of Advanced Study in Psychology (M Satpathy PhD), Department of Analytical and Applied Economics (C Swain MPhil), Utkal University, Bhubaneswar, India; Udyam-Global Association for Sustainable Development, Bhubaneswar, India (M Satpathy PhD);

Department of Public Health Sciences (M Sawhney PhD), University of North Carolina at Charlotte, Charlotte, NC, USA; Department of Medical Laboratory Science (M A Sebsibe MSc), Mizan-Tepi University, Mizan-Aman, Ethiopia; Fourth Department of General Surgery (D Serban PhD), Emergency University Hospital Bucharest, Bucharest, Romania; Independent Consultant, Karachi, Pakistan (M A Shaikh MD); Department of Pathology and Laboratory Medicine (S Sham MD), Northwell Health, New York, NY, USA; School of Medicine (M Shams-Beyranvand MSc), Alborz University of Medical Sciences, Karaj, Iran; Department of Pathobiology (M Shamshirgaran PhD), Shahid Bahonar University of Kerman, Kerman, Iran; Department for Evidence-based Medicine and Evaluation (A Sharifan PharmD), University for Continuing Education Krems, Krems, Austria; Department of Social and Behavioral Health (Prof M Sharma PhD), University of Nevada Las Vegas, Las Vegas, NV, USA; Department of Microbiology (S M Shenoy MD), Kasturba Medical College, Mangalore, India; Department of Biology (S P Sherchan PhD), Morgan State University, Baltimore, MD, USA; K S Hegde Medical Academy (Prof M Shetty MD), Nitte University, Mangalore, India; Department of Public Health (D Shiferaw MPH), Dambi Dollo University, Dembi Dollo, Ethiopia; Department of Medical Microbiology and Infectious Diseases (E E Siddig MD), Erasmus University, Rotterdam, Netherlands; Sport Physical Activity and Health Research & Innovation Center (SPRINT) (Prof L M R Silva PhD), Polytechnic Institute of Guarda, Guarda, Portugal; CICS-UBI Health Sciences Research Center (Prof L M R Silva PhD), University of Beira Interior, Covilhã, Portugal; Department of Biochemistry (B Singh PhD), Central University of Punjab, Bathinda, India; School of Medicine (Prof J A Singh MD), Baylor College of Medicine, Houston, TX, USA; Department of Medicine Service (Prof J A Singh MD), US Department of Veterans Affairs (VA), Houston, TX, USA; Department of Internal Medicine (R Sinto MD), University of Indonesia, Jakarta Pusat, Indonesia; Department of Internal Medicine (R Sinto MD), Dr. Cipto Mangunkusumo National Hospital, Jakarta Pusat, Indonesia; Department of Surgery (B Socea PhD), "Sf. Pantelimon" Emergency Clinical Hospital Bucharest, Bucharest, Romania; Department of Infectious Diseases (Prof A Sokhan PhD), Kharkiv National Medical University, Kharkiv, Ukraine; Clinical Science Line (Prof A Sokhan PhD), Ludwig Boltzmann Institute of Osteologie, Vienna, Austria; Department of Microbiology (P Sood PhD), All India Institute of Medical Sciences, Bilaspur, India; Student Research Committee (S Soraneh MD), Urmia University of Medical Sciences, Urmia, Iran (R Valizadeh PhD); Department of Pharmacology (S Srinivasamurthy MD), RAK Medical and Health Sciences University, Ras Al Khaimah, United Arab Emirates; Institute of Science (V K Srivastava PhD), Nirma University, Ahmedabad, India; Department of Public Health (M Stanikzai MPH), Kandahar University, Kandahar, Afghanistan; School of Exercise and Nutrition Sciences (N Subedi MPH), Deakin University, Melbourne, VIC, Australia; Department of Medical Sciences (Prof V Subramaniyan PhD), Sunway University, Subang Jaya, Malaysia; Department of Medicine (S K Sulaiman MD), Yobe State University Teaching Hospital, Yobe, Nigeria; School of Life Sciences (M Suleman PhD), Xiamen University, Xiamen, China; Collegium Medicum (Prof L Szarpak PhD), The John Paul II Catholic University of Lublin, Lublin, Poland; Department of Clinical Research and Development (Prof L Szarpak PhD), LUXMED Group, Warsaw, Poland; Department of Pharmacology (S T Y MD), All India Institute of Medical Sciences, Deoghar, India; Duhok Research Centre (Z M Taha BMedSc), University of Duhok, Duhok, Iraq; National Centre for Epidemiology and Population Health (A Talukder MSc), Australian National University, Acton, ACT, Australia; Statistics Discipline (A Talukder MSc), Khulna University, Khulna, Bangladesh; Department of Epidemiology (J L Tamuzi MSc), Stellenbosch University, Cape Town, South Africa; Department of Medicine (J L Tamuzi MSc), Northlands Medical Group, Omuthiya, Namibia; Science, Technology, and Natural Resources Department (S Tandukar PhD), Policy Research Institute, Kathmandu, Nepal; Pediatric Intensive Care Unit (Prof M Temsah MD), King Saud University, Riyadh, Saudi Arabia; University of

Ottawa, Ottawa, ON, Canada (O Thakali PhD); School of Humanities and Social Sciences (R Thakur PhD), Indian Institute of Technology Mandi, Mandi, India; Department of Global Health (Prof J Thomas PhD), Sustainable Policy Solutions Foundation, South Yatta, VIC, Australia; Department of Gastroenterology (N K Thomas MD), St. Luke's Hospital, Patanamthitta, India; Faculty of Public Health (J H V Ticoalu MPH), Universitas Sam Ratulangi (Sam Ratulangi University), Manado, Indonesia; Department of Health (N M Tran MD), Children's Hospital 1, Ho Chi Minh City, Vietnam; Department of Business Analytics (T H Tran MD), University of Massachusetts Dartmouth, Dartmouth, MA, USA; Department of Health Sciences (S J Tromans PhD), University of Leicester, Leicester, United Kingdom; Adult Learning Disability Service (S J Tromans PhD), Leicestershire Partnership National Health Service Trust, Leicester, United Kingdom; Faculty of Medicine (T T Truyen MD), Nam Can Tho University, Can Tho, Vietnam; Department of Internal Medicine (M Tumurkhuu PhD), Wake Forest University, Winston-Salem, NC, USA; Faculty of Health and Life Sciences (A Udoh PhD), University of Exeter, Exeter, United Kingdom; International Center for Chemical and Biological Sciences (S Ullah MSc), University of Karachi, Karachi, Pakistan; Department of Cardiovascular, Endocrine-metabolic Diseases and Aging (B Unim PhD), National Institute of Health, Rome, Italy; College of Health and Sport Sciences (A G Vaithinathan MSc), University of Bahrain, Zallaq, Bahrain; Department of Surgery (G Verras MD), University of Southampton, Southampton, United Kingdom; Department of Cardiology (M Vinayak MD), Icahn School of Medicine at Mount Sinai, New York, NY, USA; NUST School of Health Sciences (Prof Y Waheed PhD), National University of Sciences and Technology (NUST), Islamabad, Pakistan; Operational Research Center in Healthcare (Prof Y Waheed PhD), Near East University, Nicosia, Turkiye; School of Life Course and Population Sciences (Prof Y Wang PhD), King's College London, London, United Kingdom; Key Laboratory of Computer-Aided Drug Design (M Waqas PhD), Guangdong Medical University, Dongguan, China; Department of Biotechnology and Genetic Engineering (M Waqas PhD), Hazara University Mansehra, Mansehra, Pakistan; National Data Management Center for Health (NDMC) (A A Wolde MPH), Ethiopian Public Health Institute, Addis Ababa, Ethiopia; Department of Food Science and Human Nutrition (Prof F Wu PhD), Michigan State University, East Lansing, MI, USA; Department of Basic Medical Sciences (S Yaghoubi PhD), Neyshabur University of Medical Sciences, Neyshabur, Iran; Department of Health Management (V Yiğit PhD), Süleyman Demirel Üniversitesi (Süleyman Demirel University), Isparta, Turkiye; Department of Epidemiology (D Yin DrPH), Xuzhou Medical University, Xuzhou, China; Department of Pediatrics (Prof D Yon MD), Kyung Hee University, Seoul, South Korea; Department of Biostatistics (Prof N Yonemoto PhD), University of Toyama, Toyama, Japan; Department of Public Health (Prof N Yonemoto PhD), Juntendo University, Tokyo, Japan; Faculty of Medicine and Health Sciences (F Zakham PhD), Hodeidah University, Hodeidah, Yemen; Department of Parasitology and Entomology (L Zaki PhD), Tarbiat Modares University, Tehran, Iran; Research and Development Department (I Zare BSc), Sina Medical Biochemistry Technologies, Shiraz, Iran; Department of Bioengineering and Therapeutical Sciences (Prof M Zastrozhin PhD), University of California San Francisco, San Francisco, CA, USA; Department of Administration (Prof M Zastrozhin PhD), PGxAI, San Francisco, CA, USA; Department of Zoology and Entomology (M G M Zeariya PhD), Al-Azhar University, Cairo, Egypt; School of Public Health (H Zhang MS), Peking University, Beijing, China; Institute of Public Health and Social Sciences (H Zia BDS), Khyber Medical University, Peshawar, Pakistan; Division of Infectious Diseases and International Health (J A Platts-Mills MD), University of Virginia, Charlottesville, VA, USA

## Authors' Contributions

### Managing the overall research enterprise

Simon I Hay, Hmwe Hmwe Kyu, Jonathan F Mosser, Christopher J L Murray, Mohsen Naghavi, Amanda Novotney, and Robert C Reiner Jr.

### Writing the first draft of the manuscript

Hmwe Hmwe Kyu

### Primary responsibility for applying analytical methods to produce estimates

Samuel B Albertson, Rose Grace Bender, Matthew Cunningham, Matthew C Doxey, Jianing Ma, Sandra Spearman, Lucien R Swetschinski, Christopher E Troeger, and Avina Vongpradith.

### Primary responsibility for seeking, cataloguing, extracting, or cleaning data; designing or coding figures and tables

Samuel B Albertson, Regina-Mae Villanueva Dominguez, Jorge R Ledesma, Jianing Ma, Sarah Brooke Sirota, Sandra Spearman, Christopher E Troeger, and Avina Vongpradith.

### Providing data or critical feedback on data sources

Yohannes Habtegiorgis Abate, Samar Abd ElHafeez, Bayeh Abera, Hassan Abidi, Richard Gyan Aboagye, Yonas Derso Abtew, Victor Adekanmbi, Temitayo Esther Adeyeoluwa, Qorinah Estiningtyas Sakilah Adnani, Leticia Akua Adzigbli, Muhammad Sohail Afzal, Saira Afzal, Feleke Doyore Agide, Bright Opoku Ahinkorah, Sajjad Ahmad, Ali Ahmed, Ayman Ahmed, Haroon Ahmed, Saeed Ahmed, Salah Al Awaidy, Muaaz M Alajlani, Almaza Albakri, Mohammed Albashtawy, Abdelazeem M Algammal, Abid Ali, Syed Shujait Ali, Sheikh Mohammad Alif, Syed Mohamed Aljunid, Sabah Al-Marwani, Joseph Uy Almazan, Sami Almustanyir, Rami H Al-Rifai, Awais Altaf, Nelson Alvis-Guzman, Nelson J Alvis-Zakzuk, Dickson A Amugsi, Sebastien Antoni, Saleha Anwar, Palwasha Anwari, Geminn Louis Carace Apostol, Jalal Arabloo, Akeza Awealom Asgedom, Tahira Ashraf, Seyyed Shamsadin Athari, Alok Atreya, Davood Azadi, Ahmed Y. Azzam, Biswajit Banik, Mainak Bardhan, Till Winfried Bärnighausen, Hiba Jawdat Barqawi, Amadou Barrow, Mohammad-Mahdi Bastan, Payam Behzadi, Olorunjuwon Omolaja Bello, Apostolos Beloukas, Dinesh Bhandari, Zulfiqar A Bhutta, Hamed Borhany, Souad Bouaoud, Michael Brauer, Danilo Buonsenso, Mehtap Çakmak Barsbay, Carlos A Castañeda-Orjuela, Vijay Kumar Chattu, Hitesh Chopra, Dinh-Toi Chu, Natalia Cruz-Martins, Matthew Cunningham, Xiaochen Dai, Lalit Dandona, Rakhi Dandona, Samuel Demissie Darcho, Ivan Delgado-Enciso, Vinoth Gnana Chellaiyan Devanbu, Meghnath Dhimal, Michael J Diaz, Thanh Chi Do, Regina-Mae Villanueva Dominguez, Fariba - Dorostkar, Ojas Prakashbhai Doshi, Leila Doshmangir, Haneil Larson Dsouza, Senbagam Duraisamy, Oyewole Christopher Durojaiye, Abdel Rahman E'mar, Temitope Cyrus Ekundayo, Iman El Sayed, Mohammed Elshaer, Chadi Eltaha, Majid Eslami, Ildar Ravisovich Fakhradiyev, Aliasghar Fakhri-Demeshghieh, Alireza Feizkhah, Ginenus Fekadu, Takeshi Fukumoto, Blima Fux, Muktar A Gadanya, Santosh Gaihre, Yaseen Galali, Teferi Gebru Gebremeskel, Molla Getie, Sherief Ghozy, Alem Abera Girmay, Abraham Tamirat T Gizaw, Mahaveer Golechha, Pouya Goleij, Philimon N Gona, Ayman Grada, Vivek Kumar Gupta, Erin B Hamilton, Harapan Harapan, Simon I Hay, Mehdi Hemmati, Mehdi Hosseinzadeh, Junjie Huang, Hong-Han Huynh, Segun Emmanuel Ibitoye, Olayinka Stephen Ilesanmi, Sumant Inamdar, Mustafa Alhaji Isa, Sheikh Mohammed Shariful Islam, Nahlah Elkudssiah Ismail, Haitham Jahrami, Ammar Abdulrahman Jairoun, Mihajlo Jakovljevic, Reza Jalilzadeh Yengejeh, Shubha Jayaram, Jost B Jonas, Abel Joseph, Jacek Jerzy Jozwiak,

Dler H. Hussein Kadir, Rami S Kantar, Prabin Karki, Harkiran Kaur, Mohammad Keykhaei, Yousef Saleh Khader, Faham Khamesipour, Khaled Khatab, Amir M Khater, Atulya Aman Khosla, Yun Jin Kim, Adnan Kisa, Sonali Kochhar, Soewarta Kosen, Kewal Krishan, Barthelemy Kuate Defo, G Anil Kumar, Hmwe Hmwe Kyu, Chandrakant Lahariya, Dharmesh Kumar Lal, Nhi Huu Hanh Le, Seung Won Lee, Yeong Yeh Lee, Virendra S Ligade, Stephen S Lim, Gang Liu, Xuefeng Liu, Jianing Ma, Kashish Malhotra, Roy Rillera Marzo, Hossein Masoumi-Asl, Medha Mathur, Navgeet Mathur, Rishi P Mediratta, Tesfahun Mekene Meto, Endalkachew Belayneh Melese, Walter Mendoza, Sachith Mettananda, Le Huu Nhat Minh, Prasanna Mithra, Ahmed Ismail Mohamed, Mouhand F H Mohamed, Nouh Saad Mohamed, Mustapha Mohammed, Shafiu Mohammed, Ali H Mokdad, Lorenzo Monasta, Mohammad Ali Moni, Rohith Motappa, Vincent Mougin, Sumaira Mubarik, Francesk Mulita, Christopher J L Murray, Mohsen Naghavi, Shumaila Nargus, Zuhair S Natto, Josephine W Ngunjiri, Duc Hoang Nguyen, Nhien Ngoc Y Nguyen, Van Thanh Nguyen, Chukwudi A Nnaji, Syed Toukir Ahmed Noor, Mehran Nouri, Fred Nugen, Ismail A Odetokun, Abdulhakeem Abayomi Olorukooba, Gideon Olamilekan Oluwatunase, Olayinka Osuolale, Amel Ouyahia, Jagadish Rao Padubidri, Anamika Pandey, Ashok Pandey, Shahina Pardhan, Romil R Parikh, Jay Patel, Shankargouda Patil, Shrikant Pawar, Prince Peprah, Arokiasamy Perianayagam, Simone Perna, Anil K Philip, James A Platts-Mills, Maarten J Postma, Naeimeh Pourtaheri, Jalandhar Pradhan, Elton Junio Sady Prates, Asma Saleem Qazi, Fakher Rahim, Mohammad Rahmanian, Mahmoud Mohammed Ramadan, Shakthi Kumaran Ramasamy, Muhammed Ahmed Ahmed Rameto, Chhabi Lal Ranabhat, Santosh Kumar Rauniyar, Salman Rawaf, Kannan RR Rengasamy, Nima Rezaei, Monica Rodrigues, Jefferson Antonio Buendia Rodriguez, Leonardo Roever, Luca Ronfani, Allen Guy Ross, Shekoufeh Roudashti, Tilleye Runghien, Basema Ahmad Saddik, Mehdi Safari, S. Mohammad Sajadi, Afeez Abolarinwa Salami, Juan Sanabria, Benn Sartorius, Brijesh Sathian, Maheswar Satpathy, Monika Sawhney, Mengistu Abayneh Sebsibe, Dragos Serban, Samiah Shahid, Wajeehah Shahid, Masood Ali Shaikh, Sunder Sham, Muhammad Aaqib Shamim, Mohammed Shannawaz, Amin Sharifan, Vishal Sharma, Samendra P Sherchan, Aminu Shittu, Luís Manuel Lopes Rodrigues Silva, Baljinder Singh, Jasvinder A Singh, Sarah Brooke Sirota, Heidi M Soeters, Sandra Spearman, Chandrashekhar T Sreeramareddy, Muhammad Haroon Stanikzai, Vetriselvan Subramaniyan, Muhammad Suleman, Chandan Kumar Swain, Lukasz Szarpak, Sree Sudha T Y, Seyyed Mohammad Tabatabaei, Zanan Mohammed-Ameen Taha, Ker-Kan Tan, Sarmila Tandukar, Ocean Thakali, Nikhil Kenny Thomas, Marcos Roberto Tovani-Palone, Christopher E Troeger, Muhammad Umair, Muhammad Umar, Bhaskaran Unnikrishnan, Georgios-Ioannis Verras, Avina Vongpradith, Yasir Waheed, Mandaras Tariku Walde, Felicia Wu, Sajad Yaghoubi, Sanni Yaya, Naohiro Yonemoto, Iman Zare, Michael Zastrozhin, Abzal Zhumagaliuly, and Mohammad Zoladl.

## Developing methods or computational machinery

Qorinah Estiningtyas Sakilah Adnani, Saira Afzal, Ali Ahmed, Samuel B Albertson, Abdelazeem M Algammal, Walid A Al-Zyoud, Aleksandr Y Aravkin, Michael Benjamin Arndt, Ahmed Y. Azzam, Mohammad-Mahdi Bastan, Hamed Borhany, Michael Brauer, Mehtap Çakmak Barsbay, Hitesh Chopra, Dinh-Toi Chu, Xiaochen Dai, Thanh Chi Do, Matthew C Doxey, Muktar A Gadanya, Sherief Ghozy, Alem Abera Girmay, Abraham Tamirat T Gizaw, Erin B Hamilton, Simon I Hay, Jiawei He, Mehdi Hosseinzadeh, Hong-Han Huynh, Haitham Jahrami, Ibrahim A Khalil, Atulya Aman Khosla, Adnan Kisa, Chandrakant Lahariya, Nhi Huu Hanh Le, Medha Mathur, Endalkachew Belayneh Melese, Le Huu Nhat Minh, Ali H Mokdad, Mohammad Ali Moni, Vincent Mougin, Francesk Mulita, Christopher J L Murray, Mohsen Naghavi, Shumaila Nargus, Josephine W Ngunjiri, Van Thanh Nguyen, Amel Ouyahia, James A Platts-Mills, Shakthi Kumaran Ramasamy, Muhammed Ahmed Ahmed Rameto, Chhabi Lal Ranabhat, Robert C

Reiner Jr., Kannan RR Rengasamy, Monica Rodrigues, Tilleye Runghien, Mehdi Safari, Maheswar Satpathy, Austin E Schumacher, Mengistu Abayneh Sebsibe, Mohammed Shannawaz, Samendra P Sherchan, Seyed Afshin Shorofi, Sarah Brooke Sirota, Sandra Spearman, Muhammad Suleman, Chandan Kumar Swain, Sree Sudha T Y, Christopher E Troeger, Munkhtuya Tumurkhuu, Muhammad Umair, Avina Vongpradith, Theo Vos, and Mohammed G M Zeariya.

### Providing critical feedback on methods or results

Yohannes Habtegiorgis Abate, Abdallah H A Abd Al Magied, Samar Abd ElHafeez, Meriem Abdoun, Hassan Abidi, Richard Gyan Aboagye, Yonas Derso Abtew, Hasan Abualruz, Eman Abu-Gharbieh, Hana J Abukhadijah, Salahdein Aburuz, Isaac Yeboah Addo, Victor Adekanmbi, Temitayo Esther Adeyeoluwa, Ripon Kumar Adhikary, Qorinah Estiningtyas Sakilah Adnani, Saryia Adra, Leticia Akua Adzigbli, Aanuoluwapo Adeyimika Afolabi, Muhammad Sohail Afzal, Saira Afzal, Suneth Buddhika Agampodi, Feleke Doyore Agide, Bright Opoku Ahinkorah, Aqeel Ahmad, Sajjad Ahmad, Ali Ahmed, Ayman Ahmed, Haroon Ahmed, Saeed Ahmed, Karolina Akinosoglou, Ema Akter, Salah Al Awaidy, Muaaz M Alajlani, Khurshid Alam, Almaza Albakri, Mohammed Albashtawy, Abdelazeem M Algammal, Adel Ali Saeed Al-Gheethi, Abid Ali, Syed Shujait Ali, Waad Ali, Sheikh Mohammad Alif, Syed Mohamed Aljunid, Sabah Al-Marwani, Joseph Uy Almazan, Hesham M Al-Mekhlafi, Sami Almustanyir, Saleh A Alqahatni, Rami H Al-Rifai, Mohammed A Alsabri, Awais Altaf, Khalid A Altirkawi, Nelson Alvis-Guzman, Nelson J Alvis-Zakzuk, Mohammad Sharif Ibrahim Alyahya, Walid A Al-Zyoud, Dickson A Amugsi, Catalina Liliana Andrei, Boluwatife Stephen Anuoluwa, Iyadunni Adesola Anuoluwa, Saleha Anwar, Palwasha Anwari, Geminn Louis Carace Apostol, Jalal Arabloo, Mosab Arafat, Demelash Areda, Michael Benjamin Arndt, Akeza Awealom Asgedom, Tahira Ashraf, Seyyed Shamsadin Athari, Alok Atreya, Firayad Ayele, Davood Azadi, Gulrez Shah Azhar, Shahkaar Aziz, Ahmed Y. Azzam, Giridhara Rathnaiah Babu, Pegah Bahrami Taghanaki, Saeed Bahramian, Senthilkumar Balakrishnan, Biswajit Banik, Simachew Animen Bante, Mainak Bardhan, Till Winfried Bärnighausen, Hiba Jawdat Barqawi, Amadou Barrow, Zarrin Basharat, Quique Bassat, Mohammad-Mahdi Bastan, Saurav Basu, Payam Behzadi, Olorunjuwon Omolaja Bello, Apostolos Beloukas, Rose Grace Bender, Dinesh Bhandari, Pankaj Bhardwaj, Zulfiqar A Bhutta, Hamed Borhany, Souad Bouaoud, Danilo Buonsenso, Zahid A Butt, Mehtap Çakmak Barsbay, Luis Alberto Cámera, Angelo Capodici, Carlos A Castañeda-Orjuela, Chiranjib Chakraborty, Vijay Kumar Chattu, Fatemeh Chichagi, Jesus Lorenzo Chirinos-Caceres, Hitesh Chopra, Sonali Gajanan Choudhari, Enayet Karim Chowdhury, Dinh-Toi Chu, Isaac Sunday Chukwu, Muhammad Chutiyami, Natalia Cruz-Martins, Omid Dadras, Xiaochen Dai, Lalit Dandona, Rakhi Dandona, Samuel Demissie Darcho, Jai K Das, Nihar Ranjan Dash, Ivan Delgado-Enciso, Belay Desye, Vinoth Gnana Chellaiyan Devanbu, Kuldeep Dhama, Meghnath Dhimal, Michael J Diaz, Thanh Chi Do, Regina-Mae Villanueva Dominguez, Fariba - Dorostkar, Ojas Prakashbhai Doshi, Leila Doshmangir, Haneil Larson Dsouza, Senbagam Duraisamy, Oyewole Christopher Durojaiye, Abdel Rahman E’mar, Abdelaziz Ed-Dra, Hisham Atan Edinur, Defi Efendi, Ferry Efendi, Foolad Eghbali, Temitope Cyrus Ekundayo, Iman El Sayed, Muhammed Elhadi, Ashraf A El-Metwally, Mohammed Elshaer, Ibrahim Elsohaby, Chadi Eltaha, Babak Eshrati, Majid Eslami, Ayesha Fahim, Ildar Ravisovich Fakhradiyev, Aliasghar Fakhri-Demeshghieh, Mohammad Farahmand, Alireza Feizkhah, Ginenus Fekadu, Getahun Fetensa, Florian Fischer, Takeshi Fukumoto, Blima Fux, Santosh Gaihre, Márió Gajdács, Yaseen Galali, Aravind P Gandhi, Rupesh K Gautam, Miglas Welay Gebregergis, Mesfin Gebrehiwot, Teferi Gebru Gebremeskel, Motuma Erena Getachew, Genanew K Getahun, Molla Getie, Afsaneh Ghasemzadeh, Ramy Mohamed Ghazy, Sherief Ghozy, Alem Abera Girmay, Abraham Tamirat T Gizaw, Mahaveer Golechha, Philimon N Gona, Ayman Grada, Mesay Dechasa Gudeta, Vivek Kumar Gupta, Awoke Derbie Habteyohannes, Najah R Hadi, Samer Hamidi, Harapan Harapan, Md.

Kamrul Hasan, S. M. Mahmudul Hasan, Hamidreza Hasani, Md Saquib Hasnain, Ikrama Ibrahim Hassan, Simon I Hay, Jiawei He, Mehdi Hemmati, Kamal Hezam, Mehdi Hosseinzadeh, Hong-Han Huynh, Segun Emmanuel Ibitoye, Olayinka Stephen Ilesanmi, Irena M Ilic, Milena D Ilic, Sumant Inamdar, Mustafa Alhaji Isa, Md. Rabiul Islam, Sheikh Mohammed Shariful Islam, Nahlah Elkudssiah Ismail, Chidozie Declan Iwu, Kathryn H Jacobsen, Haitham Jahrami, Akhil Jain, Nityanand Jain, Ammar Abdulrahman Jairoun, Mihajlo Jakovljevic, Reza Jalilzadeh Yengejeh, Javad Javidnia, Shubha Jayaram, Mohammad Jokar, Jost B Jonas, Abel Joseph, Nitin Joseph, Jacek Jerzy Jozwiak, Dler H. Hussein Kadir, Md Moustafa Kamal, Vineet Kumar Kamal, Tanuj Kanchan, Kehinde Kazeem Kanmodi, Rami S Kantar, Jafar Karami, Prabin Karki, Harkiran Kaur, Yousef Saleh Khader, Ibrahim A Khalil, Alireza Khalilian, Faham Khamesipour, Gulfaraz Khan, Mohammad Jobair Khan, Zeeshan Ali Khan, Vishnu Khanal, Khaled Khatab, Moawiah Mohammad Khatatbeh, Amir M Khater, Feriha Fatima Khidri, Atulya Aman Khosla, Kwanghyun Kim, Yun Jin Kim, Adnan Kisa, Niranjan Kissoon, Desmond Klu, Sonali Kochhar, Ali-Asghar Kolahi, Farzad Kompani, Kewal Krishan, Barthelemy Kuate Defo, Md Abdul Kuddus, Mohammed Kuddus, G Anil Kumar, Frank Kyei-Arthur, Hmwe Hmwe Kyu, Chandrakant Lahariya, Dharmesh Kumar Lal, Nhi Huu Hanh Le, Seung Won Lee, Wei-Chen Lee, Yeong Yeh Lee, Ming-Chieh Li, Virendra S Ligade, Stephen S Lim, Gang Liu, Shuke Liu, Xiaofeng Liu, Xuefeng Liu, Chun-Han Lo, Giancarlo Lucchetti, Kashish Malhotra, Ahmad Azam Malik, Miquel Martorell, Roy Rillera Marzo, Medha Mathur, Navgeet Mathur, Rishi P Mediratta, Tesfahun Mekene Meto, Hadush Negash Meles, Endalkachew Belayneh Melese, Walter Mendoza, Mohsen Merati, Tomislav Mestrovic, Sachith Mettananda, Le Huu Nhat Minh, Vinaytosh Mishra, Prasanna Mithra, Ashraf Mohamadkhani, Mouhand F H Mohamed, Nouh Saad Mohamed, Mustapha Mohammed, Shafiu Mohammed, Ali H Mokdad, Mohammad Ali Moni, Jonathan F Mosser, Rohith Motappa, Sumaira Mubarik, Francesk Mulita, Kavita Munjal, Yanjinlkham Munkhsaikhan, Christopher J L Murray, Mohsen Naghavi, Pirouz Naghavi, Gurudatta Naik, Tapas Sadasivan Nair, Hastyar Hama Rashid Najmuldeen, Shumaila Nargus, Delaram Narimani Davani, Abdulqadir J Nashwan, Zuhair S Natto, Athare Nazri-Panjaki, G Takop Nchanji, Pacifique Ndishimye, Josephine W Ngunjiri, Duc Hoang Nguyen, Nhien Ngoc Y Nguyen, Van Thanh Nguyen, Yeshambel T Nigatu, Ali Nikoobar, Vikram Niranjan, Chukwudi A Nnaji, Efaq Ali Noman, Syed Toukir Ahmed Noor, Mehran Nouri, Majid Nozari, Chisom Adaobi Nri-Ezedi, Fred Nugen, Ismail A Odetokun, Tolulope R Ojo-Akosile, Akinkunmi Paul Okekunle, Abdulhakeem Abayomi Olorukooba, Isaac Iyinoluwa Olufadewa, Gideon Olamilekan Oluwatunase, Doris V Ortega-Altamirano, Esteban Ortiz-Prado, Uchechukwu Levi Osuagwu, Olayinka Osuolale, Amel Ouyahia, Jagadish Rao Padubidri, Anamika Pandey, Ashok Pandey, Victoria Pando-Robles, Shahina Pardhan, Romil R Parikh, Jay Patel, Shankargouda Patil, Shrikant Pawar, Prince Peprah, Arokiasamy Perianayagam, Ionela-Roxana Petcu, Anil K Philip, James A Platts-Mills, Roman V Polibin, Maarten J Postma, Naeimeh Pourtaheri, Jalandhar Pradhan, Elton Junio Sady Prates, Dimas Ria Angga Pribadi, Nameer Hashim Qasim, Asma Saleem Qazi, Deepthi R, Venkatraman Radhakrishnan, Fakher Rahim, Mosiur Rahman, Muhammad Aziz Rahman, Mohammad Rahmanian, Nazanin Rahmanian, Mahmoud Mohammed Ramadan, Shakthi Kumaran Ramasamy, Sheena Ramazanu, Muhammed Ahmed Ahmed Rameto, Pramod W Ramteke, Kritika Rana, Chhabi Lal Ranabhat, Mohammad-Mahdi Rashidi, Devarajan Rathish, Santosh Kumar Rauniyar, Salman Rawaf, Elrashdy M. Moustafa Mohamed Redwan, Aavishkar Raj Regmi, Kannan RR Rengasamy, Nazila Rezaei, Nima Rezaei, Mohsen Rezaeian, Monica Rodrigues, Leonardo Roever, Ravi Rohilla, Moustaq Karim Khan Rony, Allen Guy Ross, Tilleye Runghien, Basema Ahmad Saddik, Erfan Sadeghi, Mehdi Safari, Soumya Swaroop Sahoo, S. Mohammad Sajadi, Afeez Abolarinwa Salami, Mohamed A Saleh, Hossein Samadi Kafil, Yoseph Leonardo Samodra, Juan Sanabria, Rama Krishna Sanjeev, Tanmay Sarkar, Benn Sartorius, Brijesh Sathian, Maheswar Satpathy, Monika Sawhney,

Mengistu Abayneh Sebsibe, Dragos Serban, Mahan Shafie, Samiah Shahid, Masood Ali Shaikh, Muhammad Aaqib Shamim, Mehran Shams-Beyranvand, Mohammad Ali Shamshirgaran, Mohd Shanawaz, Mohammed Shannawaz, Amin Sharifan, Vishal Sharma, Suchitra M Shenoy, Samendra P Sherchan, Desalegn Shiferaw, Aminu Shittu, Seyed Afshin Shorofi, Emmanuel Edwar Siddig, Luís Manuel Lopes Rodrigues Silva, Baljinder Singh, Jasvinder A Singh, Sarah Brooke Sirota, Heidi M Soeters, Prashant Sood, Soroush Soraneh, Chandrashekhar T Sreeramareddy, Suresh Kumar Srinivasamurthy, Vijay Kumar Srivastava, Muhammad Haroon Stanikzai, Vetriselvan Subramaniyan, Sahabi K Sulaiman, Muhammad Suleman, Chandan Kumar Swain, Lucien R Swetschinski, Lukasz Szarpak, Sree Sudha T Y, Seyyed Mohammad Tabatabaei, Celine Tabche, Zanan Mohammed-Ameen Taha, Ashis Talukder, Jacques Lukenze Tamuzi, Ker-Kan Tan, Mohamad-Hani Temsah, Ocean Thakali, Ramna Thakur, Sathish Thirunavukkarasu, Joe Thomas, Nikhil Kenny Thomas, Jansje Henny Vera Ticoalu, Krishna Tiwari, Marcos Roberto Tovani-Palone, Khai Hoan Tram, An Thien Tran, Nghia Minh Tran, Christopher E Troeger, Samuel Joseph Tromans, Munkhtuya Tumurkhuu, Aniefiok John Udoakang, Arit Udoh, Saeed Ullah, Muhammad Umair, Bhaskaran Unnikrishnan, Sanaz Vahdati, Rohollah Valizadeh, Madhur Verma, Georgios-Ioannis Verras, Manish Vinayak, Avina Vongpradith, Yasir Waheed, Mandaras Tariku Walde, Yanzhong Wang, Muhammad Waqas, Kosala Gayan Weerakoon, Nuwan Darshana Wickramasinghe, Asrat Arja Wolde, Felicia Wu, Sanni Yaya, Saber Yezli, Vahit Yiğit, Dehui Yin, Dong Keon Yon, Naohiro Yonemoto, Hadiza Yusuf, Mondal Hasan Zahid, Fathiah Zakham, Michael Zastrozhin, Mohammed G M Zeariya, Haijun Zhang, Zhi-Jiang Zhang, Abzal Zhumagaliuly, and Mohammad Zoladl.

### Drafting the work or revising it critically for important intellectual content

Yohannes Habtegiorgis Abate, Abdallah H A Abd Al Magied, Samar Abd ElHafeez, Hassan Abidi, Hasan Abualruz, Eman Abu-Gharbieh, Hana J Abukhadijah, Salahdein Aburuz, Isaac Yeboah Addo, Victor Adekanmbi, Charles Oluwaseun Oluwaseun Adetunji, Qorinah Estiningtyas Sakilah Adnani, Saryia Adra, Aanuoluwapo Adeyimika Afolabi, Muhammad Sohail Afzal, Saira Afzal, Suneth Buddhika Agampodi, Feleke Doyore Agide, Bright Opoku Ahinkorah, Ali Ahmed, Ayman Ahmed, Haroon Ahmed, Ema Akter, Salah Al Awaidy, Muaaz M Alajlani, Khurshid Alam, Almaza Albakri, Mohammed Albashtawy, Wafa A Aldhaleei, Abdelazeem M Algammal, Abid Ali, Syed Shujait Ali, Waad Ali, Sami Almustanyir, Saleh A Alqahatni, Ahmad Alrawashdeh, Rami H Al-Rifai, Awais Altaf, Nelson Alvis-Guzman, Nelson J Alvis-Zakzuk, Mohammad Sharif Ibrahim Alyahya, Walid A Al-Zyoud, Dickson A Amugsi, Catalina Liliana Andrei, Boluwatife Stephen Anuoluwa, Iyadunni Adesola Anuoluwa, Palwasha Anwari, Geminn Louis Carace Apostol, Jalal Arabloo, Brhane Berhe Aregawi, Abdulfatai Aremu, Seyyed Shamsadin Athari, Alok Atreya, Davood Azadi, Shahkaar Aziz, Ahmed Y. Azzam, Giridhara Rathnaiah Babu, Senthilkumar Balakrishnan, Mainak Bardhan, Till Winfried Bärnighausen, Hiba Jawdat Barqawi, Amadou Barrow, Quique Bassat, Mohammad-Mahdi Bastan, Prapthi Persis Bathini, Payam Behzadi, Maryam Beiranvand, Muhammad Bashir Bello, Olorunjuwon Omolaja Bello, Apostolos Beloukas, Azizullah Beran, Dinesh Bhandari, Hamed Borhany, Souad Bouaoud, Michael Brauer, Danilo Buonsenso, Mehtap Çakmak Barsbay, Angelo Capodici, Carlos A Castañeda-Orjuela, Muthia Cenderadewi, Chiranjib Chakraborty, Sandip Chakraborty, Vijay Kumar Chattu, Anis Ahmad Chaudhary, Fatemeh Chichagi, Patrick R Ching, Jesus Lorenzo Chirinos-Caceres, Muhammad Chutiyami, Natalia Cruz-Martins, Samuel Demissie Darcho, Nihar Ranjan Dash, Ivan Delgado-Enciso, Belay Desye, Meghnath Dhimal, Michael J Diaz, Thanh Chi Do, Sushil Dohare, Regina-Mae Villanueva Dominguez, Ojas Prakashbhai Doshi, Leila Doshmangir, Matthew C Doxey, Haneil Larson Dsouza, Senbagam Duraisamy, Oyewole Christopher Durojaiye, Abdel Rahman E'mar, Iman El Sayed, Muhammed Elhadi, Ashraf A El-Metwally, Mohammed Elshaer, Chadi Eltaha, Ayesha Fahim, Aliasghar Fakhri-Demeshghieh, Folorunso Oludayo Fasina, Modupe Margaret Fasina, Nuno Ferreira, Getahun

Fetensa, Florian Fischer, Takeshi Fukumoto, Blima Fux, Muktar A Gadanya, Santosh Gaihre, Márió Gajdács, Yaseen Galali, Rupesh K Gautam, Miglas Welay Gebregergis, Teferi Gebru Gebremeskel, Molla Getie, Afsaneh Ghasemzadeh, Ramy Mohamed Ghazy, Sherief Ghozy, Artyom Urievich Gil, Alem Abera Girmay, Abraham Tamirat T Gizaw, Ayman Grada, Giovanni Guarducci, Mesay Dechasa Gudeta, Vivek Kumar Gupta, Awoke Derbie Habteyohannes, Najah R Hadi, Harapan Harapan, Md. Kamrul Hasan, S. M. Mahmudul Hasan, Hamidreza Hasani, Md Saquib Hasnain, Simon I Hay, Mehdi Hemmati, Kamal Hezam, Hong-Han Huynh, Segun Emmanuel Ibitoye, Kevin S Ikuta, Olayinka Stephen Ilesanmi, Irena M Ilic, Milena D Ilic, Md. Rabiul Islam, Sheikh Mohammed Shariful Islam, Nahlah Elkudssiah Ismail, Chidozie Declan Iwu, Kathryn H Jacobsen, Haitham Jahrami, Akhil Jain, Nityanand Jain, Mihajlo Jakovljevic, Shubha Jayaram, Jost B Jonas, Abel Joseph, Nitin Joseph, Jacek Jerzy Jozwiak, Hannaneh Kabir, Md Moustafa Kamal, Arun Kamireddy, Kehinde Kazeem Kanmodi, Suthanthira Kannan S, Rami S Kantar, Jafar Karami, Hengameh Kasraei, Ibrahim A Khalil, Gulfaraz Khan, Mohammad Jobair Khan, Zeeshan Ali Khan, Vishnu Khanal, Khaled Khatab, Moawiah Mohammad Khatatbeh, Khalid A Kheirallah, Feriha Fatima Khidri, Atulya Aman Khosla, Kwanghyun Kim, Yun Jin Kim, Adnan Kisa, Niranjan Kissoon, Desmond Klu, Sonali Kochhar, Farzad Kompani, Kewal Krishan, Barthelemy Kuate Defo, Md Abdul Kuddus, Mohammed Kuddus, Mukhtar Kulimbet, Rakesh Kumar, Frank Kyei-Arthur, Hmwe Hmwe Kyu, Chandrakant Lahariya, Nhi Huu Hanh Le, Jorge R Ledesma, Yeong Yeh Lee, Chun-Han Lo, Giancarlo Lucchetti, Lei Lv, Kashish Malhotra, Ahmad Azam Malik, Bishnu P Marasini, Miquel Martorell, Roy Rillera Marzo, Hossein Masoumi-Asl, Medha Mathur, Navgeet Mathur, Rishi P Mediratta, Elahe Meftah, Hadush Negash Meles, Endalkachew Belayneh Melese, Walter Mendoza, Mohsen Merati, Tuomo J Meretoja, Tomislav Mestrovic, Sachith Mettananda, Le Huu Nhat Minh, Prasanna Mithra, Mouhand F H Mohamed, Nouh Saad Mohamed, Mustapha Mohammed, Shafiu Mohammed, Ali H Mokdad, Lorenzo Monasta, Mohammad Ali Moni, Jonathan F Mosser, Rohith Motappa, Francesk Mulita, Yanjinlkham Munkhsaikhan, Mohsen Naghavi, Tapas Sadasivan Nair, Shumaila Nargus, Delaram Narimani Davani, Abdulqadir J Nashwan, Zuhair S Natto, G Takop Nchanji, Pacifique Ndishimye, Josephine W Ngunjiri, Duc Hoang Nguyen, Nhien Ngoc Y Nguyen, Van Thanh Nguyen, Yeshambel T Nigatu, Vikram Niranjan, Nurulamin M Noor, Mehran Nouri, Amanda Novotney, Chisom Adaobi Nri-Ezedi, Fred Nugen, Ismail A Odetokun, Adesola Adenike Ogunfowokan, Iruka N Okeke, Abdulhakeem Abayomi Olorukooba, Gideon Olamilekan Oluwatunase, Verner N Orish, Doris V Ortega-Altamirano, Esteban Ortiz-Prado, Uchechukwu Levi Osuagwu, Amel Ouyahia, Jagadish Rao Padubidri, Ashok Pandey, Victoria Pando-Robles, Shahina Pardhan, Romil R Parikh, Jay Patel, Shankargouda Patil, Shrikant Pawar, Arokiasamy Perianayagam, Ionela-Roxana Petcu, Maarten J Postma, Jalandhar Pradhan, Elton Junio Sady Prates, Nameer Hashim Qasim, Asma Saleem Qazi, Deepthi R, Venkatraman Radhakrishnan, Fakher Rahim, Shayan Rahmani, Mohammad Rahmanian, Mahmoud Mohammed Ramadan, Shakthi Kumaran Ramasamy, Muhammed Ahmed Ahmed Rameto, Kritika Rana, Chhabi Lal Ranabhat, Davide Rasella, Ashkan Rasouli-Saravani, Devarajan Rathish, Salman Rawaf, Elrashdy M. Moustafa Mohamed Redwan, Aavishkar Raj Regmi, Nazila Rezaei, Nima Rezaei, Abanoub Riad, Monica Rodrigues, Jefferson Antonio Buendia Rodriguez, Leonardo Roever, Ravi Rohilla, Luca Ronfani, Moustaq Karim Khan Rony, Allen Guy Ross, Bedanta Roy, Mamta Sachdeva Dhingra, Basema Ahmad Saddik, Mehdi Safari, Soumya Swaroop Sahoo, S. Mohammad Sajadi, Afeez Abolarinwa Salami, Hossein Samadi Kafil, Juan Sanabria, Tanmay Sarkar, Maheswar Satpathy, Mengistu Abayneh Sebsibe, Dragos Serban, Mahan Shafie, Samiah Shahid, Muhammad Aaqib Shamim, Mehran Shams-Beyranvand, Mohd Shanawaz, Mohammed Shannawaz, Amin Sharifan, Manoj Sharma, Vishal Sharma, Suchitra M Shenoy, Mahabalesh Shetty, Pavanchand H Shetty, Aminu Shittu, Seyed Afshin Shorofi, Emmanuel Edwar Siddig, Luís Manuel Lopes Rodrigues Silva, Jasvinder A Singh, Robert

Sinto, Bogdan Socea, Heidi M Soeters, Anton Sokhan, Prashant Sood, Soroush Soraneh, Chandrashekhar T Sreeramareddy, Suresh Kumar Srinivasamurthy, Muhammad Haroon Stanikzai, Narayan Subedi, Vetriselvan Subramaniyan, Muhammad Suleman, Chandan Kumar Swain, Lukasz Szarpak, Sree Sudha T Y, Celine Tabche, Zanan Mohammed-Ameen Taha, Ashis Talukder, Jacques Lukenze Tamuzi, Ker-Kan Tan, Mohamad-Hani Temsah, Ocean Thakali, Ramna Thakur, Sathish Thirunavukkarasu, Marcos Roberto Tovani-Palone, An Thien Tran, Nghia Minh Tran, Thang Huu Tran, Samuel Joseph Tromans, Thien Tan Tri Tai Truyen, Aniefiok John Udoakang, Arit Udoh, Muhammad Umair, Brigid Unim, Bhaskaran Unnikrishnan, Sanaz Vahdati, Asokan Govindaraj Vaithinathan, Madhur Verma, Georgios-Ioannis Verras, Manish Vinayak, Theo Vos, Yanzhong Wang, Kosala Gayan Weerakoon, Nuwan Darshana Wickramasinghe, Sanni Yaya, Saber Yezli, Vahit Yiğit, Dong Keon Yon, Naohiro Yonemoto, Hadiza Yusuf, Mondal Hasan Zahid, Fathiah Zakham, Leila Zaki, Iman Zare, Michael Zastrozhin, Haijun Zhang, Zhi-Jiang Zhang, Hafsa Zia, Mohammad Zoladl,

## Managing the estimation or publication process

Erin B Hamilton, Simon I Hay, Hmwe Hmwe Kyu, Ali H Mokdad, Christopher J L Murray, Mohsen Naghavi, Amanda Novotney, and Theo Vos.
